# Lack of interferon-γ receptor results in a microenvironment favorable for intestinal tumorigenesis

**DOI:** 10.18632/oncotarget.9867

**Published:** 2016-06-07

**Authors:** Caibo Zhang, Dong Hou, Haifeng Wei, Minnan Zhao, Lin Yang, Qiao Liu, Xiyu Zhang, Yaoqin Gong, Changshun Shao

**Affiliations:** ^1^ Key Laboratory of Experimental Teratology, Ministry of Education and Department of Molecular Medicine and Genetics, Shandong University School of Medicine, Jinan, Shandong, 250012, China; ^2^ Department of Life Sciences, Qilu Normal University, Jinan, Shandong, 250013, China; ^3^ First Clinical Medical College, Shandong University of Traditional Chinese Medicine, Jinan, Shandong, 250012, China; ^4^ Huaiyin People's Hospital, Jinan, Shandong, 250021, China; ^5^ Department of Genetics/Human Genetics Institute of New Jersey, Piscataway, NJ, 08854, USA

**Keywords:** IFN-γ receptor, intestinal tumorigenesis, gene expression profiling, inflammation, tumor microenvironment

## Abstract

IFN-γ plays an important role in innate and adaptive immunity. IFN-γ signaling is also involved in tumorigenesis, with both pro- and antitumor activities documented. We here report the characterization of intestinal tumorigenesis in Apc^*Min/*+^ mice that lack IFN-γ receptor. We observed that *Ifngr1*^−/−^*Apc*^Min/+^ mice are shorter-lived than *Ifngr1^+/+^Apc*^Min/+^ mice. The tumors in *Ifngr1*^−/−^*Apc*^Min/+^ mice are more likely to progress into invasive adenocarcinomas. Gene expression profiling by RNA sequencing revealed a significant upregulation of genes involved in inflammation and tissue remodeling in tumors of *Ifngr1*^−/−^*Apc*^Min/+^ mice when compared to those in *Ifngr1^+/+^Apc*^Min/+^ mice. In particular, five genes encoding matrix metallopeptidases (MMPs) were among the upregulated. On the other hand, genes that promote or maintain intestinal differentiation, such as *Cdx2*, *Cdhr2* and *Cdhr5*, were downregulated. Tumor-associated macrophages were more abundant and were more favored toward M2 polarization in *Ifngr1*^−/−^*Apc*^Min/+^ mice than in *Ifngr1^+/+^Apc*^Min/+^ mice. Furthermore, the *Ifngr1* was significantly downregulated in intestinal tumors when compared to mucosa. A similar trend was noted for human colorectal carcinomas. Together, our results indicate that adequate IFN-γ signaling is critical for maintaining a tumor-prohibitive microenvironment.

## INTRODUCTION

The *Apc^Min/^*^+^ mouse is an animal model of human familial adenomatous polyposis [1–2]. *Apc^Min/^*^+^ mice usually develop numerous adenomas in their intestinal tracts, as a result of loss or mutation of the wildtype *Apc* allele, and consequently the upregulation of β-catenin [3]. *Apc^Min/^*^+^ mice have been frequently used to study the mechanisms underlying the development of colorectal cancer (CRC) and have led to the identification of many factors that either promote or suppress intestinal tumorigenesis. Inflammatory factors are frequently studied for their roles during tumorigenesis in *Apc^Min^* mice, and increased inflammation has generally been documented to promote tumorigenesis [4–9].

IFN-γ is a pro-inflammatory cytokine that is mainly produced by T cells and natural killer (NK) cells [10]. It has been shown to participate in regulation of antiviral and antitumor immunity [11]. Mouse models deficient in IFN-γ signaling display increased tumor cell proliferation [12–13]. IFN-γ was reported to selectively induce apoptosis of tumor-initiating label-retaining colon cancer cells [14]. It could alter macrophages from M2 to M1 in *Apc^Min/^*^+^ mouse polyps [15]. However, IFN-γ signaling has also been reported to sequentially activate and inhibit β-catenin, via AKT and DKK1, respectively [16], which presumably would exert opposing effects on β-catenin-mediated tumorigenesis. There were also reports of IFN-γ acting as a pro-tumorigenic factor. For example, transgenic expression of IFN-γ in mouse stomach leads to increased inflammation and tumor formation [17]. Development of colorectal carcinomas in *Socs1*-deficient mice was also found to be dependent on IFN-γ signaling [18]. IFN-γ was recently reported to promote spontaneous papilloma and cutaneous malignant melanoma [19–20]. Importantly, IFN-γ can induce the production of immunosuppressive PD-L1 in cancer cells, which, through binding to its receptor PD-1 on T cells, may render cancer cells resistant to host immune surveillance [21]. Thus, the role of IFN-γ in tumorigenesis could be context-dependent.

In this study we generated *Ifngr1*^−/−^*Apc^Min^*^/+^ mice and studied the effect of IFN-γ receptor deficiency on the intestinal tumorigenesis. We observed that tumorigenesis is significantly accelerated in the absence of IFN-γ receptor. Gene expression profiling showed that the tumor microenvironment in *Ifngr1*^−/−^*Apc^Min/^*^+^ mice is highly inflammatory and is more permissive for tissue remodeling when compared to that in *Ifngr1*^+/+^*Apc^Min/^*^+^ mice. Correspondingly, *Ifngr1* is downregulated in intestinal tumors from *Ifngr1*^+/+^*Apc^Min/^*^+^ mice. Our results suggest that disruption of tissue homeostasis associated with a dysregulated IFN-γ signaling may enhance tumor progression.

## RESULTS

### IFN-γ receptor deficiency shortened lifespan and enhanced intestinal tumorigenesis in *Apc^Min/+^* mice

**Figure 1:**
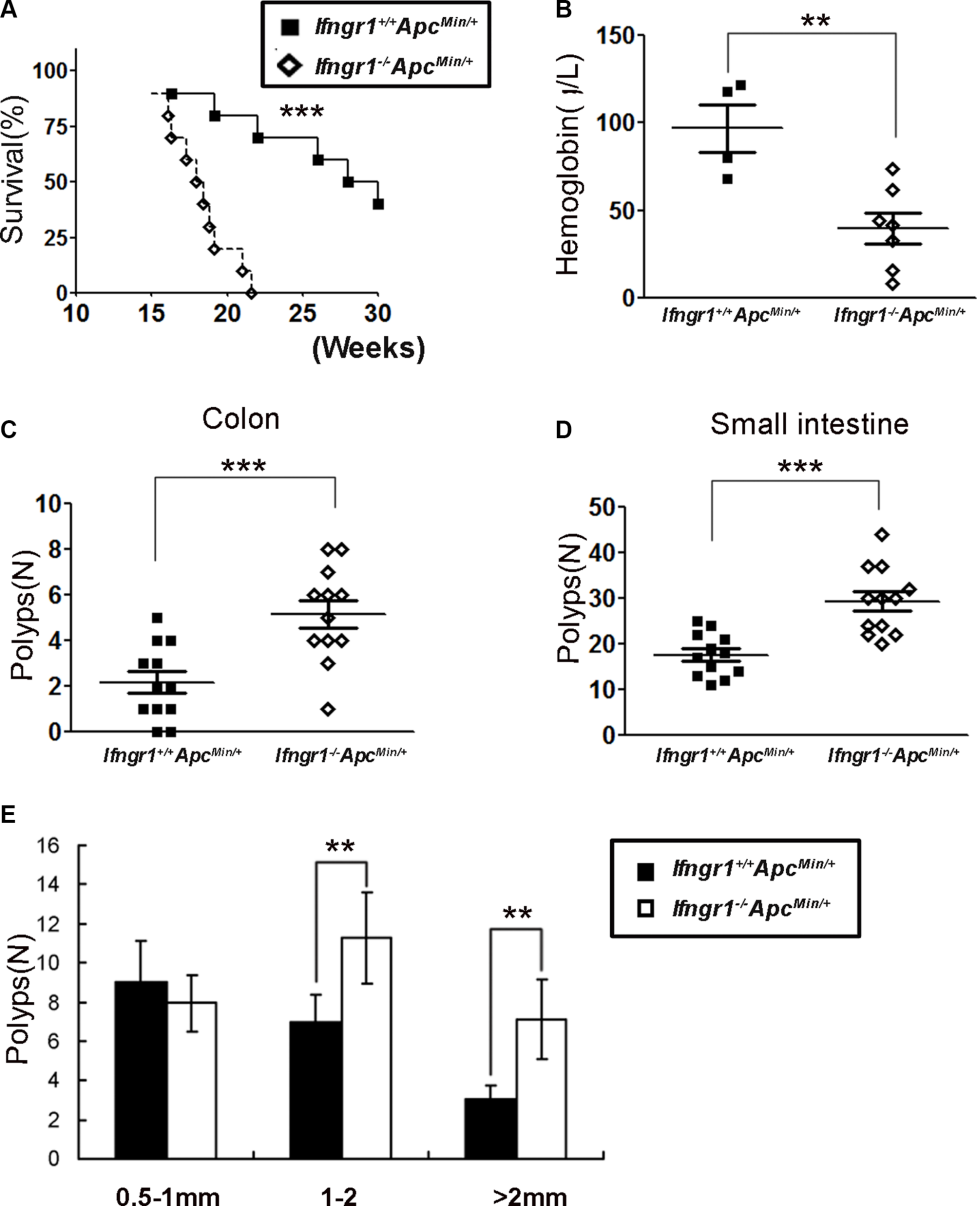
IFN-γ receptor deficiency enhanced intestinal tumorigenesis in *Apc^Min/^*^+^ mice (**A**) Decreased survival of *Ifngr1*^−/−^*Apc^Min/+^* mice (*n* = 14) when compared to *Ifngr1^+/+^Apc^Min/+^* mice (*n* = 10). (*P < 0.05, **P < 0.01, ***P < 0.005, *Mantel-Cox* test.) (**B**) Concentrations of hemoglobin in *Ifngr1^+/+^Apc^Min/+^* mice (*n* = 4) and *Ifngr1*^−/−^*Apc^Min/+^* mice (*n* = 7), measured when the mice were 18 weeks old.(*P < 0.05, **P < 0.01, ***P < 0.005, Unpaired *t* test.) (**C**, **D**) Scoring of macroadenomas (≥ 0.5 mm in diameter) in small intestines and colons of *Apc^Min/+^* mice. (**E**) Size distribution of macroadenomas in small intestines. Tumors were scored under a stereoscopic microscopy in age matched *Ifngr1^+/+^Apc^Min/+^* mice (*n* = 12) and *Ifngr1*^−/−^*Apc^Min/+^* mice (*n* = 12).(*P < 0.05, **P < 0.01, ***P < 0.005, Unpaired *t* test.).

To determine the role of IFN-γ signaling in the development of intestinal polyposis in *Apc^Min/^*^+^ mice, we introduced IFN-γ receptor deficiency into *Apc^Min/^*^+^ mice. The *Ifngr1^Min/+^Apc^Min/^*^+^ mice showed a significantly shorter lifespan than the *Ifngr1^+/+^Apc^Min/^*^+^ mice (Figure 1A). Measurement of hemoglobin levels in those mice indicated that anemia was more severe in *Ifngr1*^−/−^*Apc^Min/+^* mice (Figure 1B). Since most of the *Ifngr1*^−/−^*Apc^Min/^*^+^mice die by 20 weeks, we sacrificed the animals and scored the polyps when they were 18 weeks old. The numbers of polyps in the small intestine and colon of the *Ifngr1*^−/−^*Apc^Min/^*^+^mice were significantly greater than those in the *Ifngr1^+/+^Apc^Min/^*^+^mice (Figure 1C and 1D). In addition, the polyps in *Ifngr1*^−/−^*Apc^Min/+^* mice were larger than those in *Ifngr1*^+/+^*Apc^Min/^*^+^ mice (Figure 1E). These results suggest that intestinal tumorigenesis is accelerated in *Apc^Min/^*^+^ mice in the absence of IFN-γ receptor.

### IFN-γ receptor deficiency promotes tumor invasion in *Apc^Min/+^* mice

**Figure 2:**
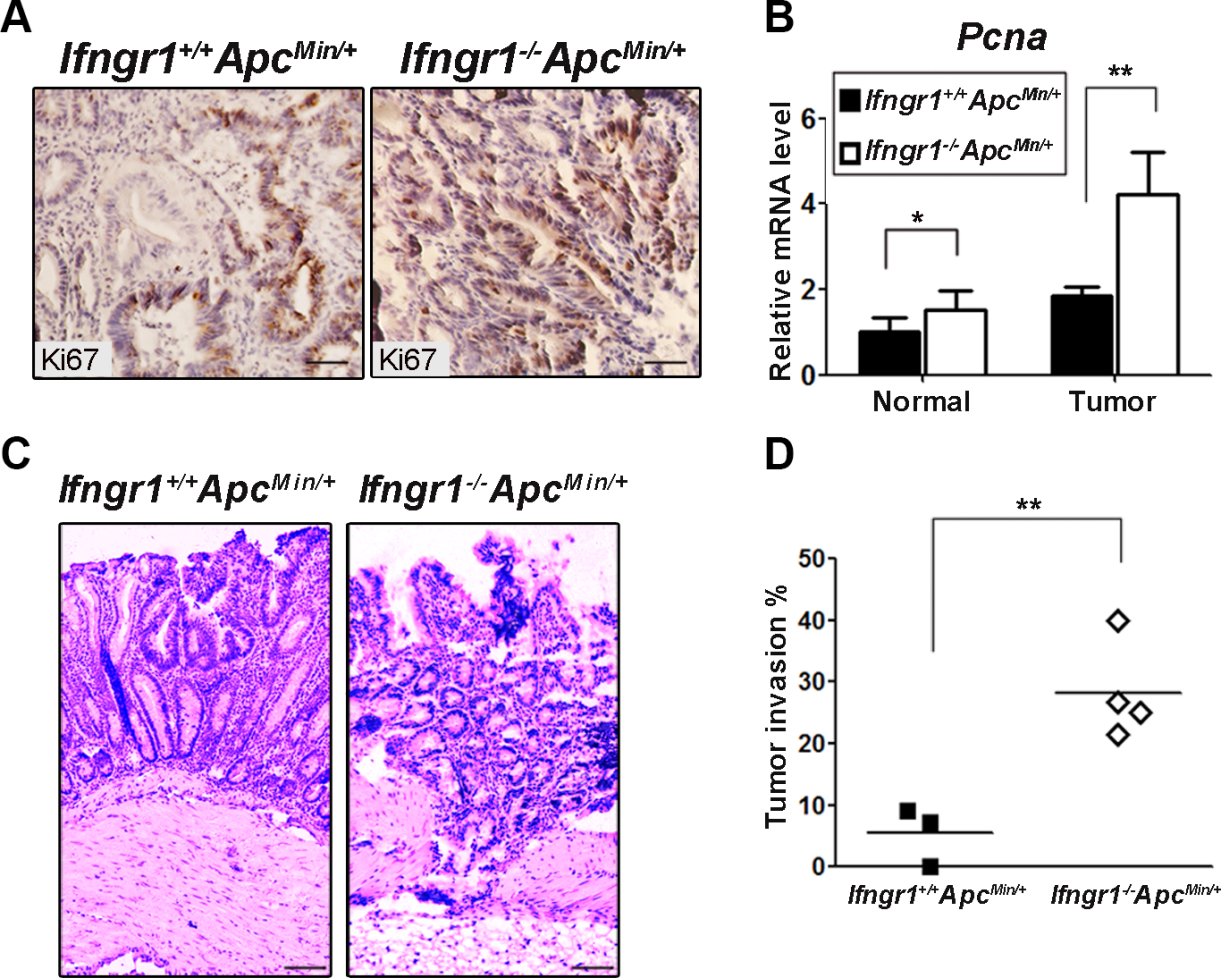
Increased malignancy in tumors of *Ifngr1*^−/−^*Apc^Min/^*^+^ mice (**A**) Immunohistochemistry for Ki67 revealed increased proliferation in tumors of *Ifngr1*^−/−^*Apc^Min/+^* mice (Bar = 50 μm). (**B**) RNA was extracted from size-matched intestinal normal tissues and tumors of *Ifngr1^+/+^Apc^Min/+^* mice and *Ifngr1*^−/−^*Apc^Min/+^* mice, and the levels of *Pcna* were evaluated by qRT–PCR (mean ± SD, *n* = 3 for each group; *P < 0.05, **P < 0.01, ***P < 0.005, Unpaired t test.). (**C**) Histological images of tumors in *Ifngr1^+/+^Apc^Min/+^* mice and *Ifngr1*^−/−^*Apc^Min^*/+ mice (Bar = 50 μm). (**D**) Histopathology of intestinal tumors in *Ifngr1^+/+^Apc^Min/+^* and *Ifngr1*^−/−^*Apc^Min/+^* mice. At least 10 randomly picked tumors were examined per mouse (*P < 0.05, **P < 0.01, ***P < 0.005, Chi-square test.).

We next characterized proliferation and apoptosis of the intestinal tumors. Ki67 immunostaining indicated that there were more proliferating cells in tumors of *Ifngr1*^−/−^*Apc^Min/+^* mice than in those of *Ifngr1^+/+^Apc^Min/+^* mice (Figure 2A). Expression level of *Pcna*, another marker of proliferation, showed the same trend (Figure 2B). In contrast, the level of apoptosis, as assessed by TUNEL-staining, was not significantly different between *Ifngr1*^−/−^*Apc^Min/+^* and *Ifngr1^+/+^Apc^Min/+^* mice (Supplementary Figure S1).

In a C57BL/6J background, most tumors in the *Apc^Min/+^* mice are benign adenomas and do not exhibit invasion or metastasis [22]. We performed histological examination of the polyps in *Ifngr1*^−/−^*Apc^Min/+^* mice and observed that > 30% of them were of invasive nature (Figure 2C and 2D). These results indicate that the IFN-γ receptor deficiency renders increased invasiveness to the intestinal tumors.

### Characterization of global gene expression changes in intestinal tumors of *Apc^Min/+^* mice

**Figure 3:**
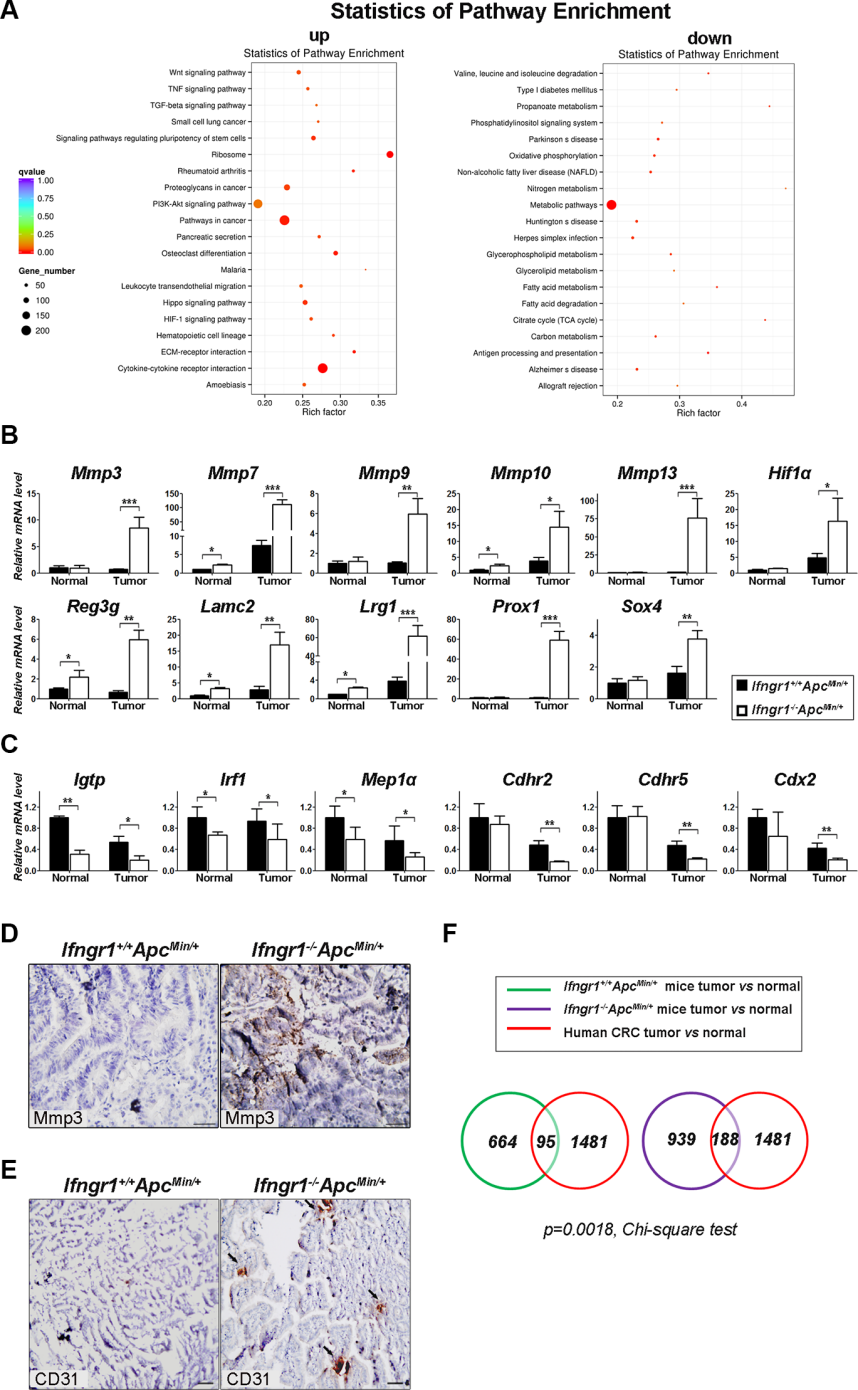
Molecular characterization of intestinal tumors in Ifngr1^−/−^ApcMin/+ mice (**A**) KEGG enrichment results for differentially expressed genes detected by RNA-seq in the tumors of *Ifngr1*^−/−^*Apc^Min/+^* and *Ifngr1^+/+^Apc^min/+^* mice. There were three biological repeats for each group. The complete lists of differentially expressed genes with *p* value < 0.05 were shown in Supplementary Tables S2 and S3. (**B**) Validation of representative genes that were shown to be upregulated by RNA-seq. RNA was extracted from intestinal normal tissues and tumors of different groups of mice, and mRNA expression was evaluated by qRT–PCR (mean ± SD, *n* = 3 for each group; *p < 0.05, **P < 0.01, ***P < 0.005, Unpaired *t* test.) (**C**) Validation of representative genes that were shown to be downregulated by RNA-seq. Analysis was performed as in (B) (mean ± SD, *n* = 3 each; *p < 0.05, **P < 0.01, ***P < 0.005, Unpaired *t* test.) (**D**) Immunohistochemistry for Mmp3 in tumors of *Apc^Min/+^* mice and *Ifngr1*^−/−^*Apc^Min/+^* mice (Bar = 50 μm). (**E**) Immunohistochemistry for CD31 in tumors of *Apc^Min/+^* mice and *Ifngr1*^−/−^*Apc^Min/+^* mice (Arrows indicate CD31 positive cells, Bar = 50 μm) (**F**) Comparison of tumor-specific genes in *Apc^Min/+^* mice and in human CRC patients. RNA was extracted from intestinal normal tissues and tumors of *Ifngr1^+/+^Apc^min/+^* or *Ifngr1*^−/−^*Apc^min/+^* mice and was subjected to RNA-seq. The upregulated genes in tumors when compared to normal tissues were shown in Supplementary Tables S4 and S5. The upregulated genes in human CRC specimens when compared to normal mucosa were from GSE20842.

In order to gain insight into the molecular basis underlying the enhanced tumorigenesis in *Ifngr1*^−/−^*Apc^Min/+^* mice, we interrogated the global gene expression in tumors from *Ifngr1^+/+^Apc^Min/+^* and *Ifngr1*^−/−^*Apc^Min/+^* mice by RNA sequencing. 4021 genes were differentially expressed (adjusted *p* value < 0.05) between the two types of tumors (Supplementary Tables S2 and S3). KEGG analysis revealed a significant enrichment of multiple pathways that are known to enhance tumor progression (Figure 3A). Inflammatory response (TNF signaling pathway, cytokine-cytokine receptor interaction, leukocyte transendothelial migration), cell proliferation and growth (Wnt signaling pathway, ribosome genesis, PI3K-Akt signaling pathway, Hippo signaling pathway) and tissue remodeling (TGF β signaling pathway, production of proteoglycans, HIF-1 signaling pathway, ECM-receptor interaction) appeared to be upregulated in tumors of *Ifngr1*^−/−^*Apc^Min/+^* mice when compared to *Ifngr1^+/+^Apc^Min/+^* mice. Citric acid cycle and oxidative phosphorylation, processes that generate ATP, were downregulated in tumors of *Ifngr1*^−/−^*Apc^Min/+^* mice. Thus, the gene expression patterns in tumors of *Ifngr1*^−/−^*Apc^Min/+^* mice generally indicate an increased degree of malignancy when compared to those in *Ifngr1^+/+^Apc^Min/+^* mice.

We next performed qRT-PCR to validate the gene expression results obtained with RNA-seq. As shown in Figure 3B, representative genes, such as *Mmp3*, *Mmp7*, *Mmp9* and *Hif1α*, were all confirmed to be more pronouncedly upregulated in tumors of *Ifngr1*^−/−^*Apc^Min/+^* mice than in those of *Ifngr1^+/+^Apc^Min/+^* mice. The expression levels of *Cdhr2* and *Cdx2*, which act as tumor suppressors, were more pronouncedly downregulated in tumors from *Ifngr1*^−/−^*Apc^Min/+^* mice (Figure 3C). Immunostaining further confirmed the high level expression of Mmp3 in tumors of *Ifngr1*^−/−^*Apc^Min/+^* mice, in contrast to its absence in tumors of *Apc^Min/+^* mice (Figure 3D).

It was reported that IFN-γ receptor expression by non-hematopoietic cells is required for tumor immunity and inhibition of angiogenesis [23]. We therefore performed immunohistochemical analysis of CD31, a marker of endothelial cells and angiogenesis, in intestinal tumors. CD31-positive cells were indeed more abundant in tumors of *Ifngr1*^−/−^*Apc^Min/+^* mice than in those of *Ifngr1^+/+^Apc^Min/+^* mice (Figure 3E), suggesting that the tumors of *Ifngr1*^−/−^*Apc^Min/+^* mice were more vascularized.

We also surveyed the genes that are differentially expressed between tumors and mucosal tissues by RNA-seq. While 664 genes were upregulated at least 2-fold in tumors when compared to normal mucosa in *Ifngr1^+/+^Apc^Min/+^* mice (Supplementary Table S4), more genes, 939, were upregulated in tumors of *Ifngr1*^−/−^*Apc^Min/+^* mice (Supplementary Table S5). The upregulated genes were compared to those previously reported to be upregulated in human colorectal cancer specimens when compared to mucosa (GSE20842) [24]. Interestingly, the upregulated genes in tumors of *Ifngr1*^−/−^*Apc^Min/+^* mice were found to be more likely to overlap with those upregulated in human colorectal cancers (Figure 3F, *p*-value = 0.0018). This result further supports that tumors of *Ifngr1*^−/−^*Apc^Min/+^* mice more resemble human colorectal cancers than those in *Ifngr1^+/+^Apc^Min/+^* mice.

### Intestinal tumors of *Ifngr1^−/−^Apc^Min/+^* mice exhibit increased inflammation

**Figure 4:**
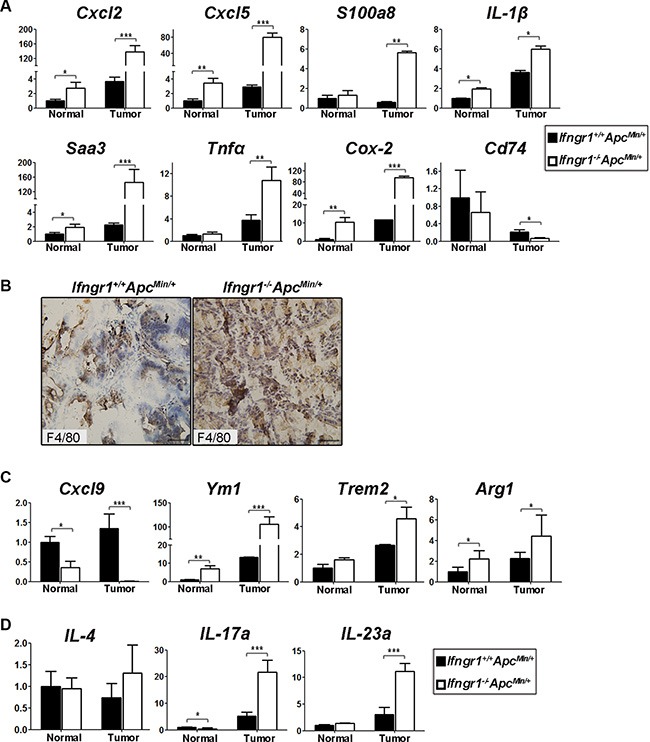
Characterization of cytokine milieu in tumors of *Ifngr1*^+/+^*Apc^Min/^*^+^ and *Ifngr1*^−/−^*Apc^Min/^*^+^ mice (**A**) RNA was extracted from intestinal normal tissues and tumors of *Ifngr1^+/+^Apc^Min/+^* mice and *Ifngr1*^−/−^*Apc^Min/+^* mice, and expression levels of the indicated genes were evaluated by qRT–PCR (mean ± SD, *n* = 3 for each group; *P < 0.05, **P < 0.01, ***P < 0.005, Unpaired *t* test.). (**B**) Immunohistochemistry for F4/80. Macrophages were more abundant in tumors of *Ifngr1*^−/−^*Apc^Min/+^* mice than in those of *Ifngr1^+/+^Apc^Min/+^* mice (Bar = 50 μm). (**C**) Transcript levels of typical M1 and M2 markers. (**D**) Transcript levels of typical Th2 and Th17 markers. (mean ± SD, *n* = 3 for each group; *P < 0.05, **P < 0.01, ***P < 0.005, Unpaired *t* test.).

Because IFN-γ is a pro-inflammatory cytokine, lack of IFN-γ receptor would be expected to bring about changes in the repertoire of cytokines. Gene expression profiling of tumors from *Apc^Min/+^* and *Ifngr1*^−/−^*Apc^Min/+^* mice indeed showed a significant enrichment of genes involved in inflammatory response, including *Cxcl2*, *Cxcl5, Reg3b, IL-1β, Saa3, Tnfα*, and *Cox-2(Ptgs2)* in tumors of *Ifngr1*^−/−^*Apc^Min/+^* mice. We confirmed the upregulation of those genes in tumors of *Ifngr1*^−/−^*Apc^Min/+^* mice by qRT-PCR (Figure 4A). Interestingly, many pro-inflammatory factors, such as *Cxcl2*, *Cxcl5* and *Cox-2*, were upregulated even in non-tumor tissues in the absence of IFN-γ receptor, suggesting that in addition to being favorable for tumor progression, the microenvironment in the intestines of *Ifngr1*^−/−^mice may also be conducive to tumor initiation.

We next examined tumor-associated macrophages (TAMs) in intestinal polyps by immunohistochemical staining of F4/80, a marker for macrophages, and found that macrophages were more abundantly infiltrated in tumors of *Ifngr1*^−/−^*Apc^Min/+^* mice than in those of *Ifngr1^+/+^Apc^Min/+^* mice (Figure 4B).

IFN-γ was reported to promote the phenotypic conversion of TAMs from M2 to M1 in polyps of *Apc^Min/+^* mice [15]. Expression levels of *Cxcl9* and *Ccl5*, which act downstream of IFN-γ-Stat1 signaling pathway and function as key effector molecules produced by M1 macrophages, were indeed significantly lower in tumors of *Ifngr1*^−/−^*Apc^Min/+^* mice, as revealed RNA-seq (Supplementary Table S3). In contrast, expression levels of *Arginase1*, *Trem2*, and *Chil3* (*Ym1)*, which are typical M2 markers, were significantly higher in tumors of *Ifngr1*^−/−^*Apc^Min/+^* mice than in those of *Ifngr1^+/+^Apc^Min/+^* mice. However, the expression of *IL-4*, which induces M2 polarization, was not changed (Supplementary Table S2). The expression levels of representative M1- and M2-associated genes were confirmed by qRT–PCR. (Figure 4C). Thus, the impaired IFN-γ signaling due to lack of IFN-γ receptor probably generated a cytokine milieu that favors TAMs toward M-2 polarization.

We next examined CD4+ cells in the tumors of *Apc^Min/+^* mice by immunofluorescence staining, but did not detect a significant difference in abundance between *Ifngr*^−/−^*Apc^Min/+^* mice and *Ifngr1^+/+^Apc^Min/+^* mice (Supplementary Figure S2). The function of Th1 cells is presumably impaired due to disruption of IFN-γ signaling in *Ifngr*^−/−^ mice. The expression level of IL-4, which induces Th2 differentiation and also functions as an effector of Th2 cells, was not significantly increased *Ifngr*^−/−^*Apc^Min/+^* mice (Figure 4D). However, the expression levels of *IL-23a* and *IL-17a*, which induces Th17 differentiation and functions as Th17 effector, respectively, were significantly increased in tumors of *Ifngr*^−/−^*Apc^Min/+^* mice (Figure 4D). The upregulation of IL-23a/IL-17a axis further substantiates the notion of increased inflammation in in tumors of *Ifngr*^−/−^*Apc^Min/+^* mice.

Mutation or loss of the *Apc* gene is thought to represent the initiating event in the process of intestinal tumorigenesis. It is the functional loss of *Apc* that leads to the nuclear accumulation of β-catenin. In C57BL/6 *Apc^Min/+^* mice, all the tumors are developed as a result of loss of the wide-type allele of *Apc* gene, mainly via mitotic recombination [25]. To determine whether wild-type *Apc* allele is also lost in tumors from *Ifngr1*^−/−^*Apc^Min/+^* mice, *Apc* loss of heterozygosity (LOH) analysis was performed in 10 randomly selected tumors from *Ifngr1*^−/−^*Apc^Min/+^* and *Ifngr1*^−/−^*Apc^Min/+^* mice, respectively. We observed that only the *Apc^Min^* allele, but not the wild-type allele, was detected in tumors arising from *Ifngr1*^−/−^*Apc^Min/+^* mice, as in those from *Apc^Min/+^* mice (Supplementary Figure S3), suggesting that lack of IFN-γ receptor led to increased tumor multiplicity similarly via loss of the wild-type *Apc* allele.

### IFNGR1 was downregulated in human colorectal cancers

**Figure 5:**
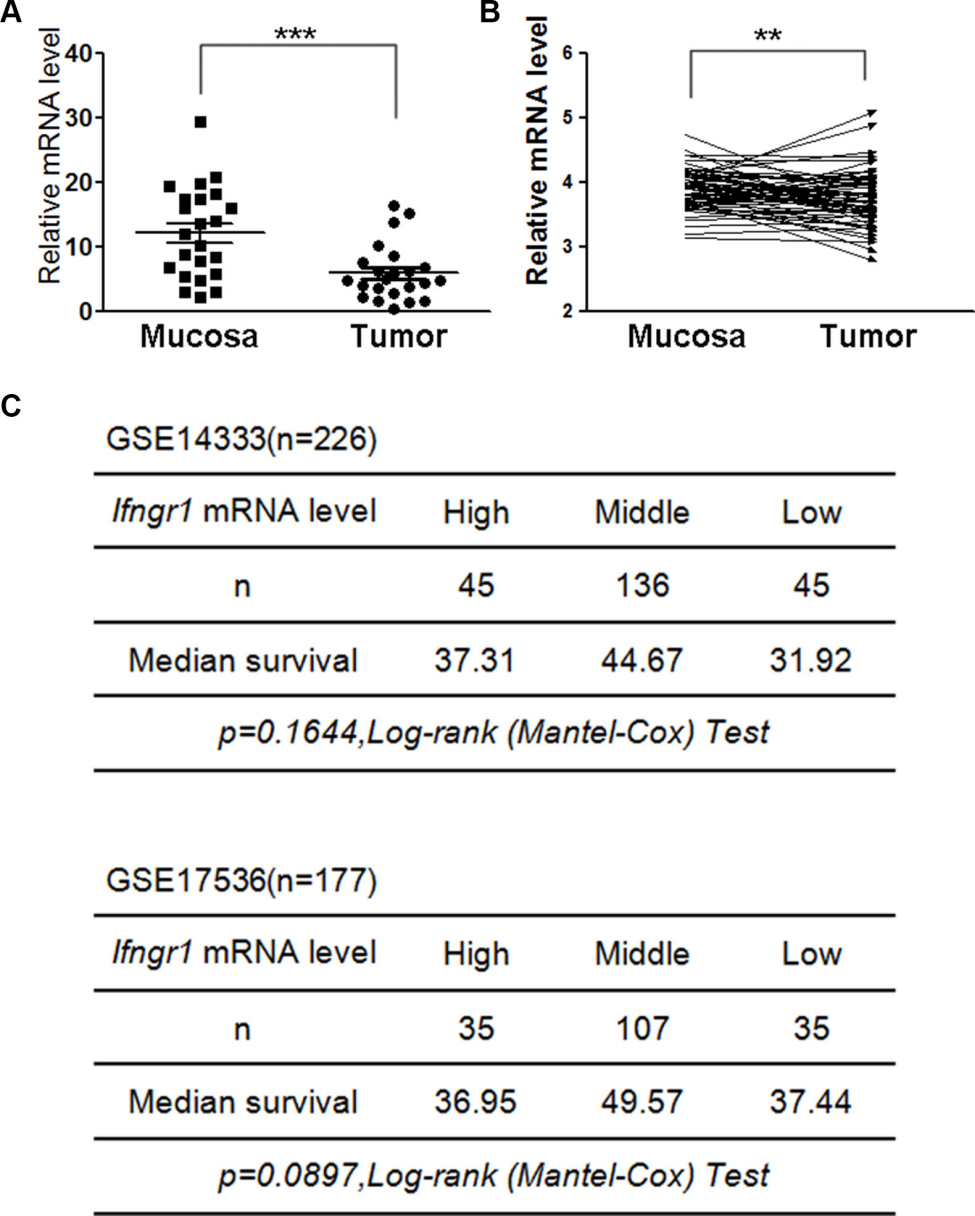
Expression levels of *IFNGR1* in colon cancers (**A**) 30 pairs of intestinal mucosal tissues and tumors were excised from *Apc^min/+^* mice, and expression levels of *Ifngr1* were evaluated by qRT–PCR (*P < 0.05, **P < 0.01, ***P < 0.005, Unpaired *t* test). (**B**) *IFNGR1* mRNA levels in normal and cancer tissues of colorectal cancers patients were compared. Data were from GSE20842 (*P < 0.05, **P < 0.01, ***P < 0.005, Paired *t* test). (**C**) *IFNGR1* expression levels and prognosis in human colorectal cancer. Survival information and expression levels of *Ifngr1* were obtained from the public available databases (GSE14333 and GSE17536).

Since tumorigenesis in *Apc^min/+^* mice is enhanced in the absence of IFN-γ receptor, we speculated that IFN-γ receptor may function to impede intestinal tumorigenesis and that the intestinal tumors may exhibit a reduced expression of IFN-γ receptor. We first determined the transcript levels of *Ifngr1* in adenomas and adjacent normal tissues in *Apc^Min/+^* mice by qRT-PCR. Indeed, *Ifngr1* expression was significantly downregulated in tumors when compared to that in mucosa tissues (Figure 5A). We next compared the expression levels of *IFNGR1* in 65 pairs of human colorectal cancers and normal mucosa tissues (GSE20842) [24]). Consistently, *IFNGR1* was significantly downregulated in cancer specimens when compared to mucosa tissues (Figure 5B). It is noteworthy that the single nucleotide polymorphismrs3799488 in *IFNGR1* was reported to be associated with increased risk of rectal cancers [26].

Since *IFNGR1* expression was downregulated in human colorectal cancers, we next interrogated whether the expression levels of *IFNGR1* were correlated with prognosis of cancer patients using the public available databases GSE14333 and GSE17536. Although no statistically significant correlations between *IFNGR1* expression levels and survival were detected, the results suggest a trend that the group with the intermediate levels of *IFNGR1* had the longest median survival time (Figure 5C).

## DISCUSSION

We showed here that lack of IFN-γ receptor resulted in enhanced tumorigenesisin *Apc^Min/+^* mice. Gene expression profiling of the intestinal tumors by RNA-seq revealed a number of molecular features that are consistent with increased tumor progression and malignancy in *Ifngr1*^−/−^*Apc^Min/+^* mice. In particular, pathways involved in inflammatory response, cell proliferation and tissue remodeling were upregulated with the absence of IFN-γ receptor. Meanwhile, citric acid cycle and oxidative phosphorylation were downregulated, presumably to favor biosynthesis that is in high demand for tumor growth. As expected, cytokine milieu in the intestinal tumors of in *Ifngr1*^−/−^*Apc^Min/+^* mice favored the M2-polarization of TAMs. Consistent with its inhibitory effect on intestinal tumorigenesis, the expression of IFN-γ receptor is commonly downregulated in mouse intestinal adenomas and human colorectal cancers.

Increased tumorigenesis was also reported in *IFN-γ^+/−^ Apc^Min/+^* mice [27]. Therefore, it appears that deficiency in either the IFN-γ receptor or its ligand would lead to increased intestinal tumorigenesis. While it is clear that IFN-γ signaling functions to impede tumor progression in *Apc^Min/+^* mice, whether the IFN-γ signaling exerts its antitumor effect simply by acting on tumor cells or by modulating tumor microenvironment needs to be clarified. If IFN-γ can inhibit EGFR/Erk1/2 and Wnt/β-catenin signaling pathways, as was reported [27], lack of IFN-γ receptor would be expected to lead to an upregulation of those pathways in tumor cells, consequently driving the proliferation of tumor cells. It can then be argued that the increased inflammatory response in the tumors of *Ifngr1*^−/−^*Apc^Min/+^* mice could be the consequence of accelerated tumorigenesis due to lack of IFN-γ receptor in the tumor cells per se. Our data presented in this study, as well as evidence in some previous studies, implicate IFN-γ signaling as an important player in the regulation of tumor microenvironment. An earlier report showed that expression of IFN-γ receptor on non-hematopoietic host cells, but not that on tumor cells, is essential for antitumor immunity of the host in the effector phase [23]. The facts that IFN-γ-Stat1 signaling is critical for driving M1-polarization of TAMs, and that some pro-inflammatory factors, such as *Ptgs2*, and M2-markers are differentially expressed even in normal tissues of *Ifngr1*^−/−^*Apc^Min/+^* mice when compared to *Ifngr1^+/+^Apc^Min/+^* mice, further support the idea that establishment of a more permissive microenvironment due to lack of IFN-γ receptor can also be responsible for the enhanced tumorigenesis in *Ifngr1*^−/−^*Apc^Min/+^* mice. The increased expression of MMPs in tumors of *Ifngr1*^−/−^*Apc^Min/+^* mice was consistent with the previously reported inhibition of MMPs by IFN-γ during inflammation [28]. However, it should be pointed out that while IFN-γ signaling is essential for tumor immunity, excessive activation of IFN-γ signaling pathway may not necessarily lead to better outcomes in patients of colorectal cancer. An examination of possible correlation between *IFNGR1* expression levels and survival in colorectal patients revealed no statistically significant association, though tumors with highest and lowest expression of *IFNGR1* tend to render patients a shorter survival. A genetic polymorphism in *IFNGR1* was reported to be associated with increased risk of rectal cancers [26], however, whether or not this polymorphism reduces IFN-γ receptor function remains to be determined.

The *Ifngr1*^−/−^*Apc^Min/+^* mice showed a significantly shorter lifespan than the *Ifngr1^+/+^Apc^Min/+^* mice. While the intestinal tumorigenesis is significantly enhanced in *Ifngr1*^−/−^*Apc^Min/+^* mice when compared to that in *Ifngr1^+/+^Apc^Min/+^* mice, enhanced tumorigenesis is not necessarily the major cause of their shorter survival. *Apc^Min/+^* mice are also impaired in hematopoiesis due to aberrant activation of Wnt signaling pathway [29–31]. IFN-γ, on the other hand, also regulates hematopoiesis [32–34]. It is possible that aberrant hematopoiesis may also contribute to early lethality of *Ifngr1*^−/−^*Apc^Min/+^* mice. Indeed, *Ifngr1*^−/−^*Apc^Min/+^* mice were found to exhibit more severe anemia. How hematopoiesis is affected in *Ifngr1*^−/−^*Apc^Min/+^* mice needs to be further studied.

In summary, we showed that lack of IFN-γ receptor led to enhanced inflammatory response, upregulated tissue remodeling, augmented IL-23/IL-17 axis and M2-polarization of TAMs in tumors of *Apc^Min/+^* mice. Those alterations could function to promote intestinal tumorigenesis. Our results provided further insight into pathogenesis of colorectal cancer and should bear implications in the clinical application of IFN-γ in the management of colon cancer.

## MATERIALS AND METHODS

### Mice

The *Apc^Min/^*^+^ mice and *Ifngr1*^−/−^ mice in C57BL/6 background were purchased from Jackson Laboratory (Bar Harbor, ME). All the animals were bred and maintained in a specific pathogen-free animal facility. DNA was isolated from tail biopsies as described previously. Genotyping of *Ifngr1*^−/−^mice was conducted following a protocol provided by Jackson Laboratory. The genotypes of the *Apc* alleles were determined by PCR using the following primers: ApcMin1: 5′-GCCATCCCTTCACGTTAG-3′; ApcMin2: 5′-TTCCACTTTGGCATAAGG-3′; ApcMin3: 5′- TTCTGAGAAAGACAGAAGTTA-3′. Reactions were carried out using a Takara PCR kit(Dalian). Reactions were carried out in cycling condition of 94°C for 3 min, followed by 35 cycles of 94°C for 30 s, 55°C for 1 min, and 72°C for 1 min, followed by 72°C for 5 min. The use and care of the animals were reviewed and approved by the Institutional Animal Care and Use committee at Shandong University School of Medicine.

### Tumor scoring and tissue preparation

All mice were sacrificed around 18 weeks old. The entire intestinal tract was removed, cut open along its longitudinal axis, and flushed clean with saline. Tumors were counted and measured for the entire length of intestine using a dissecting microscope with a micrometer. All tumors were scored by a single examiner who was blind to the genotype of the subject. Tissues were divided for storage in −80°C and for immediate processing. For histology study, the intestines were fixed flat on slides in 10% buffered formalin for 24 h at room temperature and embedded in paraffin by en face preparation.

### Immunohistochemistry

After deparaffinization and rehydration, the sections were boiled in citrate sodium buffer for 15 minutes for antigen recovery, and immersed in 3% H_2_O_2_ for 10 minutes to quench endogenous peroxidase. Sections were then blocked with 10% serum at 37°C for 1 hour. The primary antibodies (Ki67 Abcam, 1:200 dilution; Mmp3, Abcam, 1:200 dilution; CD31, Abcam, 1:200; F4/80, Proteintech, 1:200 dilution) were added to the sections and incubated overnight at 4°C. After washing, the sections were coated with a horseradish peroxidase (HRP) conjugated second antibody (Jackson ImmunoResearch; 1:200 dilution) and then incubated at 37°C for 1 hour. DAB was used to visualize immunoreactions [35].

### RNA sequencing

Tumor samples in each group were pooled and were subjected to RNA extraction. Sequencing libraries were generated using NEBNext^®^ Ultra^™^ RNA Library Prep Kit for Illumina^®^ (NEB, USA) following manufacturer's recommendations. Briefly, mRNA was purified from total RNA using poly-T oligo-attached magnetic beads. Fragmentation was carried out using divalent cations under elevated temperature in NEBNext First Strand Synthesis Reaction Buffer. First strand cDNA was synthesized using random hexamer primer and M-MuLV Reverse Transcriptase. Second strand cDNA synthesis was subsequently performed using DNA Polymerase I and RNase H. Remaining overhangs were converted into blunt ends via exonuclease/polymerase activities. PCR was performed with Phusion High-Fidelity DNA polymerase, Universal PCR primers and Index (X) Primer. PCR products were purified (AMPure XP system) and library quality was assessed on the Agilent Bioanalyzer 2100 system. The clustering of the index-coded samples was performed on a cBot Cluster Generation System using TruSeq PE Cluster Kit v3-cBot-HS (Illumina) according to the manufacturer's instructions. After cluster generation, the library preparations were sequenced on an Illumina Hiseq 2000/2500 platform and 100 bp/50 bp single-end reads were generated.

### Quantitative RT-PCR

Total RNA was extracted from frozen tissues or cultured cells using TRIzol reagent (Invitrogen), and was reverse transcribed using reverse transcriptase (TOYOBO). Quantitative real-time PCR analysis was performed on a Roche LightCycler^®^ 480 System using SYBR GREEN mix (TOYOBO). Primers used for quantitative RT-PCR were listed in the supplementary Table S1.

### LOH analysis of Apc

Tumor or normal tissue was excised carefully and DNA extracted. Loss of heterozygosity (LOH) at Apc locus was determined by PCR, as described for genotyping of the mouse tails.

### Statistical methods

Data analysis was performed using the *Mantel-Cox* test or 2-tailed student's *t*-test (SPSS 11.5 software, SPSS Inc., Chicago, IL). All data were expressed as the mean ± SD, and significant outcomes were shown as follows: *P < 0.05, **P < 0.01, and ***P < 0.005.

## SUPPLEMENTARY FIGURES AND TABLES


