# Supplementary material for: Lack of interferon-γ receptor results in a microenvironment favorable for intestinal tumorigenesis
Source: Oncotarget. 2016 Jun 7;7(27):42099–109. doi: 10.18632/oncotarget.9867 (PMC5173119; doi:10.18632/oncotarget.9867)
Supplement: Supplementary file 1 [file oncotarget-07-42099-s001.pdf]

Lack of interferon-γ receptor results in a microenvironment favorable for intestinal tumorigenesis

Supplementary Materials

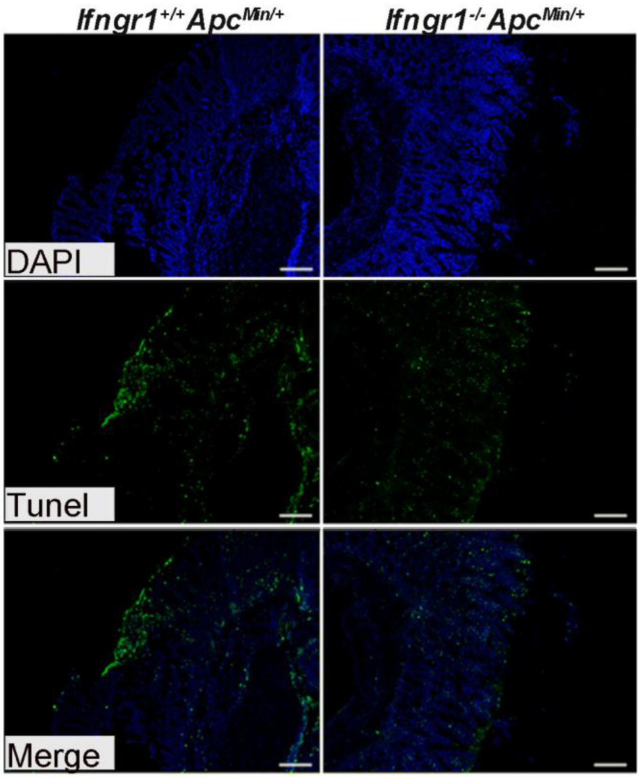

Supplementary Figure S1: The levels of apoptosis in tumors were not significantly different between *Ifngr1*$^{-/-}$ *Apc*$^{Min/+}$ and *Ifngr1*$^{+/+}$*Apc*$^{Min/+}$ mice. The apoptotic index assessed by Tunel-staining (Bar = 200 μm).

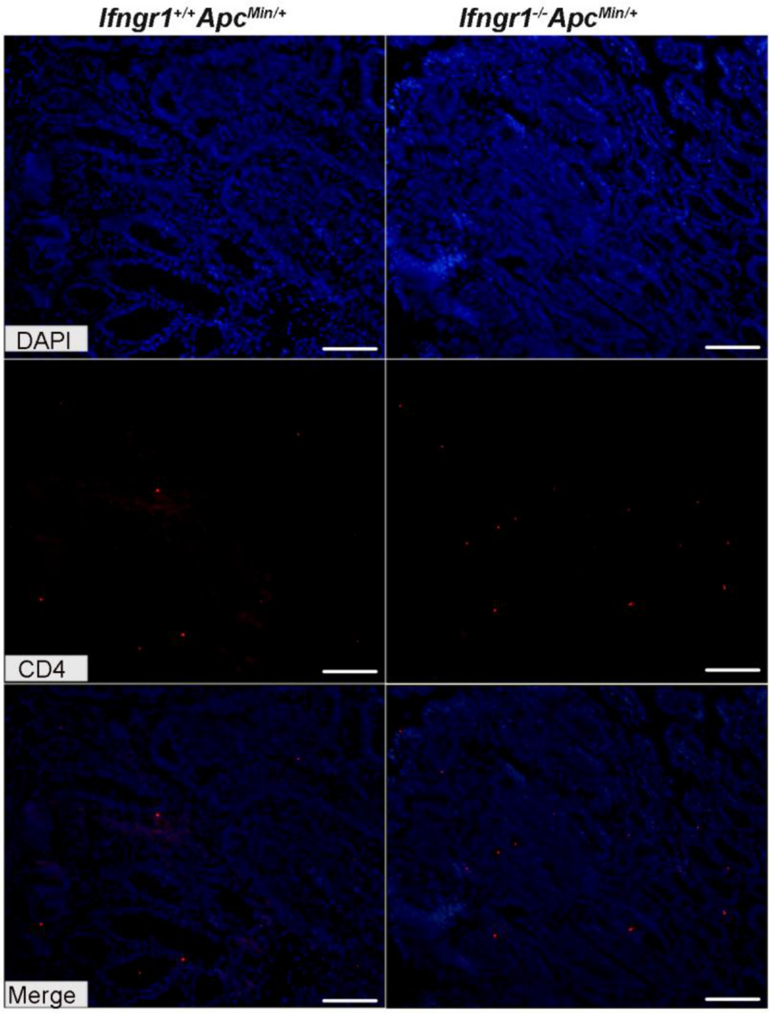

Supplementary Figure S2: CD4+ T cells infiltrating tumors in *Ifngr1*$^{+/+}$*Apc*$^{Min/+}$ and *Ifngr1*$^{-/-}$*Apc*$^{Min/+}$ mice. Tumor sections were stained with CD4 mAb (Bar = 100 µm).

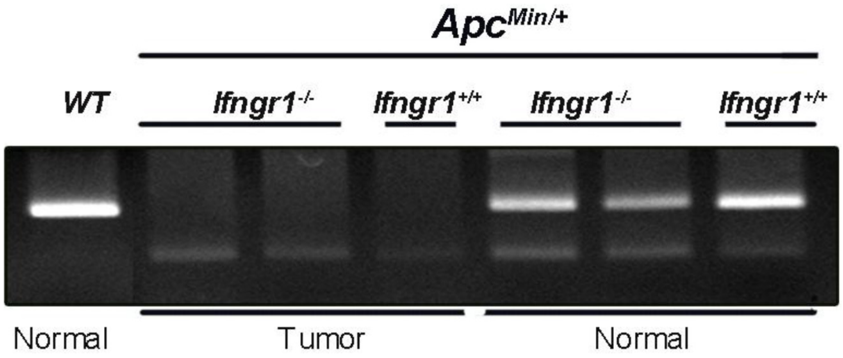

Supplementary Figure S3: LOH analysis of Apc gene. PCR bands representing the wild type allele (600 bp) and mutant allele (*Apc*^{Min}) alleles (340 bp) were shown.

Supplementary Table S1: Primers used for qRT-PCR

Name	Primer sequence (5′→3′)
mGapdh-F	AGGTCGGTGTGAACGGATTTG
mGapdh-R	TGTAGACCATGTAGTTGAGGTCA
mPcna-F	TTTGAGGCACGCCTGATCC
mPcna-R	GGAGACGTGAGACGAGTCCAT
mMmp3-F	GGCCTGGAACAGTCTTGGC
mMmp3-R	TGTCCATCGTTCATCATCGTCA
mMmp7-F	CTTACCTCGGATCGTAGTGGA
mMmp7-R	CCCCAACTAACCCTCTTGAAGT
mMmp9-F	GAGCCACTAGCCATCCTGG
mMmp9-R	CTGAGCAAGATCCATGCTTGG
mMmp10-F	GAGCCACTAGCCATCCTGG
mMmp10-R	CTGAGCAAGATCCATGCTTGG
mMmp13-F	TGTTTGCAGAGCACTACTTGAA
mMmp13-R	CAGTCACCTCTAAGCCAAAGAAA
mHif1a-F	GTCCCAGCTACGAAGTTACAGC
mHif1a-R	CAGTGCAGGATACACAAGGTTT
mReg3g-F	ATGCTTCCCCGTATAACCATCA
mReg3g-R	ACTTCACCTTGCACCTGAGAA
mLamc2-F	TTGCCTCAACTGCAATGACAA
mLamc2-R	TCTCGATGTCGGTAAAACCCC
mLrg1-F	TTGGCAGCATCAAGGAAGC
mLrg1-R	CAGATGGACAGTGTCGGCA
mProx1-F	AGAAGGGTTGACATTGGAGTGA
mProx1-R	TGCGTGTTGCACCACAGAATA
mSox4-F	GACAGCGACAAGATTCCGTTC
mSox4-R	GTTGCCCGACTTCACCTTC
mIgtp-F	CTCATCAGCCCGTGGTCTAAA
mIgtp-R	TCACCGCCTTACCAATATCTTCA
hIrf1-F	ATGCCCATCACTCGGATGC
hIrf1-R	CCCTGCTTTGTATCGGCCTG
mMep1a-F	AGTCTTGTGTGGATTTCAAGCC
mMep1a-R	GTCACATCCCTCACCAATGGA
mCdhr2-F	GGCCTCGATTCTACAGCCAAT
mCdhr2-R	GCTCAGAGCCATTCTCAGTCAC
mCdx2-F	TACCCGGACTACGGTGGTTAC
mCdx2-R	GTGATGGTGCGCGTGGTAT
mCxclL2-F	CCAACCACCAGGCTACAGG
mCxcl2-R	GCGTCACACTCAAGCTCTG
mCxcl5-F	GTTCCATCTCGCCATTCATGC
mCxcl5-R	GCGGCTATGACTGAGGAAGG
m-S100a8-F	AAATCACCATGCCCTCTACAAG
m-S100a8-R	CCCACTTTTATCACCATCGCAA
mIL-1b-F	GAAATGCCACCTTTTGACAGTG
mIL-1b-R	TGGATGCTCTCATCAGGACAG
mSaa3-F	AGAGAGGCTGTTCAGAAGTTCA

mSaa3-R	AGCAGGTCGGAAGTGGTTG
mTnfα-F	CAGGCGGTGCCTATGTCTC
mTnfα-R	CGATCACCCCGAAGTTCAGTAG
mCox-2-F	ACGGTCCTGAACGCATTTATG
mCox-2-R	TTGGCCCCATTTAGCAATCTG
mCd74-F	AGATGCGGATGGCTACTCC
mCd74-R	TCATGTTGCCGTACTTGGTAAC
mYm1-F	TTATCCTGAGTGACCCTTCTAAG
mYm1-R	TCATTACCCTGATAGGCATAGG
mTrem2-F	CTGGAACCGTCACCATCACTC
mTrem2-R	CGAAACTCGATGACTCCTCGG
mArg1-F	TGGCTTGCGAGACGTAGAC
mArg1-R	GCTCAGGTGAATCGGCCTTTT
mCxclL9-F	GGAGTTCGAGGAACCCTAGTG
mCxcl9-R	GGGATTTGTAGTGGATCGTGC
mIL-4-F	ACCTTGACGGTGTTCATACAGT
mIL-4-R	CTGCTCCTATTCGACCACTATCT
mIL-17a-F	TCAGCGTGTCCAAACACTGAG
mIL-17a-R	CGCCAAGGGAGTTAAAGACTT
mIL-23a-F	AATAATGTGCCCCGTATCCAGT
mIL-23a-R	GCTCCCCTTTGAAGATGTCAG

Supplementary Table S2: List of upregulated genes in tumors (GR_T) of *Ifngr1*$^{-/-}$ mice

Gene_id	read count_ GR_T	read count_ WT_T	log2 FoldChange	pval	padj	Gene_Name
ENSMUSG00000000125	12.08404	1.756199	2.7826	0.000666	0.00478	Wnt3
ENSMUSG00000000142	1140.65	435.45	1.3893	3.39E-06	4.77E-05	Axin2
ENSMUSG00000000202	19.86824	3.709066	2.4213	0.001692	0.010491	Btbd17
ENSMUSG00000000204	917.9862	731.205	0.3282	0.002155	0.012814	Slfn4
ENSMUSG00000000253	227.4493	162.461	0.48545	0.002289	0.013486	Gmpr
ENSMUSG00000000290	258.8408	188.0293	0.46111	0.001205	0.007829	Itgb2
ENSMUSG00000000295	225.0227	132.1548	0.76784	2.18E-06	3.21E-05	Hddc2
ENSMUSG00000000320	412.5599	75.65087	2.4472	6.85E-05	0.000668	Alox12
ENSMUSG00000000420	1836.905	1463.067	0.32828	0.000561	0.004145	Galnt1
ENSMUSG00000000486	121.9231	71.26328	0.77474	0.000182	0.001576	1-Sep
ENSMUSG00000000489	153.2227	80.47632	0.92899	2.00E-06	2.99E-05	Pdgfb
ENSMUSG00000000531	73.45668	32.12588	1.1932	2.15E-05	0.000241	Grasp
ENSMUSG00000000555	630.0765	485.7188	0.37541	0.009325	0.043014	Itga5
ENSMUSG00000000568	1676.511	1368.013	0.29338	0.002007	0.012111	Hnrnpd
ENSMUSG00000000581	540.7576	423.4579	0.35276	0.00406	0.02156	C1d
ENSMUSG00000000628	4169.851	2324.437	0.84311	6.95E-20	7.21E-18	Hk2
ENSMUSG00000000740	5879.005	4526.318	0.37723	1.50E-05	0.000176	Rpl13
ENSMUSG00000000782	227.7753	79.47458	1.519	1.96E-10	6.58E-09	Tcf7
ENSMUSG00000000787	6968.145	4486.729	0.63511	2.66E-13	1.35E-11	Ddx3x
ENSMUSG00000000811	139.0185	78.01577	0.83344	1.44E-05	0.000169	Txnrd3
ENSMUSG00000000826	1951.391	1502.013	0.37761	9.52E-05	0.000894	Dnajc5
ENSMUSG00000000827	612.7673	458.4631	0.41853	0.000263	0.002153	Tpd52l2
ENSMUSG00000000861	142.7835	96.46568	0.56574	0.002861	0.016157	Bcl11a
ENSMUSG00000000901	51.72257	28.78441	0.84551	0.00551	0.027764	Mmp11

ENSMUSG00000000957	1707.093	854.3743	0.9986	2.59E-10	8.61E-09	Mmp14
ENSMUSG00000000982	16.42947	2.783295	2.5614	0.000201	0.001719	Ccl3
ENSMUSG00000000983	1596.473	204.596	2.964	5.30E-12	2.31E-10	Wfdc18
ENSMUSG00000001025	7165.734	5496.567	0.38258	6.36E-06	8.29E-05	S100a6
ENSMUSG00000001053	162.5343	91.43793	0.82988	1.20E-05	0.000146	N4bp3
ENSMUSG00000001056	718.0322	496.1987	0.53313	1.44E-06	2.22E-05	Nhp2
ENSMUSG00000001128	196.3774	107.542	0.86873	1.28E-06	2.00E-05	Cfp
ENSMUSG00000001131	110.0419	30.13426	1.8686	6.38E-06	8.30E-05	Timp1
ENSMUSG00000001227	212.7823	61.95616	1.7801	6.48E-22	7.92E-20	Sema6b
ENSMUSG00000001247	1723.509	1048.841	0.71655	5.25E-06	7.00E-05	Lsr
ENSMUSG00000001249	87.1979	48.08988	0.85856	0.000278	0.002251	Hpn
ENSMUSG00000001435	1034.808	649.162	0.67271	1.31E-10	4.55E-09	Col18a1
ENSMUSG00000001467	2603.023	2139.428	0.28296	0.002778	0.015783	Cyp51
ENSMUSG00000001473	369.3194	286.5185	0.36624	0.00419	0.022146	Tubb6
ENSMUSG00000001506	3102.35	2164.968	0.51902	8.04E-09	2.02E-07	Col1a1
ENSMUSG00000001510	15.54216	3.385638	2.1987	0.001094	0.007229	Dlx3
ENSMUSG00000001525	3711.285	2907.657	0.35206	0.007828	0.037293	Tubb5
ENSMUSG00000001555	123.9325	78.60506	0.65686	0.001349	0.008615	Fkbp10
ENSMUSG00000001569	443.5462	340.6394	0.38084	0.002659	0.015231	Nom1
ENSMUSG00000001627	528.5795	287.8333	0.87689	0.000125	0.001141	Ifrd1
ENSMUSG00000001670	509.4333	373.9191	0.44617	0.000567	0.004182	Tat
ENSMUSG00000001674	535.233	355.898	0.5887	6.50E-07	1.09E-05	Ddx18
ENSMUSG00000001707	240.284	151.9995	0.66067	1.85E-05	0.000212	Eef1e1
ENSMUSG00000001729	2199.112	1816.102	0.27608	0.003661	0.019861	Akt1
ENSMUSG00000001768	852.8037	707.9721	0.26852	0.006946	0.033843	Rin2
ENSMUSG00000001783	1622.719	1322.509	0.29514	0.002129	0.012694	Rtcb
ENSMUSG00000001785	361.2864	268.3582	0.42898	0.000937	0.006358	Pwp1
ENSMUSG00000001794	3184.933	2434.752	0.38749	1.89E-05	0.000216	Capns1
ENSMUSG00000001815	54.50885	11.54555	2.2392	2.95E-05	0.000319	Evx2
ENSMUSG00000001819	910.6775	89.18071	3.3521	1.65E-58	1.09E-55	Hoxd13
ENSMUSG00000001823	102.9487	2.650176	5.2797	4.33E-39	1.46E-36	Hoxd12
ENSMUSG00000001833	1478.5	1216.601	0.28128	0.005499	0.027725	7-Sep
ENSMUSG00000001864	84.26221	19.27368	2.1283	7.13E-07	1.19E-05	Aif1l
ENSMUSG00000001930	450.9903	246.9744	0.86873	3.05E-11	1.17E-09	Vwf
ENSMUSG00000001946	127.2664	74.98464	0.76318	0.00507	0.025941	Esam
ENSMUSG00000001995	279.0544	203.5699	0.45502	0.001504	0.009463	Sipa1l2
ENSMUSG00000002014	1743.13	1363.352	0.35452	0.000254	0.00209	Ssr4
ENSMUSG00000002020	98.97523	33.39378	1.5675	1.09E-09	3.25E-08	Ltbp2
ENSMUSG00000002058	324.0822	231.2067	0.48718	0.000686	0.004887	Unc119
ENSMUSG00000002257	70.60537	42.29487	0.7393	0.00302	0.016923	Def6
ENSMUSG00000002289	70.55986	24.21316	1.5431	1.20E-07	2.36E-06	Angptl4
ENSMUSG00000002297	367.3927	282.7322	0.37789	0.011065	0.049282	Dbf4
ENSMUSG00000002395	835.2665	653.166	0.35479	0.000866	0.005939	Use1
ENSMUSG00000002477	506.2617	402.3328	0.33149	0.004765	0.024615	Snrpd1
ENSMUSG00000002489	256.1458	181.1504	0.49978	0.000747	0.005253	Tiam1
ENSMUSG00000002603	234.2219	135.0579	0.7943	6.04E-07	1.02E-05	Tgfb1
ENSMUSG00000002633	30.64068	13.40308	1.1929	0.00356	0.019382	Shh
ENSMUSG00000002688	53.14478	21.4991	1.3057	3.36E-05	0.00036	Prkd1
ENSMUSG00000002825	205.1803	131.2993	0.64403	0.000119	0.00109	Qtrt1
ENSMUSG00000002847	77.77596	39.1383	0.99074	0.000349	0.002743	Pla1a
ENSMUSG00000002985	3389.417	2866.184	0.24191	0.004534	0.023665	Apoe
ENSMUSG00000003131	818.2614	639.2963	0.35608	0.000668	0.004785	Pafah1b2

ENSMUSG00000003153	68.22402	36.81727	0.8899	0.000613	0.004457	Slc2a3
ENSMUSG00000003226	2108.508	1726.98	0.28797	0.003921	0.021028	Ranbp2
ENSMUSG00000003418	85.10942	46.09924	0.88458	0.001172	0.007657	St8sia6
ENSMUSG00000003458	1716.895	1431.216	0.26256	0.005841	0.029132	Ncstn
ENSMUSG00000003484	63.66974	35.12428	0.85814	0.00169	0.01048	Cyp4f18
ENSMUSG00000003541	541.6478	144.8595	1.9027	5.56E-05	0.00056	Ier3
ENSMUSG00000003573	68.36068	35.51792	0.94462	0.000715	0.005068	Homer3
ENSMUSG00000003617	637.9791	204.2402	1.6432	5.21E-39	1.69E-36	Cp
ENSMUSG00000003824	74.89819	41.64234	0.84688	0.002184	0.012967	Syce2
ENSMUSG00000003848	277.0118	195.8166	0.50044	0.00116	0.007592	Nob1
ENSMUSG00000003882	64.9939	25.19579	1.3671	4.28E-06	5.84E-05	Il7r
ENSMUSG00000004032	528.988	306.0455	0.78949	1.52E-05	0.000177	Gstm5
ENSMUSG00000004035	118.2172	71.04787	0.73458	0.007618	0.036439	Gstm7
ENSMUSG00000004040	2069.706	1745.341	0.24592	0.00888	0.041257	Stat3
ENSMUSG00000004098	212.116	138.5512	0.61443	0.000136	0.001223	Col5a3
ENSMUSG00000004285	755.1405	570.1411	0.40543	0.000186	0.001607	Atp6v1f
ENSMUSG00000004319	1792.536	1350.023	0.40902	1.91E-05	0.000218	Clcn3
ENSMUSG00000004328	145.2231	91.49516	0.6665	0.000607	0.004424	Hif3a
ENSMUSG00000004371	46.86155	5.674611	3.0458	5.29E-12	2.31E-10	Il11
ENSMUSG00000004552	854.7913	518.013	0.72258	5.55E-09	1.46E-07	Ctse
ENSMUSG00000004609	55.15441	27.37004	1.0109	0.000602	0.004394	Cd33
ENSMUSG00000004709	48.49935	23.53489	1.0432	0.001187	0.007738	Cd244
ENSMUSG00000004771	1836.615	1412.008	0.3793	0.000111	0.001021	Rab11a
ENSMUSG00000004788	451.6109	367.3042	0.2981	0.010701	0.047998	Eif2b2
ENSMUSG00000004814	14.9354	2.117741	2.8181	0.000273	0.002222	Ccl24
ENSMUSG00000004837	64.3152	34.06603	0.91683	0.002111	0.012624	Grap
ENSMUSG00000004988	108.3322	19.35024	2.485	3.70E-16	2.62E-14	Fxyd4
ENSMUSG00000005054	825.8692	549.5863	0.58757	1.27E-07	2.49E-06	Cstb
ENSMUSG00000005087	883.6141	624.83	0.49995	1.59E-05	0.000185	Cd44
ENSMUSG00000005124	64.83085	40.20923	0.68915	0.010933	0.048792	Wisp1
ENSMUSG00000005125	1681.021	1294.952	0.37644	0.007166	0.03466	Ndrg1
ENSMUSG00000005355	263.681	172.2547	0.61425	5.21E-05	0.00053	Casp14
ENSMUSG00000005364	17.62375	5.598344	1.6544	0.004246	0.022395	Il5ra
ENSMUSG00000005413	194.1657	62.20968	1.6421	1.54E-09	4.53E-08	Hmox1
ENSMUSG00000005483	647.9628	507.9438	0.35124	0.002408	0.014027	Dnajb1
ENSMUSG00000005503	140.0688	38.11692	1.8776	6.47E-08	1.36E-06	Evx1
ENSMUSG00000005553	13.20636	3.265237	2.016	0.004588	0.023901	Atp4a
ENSMUSG00000005609	950.3664	660.0458	0.52592	8.51E-06	0.000108	Ctr9
ENSMUSG00000005610	10326.3	8400.483	0.29778	0.00787	0.037446	Eif4g2
ENSMUSG00000005656	723.3415	562.8027	0.36205	0.002572	0.014818	Snx6
ENSMUSG00000005800	75.86437	1.807031	5.3917	9.25E-30	1.72E-27	Mmp8
ENSMUSG00000005846	859.7368	585.5608	0.55408	1.99E-05	0.000225	Rsl1d1
ENSMUSG00000005958	403.9791	303.1751	0.41413	0.004036	0.021479	Ephb3
ENSMUSG00000006205	765.4817	366.4345	1.0628	1.46E-05	0.000172	Htra1
ENSMUSG00000006221	149.6667	92.19239	0.69903	0.000281	0.002276	Hspb7
ENSMUSG00000006273	1185.628	960.9671	0.30309	0.003152	0.017488	Atp6v1b2
ENSMUSG00000006333	7022.086	5629.896	0.31879	0.000265	0.002164	Rps9
ENSMUSG00000006403	71.18582	39.36608	0.85464	0.001618	0.010078	Adamts4
ENSMUSG00000006411	305.6492	51.93873	2.557	3.21E-18	2.92E-16	Pvrl4
ENSMUSG00000006435	85.35816	44.79319	0.93025	0.006072	0.030119	Neurl1a
ENSMUSG00000006445	415.5028	194.4363	1.0956	1.37E-11	5.51E-10	Epha2
ENSMUSG00000006494	1215.093	797.3621	0.60776	4.32E-09	1.17E-07	Pdk1

ENSMUSG00000006519	1521.179	715.6637	1.0878	1.77E-05	0.000203	Cyba
ENSMUSG00000006522	19.67892	7.075628	1.4757	0.005902	0.029391	Itih3
ENSMUSG00000006720	29.91641	12.28068	1.2845	0.001342	0.008585	Zfp184
ENSMUSG00000006728	1331.122	908.2518	0.55148	0.000426	0.003264	Cdk4
ENSMUSG00000006740	3452.865	2625.126	0.39541	1.07E-05	0.000131	Kif5b
ENSMUSG00000006777	622.6085	178.8062	1.7999	7.62E-07	1.26E-05	Krt23
ENSMUSG00000006800	937.7172	746.3212	0.32936	0.002034	0.012252	Sulf2
ENSMUSG00000006932	10608.17	8423.855	0.33262	0.000112	0.00103	Ctnnb1
ENSMUSG00000007039	175.7141	73.95822	1.2484	0.001362	0.008685	Ddah2
ENSMUSG00000007041	3542.613	2634.424	0.42733	2.16E-06	3.18E-05	Clic1
ENSMUSG00000007207	74.46829	33.13996	1.1681	0.003888	0.020908	Stx1a
ENSMUSG00000007613	671.3065	440.8056	0.60683	2.25E-07	4.21E-06	Tgfbr1
ENSMUSG00000007659	646.6925	454.0414	0.51026	0.000253	0.002083	Bcl2l1
ENSMUSG00000007682	70.53353	8.565589	3.0417	3.63E-17	2.90E-15	Dio2
ENSMUSG00000007836	2636.784	2044.143	0.36728	0.00034	0.002685	Hnrnpa0
ENSMUSG00000007891	3827.873	2700.407	0.50337	0.00014	0.001257	Ctsd
ENSMUSG00000007892	6181.327	4784.364	0.36959	9.92E-05	0.000927	Rplp1
ENSMUSG00000007908	32.06286	15.03251	1.0928	0.008494	0.039828	Hmgcll1
ENSMUSG00000008090	382.5181	125.923	1.603	1.68E-06	2.56E-05	Fgfrl1
ENSMUSG00000008136	316.7483	233.5731	0.43946	0.002502	0.014491	Fhl2
ENSMUSG00000008318	40.80632	20.17401	1.0163	0.002327	0.013672	Relt
ENSMUSG00000008333	434.9978	339.8529	0.3561	0.006961	0.033908	Snrpb2
ENSMUSG00000008393	1591.362	1025.904	0.63337	3.17E-10	1.04E-08	Carhsp1
ENSMUSG00000008398	315.4556	146.1582	1.1099	2.02E-08	4.69E-07	Elk3
ENSMUSG00000008475	3567.659	2865.97	0.31595	0.000529	0.003928	Arpc5
ENSMUSG00000008668	5752.009	4028.008	0.514	3.10E-09	8.59E-08	Rps18
ENSMUSG00000008682	3522.341	2614.953	0.42975	0.000499	0.003728	Rpl10
ENSMUSG00000008683	4559.468	3131.669	0.54193	1.11E-09	3.31E-08	Rps15a
ENSMUSG00000008734	58.72999	33.46974	0.81124	0.00545	0.027533	Gprc5b
ENSMUSG00000008859	983.7554	784.6068	0.32633	0.002444	0.014182	Rala
ENSMUSG00000008999	176.0792	102.9135	0.77479	1.51E-05	0.000176	Bmp7
ENSMUSG00000009030	555.3681	452.9782	0.294	0.009291	0.042877	Pdcl
ENSMUSG00000009207	232.6297	164.7547	0.49772	0.001243	0.008039	Lnp
ENSMUSG00000009248	91.92195	38.83361	1.2431	9.13E-05	0.000862	Ascl2
ENSMUSG00000009378	91.08031	50.25146	0.85797	0.00049	0.003669	Slc16a12
ENSMUSG00000009418	437.7769	343.3211	0.35064	0.008535	0.039952	Nav1
ENSMUSG00000009470	2051.97	1573.597	0.38294	3.58E-05	0.00038	Tnpo1
ENSMUSG00000009535	405.1666	275.5174	0.55637	1.32E-05	0.000158	Rnmt
ENSMUSG00000009549	533.0673	416.3706	0.35645	0.001466	0.009263	Srp14
ENSMUSG00000009563	499.5881	338.6924	0.56076	4.60E-06	6.22E-05	Tor2a
ENSMUSG00000009628	127.9447	29.86268	2.0991	1.21E-09	3.59E-08	Tex15
ENSMUSG00000009630	1480.656	1189.033	0.31645	0.001113	0.007321	Ppp2cb
ENSMUSG00000009633	76.26941	34.1035	1.1612	6.60E-05	0.000648	G0s2
ENSMUSG00000009687	264.7064	165.8908	0.67416	8.25E-06	0.000105	Fxyd5
ENSMUSG00000009739	146.2083	92.40245	0.66202	0.004217	0.022269	Pou6f1
ENSMUSG00000009905	423.0131	293.5378	0.52716	3.39E-05	0.000362	Kdsr
ENSMUSG00000009927	5090.503	3754.189	0.43931	4.72E-07	8.18E-06	Rps25
ENSMUSG00000010067	456.935	364.6299	0.32556	0.007997	0.037918	Rassf1
ENSMUSG00000010175	939.3103	172.2928	2.4467	6.61E-10	2.04E-08	Prox1
ENSMUSG00000010760	33.09398	9.047193	1.871	1.29E-05	0.000155	Phlda2
ENSMUSG00000011148	41.08697	14.41109	1.5115	5.52E-05	0.000557	Adssl1
ENSMUSG00000011305	17.43434	4.710726	1.8879	0.002201	0.013047	Plin5

ENSMUSG00000011463	11.84909	1.629439	2.8623	0.001063	0.007067	Cpb1
ENSMUSG00000011837	155.3237	112.6838	0.463	0.010746	0.048128	Snapc2
ENSMUSG00000012405	2913.563	2113.256	0.46332	2.81E-07	5.16E-06	Rpl15
ENSMUSG00000012428	812.0611	241.5409	1.7493	7.18E-47	3.77E-44	Steap4
ENSMUSG00000012848	8019.629	5920.148	0.4379	3.57E-05	0.00038	Rps5
ENSMUSG00000012889	38.60743	15.24822	1.3402	0.000748	0.005255	Podnl1
ENSMUSG00000013275	446.8738	296.5856	0.59142	0.000891	0.006081	Slc41a1
ENSMUSG00000013483	362.6316	247.5694	0.55067	2.33E-05	0.000259	Card14
ENSMUSG00000013698	601.5702	382.2206	0.65433	1.45E-08	3.47E-07	Pea15a
ENSMUSG00000013766	128.05	67.01571	0.93414	7.98E-06	0.000102	Ly6g6e
ENSMUSG00000013974	25.26413	5.56023	2.1839	2.53E-05	0.000279	Mcemp1
ENSMUSG00000014444	412.7423	314.8082	0.39077	0.001854	0.01133	Piezo1
ENSMUSG00000014496	661.1673	418.0438	0.66136	1.83E-08	4.29E-07	Ankrd28
ENSMUSG00000014504	493.2193	394.8026	0.3211	0.009985	0.04546	Srp19
ENSMUSG00000014601	547.5382	423.4346	0.37082	0.001778	0.01091	Strip1
ENSMUSG00000014725	51.11578	1.807031	4.8221	1.94E-19	1.92E-17	Adam28
ENSMUSG00000014769	1753.282	1351.883	0.37509	0.00015	0.001333	Psmb1
ENSMUSG00000014813	60.466	17.42917	1.7946	2.58E-08	5.86E-07	Stc1
ENSMUSG00000015120	1402.964	1131.809	0.30985	0.001783	0.010942	Ube2i
ENSMUSG00000015134	876.4754	107.3577	3.0293	2.26E-93	3.36E-90	Aldh1a3
ENSMUSG00000015243	625.4522	263.6759	1.2461	5.05E-17	3.99E-15	Abca1
ENSMUSG00000015312	240.5717	169.948	0.50137	0.000607	0.004427	Gadd45b
ENSMUSG00000015340	301.9165	200.6837	0.58923	0.000151	0.00134	Cybb
ENSMUSG00000015468	246.013	122.536	1.0055	2.10E-10	7.02E-09	Notch4
ENSMUSG00000015647	947.6305	467.7923	1.0185	4.51E-07	7.86E-06	Lama5
ENSMUSG00000015652	161.4122	96.5617	0.74123	6.76E-05	0.000661	Steap1
ENSMUSG00000015653	482.5443	249.1544	0.95362	4.72E-14	2.61E-12	Steap2
ENSMUSG00000015671	1392.989	1115.307	0.32074	0.001471	0.009288	Psma2
ENSMUSG00000015709	64.39407	25.79177	1.32	3.61E-06	5.04E-05	Arnt2
ENSMUSG00000015766	3505.797	2905.811	0.2708	0.002744	0.015621	Eps8
ENSMUSG00000015837	2584.91	2204.425	0.22971	0.010984	0.048986	Sqstm1
ENSMUSG00000015882	377.8478	294.6165	0.35897	0.007331	0.035275	Lcorl
ENSMUSG00000015950	120.5726	70.86452	0.76677	0.000365	0.002857	Ncf1
ENSMUSG00000016024	301.7151	86.80245	1.7974	3.46E-28	5.99E-26	Lbp
ENSMUSG00000016028	1238.724	975.2853	0.34496	0.002214	0.013092	Celsr1
ENSMUSG00000016239	86.16689	48.29321	0.83531	0.000851	0.005854	Lonrf3
ENSMUSG00000016382	725.6259	349.1878	1.0552	2.48E-15	1.60E-13	Pls3
ENSMUSG00000016487	611.2975	461.7079	0.40489	0.001086	0.00719	Ppfibp1
ENSMUSG00000016526	77.8739	24.30215	1.6801	8.65E-08	1.77E-06	Dyrk3
ENSMUSG00000016534	3864.951	1930.996	1.0011	1.89E-18	1.77E-16	Lamp2
ENSMUSG00000016541	346.4366	232.3413	0.57635	1.69E-05	0.000196	Atxn10
ENSMUSG00000016559	7746.161	5811.367	0.4146	1.40E-06	2.17E-05	H3f3b
ENSMUSG00000016763	253.2098	197.4763	0.35865	0.008911	0.04138	Scube1
ENSMUSG00000016918	341.5815	180.3197	0.92167	9.35E-11	3.31E-09	Sulf1
ENSMUSG00000016933	455.3104	319.3037	0.51192	0.001192	0.007763	Plcg1
ENSMUSG00000016942	24.6836	2.174592	3.5047	0.000112	0.001033	Tmprss6
ENSMUSG00000016946	1002.075	800.807	0.32346	0.001004	0.00674	Kctd5
ENSMUSG00000017002	1244.881	629.68	0.98332	6.11E-21	6.81E-19	Slpi
ENSMUSG00000017057	1577.103	1245.965	0.34001	0.004482	0.023428	Il13ra1
ENSMUSG00000017144	642.8198	494.1194	0.37955	0.002342	0.01373	Rnd3
ENSMUSG00000017204	132.9438	27.00282	2.2996	4.28E-06	5.84E-05	Gsdma
ENSMUSG00000017286	639.4065	462.5517	0.46712	7.71E-05	0.000742	Glod4

ENSMUSG00000017311	707.9043	467.2548	0.59934	1.45E-07	2.82E-06	Pyy
ENSMUSG00000017376	266.1286	206.5936	0.36533	0.01011	0.045934	Nlk
ENSMUSG00000017386	581.7629	416.3162	0.48275	0.000998	0.006716	Traf4
ENSMUSG00000017390	325.1587	189.4885	0.77903	7.67E-08	1.59E-06	Aldoc
ENSMUSG00000017404	7656.421	6256.279	0.29137	0.000665	0.00477	Rpl19
ENSMUSG00000017421	1924.342	1583.87	0.28091	0.003263	0.018014	Zfp207
ENSMUSG00000017466	902.1489	721.3938	0.32258	0.002395	0.013983	Timp2
ENSMUSG00000017491	65.77042	25.41119	1.372	0.003217	0.017803	Rarb
ENSMUSG00000017493	2186.976	1155.377	0.92057	9.99E-07	1.59E-05	Igfbp4
ENSMUSG00000017724	137.8437	80.07001	0.7837	5.70E-05	0.000572	Etv4
ENSMUSG00000017737	333.1388	28.78407	3.5328	3.90E-39	1.34E-36	Mmp9
ENSMUSG00000017774	1747.94	1330.723	0.39345	2.93E-05	0.000317	Myo1c
ENSMUSG00000017776	2753.473	2256.183	0.28737	0.001619	0.010082	Crk
ENSMUSG00000018012	175.4931	65.00032	1.4329	0.001502	0.009458	Rac3
ENSMUSG00000018171	2121.281	1624.467	0.38497	2.03E-05	0.000229	Vmp1
ENSMUSG00000018199	334.5353	234.1032	0.51501	0.000106	0.000988	Trove2
ENSMUSG00000018286	1181.598	984.0921	0.26387	0.009457	0.043484	Psmb6
ENSMUSG00000018339	730.4031	548.0989	0.41426	0.000147	0.001311	Gpx3
ENSMUSG00000018387	33.8445	16.18637	1.0641	0.004004	0.021375	Shroom1
ENSMUSG00000018395	126.6401	86.43587	0.55103	0.010723	0.048059	Kif3a
ENSMUSG00000018417	797.479	625.8545	0.34962	0.000843	0.005806	Myo1b
ENSMUSG00000018425	389.8124	295.925	0.39755	0.006158	0.030505	Dhx40
ENSMUSG00000018570	81.99137	40.49556	1.0177	0.00329	0.018144	2810408A11Rik
ENSMUSG00000018593	3311.82	2158.17	0.61782	1.45E-11	5.81E-10	Sparc
ENSMUSG00000018623	1804.631	251.2866	2.8443	4.19E-07	7.34E-06	Mmp7
ENSMUSG00000018821	211.4245	78.86592	1.4227	2.24E-05	0.00025	Avpi1
ENSMUSG00000018848	1451.463	1115.519	0.37979	0.000155	0.001373	Rars
ENSMUSG00000018862	11.24231	1.211046	3.2146	0.000724	0.005121	Otop3
ENSMUSG00000018906	122.5489	81.59104	0.58688	0.004565	0.02379	P4ha2
ENSMUSG00000019066	1866.476	1454.38	0.35991	0.000155	0.001373	Rab3d
ENSMUSG00000019122	273.3255	181.4297	0.59121	5.06E-05	0.000517	Ccl9
ENSMUSG00000019235	32.55904	11.96395	1.4444	0.003435	0.0188	Rps6kl1
ENSMUSG00000019433	693.3841	555.5455	0.31975	0.002807	0.015924	Gipc1
ENSMUSG00000019564	162.8677	89.03559	0.87125	0.000373	0.00291	Arid3a
ENSMUSG00000019768	106.3156	51.39956	1.0485	3.90E-06	5.39E-05	Esr1
ENSMUSG00000019772	301.433	166.6437	0.85507	0.002597	0.01493	Vip
ENSMUSG00000019777	824.7392	625.1697	0.39969	0.000152	0.00135	Hdac2
ENSMUSG00000019779	1173.231	826.3485	0.50566	2.21E-06	3.24E-05	Frk
ENSMUSG00000019808	59.59799	37.01992	0.68696	0.010123	0.045969	Adat2
ENSMUSG00000019814	422.2153	263.0471	0.68266	2.67E-07	4.92E-06	Ltv1
ENSMUSG00000019817	64.62194	30.96506	1.0614	0.000135	0.001215	Plagl1
ENSMUSG00000019832	397.4932	256.2866	0.63317	7.06E-07	1.18E-05	Rab32
ENSMUSG00000019849	630.2329	505.209	0.319	0.005606	0.028168	Prep
ENSMUSG00000019851	3097.832	2441.287	0.34362	0.000517	0.003849	Perp
ENSMUSG00000019853	197.8853	89.87836	1.1386	1.85E-10	6.24E-09	Hebp2
ENSMUSG00000019857	214.7132	158.656	0.43651	0.004638	0.024098	Asf1a
ENSMUSG00000019872	6267.599	2847.793	1.1381	3.00E-19	2.95E-17	Smpdl3a
ENSMUSG00000019877	1749.148	1293.957	0.43486	0.000592	0.004339	Serinc1
ENSMUSG00000019923	532.4593	418.5133	0.3474	0.003625	0.019696	Zwint
ENSMUSG00000019929	2297.671	1406.418	0.70815	1.12E-05	0.000136	Dcn
ENSMUSG00000019942	646.5662	484.1248	0.41742	9.48E-05	0.000891	Cdk1
ENSMUSG00000019975	258.957	149.4633	0.79292	9.98E-06	0.000123	Ikbip

ENSMUSG00000019978	850.2988	622.6921	0.44945	1.92E-05	0.000218	Epb4.1l2
ENSMUSG00000019979	727.6447	564.1984	0.36703	0.000789	0.005497	Apaf1
ENSMUSG00000019987	450.6706	27.17371	4.0518	7.85E-71	7.01E-68	Arg1
ENSMUSG00000020000	26.26897	10.33451	1.3459	0.003613	0.019641	Moxd1
ENSMUSG00000020015	261.685	173.8088	0.59033	8.86E-05	0.000838	Cdk17
ENSMUSG00000020023	1996.199	1677.278	0.25113	0.010034	0.045636	Tmcc3
ENSMUSG00000020032	105.5003	41.40119	1.3495	9.78E-09	2.41E-07	Nuak1
ENSMUSG00000020042	45.54984	24.94193	0.86887	0.006322	0.031236	Btbd11
ENSMUSG00000020053	365.6198	183.5093	0.99449	9.05E-13	4.30E-11	Igf1
ENSMUSG00000020077	472.2018	215.4448	1.1321	2.31E-17	1.90E-15	Srgn
ENSMUSG00000020086	63.80644	35.43529	0.84852	0.010516	0.047343	H2afy2
ENSMUSG00000020088	1521.748	1278.294	0.25151	0.010702	0.047998	Sar1a
ENSMUSG00000020092	101.318	57.89865	0.80729	0.000257	0.00211	Pald1
ENSMUSG00000020107	495.7979	382.4727	0.3744	0.008086	0.038313	Anapc16
ENSMUSG00000020108	1623.61	843.4353	0.94486	3.26E-15	2.07E-13	Ddit4
ENSMUSG00000020120	249.0592	142.7864	0.80263	3.21E-07	5.79E-06	Plek
ENSMUSG00000020121	358.7292	276.8496	0.37379	0.003639	0.019759	Srgap1
ENSMUSG00000020122	561.5149	441.2541	0.34771	0.00181	0.011096	Egfr
ENSMUSG00000020130	735.9553	581.4171	0.34004	0.003065	0.017119	Tbc1d15
ENSMUSG00000020140	299.8302	141.0987	1.0874	0.006356	0.031363	Lgr5
ENSMUSG00000020152	3815.626	3212.648	0.24816	0.005895	0.029361	Actr2
ENSMUSG00000020154	287.3282	193.8256	0.56794	0.000116	0.001063	Ptprb
ENSMUSG00000020159	55.13491	5.611062	3.2966	3.34E-10	1.08E-08	Gabrp
ENSMUSG00000020167	734.1048	485.7088	0.59589	0.002318	0.013629	Tcf3
ENSMUSG00000020171	417.7525	302.4763	0.46583	0.000388	0.003008	Yeats4
ENSMUSG00000020189	275.537	183.6806	0.58505	8.29E-05	0.000792	Osbpl8
ENSMUSG00000020205	1058.097	328.7381	1.6865	3.15E-05	0.00034	Phlda1
ENSMUSG00000020218	1078.726	156.8423	2.7819	6.00E-10	1.87E-08	Wif1
ENSMUSG00000020224	571.4068	416.2569	0.45704	8.02E-05	0.000769	Llph
ENSMUSG00000020250	3621.921	2759.849	0.39217	1.45E-05	0.00017	Txnrd1
ENSMUSG00000020255	405.5843	275.1558	0.55975	1.27E-05	0.000152	D10Wsu102e
ENSMUSG00000020275	429.1118	115.4453	1.8941	0.002121	0.012673	Rel
ENSMUSG00000020282	334.1052	251.8576	0.40769	0.003343	0.018381	Rhbdf1
ENSMUSG00000020303	606.5233	273.6347	1.1483	1.78E-20	1.90E-18	Stc2
ENSMUSG00000020305	145.8105	96.42091	0.59668	0.001283	0.008256	Asb3
ENSMUSG00000020310	54.65826	22.3874	1.2878	0.001311	0.008399	Madcam1
ENSMUSG00000020311	577.1609	401.7371	0.52272	1.01E-05	0.000124	Erlec1
ENSMUSG00000020328	492.1037	364.8111	0.43181	0.000257	0.002109	Nudcd2
ENSMUSG00000020372	10859.19	8385.822	0.37289	1.63E-05	0.000189	Gnb2l1
ENSMUSG00000020388	217.0623	84.37502	1.3632	1.36E-05	0.000161	Pdlim4
ENSMUSG00000020392	576.906	380.4912	0.60047	4.56E-07	7.93E-06	Cdkn2aipnl
ENSMUSG00000020419	12.89952	3.512398	1.8768	0.009001	0.041768	Hormad2
ENSMUSG00000020427	528.0685	362.141	0.54417	2.77E-06	3.98E-05	Igfbp3
ENSMUSG00000020430	820.9692	559.2279	0.55389	2.83E-07	5.19E-06	Pes1
ENSMUSG00000020431	136.7019	36.72256	1.8963	2.34E-07	4.36E-06	Adcy1
ENSMUSG00000020444	643.7288	509.8978	0.33624	0.003436	0.0188	Guk1
ENSMUSG00000020453	217.003	111.5998	0.95938	1.64E-08	3.88E-07	Patz1
ENSMUSG00000020458	1856.854	1076.617	0.78635	4.39E-16	3.09E-14	Rtn4
ENSMUSG00000020460	7494.336	5230.709	0.51879	7.29E-07	1.21E-05	Rps27a
ENSMUSG00000020462	234.3656	162.5549	0.52783	0.000587	0.004311	Cfap36
ENSMUSG00000020536	407.5093	311.7202	0.38658	0.001857	0.011341	Llgl1
ENSMUSG00000020547	979.9588	614.1652	0.67409	7.29E-09	1.85E-07	Bzw2

ENSMUSG00000020561	212.4809	153.9893	0.4645	0.005036	0.025782	Twistnb
ENSMUSG00000020570	2991.444	2290.77	0.38501	0.001349	0.008615	Sypl
ENSMUSG00000020592	3004.862	1676.963	0.84145	4.35E-08	9.48E-07	Sdc1
ENSMUSG00000020594	1934.889	1632.313	0.24533	0.006121	0.030337	Pum2
ENSMUSG00000020601	158.6713	80.29805	0.9826	1.56E-07	3.00E-06	Trib2
ENSMUSG00000020604	67.28414	39.20787	0.77912	0.006063	0.030083	Arsg
ENSMUSG00000020607	198.3049	73.12321	1.4393	0.002692	0.015392	Fam84a
ENSMUSG00000020612	1988.157	1624.957	0.29103	0.002129	0.012694	Prkar1a
ENSMUSG00000020644	383.6991	198.4666	0.95108	1.07E-11	4.42E-10	Id2
ENSMUSG00000020646	168.595	56.8836	1.5675	3.06E-15	1.96E-13	Mboat2
ENSMUSG00000020650	245.0399	123.1127	0.99304	6.13E-10	1.90E-08	Bcap29
ENSMUSG00000020653	124.4481	66.05751	0.91375	8.75E-06	0.00011	Klf11
ENSMUSG00000020674	455.0161	301.1628	0.59537	3.36E-06	4.74E-05	Pxdn
ENSMUSG00000020677	488.2459	326.5997	0.58008	4.04E-06	5.56E-05	Ddx52
ENSMUSG00000020694	467.3093	364.0329	0.36031	0.002799	0.015891	Tlk2
ENSMUSG00000020696	407.3714	270.9793	0.58816	1.26E-05	0.000151	Rffl
ENSMUSG00000020717	428.3096	293.5801	0.5449	2.15E-05	0.000241	Pecam1
ENSMUSG00000020728	89.14257	16.33853	2.4478	8.45E-17	6.45E-15	Cep112
ENSMUSG00000020736	1031.676	810.8757	0.34744	0.000735	0.00519	Nt5c
ENSMUSG00000020738	1899.59	1436.504	0.40313	1.04E-05	0.000128	Sumo2
ENSMUSG00000020755	396.0324	286.6001	0.46658	0.000208	0.001766	Sap30bp
ENSMUSG00000020794	656.6461	496.9845	0.40192	0.000311	0.002482	Ube2g1
ENSMUSG00000020828	101.5985	62.43209	0.70252	0.001575	0.009844	Pld2
ENSMUSG00000020829	156.4074	96.10516	0.70262	0.000165	0.001455	Slc46a1
ENSMUSG00000020844	247.5787	157.1151	0.65606	0.001018	0.006813	Nxn
ENSMUSG00000020916	361.6475	36.50652	3.3084	3.77E-05	0.000399	Krt36
ENSMUSG00000020937	78.39654	47.06343	0.73618	0.008161	0.03854	Plcd3
ENSMUSG00000020946	905.7917	750.2	0.2719	0.010438	0.047062	Gosr2
ENSMUSG00000020949	265.3256	207.0298	0.35793	0.01066	0.047896	Fkbp3
ENSMUSG00000020962	411.2166	330.5434	0.31506	0.005923	0.029479	Gtf2a1
ENSMUSG00000020978	436.9297	290.1318	0.59069	2.80E-06	4.03E-05	Klhdc2
ENSMUSG00000021003	135.0381	89.64925	0.591	0.001656	0.010285	Galc
ENSMUSG00000021022	266.69	182.0136	0.55112	0.000234	0.001959	Ppp2r3c
ENSMUSG00000021039	539.1601	404.8768	0.41323	0.000846	0.005821	Snw1
ENSMUSG00000021069	115.196	71.76994	0.68264	0.000642	0.004646	Pygl
ENSMUSG00000021072	1245.022	980.3817	0.34476	0.000764	0.005358	Tmx1
ENSMUSG00000021091	664.6906	433.8481	0.61549	2.60E-05	0.000286	Serpina3n
ENSMUSG00000021103	179.792	132.2428	0.44314	0.008647	0.040368	Mnat1
ENSMUSG00000021108	155.1994	106.5394	0.54274	0.002711	0.015469	Prkch
ENSMUSG00000021109	3306.648	1418.643	1.2209	4.92E-18	4.37E-16	Hif1a
ENSMUSG00000021115	319.9979	244.6665	0.38725	0.00388	0.020876	Vrk1
ENSMUSG00000021116	854.6427	662.4113	0.36759	0.000514	0.003834	Eif2s1
ENSMUSG00000021118	614.8105	391.7856	0.65008	3.54E-08	7.83E-07	Plek2
ENSMUSG00000021130	69.31307	36.93763	0.90804	0.001202	0.007818	Galnt16
ENSMUSG00000021131	1151.782	872.251	0.40105	8.61E-05	0.000817	Erh
ENSMUSG00000021136	78.76135	38.97944	1.0148	0.000104	0.000968	Smoc1
ENSMUSG00000021149	634.1418	390.9993	0.69764	6.54E-09	1.67E-07	Gtpbp4
ENSMUSG00000021187	837.8329	357.1319	1.2302	6.37E-14	3.44E-12	Tc2n
ENSMUSG00000021189	401.8317	317.6728	0.33905	0.008588	0.040111	Atxn3
ENSMUSG00000021190	1213.106	805.6807	0.59043	3.97E-09	1.08E-07	Lgmn
ENSMUSG00000021215	3516.604	2763.03	0.34793	0.000109	0.00101	Net1
ENSMUSG00000021218	5279.703	4350.791	0.27918	0.004135	0.021906	Gdi2

ENSMUSG00000021242	2208.527	1755.843	0.33092	0.000438	0.00334	Npc2
ENSMUSG00000021243	430.8015	319.4287	0.43153	0.000866	0.005939	Fcf1
ENSMUSG00000021262	170.8272	76.25318	1.1637	5.28E-10	1.66E-08	Evl
ENSMUSG00000021282	3106.902	2616.564	0.2478	0.006467	0.031788	Eif5
ENSMUSG00000021306	785.6297	394.603	0.99345	1.57E-18	1.47E-16	Gpr137b
ENSMUSG00000021319	33.42022	16.19273	1.0454	0.007207	0.03481	Sfrp4
ENSMUSG00000021326	559.9555	410.5696	0.44769	0.000111	0.001027	Trim27
ENSMUSG00000021360	463.2499	342.2913	0.43657	0.000441	0.003361	Gcnt2
ENSMUSG00000021377	1319.66	1089.14	0.27698	0.004553	0.02374	Dek
ENSMUSG00000021385	313.0292	180.4721	0.79452	3.63E-08	7.99E-07	Ippk
ENSMUSG00000021395	960.9404	680.3675	0.49813	1.38E-06	2.14E-05	Spin1
ENSMUSG00000021417	526.9198	357.9604	0.55778	6.52E-06	8.47E-05	Eci2
ENSMUSG00000021428	254.6778	187.726	0.44004	0.003	0.016842	Riok1
ENSMUSG00000021451	329.4916	233.5965	0.49622	0.00022	0.001855	Sema4d
ENSMUSG00000021453	146.8678	99.45874	0.56235	0.002454	0.014234	Gadd45g
ENSMUSG00000021477	1599.468	996.3173	0.68292	2.28E-12	1.04E-10	Ctsl
ENSMUSG00000021493	615.9857	423.6526	0.54002	0.01021	0.046257	Pdlim7
ENSMUSG00000021508	336.3819	149.8679	1.1664	1.22E-15	8.27E-14	Cxcl14
ENSMUSG00000021537	574.9035	432.8088	0.40959	0.000295	0.00237	Cetn3
ENSMUSG00000021539	20.66428	1.027096	4.3305	0.010507	0.047326	Lect2
ENSMUSG00000021540	757.0506	592.9113	0.35257	0.001197	0.007784	Smad5
ENSMUSG00000021546	4743.496	3982.938	0.25212	0.00397	0.021234	Hnrnpk
ENSMUSG00000021553	372.0591	173.2266	1.1029	2.77E-14	1.60E-12	Slc28a3
ENSMUSG00000021585	2453.542	1870.913	0.39112	0.000664	0.00477	Cast
ENSMUSG00000021594	238.685	108.849	1.1328	3.80E-11	1.42E-09	Srd5a1
ENSMUSG00000021595	1200.192	949.1955	0.33849	0.00067	0.004797	Nsun2
ENSMUSG00000021596	77.02545	39.93027	0.94785	0.000467	0.003524	Mctp1
ENSMUSG00000021604	44.08189	8.927131	2.3039	0.000869	0.005952	Irx4
ENSMUSG00000021643	108.9128	65.88726	0.7251	0.000821	0.005681	Serf1
ENSMUSG00000021660	4406.857	3528.434	0.32072	0.000513	0.003824	Btf3
ENSMUSG00000021701	329.7	183.0273	0.84909	2.13E-08	4.91E-07	Plk2
ENSMUSG00000021715	173.5279	120.3243	0.52824	0.004034	0.021477	Cwc27
ENSMUSG00000021720	118.3998	40.82429	1.5362	2.54E-11	9.85E-10	Rnf180
ENSMUSG00000021728	113.3757	50.34672	1.1711	8.25E-08	1.69E-06	Emb
ENSMUSG00000021745	577.8071	410.36	0.4937	1.82E-05	0.000209	Ptprg
ENSMUSG00000021765	46.509	15.07067	1.6258	5.96E-06	7.86E-05	Fst
ENSMUSG00000021774	318.2619	202.7829	0.65028	4.71E-06	6.35E-05	Ube2e1
ENSMUSG00000021806	120.7552	80.81209	0.57944	0.006744	0.032995	Nid2
ENSMUSG00000021814	2051.365	1560.988	0.39412	3.54E-05	0.000377	Anxa7
ENSMUSG00000021822	267.277	129.6679	1.0435	5.75E-09	1.51E-07	Plau
ENSMUSG00000021830	616.5574	419.793	0.55456	2.19E-06	3.22E-05	Txndc16
ENSMUSG00000021831	1413.903	907.848	0.63916	1.86E-07	3.53E-06	Ero1l
ENSMUSG00000021832	1138.877	921.528	0.30551	0.002624	0.015071	Psmc6
ENSMUSG00000021843	883.6589	715.3572	0.30483	0.002425	0.014109	Ktn1
ENSMUSG00000021890	589.4608	464.296	0.34435	0.002893	0.016313	Eaf1
ENSMUSG00000021917	1169.362	894.1612	0.38711	0.000218	0.001838	Spcs1
ENSMUSG00000021936	460.3865	308.1631	0.57915	3.36E-06	4.74E-05	Mapk8
ENSMUSG00000021950	2662.146	599.2702	2.1513	########	2.99E-98	Anxa8
ENSMUSG00000021969	1013.046	745.2912	0.44282	2.93E-05	0.000317	Zdhhc20
ENSMUSG00000021986	21.40792	4.539494	2.2375	0.000129	0.001172	Amer2
ENSMUSG00000021994	228.2387	140.6819	0.69811	1.83E-05	0.00021	Wnt5a
ENSMUSG00000021998	896.1342	667.9515	0.42397	3.89E-05	0.00041	Lcp1

ENSMUSG00000022008	515.2462	395.3728	0.38205	0.002444	0.014182	Gpalpp1
ENSMUSG00000022009	149.1511	100.5801	0.56843	0.002154	0.012814	Nufip1
ENSMUSG00000022015	26.31465	10.76562	1.2894	0.005256	0.026723	Tnfsf11
ENSMUSG00000022018	123.0784	67.48464	0.86695	1.66E-05	0.000193	Rgcc
ENSMUSG00000022032	96.7043	29.82389	1.6971	7.15E-11	2.58E-09	Scara5
ENSMUSG00000022037	1650.706	671.1127	1.2985	2.52E-07	4.69E-06	Clu
ENSMUSG00000022051	1626.555	859.4036	0.92041	1.28E-19	1.28E-17	Bnip3l
ENSMUSG00000022089	364.8304	264.9681	0.46141	0.000464	0.003502	Bin3
ENSMUSG00000022091	107.6206	71.70736	0.58576	0.008207	0.038729	Sorbs3
ENSMUSG00000022092	112.1815	74.55387	0.58948	0.004614	0.024003	Ppp3cc
ENSMUSG00000022098	299.5822	169.2005	0.82422	1.84E-08	4.31E-07	Bmp1
ENSMUSG00000022111	253.3466	192.0814	0.39939	0.007258	0.034989	Uchl3
ENSMUSG00000022126	70.0704	16.56029	2.0811	1.20E-11	4.92E-10	Irg1
ENSMUSG00000022132	21.26423	3.93715	2.4332	5.29E-05	0.000536	Cldn10
ENSMUSG00000022142	371.7992	301.2066	0.30377	0.008928	0.041442	Nup155
ENSMUSG00000022146	325.5429	160.7416	1.0181	8.73E-12	3.67E-10	Osmr
ENSMUSG00000022151	255.1406	194.4076	0.39221	0.007073	0.034296	Ttc33
ENSMUSG00000022176	73.81578	8.578307	3.1052	0.000595	0.004352	Rem2
ENSMUSG00000022178	179.3688	71.28901	1.3312	1.48E-05	0.000173	Ajuba
ENSMUSG00000022205	1050.908	816.2479	0.36456	0.000769	0.005382	Sub1
ENSMUSG00000022218	45.57603	10.0238	2.1848	1.68E-06	2.57E-05	Tgm1
ENSMUSG00000022221	637.0285	517.3784	0.30014	0.007297	0.035161	Ripk3
ENSMUSG00000022226	144.662	42.8912	1.7539	1.80E-15	1.19E-13	Mcpt2
ENSMUSG00000022227	123.7766	45.2751	1.4509	1.14E-10	4.00E-09	Mcpt1
ENSMUSG00000022247	374.0946	296.7119	0.33434	0.008122	0.038407	Brix1
ENSMUSG00000022280	450.7556	359.2286	0.32744	0.010625	0.047776	Rnf19a
ENSMUSG00000022283	11271.57	8234.538	0.45293	6.97E-05	0.000677	Pabpc1
ENSMUSG00000022297	237.4522	132.0268	0.84681	6.14E-08	1.30E-06	Fzd6
ENSMUSG00000022299	170.9958	123.5752	0.46857	0.007542	0.036124	Slc25a32
ENSMUSG00000022300	556.5996	422.1883	0.39875	0.008496	0.039828	Dcaf13
ENSMUSG00000022312	2122.466	1782.959	0.25147	0.006255	0.030939	Eif3h
ENSMUSG00000022336	2131.231	1450.167	0.55547	1.16E-08	2.82E-07	Eif3e
ENSMUSG00000022346	370.1874	194.9535	0.92513	1.26E-11	5.13E-10	Myc
ENSMUSG00000022372	133.1846	85.58505	0.638	0.001058	0.007035	Sla
ENSMUSG00000022376	391.1977	36.60822	3.4177	1.02E-07	2.03E-06	Adcy8
ENSMUSG00000022378	624.5957	414.8807	0.59023	6.36E-07	1.07E-05	Fam49b
ENSMUSG00000022390	694.9038	534.8945	0.37756	0.00046	0.003482	Zc3h7b
ENSMUSG00000022415	104.0974	55.48322	0.90781	6.98E-05	0.000678	Syngr1
ENSMUSG00000022425	252.707	165.154	0.61365	4.84E-05	0.000496	Enpp2
ENSMUSG00000022433	416.8011	235.144	0.82581	0.000136	0.001228	Csnk1e
ENSMUSG00000022464	152.779	22.47002	2.7654	2.65E-21	3.09E-19	Slc38a4
ENSMUSG00000022488	262.6374	179.1988	0.55151	0.000421	0.003227	Nckap1l
ENSMUSG00000022491	38.47064	1.451848	4.7278	2.22E-15	1.43E-13	Glycam1
ENSMUSG00000022512	29.04854	10.778	1.4304	0.00077	0.005393	Cldn1
ENSMUSG00000022533	1941.326	1506.161	0.36617	0.000491	0.003678	Atp13a3
ENSMUSG00000022534	35.14934	14.56329	1.2712	0.001194	0.007772	Mefv
ENSMUSG00000022548	107.0788	47.04401	1.1866	7.91E-07	1.30E-05	Apod
ENSMUSG00000022582	2200.084	1201.923	0.87221	1.53E-10	5.29E-09	Ly6g
ENSMUSG00000022634	175.5112	118.5477	0.5661	0.001098	0.007244	Yaf2
ENSMUSG00000022636	811.7735	373.46	1.1201	2.40E-22	3.08E-20	Alcam
ENSMUSG00000022650	2395.376	1470.25	0.70419	1.09E-05	0.000133	Retnlb
ENSMUSG00000022651	259.291	2.396656	6.7574	2.11E-42	9.17E-40	Retnlg

ENSMUSG00000022674	392.1163	307.8895	0.34887	0.005186	0.026423	Ube2v2
ENSMUSG00000022685	302.5566	225.1122	0.42656	0.001613	0.010055	Parn
ENSMUSG00000022686	2152.085	789.0454	1.4476	2.15E-12	9.86E-11	B3gnt5
ENSMUSG00000022722	53.33415	27.52254	0.95445	0.001579	0.009864	Arl6
ENSMUSG00000022752	1212.059	1007.322	0.26694	0.008542	0.039952	Tomm70a
ENSMUSG00000022754	65.03931	40.14568	0.69607	0.010409	0.046979	Tmem45a
ENSMUSG00000022790	43.30521	19.54025	1.1481	0.000845	0.005814	Igsf11
ENSMUSG00000022797	9349.907	4008.622	1.2218	7.00E-22	8.49E-20	Tfrc
ENSMUSG00000022800	584.6643	438.9065	0.41369	0.000619	0.004499	Fyttd1
ENSMUSG00000022808	887.7156	611.1132	0.53866	9.06E-07	1.47E-05	Snx4
ENSMUSG00000022816	1252.063	735.3938	0.76772	1.93E-13	9.94E-12	Fstl1
ENSMUSG00000022824	36089.55	29725.52	0.27988	0.003644	0.019777	Muc13
ENSMUSG00000022828	179.5376	119.639	0.5856	0.000768	0.005379	Gtf2e1
ENSMUSG00000022848	999.6983	655.5734	0.60874	5.97E-09	1.55E-07	Dirc2
ENSMUSG00000022858	1667.563	1219.894	0.45099	4.14E-06	5.68E-05	Tra2b
ENSMUSG00000022860	102.0614	25.19579	2.0182	1.70E-14	1.01E-12	Chodl
ENSMUSG00000022863	59.92425	33.10854	0.85594	0.003118	0.017348	Btg3
ENSMUSG00000022876	53.6147	26.24828	1.0304	0.000843	0.005806	Samsn1
ENSMUSG00000022883	434.346	105.6454	2.0396	5.66E-15	3.54E-13	Robo1
ENSMUSG00000022886	21.64306	0.893977	4.5975	4.24E-08	9.26E-07	Prl2a1
ENSMUSG00000022892	4787.676	3726.587	0.36147	6.65E-05	0.000653	App
ENSMUSG00000022893	121.4266	57.80895	1.0707	2.19E-05	0.000245	Adamts1
ENSMUSG00000022895	2911.819	2032.658	0.51855	2.53E-07	4.71E-06	Ets2
ENSMUSG00000022912	266.108	188.4604	0.49775	0.002483	0.014386	Pros1
ENSMUSG00000022940	244.4471	175.8193	0.47543	0.001251	0.008083	Pigp
ENSMUSG00000022949	643.8835	473.4438	0.44361	0.000103	0.000955	Clic6
ENSMUSG00000022952	318.2819	172.6491	0.88246	0.000739	0.005208	Runx1
ENSMUSG00000022965	2044.514	1516.225	0.43127	1.08E-05	0.000132	Ifngr2
ENSMUSG00000022971	153.6667	106.3698	0.53072	0.00501	0.025678	Ifnar2
ENSMUSG00000022992	517.8125	415.5468	0.31742	0.008827	0.041066	Kansl2
ENSMUSG00000023008	151.7813	104.8152	0.53415	0.001812	0.011103	Fmnl3
ENSMUSG00000023021	356.6801	280.8306	0.34493	0.00976	0.044558	Cers5
ENSMUSG00000023031	1240.998	773.5773	0.68188	5.29E-11	1.94E-09	Cela1
ENSMUSG00000023034	184.3858	110.8079	0.73467	0.00556	0.027982	Nr4a1
ENSMUSG00000023039	2374.686	1081.823	1.1343	2.63E-06	3.81E-05	Krt7
ENSMUSG00000023043	4584.244	2738.918	0.74308	6.99E-10	2.15E-08	Krt18
ENSMUSG00000023074	218.1049	145.8733	0.58031	0.004771	0.024637	Mospd1
ENSMUSG00000023078	323.644	179.5069	0.85037	8.67E-05	0.000822	Cxcl13
ENSMUSG00000023122	268.444	193.5397	0.47199	0.010077	0.045797	Sult1c2
ENSMUSG00000023140	24.72937	4.939149	2.3239	2.64E-05	0.000289	Reg2
ENSMUSG00000023169	1024.885	814.5205	0.33144	0.000687	0.004898	Slc38a1
ENSMUSG00000023277	255.8192	192.6518	0.40913	0.005472	0.027611	Twf2
ENSMUSG00000023349	130.9204	68.86056	0.92694	6.30E-05	0.000623	Clec4n
ENSMUSG00000023433	13.27829	0	Inf	1.86E-05	0.000213	Cela3b
ENSMUSG00000023452	326.3805	229.9025	0.50553	0.008454	0.039695	Pisd
ENSMUSG00000023456	8753.595	7001.749	0.32216	0.000267	0.002178	Tpi1
ENSMUSG00000023885	398.8059	213.0034	0.90481	2.00E-07	3.77E-06	Thbs2
ENSMUSG00000023886	743.4865	495.6667	0.58494	0.002392	0.013971	Smoc2
ENSMUSG00000023903	19.3984	7.601704	1.3515	0.009189	0.042452	Mmp25
ENSMUSG00000023905	284.3728	109.8561	1.3722	3.45E-10	1.12E-08	Tnfrsf12a
ENSMUSG00000023906	9.467714	0.304351	4.9592	0.000314	0.002498	Cldn6
ENSMUSG00000023913	385.6895	151.7516	1.3457	9.63E-21	1.05E-18	Pla2g7

ENSMUSG00000023972	502.6872	219.7878	1.1935	1.86E-06	2.81E-05	Ptk7
ENSMUSG00000023992	43.61208	19.32485	1.1743	0.00067	0.004797	Trem2
ENSMUSG00000024011	90.81924	33.48242	1.4396	3.04E-08	6.80E-07	Pi16
ENSMUSG00000024029	5837.313	4284.98	0.44602	1.05E-05	0.000129	Tff3
ENSMUSG00000024032	8.155985	0.900336	3.1793	0.006369	0.031406	Tff1
ENSMUSG00000024053	86.26503	29.46269	1.5499	4.72E-09	1.27E-07	Emilin2
ENSMUSG00000024070	303.9591	234.5035	0.37427	0.008631	0.0403	Prkd3
ENSMUSG00000024095	768.2004	614.5516	0.32195	0.00495	0.025422	Hnrnpll
ENSMUSG00000024109	73.13045	20.54255	1.8319	2.83E-09	7.94E-08	Nrxn1
ENSMUSG00000024114	24.00505	7.221425	1.733	0.000504	0.003764	Prss41
ENSMUSG00000024140	1691.317	1219.867	0.47142	2.00E-06	2.99E-05	Epas1
ENSMUSG00000024143	479.2621	347.5797	0.46347	0.000181	0.00157	Rhoq
ENSMUSG00000024151	428.9612	327.3605	0.38997	0.009747	0.044524	Msh2
ENSMUSG00000024164	2199.756	1647.212	0.41732	0.000247	0.002048	C3
ENSMUSG00000024177	125.6753	50.80429	1.3067	3.33E-05	0.000357	Nme4
ENSMUSG00000024190	389.3124	262.0532	0.57107	1.22E-05	0.000148	Dusp1
ENSMUSG00000024225	70.74898	38.85908	0.86446	0.003091	0.017219	Clps
ENSMUSG00000024232	158.6063	38.57979	2.0395	2.82E-20	2.96E-18	Bambi
ENSMUSG00000024235	48.44683	25.70915	0.91412	0.004214	0.022259	Map3k8
ENSMUSG00000024245	15.09845	3.550512	2.0883	0.009447	0.043451	Tmem178
ENSMUSG00000024258	218.8165	165.148	0.40596	0.010512	0.047337	Polr2d
ENSMUSG00000024270	142.4172	73.16485	0.9609	1.60E-06	2.45E-05	Slc39a6
ENSMUSG00000024271	458.3504	260.0548	0.81764	3.24E-10	1.06E-08	Elp2
ENSMUSG00000024287	396.879	302.5135	0.3917	0.002526	0.014607	Thoc1
ENSMUSG00000024293	498.9673	371.2357	0.42661	0.00051	0.003805	Esco1
ENSMUSG00000024317	225.8766	141.9171	0.67049	2.15E-05	0.000241	Rnf138
ENSMUSG00000024349	646.1493	243.2026	1.4097	7.55E-09	1.91E-07	Tmem173
ENSMUSG00000024352	59.66988	26.83155	1.1531	9.69E-05	0.000907	Spata24
ENSMUSG00000024360	1801.211	1445.528	0.31737	0.001272	0.008195	Etf1
ENSMUSG00000024365	21.1992	3.398356	2.6411	0.002319	0.013631	Cyp21a1
ENSMUSG00000024399	101.0373	50.11966	1.0114	1.69E-05	0.000196	Ltb
ENSMUSG00000024401	130.2751	49.45373	1.3974	2.26E-09	6.44E-08	Tnf
ENSMUSG00000024421	1197.825	472.0308	1.3435	4.31E-36	1.15E-33	Lama3
ENSMUSG00000024424	57.54284	29.04463	0.98636	0.001007	0.006755	Ttc39c
ENSMUSG00000024427	124.8269	69.9819	0.83487	2.78E-05	0.000303	Spry4
ENSMUSG00000024471	53.14491	5.877299	3.1767	3.77E-12	1.69E-10	Myot
ENSMUSG00000024479	3024.271	2456.89	0.29975	0.00188	0.011431	Mal2
ENSMUSG00000024486	275.1072	148.7921	0.8867	7.83E-09	1.97E-07	Hbegf
ENSMUSG00000024493	947.8428	655.3534	0.53237	3.40E-07	6.08E-06	Lars
ENSMUSG00000024498	592.7619	481.8827	0.29877	0.007298	0.035161	Tcerg1
ENSMUSG00000024501	540.8179	293.4083	0.88223	7.01E-07	1.17E-05	Dpysl3
ENSMUSG00000024503	3390.382	2048.359	0.72698	0.001122	0.007374	Spink1
ENSMUSG00000024511	719.9485	500.9918	0.52311	0.000273	0.002218	Rab27b
ENSMUSG00000024512	6.544444	0	Inf	0.00078	0.005449	Dynap
ENSMUSG00000024521	428.976	265.5135	0.69211	8.32E-08	1.71E-06	Pmaip1
ENSMUSG00000024524	172.2757	99.63558	0.78999	7.30E-06	9.38E-05	Gnal
ENSMUSG00000024529	279.5037	91.45595	1.6117	3.27E-22	4.12E-20	Lox
ENSMUSG00000024538	194.0418	96.43257	1.0088	9.91E-09	2.44E-07	Ppic
ENSMUSG00000024544	108.9517	52.68055	1.0483	5.18E-06	6.91E-05	Ldlrad4
ENSMUSG00000024558	76.87622	31.54867	1.285	1.79E-06	2.71E-05	Mapk4
ENSMUSG00000024580	739.5645	526.5567	0.49009	8.82E-06	0.00011	Grpel2
ENSMUSG00000024583	938.9837	765.8261	0.29408	0.003874	0.020851	Txnl1

ENSMUSG00000024597	6385.943	4250.944	0.58712	1.28E-11	5.20E-10	Slc12a2
ENSMUSG00000024608	9989.299	6827.76	0.54897	0.000115	0.001057	Rps14
ENSMUSG00000024621	458.696	364.8306	0.33031	0.006647	0.032598	Csf1r
ENSMUSG00000024640	302.9477	219.3435	0.46588	0.001248	0.008068	Psat1
ENSMUSG00000024659	4866.176	2060.81	1.2396	1.95E-23	2.72E-21	Anxa1
ENSMUSG00000024673	128.0887	71.13648	0.84848	5.65E-05	0.000568	Ms4a1
ENSMUSG00000024677	116.4553	73.29835	0.66792	0.001381	0.008787	Ms4a6b
ENSMUSG00000024679	93.67069	53.71998	0.80214	0.000537	0.003984	Ms4a6d
ENSMUSG00000024735	1147.496	936.3383	0.29339	0.00422	0.022276	Prpf19
ENSMUSG00000024736	117.891	54.67115	1.1086	0.000166	0.001459	Tmem132a
ENSMUSG00000024737	108.3848	56.32603	0.94429	0.001553	0.00973	Slc15a3
ENSMUSG00000024750	1224.293	949.8112	0.36624	0.004032	0.021474	Zfand5
ENSMUSG00000024759	1387.467	939.582	0.56236	1.20E-08	2.93E-07	Atl3
ENSMUSG00000024771	19.28091	6.739521	1.5165	0.004206	0.022221	Lipk
ENSMUSG00000024774	142.8347	64.28261	1.1518	2.26E-08	5.19E-07	Ankrd22
ENSMUSG00000024789	1162.862	947.7279	0.29513	0.005287	0.026844	Jak2
ENSMUSG00000024810	203.3268	85.55465	1.2489	4.59E-12	2.03E-10	Il33
ENSMUSG00000024811	1971.004	1401.675	0.49178	1.91E-07	3.62E-06	Tnks2
ENSMUSG00000024905	8.436565	1.451848	2.5388	0.009373	0.043178	Mtl5
ENSMUSG00000024912	29.19217	11.31713	1.3671	0.001103	0.007261	Fosl1
ENSMUSG00000024913	1022.13	753.9879	0.43897	3.32E-05	0.000356	Lrp5
ENSMUSG00000024924	391.529	209.4215	0.90271	3.18E-11	1.21E-09	Vldlr
ENSMUSG00000024968	45.81104	13.16863	1.7986	1.43E-06	2.22E-05	Rcor2
ENSMUSG00000024986	42.0918	18.25964	1.2049	0.000602	0.004394	Hhex
ENSMUSG00000025017	99.7519	66.22976	0.59086	0.007186	0.034726	Pik3ap1
ENSMUSG00000025026	1003.07	747.8908	0.42352	3.17E-05	0.000341	Add3
ENSMUSG00000025040	333.3536	226.9206	0.55487	8.53E-05	0.000813	Fundc1
ENSMUSG00000025041	325.3288	218.3997	0.57493	3.62E-05	0.000384	Nt5c2
ENSMUSG00000025044	140.9687	75.377	0.90318	0.000131	0.001183	Msr1
ENSMUSG00000025059	1359.092	749.6084	0.85843	8.94E-07	1.45E-05	Gk
ENSMUSG00000025064	19.95955	6.834826	1.5461	0.0051	0.026059	Col17a1
ENSMUSG00000025075	91.2503	19.62284	2.2173	2.89E-07	5.26E-06	Habp2
ENSMUSG00000025082	48.82554	14.24622	1.7771	3.86E-07	6.80E-06	Vwa2
ENSMUSG00000025091	213.3954	34.78915	2.6168	6.79E-07	1.14E-05	Pnliprp2
ENSMUSG00000025103	1041.722	814.0495	0.35578	0.000595	0.004353	Btbd1
ENSMUSG00000025142	725.9681	515.2096	0.49475	0.007299	0.035161	Aspscr1
ENSMUSG00000025150	406.1001	255.7168	0.66729	1.54E-07	2.98E-06	Cbr2
ENSMUSG00000025151	1030.247	859.2175	0.26189	0.007118	0.034474	Maged1
ENSMUSG00000025161	913.0895	238.6144	1.9361	3.75E-10	1.21E-08	Slc16a3
ENSMUSG00000025171	107.8099	65.30433	0.72324	0.001499	0.00945	Ubtd1
ENSMUSG00000025176	185.1493	48.32534	1.9378	2.19E-21	2.57E-19	Hoga1
ENSMUSG00000025223	796.7296	521.239	0.61214	0.000404	0.003118	Ldb1
ENSMUSG00000025268	228.8136	140.1361	0.70734	4.00E-06	5.51E-05	Maged2
ENSMUSG00000025283	2944.687	1819.728	0.69439	6.41E-14	3.45E-12	Sat1
ENSMUSG00000025287	376.3853	197.6936	0.92894	5.90E-11	2.15E-09	Acot9
ENSMUSG00000025289	531.9508	339.2813	0.64881	1.06E-07	2.10E-06	Prdx4
ENSMUSG00000025290	8138.707	6068.01	0.42358	8.24E-07	1.35E-05	Rps24
ENSMUSG00000025314	1351.344	1123.428	0.26649	0.005001	0.025659	Ptprj
ENSMUSG00000025321	87.75229	38.1042	1.2035	3.04E-06	4.32E-05	Itgb8
ENSMUSG00000025324	162.5799	97.24494	0.74145	4.43E-05	0.000458	Atp10a
ENSMUSG00000025330	98.3364	24.62486	1.9976	0.001404	0.008908	Padi4
ENSMUSG00000025352	174.5725	50.47416	1.7902	1.99E-05	0.000226	Gdf11

ENSMUSG00000025362	4025.998	3359.992	0.26089	0.003571	0.019433	Rps26
ENSMUSG00000025372	305.8461	225.0192	0.44276	0.001172	0.007657	Baiap2
ENSMUSG00000025383	66.98392	4.539494	3.8832	1.08E-08	2.64E-07	Il23a
ENSMUSG00000025403	391.6602	312.7286	0.32469	0.010357	0.046795	Shmt2
ENSMUSG00000025432	369.5738	100.162	1.8835	0.002125	0.01269	Avil
ENSMUSG00000025473	236.7936	64.91099	1.8671	7.63E-05	0.000735	Adam8
ENSMUSG00000025481	249.764	131.4502	0.92605	4.38E-09	1.18E-07	Urah
ENSMUSG00000025487	1364.918	1133.899	0.26752	0.005956	0.029636	Psmd13
ENSMUSG00000025491	577.2149	192.7592	1.5823	2.07E-06	3.08E-05	Ifitm1
ENSMUSG00000025492	1692.367	908.1905	0.89798	2.00E-05	0.000226	Ifitm3
ENSMUSG00000025510	1788.853	1331.502	0.42598	1.11E-05	0.000135	Cd151
ENSMUSG00000025531	314.36	239.3968	0.39301	0.003693	0.019983	Chm
ENSMUSG00000025532	277.7432	200.1401	0.47274	0.001307	0.008383	Crcp
ENSMUSG00000025534	780.2214	548.9169	0.5073	0.000325	0.002584	Gusb
ENSMUSG00000025555	568.5742	455.3958	0.32023	0.007079	0.034314	Farp1
ENSMUSG00000025582	20.49436	5.737822	1.8367	0.001356	0.008653	Nptx1
ENSMUSG00000025591	141.7843	92.81381	0.61129	0.002564	0.014785	Tma16
ENSMUSG00000025592	21.87787	5.192669	2.0749	0.001438	0.009115	Dach2
ENSMUSG00000025613	2242.736	1795.395	0.32096	0.000452	0.003438	Cct8
ENSMUSG00000025630	1209.311	928.9264	0.38055	0.000234	0.001955	Hprt
ENSMUSG00000025650	62.35814	28.46738	1.1313	6.94E-05	0.000676	Col7a1
ENSMUSG00000025746	7.947327	0.906695	3.1318	0.005548	0.02794	Il6
ENSMUSG00000025804	165.4895	83.82313	0.98132	1.81E-07	3.46E-06	Ccr1
ENSMUSG00000025809	5969.79	4087.651	0.54641	9.65E-10	2.89E-08	Itgb1
ENSMUSG00000025856	836.8378	653.9253	0.35582	0.000849	0.005843	Pdgfa
ENSMUSG00000025867	237.7202	129.9865	0.8709	3.98E-07	7.00E-06	Cplx2
ENSMUSG00000025902	237.2442	57.08056	2.0553	8.66E-05	0.000821	Sox17
ENSMUSG00000025917	954.2966	663.5927	0.52414	8.80E-07	1.43E-05	Cops5
ENSMUSG00000025921	919.1024	374.3992	1.2956	1.17E-30	2.35E-28	Rdh10
ENSMUSG00000025929	43.05099	4.108383	3.3894	7.54E-13	3.60E-11	Il17a
ENSMUSG00000025931	206.3223	146.2752	0.49621	0.004072	0.021605	Paqr8
ENSMUSG00000025934	367.9222	275.011	0.41991	0.002344	0.013738	Gsta3
ENSMUSG00000025938	115.0851	22.29811	2.3677	1.57E-05	0.000183	Slco5a1
ENSMUSG00000025939	290.4005	208.3492	0.47904	0.000754	0.005291	Ube2w
ENSMUSG00000025950	3180.492	2083.469	0.61026	2.39E-11	9.30E-10	Idh1
ENSMUSG00000025967	4719.973	3725.466	0.34136	0.000119	0.001087	Eef1b2
ENSMUSG00000025979	780.3425	593.2083	0.39557	0.002108	0.012605	Mob4
ENSMUSG00000025981	399.4573	287.3428	0.47527	0.000334	0.002643	Coq10b
ENSMUSG00000025995	359.6557	265.5441	0.43766	0.000831	0.00574	Wdr75
ENSMUSG00000025997	155.7027	85.9981	0.85642	4.51E-06	6.12E-05	Ikzf2
ENSMUSG00000026003	2166.688	1640.711	0.40117	2.28E-05	0.000254	Acadl
ENSMUSG00000026004	138.5418	96.68677	0.51893	0.004865	0.02505	Kansl1l
ENSMUSG00000026009	70.57918	29.39282	1.2638	5.35E-06	7.13E-05	Icos
ENSMUSG00000026011	34.26199	17.5051	0.96883	0.010564	0.047537	Ctla4
ENSMUSG00000026020	967.0727	722.5921	0.42044	4.34E-05	0.000451	Nop58
ENSMUSG00000026042	1112.178	711.4015	0.64465	5.88E-10	1.83E-08	Col5a2
ENSMUSG00000026043	6233.975	4757.302	0.39001	9.70E-06	0.00012	Col3a1
ENSMUSG00000026065	36.74149	9.770276	1.9109	5.98E-06	7.88E-05	Slc9a4
ENSMUSG00000026068	76.28877	22.61548	1.7542	1.26E-09	3.74E-08	Il18rap
ENSMUSG00000026069	378.505	93.51082	2.0171	6.31E-38	1.91E-35	Il1rl1
ENSMUSG00000026070	50.97203	24.59311	1.0515	0.000582	0.004279	Il18r1
ENSMUSG00000026072	361.2921	245.1097	0.55974	0.001008	0.006758	Il1r1

ENSMUSG00000026073	89.25327	7.709726	3.5332	2.35E-24	3.46E-22	Il1r2
ENSMUSG00000026074	933.7262	449.1394	1.0558	1.48E-07	2.88E-06	Map4k4
ENSMUSG00000026097	197.833	145.982	0.43849	0.007886	0.037492	Ormdl1
ENSMUSG00000026103	1024.95	786.1548	0.38267	0.000176	0.001535	Gls
ENSMUSG00000026107	226.4904	172.3125	0.39442	0.010422	0.047029	Nabp1
ENSMUSG00000026117	56.14679	26.78741	1.0676	0.007064	0.034275	Zap70
ENSMUSG00000026121	280.8358	108.056	1.3779	2.99E-10	9.80E-09	Sema4c
ENSMUSG00000026134	173.3264	115.3514	0.58746	0.001193	0.007764	Prim2
ENSMUSG00000026159	755.2444	593.1609	0.34852	0.001299	0.008337	Agfg1
ENSMUSG00000026166	62.27938	28.54929	1.1253	0.000119	0.001089	Ccl20
ENSMUSG00000026167	75.72729	5.617421	3.7528	2.63E-06	3.81E-05	Wnt10a
ENSMUSG00000026180	53.30782	13.78369	1.9514	6.23E-08	1.32E-06	Cxcr2
ENSMUSG00000026181	162.9117	100.5998	0.69546	0.00029	0.002342	Ppm1f
ENSMUSG00000026185	1480.21	872.5944	0.76242	3.92E-14	2.23E-12	Igfbp5
ENSMUSG00000026187	417.1771	250.4992	0.73585	1.30E-06	2.03E-05	Xrcc5
ENSMUSG00000026193	6450.9	3108.401	1.0533	3.44E-32	7.57E-30	Fn1
ENSMUSG00000026220	13.65003	0	Inf	2.11E-07	3.95E-06	Slc16a14
ENSMUSG00000026223	1215.938	766.7436	0.66525	2.63E-09	7.42E-08	Itm2c
ENSMUSG00000026234	5998.289	4160.384	0.52783	1.58E-09	4.63E-08	Ncl
ENSMUSG00000026235	231.2462	169.0279	0.45217	0.004009	0.021382	Epha4
ENSMUSG00000026238	13733.42	11142.91	0.30156	0.000408	0.003144	Ptma
ENSMUSG00000026249	215.5743	127.3049	0.7599	8.33E-06	0.000105	Serpine2
ENSMUSG00000026276	1968.972	1505.639	0.38707	4.66E-05	0.000479	2-Sep
ENSMUSG00000026278	391.1058	196.3616	0.99405	3.45E-08	7.66E-07	Bok
ENSMUSG00000026305	1855.183	1295.963	0.51754	7.69E-08	1.59E-06	Lrrfip1
ENSMUSG00000026327	97.43553	2.796012	5.123	4.73E-37	1.38E-34	Serpinb11
ENSMUSG00000026360	297.9501	208.362	0.51598	0.000246	0.002041	Rgs2
ENSMUSG00000026365	2404.748	374.6804	2.6822	2.18E-26	3.59E-24	Cfh
ENSMUSG00000026377	427.4816	308.9182	0.46864	0.000259	0.002118	Nifk
ENSMUSG00000026383	288.1044	186.927	0.62412	0.000772	0.005404	Epb4.1l5
ENSMUSG00000026390	10.65494	1.147497	3.215	0.000906	0.006168	Marco
ENSMUSG00000026393	939.4317	660.6357	0.50793	1.98E-05	0.000224	Nek7
ENSMUSG00000026395	565.6119	454.4997	0.31553	0.006975	0.033958	Ptprc
ENSMUSG00000026399	771.9733	328.4768	1.2328	1.13E-12	5.32E-11	Cd55
ENSMUSG00000026405	148.2251	68.29599	1.1179	9.22E-09	2.30E-07	C4bp
ENSMUSG00000026411	325.4793	163.4051	0.99411	5.32E-12	2.32E-10	Tmem9
ENSMUSG00000026413	651.9227	412.2249	0.66127	6.30E-06	8.23E-05	Pkp1
ENSMUSG00000026414	89.26013	52.29357	0.77138	0.000643	0.004646	Tnnt2
ENSMUSG00000026435	710.3008	361.5667	0.97417	5.03E-17	3.99E-15	Slc45a3
ENSMUSG00000026450	24.1682	4.742481	2.3494	2.37E-05	0.000262	Chit1
ENSMUSG00000026475	93.97729	44.81223	1.0684	8.68E-06	0.000109	Rgs16
ENSMUSG00000026478	915.3894	716.7968	0.35282	0.000669	0.004795	Lamc1
ENSMUSG00000026479	2054.892	694.5975	1.5648	1.99E-54	1.22E-51	Lamc2
ENSMUSG00000026480	532.838	350.2248	0.60542	8.19E-07	1.34E-05	Ncf2
ENSMUSG00000026484	353.0585	237.571	0.57155	2.54E-05	0.00028	Rnf2
ENSMUSG00000026509	1551.405	1015.592	0.61125	4.39E-10	1.40E-08	Capn2
ENSMUSG00000026511	1195.091	929.0017	0.36337	0.000382	0.002969	Srp9
ENSMUSG00000026548	46.34594	24.65695	0.91045	0.005923	0.029479	Slamf9
ENSMUSG00000026556	140.1932	45.31359	1.6294	0.000195	0.00168	Vangl2
ENSMUSG00000026566	809.002	527.9386	0.61577	1.70E-08	4.00E-07	Mpzl1
ENSMUSG00000026579	30.94759	7.1201	2.1199	5.41E-06	7.20E-05	F5
ENSMUSG00000026580	73.59368	17.91713	2.0382	3.36E-11	1.27E-09	Selp

ENSMUSG00000026581	111.3926	23.52853	2.2432	9.29E-18	7.93E-16	Sell
ENSMUSG00000026620	96.90734	45.1106	1.1031	0.005825	0.029071	Mark1
ENSMUSG00000026627	79.83805	51.13972	0.64263	0.007121	0.034478	Tmem206
ENSMUSG00000026639	1736.655	1272.756	0.44836	1.33E-05	0.000158	Lamb3
ENSMUSG00000026656	286.4672	210.5509	0.4442	0.001697	0.010515	Fcgr2b
ENSMUSG00000026676	269.9915	168.3373	0.68156	0.000202	0.001725	Ccdc3
ENSMUSG00000026721	846.7611	701.2201	0.27209	0.008366	0.03932	Rabgap1l
ENSMUSG00000026723	83.90295	50.27719	0.73882	0.001557	0.009756	Trdmt1
ENSMUSG00000026727	448.1183	321.4202	0.47942	0.000292	0.002358	Rsu1
ENSMUSG00000026728	2200.381	1253.91	0.81132	3.99E-10	1.28E-08	Vim
ENSMUSG00000026730	169.0197	120.2778	0.49082	0.00391	0.020976	Pter
ENSMUSG00000026739	360.4447	229.6273	0.65048	1.72E-06	2.63E-05	Bmi1
ENSMUSG00000026773	612.2852	188.0943	1.7027	2.24E-39	7.98E-37	Pfkfb3
ENSMUSG00000026796	1562.615	987.5046	0.6621	9.21E-12	3.85E-10	Fam129b
ENSMUSG00000026797	801.525	307.1223	1.3839	2.01E-06	3.01E-05	Stxbp1
ENSMUSG00000026818	16.68385	0	Inf	5.24E-08	1.13E-06	Cel
ENSMUSG00000026822	935.0104	121.3056	2.9463	1.85E-11	7.35E-10	Lcn2
ENSMUSG00000026828	447.5119	345.4368	0.3735	0.0058	0.028981	Galnt5
ENSMUSG00000026832	248.7787	145.0312	0.7785	8.67E-07	1.41E-05	Cytip
ENSMUSG00000026836	215.4634	162.9737	0.4028	0.008124	0.038407	Acvr1
ENSMUSG00000026843	595.4907	419.5797	0.50513	1.02E-05	0.000126	Fubp3
ENSMUSG00000026880	2773.575	1792.331	0.62991	4.72E-07	8.18E-06	Stom
ENSMUSG00000026921	111.8623	74.65591	0.5834	0.00398	0.021277	Egfl7
ENSMUSG00000026939	108.932	72.62012	0.58499	0.005138	0.026228	Tmem141
ENSMUSG00000026950	36.06277	14.62048	1.3025	0.000782	0.005456	Neb
ENSMUSG00000026961	530.8285	361.0666	0.55598	6.31E-06	8.23E-05	Lrrc26
ENSMUSG00000026970	845.0645	428.1693	0.98088	4.66E-11	1.72E-09	Rbms1
ENSMUSG00000026971	496.6196	327.9962	0.59846	1.42E-06	2.20E-05	Itgb6
ENSMUSG00000026974	284.8222	199.6712	0.51244	0.000519	0.003865	Zmynd19
ENSMUSG00000026981	955.999	390.811	1.2905	2.71E-31	5.76E-29	Il1rn
ENSMUSG00000026994	1193.625	771.3117	0.62996	2.20E-05	0.000245	Galnt3
ENSMUSG00000027010	384.4169	296.4222	0.37502	0.007012	0.034078	Slc25a12
ENSMUSG00000027012	1457.048	1182.519	0.30119	0.001873	0.011398	Dync1i2
ENSMUSG00000027014	288.9452	221.4803	0.38362	0.01044	0.047062	Cwc22
ENSMUSG00000027018	371.3416	282.5926	0.39402	0.002421	0.014089	Hat1
ENSMUSG00000027068	1080.46	465.6333	1.2144	1.86E-28	3.25E-26	Dhrs9
ENSMUSG00000027087	953.5556	556.2346	0.77762	1.27E-13	6.61E-12	Itgav
ENSMUSG00000027111	3520.103	2758.46	0.35175	8.39E-05	0.000801	Itga6
ENSMUSG00000027134	471.8645	236.242	0.99811	6.38E-09	1.64E-07	Lpcat4
ENSMUSG00000027170	1138.956	834.8654	0.4481	1.22E-05	0.000148	Eif3m
ENSMUSG00000027189	581.527	465.8919	0.31985	0.003304	0.018203	Trim44
ENSMUSG00000027195	1773.385	1310.05	0.43688	4.60E-06	6.22E-05	Hsd17b12
ENSMUSG00000027204	538.4428	421.0552	0.35478	0.002408	0.014027	Fbn1
ENSMUSG00000027225	1225.868	858.973	0.51312	9.97E-06	0.000123	Duoxa2
ENSMUSG00000027238	42.69842	13.84054	1.6253	1.45E-05	0.00017	Frmd5
ENSMUSG00000027257	153.1314	57.27719	1.4187	8.18E-06	0.000104	Pacsin3
ENSMUSG00000027298	186.3559	125.9853	0.5648	0.000981	0.006608	Tyro3
ENSMUSG00000027314	199.5949	149.5709	0.41625	0.009365	0.043153	Dll4
ENSMUSG00000027333	793.0179	403.0201	0.9765	1.36E-15	9.13E-14	Smox
ENSMUSG00000027340	753.418	531.4852	0.50342	4.63E-06	6.26E-05	Slc23a2
ENSMUSG00000027342	772.2681	622.4384	0.31117	0.00307	0.017131	Pcna
ENSMUSG00000027346	1023.642	510.7606	1.003	1.60E-16	1.17E-14	Gpcpd1

ENSMUSG00000027351	468.763	358.5526	0.38667	0.002674	0.015301	Spred1
ENSMUSG00000027356	1433.13	1087.07	0.39873	0.000138	0.001245	Fermt1
ENSMUSG00000027358	393.1024	279.1462	0.49388	0.00025	0.002066	Bmp2
ENSMUSG00000027360	77.10404	15.71748	2.2944	3.26E-09	8.99E-08	Hdc
ENSMUSG00000027366	4206.566	3271.428	0.36272	0.00546	0.027563	Sppl2a
ENSMUSG00000027376	17.50619	3.987982	2.1341	0.000648	0.004676	Prom2
ENSMUSG00000027378	124.0763	74.45187	0.73685	0.000206	0.001756	Nphp1
ENSMUSG00000027381	247.4033	158.9794	0.63802	0.002331	0.013684	Bcl2l11
ENSMUSG00000027398	387.758	60.72506	2.6748	5.77E-18	5.10E-16	Il1b
ENSMUSG00000027399	67.10143	8.577968	2.9676	1.82E-07	3.47E-06	Il1a
ENSMUSG00000027405	981.1869	722.4163	0.4417	1.40E-05	0.000166	Nop56
ENSMUSG00000027427	235.6249	180.9414	0.38097	0.011261	0.049969	Polr3f
ENSMUSG00000027433	1001.484	728.652	0.45884	8.00E-06	0.000102	Xrn2
ENSMUSG00000027435	253.3524	166.8407	0.60267	9.98E-05	0.000932	Cd93
ENSMUSG00000027447	1826.9	1508.512	0.27627	0.003308	0.018219	Cst3
ENSMUSG00000027454	94.56471	64.49196	0.55218	0.008813	0.041029	Gins1
ENSMUSG00000027455	927.978	692.0539	0.42321	3.57E-05	0.00038	Nsfl1c
ENSMUSG00000027460	36.64345	14.84221	1.3038	0.000946	0.006413	Angpt4
ENSMUSG00000027495	503.4241	371.9075	0.43683	0.00112	0.007368	Fam210b
ENSMUSG00000027533	194.6623	104.3787	0.89915	3.51E-07	6.25E-06	Fabp5
ENSMUSG00000027540	663.1327	516.5368	0.36043	0.00073	0.005162	Ptpn1
ENSMUSG00000027544	155.057	102.4694	0.59761	0.001389	0.008829	Nfatc2
ENSMUSG00000027547	6.498836	0.595985	3.4468	0.00822	0.038778	Sall4
ENSMUSG00000027566	1745.332	1374.702	0.34438	0.000246	0.002039	Psma7
ENSMUSG00000027570	59.43473	18.86232	1.6558	0.000139	0.001248	Col9a3
ENSMUSG00000027656	33.68156	9.041173	1.8974	0.000202	0.001727	Wisp2
ENSMUSG00000027660	1331.439	907.556	0.55293	1.31E-08	3.17E-07	Skil
ENSMUSG00000027680	599.992	474.7313	0.33783	0.002606	0.014979	Fxr1
ENSMUSG00000027692	392.8878	298.6285	0.39577	0.001626	0.010122	Tnik
ENSMUSG00000027714	279.2949	203.5385	0.45649	0.002925	0.016466	Exosc9
ENSMUSG00000027737	450.9052	102.4058	2.1385	2.42E-14	1.40E-12	Slc7a11
ENSMUSG00000027750	2386.657	1614.556	0.56385	0.002465	0.014291	Postn
ENSMUSG00000027787	369.4093	238.7568	0.62968	4.92E-06	6.60E-05	Nmd3
ENSMUSG00000027799	189.54	124.1411	0.61052	0.000444	0.003383	Nbea
ENSMUSG00000027800	211.8354	137.8652	0.61968	6.05E-05	0.000602	Tm4sf1
ENSMUSG00000027803	293.3114	186.3234	0.65462	2.29E-06	3.36E-05	Wwtr1
ENSMUSG00000027805	84.81645	49.66243	0.77219	0.001184	0.007723	Pfn2
ENSMUSG00000027810	853.2659	690.074	0.30624	0.003833	0.020662	Eif2a
ENSMUSG00000027823	665.1214	519.9287	0.3553	0.001419	0.008995	Gmps
ENSMUSG00000027852	851.8839	603.3609	0.49764	2.85E-06	4.08E-05	Nras
ENSMUSG00000027864	2391.193	1960.546	0.28647	0.002057	0.012372	Ptgfrn
ENSMUSG00000027876	2542.871	1259.837	1.0132	6.78E-27	1.14E-24	Reg4
ENSMUSG00000027878	360.4199	254.9956	0.49921	0.000149	0.001321	Notch2
ENSMUSG00000027907	3868.589	1682.887	1.2009	3.04E-36	8.34E-34	S100a11
ENSMUSG00000027963	202.0682	134.7344	0.58472	0.000355	0.002787	Extl2
ENSMUSG00000027978	66.70341	10.77833	2.6296	1.17E-08	2.84E-07	Prss12
ENSMUSG00000027985	86.85219	21.37268	2.0228	4.09E-05	0.000428	Lef1
ENSMUSG00000027994	104.7235	69.88762	0.58348	0.008314	0.039118	Ccdc109b
ENSMUSG00000027996	93.81433	22.45663	2.0627	2.80E-14	1.61E-12	Sfrp2
ENSMUSG00000027997	516.4798	370.4584	0.4794	0.002282	0.013456	Casp6
ENSMUSG00000027998	483.9791	363.165	0.41432	0.001156	0.007571	Plrg1
ENSMUSG00000027999	222.7388	114.3952	0.96133	0.00029	0.00234	Pla2g12a

ENSMUSG00000028016	276.9137	202.8595	0.44896	0.002765	0.015715	Ints12
ENSMUSG00000028029	700.8267	516.4119	0.44054	7.76E-05	0.000747	Aimp1
ENSMUSG00000028031	333.0028	58.55781	2.5076	1.71E-05	0.000197	Dkk2
ENSMUSG00000028040	129.9032	69.29167	0.90668	3.98E-05	0.000418	Efna4
ENSMUSG00000028073	220.2265	114.8012	0.93985	1.53E-08	3.63E-07	Pear1
ENSMUSG00000028081	12550.02	9090.502	0.46526	7.18E-08	1.50E-06	Rps3a1
ENSMUSG00000028108	621.6858	443.7791	0.48634	2.79E-05	0.000304	Ecm1
ENSMUSG00000028111	106.7139	52.41427	1.0257	7.50E-06	9.62E-05	Ctsk
ENSMUSG00000028128	893.3477	364.3665	1.2938	5.93E-09	1.55E-07	F3
ENSMUSG00000028152	293.3446	185.1789	0.66368	6.28E-06	8.21E-05	Tspan5
ENSMUSG00000028156	898.5737	741.5551	0.27708	0.008497	0.039828	Eif4e
ENSMUSG00000028159	174.0435	120.609	0.52911	0.002388	0.013954	Dapp1
ENSMUSG00000028186	50.33902	19.965	1.3342	5.24E-05	0.000532	Uox
ENSMUSG00000028199	260.6153	155.0298	0.74938	9.67E-07	1.55E-05	Cryz
ENSMUSG00000028214	107.5294	50.79793	1.0819	3.78E-06	5.25E-05	Gem
ENSMUSG00000028218	140.1534	95.41849	0.55467	0.00276	0.015699	Fam92a
ENSMUSG00000028234	6816.157	5184.883	0.39465	0.001362	0.008685	Rps20
ENSMUSG00000028249	3690.928	2544.307	0.53671	3.57E-09	9.77E-08	Sdcbp
ENSMUSG00000028262	279.1914	121.3946	1.2016	1.68E-14	9.98E-13	Clca3a2
ENSMUSG00000028266	432.0548	297.1193	0.54017	2.88E-05	0.000312	Lmo4
ENSMUSG00000028271	392.9921	306.0664	0.36066	0.007425	0.035614	Gtf2b
ENSMUSG00000028312	774.6218	628.3435	0.30194	0.003881	0.020876	Smc2
ENSMUSG00000028329	134.014	95.81289	0.48409	0.010073	0.045789	Xpa
ENSMUSG00000028345	246.0836	165.8457	0.56931	0.000337	0.002665	Tex10
ENSMUSG00000028347	330.6857	95.62194	1.79	2.54E-30	4.87E-28	Tmeff1
ENSMUSG00000028348	12.73641	2.472585	2.3649	0.002094	0.012552	Murc
ENSMUSG00000028358	256.433	168.8888	0.60251	4.41E-05	0.000457	Zfp618
ENSMUSG00000028360	46.67903	10.16963	2.1985	8.54E-05	0.000813	Slc44a5
ENSMUSG00000028364	1285.36	1024.829	0.32679	0.001176	0.007677	Tnc
ENSMUSG00000028370	148.0552	55.78761	1.4081	1.93E-11	7.58E-10	Pappa
ENSMUSG00000028378	563.0612	371.3726	0.60042	5.30E-05	0.000537	Ptgr1
ENSMUSG00000028382	3672.311	3046.216	0.26967	0.002371	0.013869	Ptbp3
ENSMUSG00000028409	900.2851	707.2062	0.34825	0.000757	0.005309	Smu1
ENSMUSG00000028410	2275.015	1911.505	0.25117	0.006446	0.031731	Dnaja1
ENSMUSG00000028413	2548.333	1525.456	0.74031	1.28E-15	8.63E-14	B4galt1
ENSMUSG00000028415	9090.107	4983.156	0.86724	4.61E-23	6.23E-21	Spink4
ENSMUSG00000028416	2405.416	1889.899	0.34798	0.000219	0.00185	Bag1
ENSMUSG00000028431	257.6726	190.1795	0.43818	0.0027	0.015421	Ikbkap
ENSMUSG00000028456	566.3826	402.1876	0.49391	1.97E-05	0.000223	Unc13b
ENSMUSG00000028494	1113.164	906.7011	0.29597	0.004032	0.021474	Plin2
ENSMUSG00000028495	9753.747	7494.348	0.38015	1.37E-05	0.000161	Rps6
ENSMUSG00000028496	762.648	493.0603	0.62925	2.10E-08	4.86E-07	Mllt3
ENSMUSG00000028524	273.9129	207.3421	0.4017	0.004929	0.025323	Sgip1
ENSMUSG00000028525	222.1574	147.3128	0.5927	0.000178	0.001548	Pde4b
ENSMUSG00000028527	1239.502	970.0367	0.35365	0.010116	0.045951	Ak4
ENSMUSG00000028528	170.2657	16.19273	3.3944	2.61E-11	1.01E-09	Dnajc6
ENSMUSG00000028542	146.3523	95.81819	0.61107	0.000673	0.004812	Slc6a9
ENSMUSG00000028559	928.1834	743.1774	0.3207	0.002248	0.013278	Osbpl9
ENSMUSG00000028568	674.5313	456.1795	0.56428	5.70E-07	9.68E-06	Btf3l4
ENSMUSG00000028576	154.1227	90.85466	0.76245	9.28E-05	0.000875	Ift74
ENSMUSG00000028577	890.7891	599.4399	0.57147	4.07E-07	7.13E-06	Plaa
ENSMUSG00000028581	506.7059	395.3542	0.358	0.003455	0.018896	Laptm5

ENSMUSG00000028599	1013.966	550.7315	0.88059	3.13E-16	2.22E-14	Tnfrsf1b
ENSMUSG00000028601	214.609	120.1779	0.83654	0.006712	0.032883	Echdc2
ENSMUSG00000028602	197.6506	119.506	0.72587	1.89E-05	0.000216	Tnfrsf8
ENSMUSG00000028609	504.1472	384.8202	0.38966	0.001192	0.007763	Magoh
ENSMUSG00000028613	166.8728	103.6745	0.68669	9.39E-05	0.000884	Lrp8
ENSMUSG00000028626	65.48984	16.22448	2.0131	0.00019	0.001638	Col9a2
ENSMUSG00000028633	480.1366	316.7515	0.60009	0.000118	0.001079	Ctps
ENSMUSG00000028643	291.2632	213.5945	0.44745	0.001159	0.007587	Ccdc23
ENSMUSG00000028645	1895.141	1201.43	0.65755	3.19E-11	1.21E-09	Slc2a1
ENSMUSG00000028654	433.1064	184.0858	1.2343	2.45E-11	9.50E-10	Mycl
ENSMUSG00000028655	264.7591	101.1701	1.3879	1.14E-17	9.65E-16	Mfsd2a
ENSMUSG00000028680	196.7896	143.6619	0.45398	0.004805	0.024765	Plk3
ENSMUSG00000028683	162.8206	115.6126	0.49398	0.006574	0.032277	Eif2b3
ENSMUSG00000028691	6070.744	4737.135	0.35786	5.47E-05	0.000553	Prdx1
ENSMUSG00000028698	596.9183	477.215	0.32289	0.003612	0.019641	Pik3r3
ENSMUSG00000028749	1104.324	519.1584	1.0889	3.55E-18	3.20E-16	Pla2g2f
ENSMUSG00000028751	14.65487	2.238142	2.711	0.00034	0.002685	Pla2g2e
ENSMUSG00000028771	592.0186	425.1061	0.47782	2.34E-05	0.000259	Ptpn12
ENSMUSG00000028780	1926.832	1135.691	0.76266	1.09E-11	4.50E-10	Sema3c
ENSMUSG00000028788	5827.257	4910.503	0.24695	0.006872	0.033535	Ptp4a2
ENSMUSG00000028798	1621.189	1242.902	0.38334	7.12E-05	0.00069	Eif3i
ENSMUSG00000028799	194.7147	147.6362	0.39932	0.007513	0.036012	Zfp362
ENSMUSG00000028822	720.3812	566.0769	0.34776	0.003014	0.016899	Tmem50a
ENSMUSG00000028826	647.5695	483.7221	0.42086	0.000317	0.002527	Tmem57
ENSMUSG00000028832	1879.736	1317.93	0.51226	0.002104	0.012592	Stmn1
ENSMUSG00000028836	366.9447	128.0168	1.5192	1.63E-08	3.87E-07	Slc30a2
ENSMUSG00000028838	111.9728	66.07723	0.76092	0.000377	0.002937	Extl1
ENSMUSG00000028840	127.9131	87.16463	0.55335	0.004504	0.023529	Zfp593
ENSMUSG00000028859	78.68949	31.16801	1.3361	8.28E-07	1.36E-05	Csf3r
ENSMUSG00000028864	117.1864	39.18849	1.5803	8.67E-12	3.66E-10	Hgf
ENSMUSG00000028869	414.275	316.4802	0.38847	0.001462	0.009245	Gnl2
ENSMUSG00000028874	84.97962	49.00324	0.79424	0.001049	0.006988	Fgr
ENSMUSG00000028889	224.4545	163.3857	0.45814	0.003588	0.019518	Yrdc
ENSMUSG00000028936	3539.332	2679.137	0.40171	6.85E-06	8.84E-05	Rpl22
ENSMUSG00000028953	591.9081	451.7922	0.38971	0.000419	0.003212	Abcf2
ENSMUSG00000028965	46.48278	12.1479	1.936	5.39E-07	9.19E-06	Tnfrsf9
ENSMUSG00000028967	636.3622	477.9728	0.41292	0.000292	0.002358	Errfi1
ENSMUSG00000028980	755.786	519.5827	0.54062	9.98E-07	1.59E-05	H6pd
ENSMUSG00000029012	221.7538	167.3356	0.40621	0.005849	0.029164	Orc5
ENSMUSG00000029014	667.432	462.4935	0.52919	2.04E-06	3.05E-05	Dnajc2
ENSMUSG00000029086	4770.219	2951.812	0.69245	4.67E-14	2.60E-12	Prom1
ENSMUSG00000029093	484.2413	234.8135	1.0442	1.33E-08	3.20E-07	Sorcs2
ENSMUSG00000029101	246.7701	112.9123	1.128	8.57E-05	0.000815	Rgs12
ENSMUSG00000029131	1263.814	989.0507	0.35367	0.000462	0.00349	Dnajb6
ENSMUSG00000029176	624.3166	421.4754	0.56683	9.46E-07	1.52E-05	Anapc4
ENSMUSG00000029209	118.2299	74.26822	0.67078	0.001378	0.008772	Gnpda2
ENSMUSG00000029217	263.5843	181.1578	0.54102	0.000255	0.002094	Tec
ENSMUSG00000029229	496.6398	244.4453	1.0227	1.14E-15	7.74E-14	Chic2
ENSMUSG00000029234	670.3036	492.5772	0.44446	4.19E-05	0.000437	Tmem165
ENSMUSG00000029246	373.0898	278.8003	0.42029	0.001056	0.007028	Ppat
ENSMUSG00000029247	1400.953	833.968	0.74834	6.87E-14	3.69E-12	Paics
ENSMUSG00000029249	355.6309	256.6818	0.4704	0.000306	0.00245	Rest

ENSMUSG00000029250	891.8454	682.9961	0.38492	0.003946	0.021132	Polr2b
ENSMUSG00000029276	72.16439	36.32863	0.99018	0.000196	0.001682	Glmn
ENSMUSG00000029304	544.6159	57.30865	3.2484	2.73E-05	0.000299	Spp1
ENSMUSG00000029328	2139.813	1622.772	0.39902	1.60E-05	0.000186	Hnrnpdl
ENSMUSG00000029364	846.4621	693.168	0.28824	0.010606	0.047702	Wsb2
ENSMUSG00000029368	82.59138	7.703367	3.4224	6.46E-22	7.92E-20	Alb
ENSMUSG00000029371	1133.305	101.2711	3.4842	9.42E-28	1.60E-25	Cxcl5
ENSMUSG00000029372	26.4321	2.770577	3.254	4.83E-08	1.05E-06	Ppbp
ENSMUSG00000029373	139.2401	53.7511	1.3732	1.86E-07	3.53E-06	Pf4
ENSMUSG00000029377	143.2784	58.48794	1.2926	8.92E-10	2.69E-08	Ereg
ENSMUSG00000029378	242.8285	113.336	1.0993	7.81E-12	3.31E-10	Areg
ENSMUSG00000029379	75.51179	4.412734	4.097	3.77E-17	3.00E-15	Cxcl3
ENSMUSG00000029380	157.7118	11.98904	3.7175	7.00E-19	6.71E-17	Cxcl1
ENSMUSG00000029401	214.0801	154.8077	0.46767	0.003513	0.01916	Rilpl2
ENSMUSG00000029415	363.6485	279.3802	0.38031	0.006667	0.032687	Sdad1
ENSMUSG00000029465	2088.676	1650.881	0.33935	0.000495	0.003704	Arpc3
ENSMUSG00000029484	1768.159	1427.182	0.30908	0.000817	0.005662	Anxa3
ENSMUSG00000029541	26.17779	11.77364	1.1528	0.007449	0.035716	Cyp2w1
ENSMUSG00000029552	2476.572	1421.626	0.8008	2.93E-17	2.37E-15	Tes
ENSMUSG00000029570	293.3057	182.02	0.68831	0.000187	0.001622	Lfng
ENSMUSG00000029581	173.0649	102.6788	0.75317	1.87E-05	0.000214	Fscn1
ENSMUSG00000029614	6359.855	4618.164	0.46167	1.51E-07	2.93E-06	Rpl6
ENSMUSG00000029629	539.1731	377.9684	0.51248	0.000102	0.000952	Phf14
ENSMUSG00000029659	90.46689	54.19557	0.73922	0.001916	0.011624	Medag
ENSMUSG00000029661	3347.52	2526.34	0.40604	9.91E-06	0.000123	Col1a2
ENSMUSG00000029669	883.162	417.0228	1.0826	7.60E-21	8.32E-19	Tspan12
ENSMUSG00000029670	179.9287	132.7304	0.43893	0.008674	0.040483	Ing3
ENSMUSG00000029671	28.74844	2.764218	3.3785	6.90E-05	0.000672	Wnt16
ENSMUSG00000029672	380.0933	226.8854	0.74439	0.001248	0.008067	Fam3c
ENSMUSG00000029675	545.5198	319.3591	0.77245	0.009429	0.043388	Eln
ENSMUSG00000029752	588.4945	451.271	0.38303	0.000862	0.00592	Asns
ENSMUSG00000029763	726.6707	472.7715	0.62016	3.03E-08	6.79E-07	Exoc4
ENSMUSG00000029767	2935.07	2053.951	0.51499	1.07E-07	2.14E-06	Calu
ENSMUSG00000029769	66.84039	38.88414	0.78154	0.00391	0.020976	Ccdc136
ENSMUSG00000029810	2019.559	1499.34	0.42971	2.42E-06	3.54E-05	Tmem176b
ENSMUSG00000029816	121.1738	53.49894	1.1795	0.00186	0.011353	Gpnmb
ENSMUSG00000029819	14.65482	2.054191	2.8347	0.000163	0.001438	Npy
ENSMUSG00000029822	442.4625	342.4006	0.36987	0.003395	0.018612	Osbpl3
ENSMUSG00000029830	24.89242	7.069269	1.8161	0.000222	0.001868	Svopl
ENSMUSG00000029832	282.9889	42.44067	2.7372	3.20E-12	1.44E-10	Nfe2l3
ENSMUSG00000029836	1413.633	1025.323	0.46333	6.59E-05	0.000648	Cbx3
ENSMUSG00000029838	704.832	200.8458	1.8112	0.002694	0.015394	Ptn
ENSMUSG00000029851	317.218	212.9954	0.57465	0.001088	0.007193	Tcaf2
ENSMUSG00000029865	3611.884	1911.606	0.91797	2.05E-05	0.000231	Sval1
ENSMUSG00000029868	441.6596	174.7598	1.3376	1.66E-22	2.19E-20	Trpv6
ENSMUSG00000029869	286.1086	111.7514	1.3563	1.43E-05	0.000168	Ephb6
ENSMUSG00000029882	11.00738	0	Inf	6.05E-06	7.95E-05	2210010C04Rik
ENSMUSG00000029910	562.8654	453.2708	0.31242	0.008252	0.038902	Mad2l1
ENSMUSG00000029915	64.73946	19.6289	1.7217	1.55E-08	3.67E-07	Clec5a
ENSMUSG00000029919	149.8299	86.86694	0.78645	6.67E-05	0.000654	Hpgds
ENSMUSG00000029920	346.9908	246.7327	0.49195	0.000194	0.001669	Smarcad1
ENSMUSG00000030017	26734.32	2338.669	3.5149	1.44E-59	1.03E-56	Reg3g

ENSMUSG00000030022	106.2052	55.0956	0.94684	8.06E-06	0.000103	Adamts9
ENSMUSG00000030043	46.99847	25.43023	0.88607	0.005605	0.028168	Tacr1
ENSMUSG00000030057	3395.971	2678.224	0.34255	0.000198	0.001696	Cnbp
ENSMUSG00000030069	11.15114	0.304351	5.1953	0.000222	0.001865	Prok2
ENSMUSG00000030075	52.51855	7.075628	2.8919	3.96E-10	1.27E-08	Cntn3
ENSMUSG00000030088	2277.869	1315.203	0.7924	3.41E-17	2.74E-15	Aldh1l1
ENSMUSG00000030093	5.983266	0	Inf	0.001559	0.00976	Wnt7a
ENSMUSG00000030103	1687.583	982.6943	0.78014	3.51E-15	2.22E-13	Bhlhe40
ENSMUSG00000030107	193.7033	117.4411	0.72191	0.003011	0.01689	Usp18
ENSMUSG00000030108	11.94032	1.62308	2.879	0.001528	0.009593	Slc6a13
ENSMUSG00000030110	449.307	107.0982	2.0688	3.85E-44	1.76E-41	Ret
ENSMUSG00000030111	22.01475	3.081287	2.8369	4.90E-06	6.59E-05	A2m
ENSMUSG00000030116	58.24078	35.04195	0.73295	0.010635	0.047809	Mfap5
ENSMUSG00000030123	256.7067	196.4692	0.38582	0.010406	0.046979	Plxnd1
ENSMUSG00000030134	53.05347	14.24622	1.8969	4.51E-08	9.81E-07	Rasgef1a
ENSMUSG00000030137	10.23754	1.940149	2.3996	0.006022	0.029911	Tuba8
ENSMUSG00000030142	65.83545	5.452547	3.5939	9.50E-19	9.01E-17	Clec4e
ENSMUSG00000030144	58.00562	9.763917	2.5707	8.84E-12	3.71E-10	Clec4d
ENSMUSG00000030162	16.9188	3.074928	2.46	0.00034	0.002685	Olr1
ENSMUSG00000030180	817.8973	678.4977	0.26958	0.007092	0.034367	Kdm5a
ENSMUSG00000030218	792.3907	301.1431	1.3958	3.22E-07	5.79E-06	Mgp
ENSMUSG00000030219	22.76525	2.783295	3.032	7.22E-07	1.20E-05	Erp27
ENSMUSG00000030223	271.0229	117.9393	1.2004	1.60E-14	9.54E-13	Ptpro
ENSMUSG00000030224	1628.722	1185.454	0.4583	2.09E-06	3.11E-05	Strap
ENSMUSG00000030232	759.5053	617.9297	0.29762	0.004344	0.022841	Aebp2
ENSMUSG00000030247	214.7975	99.05193	1.1167	1.34E-10	4.66E-09	Kcnj8
ENSMUSG00000030254	160.1973	113.1651	0.50142	0.007845	0.037355	Rad18
ENSMUSG00000030263	483.8551	158.7002	1.6083	3.03E-31	6.37E-29	Lrmp
ENSMUSG00000030265	1535.382	1203.542	0.35131	0.00402	0.021425	Kras
ENSMUSG00000030282	3372.308	2353.129	0.51916	1.08E-08	2.64E-07	Cmas
ENSMUSG00000030315	634.9391	434.8399	0.54613	6.26E-06	8.19E-05	Vgll4
ENSMUSG00000030317	51.0703	23.29375	1.1325	0.000288	0.002326	Timp4
ENSMUSG00000030341	919.9313	749.2427	0.29609	0.005723	0.028684	Tnfrsf1a
ENSMUSG00000030342	3659.733	2594.872	0.49608	0.000333	0.00264	Cd9
ENSMUSG00000030366	118.3739	28.68975	2.0447	1.07E-08	2.63E-07	Ceacam12
ENSMUSG00000030386	136.5846	93.51717	0.54649	0.003152	0.017488	Zfp606
ENSMUSG00000030413	5040.694	3242.476	0.63653	3.30E-05	0.000354	Pglyrp1
ENSMUSG00000030417	562.2723	433.7903	0.37427	0.002186	0.012973	Pdcd5
ENSMUSG00000030421	564.4699	386.7919	0.54534	3.04E-06	4.32E-05	Uri1
ENSMUSG00000030428	164.5042	99.06608	0.73166	0.000221	0.001858	Ttyh1
ENSMUSG00000030471	1926.11	1121.279	0.78055	1.94E-14	1.13E-12	Zdhhc13
ENSMUSG00000030510	82.66989	6.695009	3.6262	2.83E-10	9.33E-09	Cers3
ENSMUSG00000030519	29.98144	11.79271	1.3462	0.002667	0.015273	Apba2
ENSMUSG00000030521	325.2622	226.7412	0.52056	0.001179	0.007691	Mphosph10
ENSMUSG00000030530	1247.364	867.1988	0.52445	0.000305	0.002445	Furin
ENSMUSG00000030557	572.7696	454.2397	0.3345	0.00402	0.021425	Mef2a
ENSMUSG00000030559	17.10815	4.69165	1.8665	0.002311	0.013597	Rab38
ENSMUSG00000030579	224.6963	129.7455	0.79229	9.63E-07	1.54E-05	Tyrobp
ENSMUSG00000030605	1022.874	749.9632	0.44774	9.17E-06	0.000114	Mfge8
ENSMUSG00000030616	696.4886	532.4207	0.38753	0.000405	0.003118	Sytl2
ENSMUSG00000030659	282.12	179.837	0.64962	0.000208	0.001766	Nucb2
ENSMUSG00000030662	1783.819	1451.552	0.29737	0.00148	0.009342	Ipo5

ENSMUSG00000030663	933.5203	573.6441	0.70253	1.54E-10	5.29E-09	1110004F10Rik
ENSMUSG00000030671	307.803	232.0045	0.40785	0.002157	0.01282	Pde3b
ENSMUSG00000030704	1631.235	1303.136	0.32398	0.000842	0.005802	Rab6a
ENSMUSG00000030707	645.5685	410.0948	0.65461	1.97E-08	4.60E-07	Coro1a
ENSMUSG00000030718	621.8632	503.3069	0.30516	0.005834	0.029107	Ppme1
ENSMUSG00000030725	128.8916	82.13052	0.65017	0.001075	0.007132	Lipt2
ENSMUSG00000030729	434.581	228.8982	0.92492	2.68E-06	3.87E-05	Pgm2l1
ENSMUSG00000030742	66.72302	34.20517	0.96397	0.000659	0.004742	Lat
ENSMUSG00000030744	10720.75	7278.452	0.5587	6.11E-11	2.22E-09	Rps3
ENSMUSG00000030747	828.3579	521.4432	0.66774	0.00793	0.037651	Dgat2
ENSMUSG00000030748	918.0524	600.2825	0.61294	1.77E-08	4.16E-07	Il4ra
ENSMUSG00000030753	789.7449	554.9319	0.50908	6.45E-05	0.000636	Prkrir
ENSMUSG00000030760	243.3302	177.2651	0.45701	0.004917	0.025289	Acer3
ENSMUSG00000030772	538.945	392.6317	0.45696	0.002615	0.015028	Dkk3
ENSMUSG00000030786	300.3768	163.7272	0.87548	3.24E-09	8.95E-08	Itgam
ENSMUSG00000030795	2727.79	2153.784	0.34086	0.000139	0.001249	Fus
ENSMUSG00000030796	329.0942	92.96498	1.8237	0.000111	0.001027	Tead2
ENSMUSG00000030798	150.0386	107.7186	0.47807	0.007854	0.037388	Cd37
ENSMUSG00000030849	603.2031	414.7742	0.54032	3.04E-06	4.33E-05	Fgfr2
ENSMUSG00000030873	142.3	85.01543	0.74314	0.000251	0.002069	Scnn1b
ENSMUSG00000030930	236.3366	167.4239	0.49734	0.000655	0.004712	Chst15
ENSMUSG00000030942	372.4902	297.759	0.32306	0.011204	0.049763	Thumpd1
ENSMUSG00000030954	21.96915	3.696348	2.5713	1.64E-05	0.00019	Gp2
ENSMUSG00000030978	1152.658	902.1102	0.35359	0.000231	0.001935	Rrm1
ENSMUSG00000030980	551.8906	392.0046	0.49351	1.91E-05	0.000218	Knop1
ENSMUSG00000030986	1460.396	1219.321	0.26028	0.009465	0.043493	Dhx32
ENSMUSG00000031068	970.6038	760.6783	0.3516	0.000403	0.003112	Glrx3
ENSMUSG00000031074	11.84904	0.608702	4.2829	0.001035	0.006915	Fgf3
ENSMUSG00000031093	295.8821	177.8983	0.73397	3.14E-07	5.68E-06	Dock11
ENSMUSG00000031098	90.54556	27.51656	1.7183	7.75E-11	2.78E-09	Syt8
ENSMUSG00000031112	102.2896	54.1307	0.91814	0.004794	0.024722	Stk26
ENSMUSG00000031119	431.7884	336.2315	0.36087	0.003112	0.017319	Gpc4
ENSMUSG00000031150	182.8009	120.9969	0.5953	0.004341	0.022832	Ccdc120
ENSMUSG00000031167	5701.147	2647.781	1.1065	2.61E-34	6.22E-32	Rbm3
ENSMUSG00000031176	869.3167	643.9291	0.43298	5.09E-05	0.000519	Dynlt3
ENSMUSG00000031198	462.5313	323.9484	0.51379	0.001748	0.010762	Fundc2
ENSMUSG00000031207	1016.718	674.8866	0.5912	1.83E-06	2.77E-05	Msn
ENSMUSG00000031226	309.3289	220.2506	0.49	0.000697	0.004957	Pbdc1
ENSMUSG00000031246	2063.455	1484.928	0.47467	0.000999	0.006718	Sh3bgrl
ENSMUSG00000031257	1086.769	830.2569	0.38842	0.002063	0.012392	Nox1
ENSMUSG00000031278	507.1559	310.114	0.70963	2.82E-06	4.04E-05	Acsl4
ENSMUSG00000031289	26.96694	8.93951	1.5929	0.000879	0.006015	Il13ra2
ENSMUSG00000031303	46.43722	23.05931	1.0099	0.003138	0.017423	Map3k15
ENSMUSG00000031311	3086.704	2625.65	0.23339	0.007233	0.03489	Nono
ENSMUSG00000031320	9092.93	6774.774	0.42457	1.42E-06	2.20E-05	Rps4x
ENSMUSG00000031342	138.6662	100.5166	0.46418	0.008344	0.039227	Gpm6b
ENSMUSG00000031351	49.73917	17.1118	1.5394	1.85E-06	2.80E-05	Zfp185
ENSMUSG00000031353	1820.375	1514.279	0.2656	0.005695	0.028584	Rbbp7
ENSMUSG00000031371	159.3433	112.9119	0.49694	0.00573	0.028691	Haus7
ENSMUSG00000031383	37.98127	9.655895	1.9758	9.28E-07	1.50E-05	Dusp9
ENSMUSG00000031422	2389.964	1889.779	0.33877	0.000232	0.001945	Morf4l2
ENSMUSG00000031430	386.2496	138.3597	1.4811	3.18E-09	8.80E-08	Vsig1

ENSMUSG00000031438	3895.331	2347.72	0.73049	2.00E-10	6.71E-09	Rnf128
ENSMUSG00000031441	741.0324	527.497	0.49037	1.27E-05	0.000153	Atp11a
ENSMUSG00000031443	8.390993	0.602344	3.8002	0.001653	0.010274	F7
ENSMUSG00000031465	125.9297	32.88648	1.9371	1.36E-16	1.01E-14	Angpt2
ENSMUSG00000031480	30.92132	13.20005	1.2281	0.002418	0.014075	Thsd1
ENSMUSG00000031490	410.4383	201.6816	1.0251	1.76E-13	9.09E-12	Eif4ebp1
ENSMUSG00000031502	3593.185	1973.596	0.86444	3.49E-21	3.99E-19	Col4a1
ENSMUSG00000031503	2286.895	1342.308	0.76867	1.48E-15	9.91E-14	Col4a2
ENSMUSG00000031530	346.6782	89.88505	1.9474	4.07E-14	2.31E-12	Dusp4
ENSMUSG00000031538	1173.938	160.3556	2.872	9.22E-24	1.32E-21	Plat
ENSMUSG00000031576	7.340567	1.020737	2.8463	0.0077	0.036751	Kcnu1
ENSMUSG00000031578	274.944	210.1004	0.38806	0.005765	0.028834	Mak16
ENSMUSG00000031585	327.0577	249.7388	0.38913	0.003416	0.018708	Gtf2e2
ENSMUSG00000031595	95.09934	13.58703	2.8072	5.99E-06	7.88E-05	Pdgfrl
ENSMUSG00000031596	63.98898	35.07307	0.86746	0.001074	0.007129	Slc7a2
ENSMUSG00000031601	515.6912	416.0978	0.30958	0.006614	0.032454	Cnot7
ENSMUSG00000031617	246.1888	116.0113	1.0855	1.16E-11	4.80E-10	Tmem184c
ENSMUSG00000031633	1280.553	790.4757	0.69597	1.21E-11	4.96E-10	Slc25a4
ENSMUSG00000031642	540.5449	376.3783	0.52223	1.22E-05	0.000148	Sh3rf1
ENSMUSG00000031661	1182.453	301.3607	1.9722	3.47E-06	4.86E-05	Nkd1
ENSMUSG00000031697	190.9098	129.0856	0.56456	0.001103	0.007261	Orc6
ENSMUSG00000031722	534.8547	58.40561	3.195	8.71E-91	1.20E-87	Hp
ENSMUSG00000031737	36.20649	1.217405	4.8944	2.16E-06	3.18E-05	Irx5
ENSMUSG00000031740	544.4719	399.0297	0.44836	0.000156	0.001377	Mmp2
ENSMUSG00000031750	122.8892	69.03141	0.83203	4.15E-05	0.000434	Il34
ENSMUSG00000031754	623.4677	502.5076	0.31117	0.005278	0.026822	Nudt21
ENSMUSG00000031757	193.0709	25.37338	2.9277	0.000108	0.000997	Mt4
ENSMUSG00000031760	280.4385	76.84318	1.8677	0.00249	0.014425	Mt3
ENSMUSG00000031781	448.5384	348.2221	0.36522	0.00254	0.014662	Ciapin1
ENSMUSG00000031785	620.7525	421.5204	0.55842	0.00034	0.002685	Adgrg1
ENSMUSG00000031790	1188.905	946.5134	0.32894	0.001259	0.008123	Mmp15
ENSMUSG00000031799	2900.778	1708.239	0.76393	6.75E-17	5.23E-15	Tpm4
ENSMUSG00000031841	264.5567	95.39979	1.4715	0.000147	0.001312	Cdh13
ENSMUSG00000031843	262.8394	166.3848	0.65966	2.72E-05	0.000297	Mphosph6
ENSMUSG00000031871	225.035	116.7785	0.94637	6.50E-09	1.67E-07	Cdh5
ENSMUSG00000031877	310.5496	190.3053	0.70651	6.73E-07	1.13E-05	Ces2g
ENSMUSG00000031885	759.5614	578.5312	0.39277	0.000382	0.002969	Cbfb
ENSMUSG00000031906	1908.304	1504.507	0.343	0.000435	0.003319	Smpd3
ENSMUSG00000031907	98.64889	50.29623	0.97185	2.33E-05	0.000259	Zfp90
ENSMUSG00000031917	300.3905	171.6594	0.80729	2.64E-08	5.99E-07	Nip7
ENSMUSG00000031939	348.178	269.6535	0.36872	0.006814	0.033309	Taf1d
ENSMUSG00000031957	52.0225	0	Inf	1.92E-25	3.00E-23	Ctrb1
ENSMUSG00000031958	105.6109	59.96548	0.81655	0.000395	0.003053	Ldhd
ENSMUSG00000031963	43.44211	22.40647	0.95518	0.011118	0.049432	Bmper
ENSMUSG00000031983	472.202	371.5203	0.34596	0.005123	0.026167	2310022B05Rik
ENSMUSG00000031996	2936.536	2392.291	0.29572	0.000841	0.005797	Aplp2
ENSMUSG00000032000	899.6635	641.6979	0.48749	2.85E-05	0.00031	Birc3
ENSMUSG00000032002	509.4799	417.4151	0.28754	0.008127	0.038413	Dcun1d5
ENSMUSG00000032006	54.87381	32.26506	0.76615	0.010057	0.045726	Pdgfd
ENSMUSG00000032011	253.7835	145.2405	0.80515	3.01E-07	5.45E-06	Thy1
ENSMUSG00000032013	52.94299	14.13218	1.9055	9.11E-07	1.47E-05	Trim29
ENSMUSG00000032035	431.9116	273.102	0.6613	4.01E-07	7.03E-06	Ets1

ENSMUSG00000032041	361.6128	279.8727	0.36968	0.004803	0.02476	Tirap
ENSMUSG00000032050	352.0344	241.1962	0.54551	8.01E-05	0.000769	Rdx
ENSMUSG00000032068	5799.646	4090.834	0.50357	0.001994	0.012043	Plet1
ENSMUSG00000032092	660.6843	482.0221	0.45486	6.75E-05	0.00066	Mpzl2
ENSMUSG00000032184	94.21222	51.24078	0.87862	0.000667	0.004785	Lysmd2
ENSMUSG00000032186	153.8364	85.04693	0.85507	4.48E-06	6.08E-05	Tmod2
ENSMUSG00000032228	1012.969	728.7805	0.47503	2.75E-05	0.000301	Tcf12
ENSMUSG00000032231	10708.36	6216.964	0.78446	4.04E-19	3.94E-17	Anxa2
ENSMUSG00000032232	716.1213	508.6896	0.49342	6.54E-06	8.48E-05	Cgnl1
ENSMUSG00000032244	589.8127	483.6198	0.28638	0.010665	0.047902	Fem1b
ENSMUSG00000032281	69.01297	16.12316	2.0977	2.30E-09	6.53E-08	Acsbg1
ENSMUSG00000032294	16672.46	13217.63	0.335	0.000131	0.001183	Pkm
ENSMUSG00000032300	389.2399	213.5874	0.86583	1.11E-05	0.000135	1700017B05Rik
ENSMUSG00000032313	28.55916	11.94487	1.2576	0.004353	0.022864	AI118078
ENSMUSG00000032320	412.467	313.9387	0.3938	0.002165	0.012863	Rcn2
ENSMUSG00000032322	73.13051	44.50156	0.71662	0.004262	0.022451	Pstpip1
ENSMUSG00000032327	647.2983	85.364	2.9227	1.16E-06	1.82E-05	Stra6
ENSMUSG00000032348	471.5165	253.8985	0.89306	1.92E-11	7.55E-10	Gsta4
ENSMUSG00000032359	795.5998	648.1753	0.29566	0.008087	0.038313	Ctsh
ENSMUSG00000032363	58.99121	28.18207	1.0657	0.000303	0.00243	Adamts7
ENSMUSG00000032369	2299.193	1380.599	0.73583	3.96E-15	2.50E-13	Plscr1
ENSMUSG00000032383	989.9142	795.1469	0.31608	0.006452	0.031737	Ppib
ENSMUSG00000032388	457.4228	347.6609	0.39585	0.001597	0.009959	Spg21
ENSMUSG00000032399	14825.74	11049.55	0.42412	7.39E-07	1.23E-05	Rpl4
ENSMUSG00000032402	522.9597	411.7361	0.34498	0.003023	0.016936	Smad3
ENSMUSG00000032420	1932.374	1183.462	0.70736	4.77E-11	1.75E-09	Nt5e
ENSMUSG00000032423	1495.981	1185.743	0.3353	0.000387	0.002999	Syncrip
ENSMUSG00000032436	140.206	100.7828	0.4763	0.007942	0.037689	Cmtm7
ENSMUSG00000032452	29.68144	12.13518	1.2904	0.003132	0.017409	Clstn2
ENSMUSG00000032481	915.1227	624.7639	0.55065	9.98E-06	0.000123	Smarcc1
ENSMUSG00000032484	84.55519	0.304351	8.118	1.24E-38	3.96E-36	Ngp
ENSMUSG00000032487	148.9423	59.98358	1.3121	5.08E-11	1.86E-09	Ptgs2
ENSMUSG00000032491	56.19228	25.54427	1.1374	0.01116	0.049593	Nradd
ENSMUSG00000032496	183.2955	31.89716	2.5227	5.41E-31	1.11E-28	Ltf
ENSMUSG00000032551	178.6765	127.2849	0.48929	0.003349	0.018409	1110059G10Rik
ENSMUSG00000032554	121.095	47.91297	1.3377	1.48E-08	3.53E-07	Trf
ENSMUSG00000032624	895.7629	728.0849	0.29901	0.004736	0.024507	Eml4
ENSMUSG00000032643	66.6771	32.6012	1.0323	0.006856	0.033477	Fhl3
ENSMUSG00000032666	437.1757	330.2829	0.40451	0.005782	0.028911	1700025G04Rik
ENSMUSG00000032688	325.5638	234.1985	0.47521	0.00039	0.003019	Malt1
ENSMUSG00000032691	25.9428	9.554571	1.4411	0.00158	0.009864	Nlrp3
ENSMUSG00000032698	175.0619	69.48796	1.333	8.96E-07	1.45E-05	Lmo2
ENSMUSG00000032702	734.3958	513.5842	0.51596	4.59E-06	6.21E-05	Kank1
ENSMUSG00000032717	85.31259	15.50208	2.4603	4.76E-07	8.24E-06	Mdfi
ENSMUSG00000032719	62.22118	17.74624	1.8099	0.0001	0.000933	Sbspon
ENSMUSG00000032796	106.2702	59.43275	0.83841	0.000131	0.001187	Lama1
ENSMUSG00000032849	479.5766	252.0266	0.92819	1.88E-05	0.000215	Abcc4
ENSMUSG00000032860	348.3888	228.4626	0.60874	1.76E-05	0.000203	P2ry2
ENSMUSG00000032883	1631.821	1254.798	0.37903	0.000124	0.00113	Acsl3
ENSMUSG00000032940	27.90678	3.068569	3.185	2.13E-08	4.91E-07	Rbm11
ENSMUSG00000032965	64.34147	36.61424	0.81334	0.002357	0.013797	Ift57
ENSMUSG00000033032	381.8277	179.1969	1.0914	1.82E-12	8.49E-11	Afap1l1

ENSMUSG00000033096	604.0364	446.1069	0.43725	0.000246	0.00204	Apmap
ENSMUSG00000033149	421.44	196.7529	1.0989	2.20E-16	1.57E-14	Phldb2
ENSMUSG00000033186	518.0397	377.8271	0.45534	8.58E-05	0.000815	Mzt1
ENSMUSG00000033192	399.4045	258.0952	0.62995	2.49E-06	3.62E-05	Lpcat2
ENSMUSG00000033227	127.483	15.18501	3.0696	2.25E-05	0.000251	Wnt6
ENSMUSG00000033237	679.1319	551.233	0.30103	0.00497	0.02551	Arid2
ENSMUSG00000033256	186.3043	121.4525	0.61727	0.002731	0.01556	Shf
ENSMUSG00000033285	324.2255	244.8046	0.40537	0.001871	0.011391	Wdr3
ENSMUSG00000033307	1066.283	788.4552	0.43549	0.000467	0.003524	Mif
ENSMUSG00000033453	348.1839	152.2084	1.1938	5.18E-12	2.27E-10	Adamts15
ENSMUSG00000033467	196.0652	102.1413	0.94077	1.46E-07	2.83E-06	Crlf2
ENSMUSG00000033508	170.6319	39.30889	2.118	0.000481	0.003609	Asprv1
ENSMUSG00000033545	396.2683	265.031	0.58032	0.003137	0.017423	Znrf1
ENSMUSG00000033658	374.3825	287.4054	0.38143	0.003309	0.018219	Ddx19b
ENSMUSG00000033767	257.9076	196.6207	0.39144	0.005594	0.028123	D930015E06Rik
ENSMUSG00000033777	59.92412	36.63964	0.70973	0.010137	0.045987	Tlr13
ENSMUSG00000033793	868.7225	673.2487	0.36776	0.000937	0.006358	Atp6v1h
ENSMUSG00000033808	757.056	611.3404	0.30842	0.00529	0.026844	Tmem87a
ENSMUSG00000033849	122.7645	60.3762	1.0238	6.76E-06	8.73E-05	B3galt2
ENSMUSG00000033906	311.5543	145.5055	1.0984	7.46E-14	4.00E-12	Zdhhc15
ENSMUSG00000033917	654.2336	441.8416	0.56628	1.37E-06	2.14E-05	Gde1
ENSMUSG00000033931	230.5094	167.152	0.46367	0.002645	0.015169	Rbm34
ENSMUSG00000033987	81.56694	37.19785	1.1328	0.000208	0.001765	Dnah17
ENSMUSG00000033998	1148.79	944.3731	0.28269	0.00487	0.025071	Kcnk1
ENSMUSG00000034112	956.7236	719.7658	0.41057	0.000108	0.000997	Atp2c2
ENSMUSG00000034116	202.7797	142.0252	0.51377	0.000579	0.004258	Vav1
ENSMUSG00000034158	634.9798	473.9792	0.42189	0.000148	0.001314	Lrrc58
ENSMUSG00000034177	911.2216	406.7094	1.1638	8.68E-06	0.000109	Rnf43
ENSMUSG00000034194	202.6093	137.6459	0.55774	0.007062	0.034275	R3hcc1
ENSMUSG00000034205	804.7475	485.0145	0.73051	4.27E-11	1.59E-09	Loxl2
ENSMUSG00000034220	465.3473	175.7999	1.4044	0.000304	0.002438	Gpc1
ENSMUSG00000034265	130.5486	76.79867	0.76543	0.000447	0.003399	Zdhhc14
ENSMUSG00000034324	82.27187	15.34954	2.4222	2.03E-08	4.73E-07	Tmem132c
ENSMUSG00000034345	435.9773	303.2313	0.52383	2.51E-05	0.000277	Gtf2h5
ENSMUSG00000034362	103.9344	40.15237	1.3721	9.48E-09	2.36E-07	Csta1
ENSMUSG00000034382	859.9484	554.4613	0.63316	6.93E-09	1.76E-07	AI661453
ENSMUSG00000034394	98.70844	51.63472	0.93483	0.002204	0.013059	Lif
ENSMUSG00000034401	257.2275	118.4535	1.1187	1.95E-08	4.56E-07	Spata6
ENSMUSG00000034450	19.26141	0.722744	4.7361	0.008454	0.039695	Gulo
ENSMUSG00000034453	364.634	269.6535	0.43534	0.001143	0.007493	Polr3b
ENSMUSG00000034463	109.9176	48.71792	1.1739	2.95E-07	5.36E-06	Scara3
ENSMUSG00000034473	309.0237	236.8764	0.38358	0.006835	0.033382	Sec22a
ENSMUSG00000034484	1033.867	819.7356	0.33482	0.001085	0.007185	Snx2
ENSMUSG00000034486	15.70518	0.297992	5.7198	0.000404	0.003118	Gbx2
ENSMUSG00000034544	307.1631	223.7252	0.45728	0.001215	0.007879	Rsrc1
ENSMUSG00000034610	295.0333	226.9402	0.37857	0.009459	0.043484	Zcchc11
ENSMUSG00000034612	150.2278	72.34714	1.0541	7.24E-08	1.51E-06	Chst11
ENSMUSG00000034620	280.6733	215.4324	0.38166	0.00646	0.03177	Tmem5
ENSMUSG00000034634	308.9129	92.12824	1.7455	7.92E-20	8.17E-18	Ly6d
ENSMUSG00000034674	919.525	706.3801	0.38044	0.00024	0.001994	Tdg
ENSMUSG00000034675	339.7616	130.8107	1.377	0.000119	0.001087	Dbn1
ENSMUSG00000034684	347.9441	215.1925	0.69323	0.005969	0.029684	Sema3f

ENSMUSG00000034685	60.55712	34.98514	0.79155	0.004291	0.022589	Fam171a2
ENSMUSG00000034687	87.0154	13.66325	2.671	2.63E-11	1.01E-09	Fras1
ENSMUSG00000034738	817.5965	547.481	0.57858	1.04E-06	1.65E-05	Nostrin
ENSMUSG00000034765	141.7914	87.66603	0.69368	0.000433	0.00331	Dusp5
ENSMUSG00000034795	186.1997	103.3434	0.8494	3.42E-07	6.10E-06	Ccdc122
ENSMUSG00000034807	833.8527	592.7602	0.49234	9.13E-06	0.000114	Colgalt1
ENSMUSG00000034893	765.2913	619.4127	0.30511	0.005482	0.027654	Cog3
ENSMUSG00000034912	40.83257	16.0914	1.3434	0.000175	0.001527	Mdga2
ENSMUSG00000035020	10.40066	1.033455	3.3311	0.000671	0.004798	Epgn
ENSMUSG00000035021	612.1533	440.5389	0.47462	4.34E-05	0.000451	Baz1a
ENSMUSG00000035095	57.11826	8.445188	2.7578	1.23E-09	3.64E-08	Fam167a
ENSMUSG00000035105	1701.011	646.6922	1.3952	1.14E-41	4.52E-39	Egln3
ENSMUSG00000035142	101.9966	64.68893	0.65693	0.002521	0.014581	Nubpl
ENSMUSG00000035150	2923.912	1823.784	0.68097	1.33E-13	6.91E-12	Eif2s3x
ENSMUSG00000035164	60.231	30.16601	0.99758	0.000282	0.002281	Zc3h12c
ENSMUSG00000035184	516.9685	198.6551	1.3798	2.86E-21	3.31E-19	Fam124a
ENSMUSG00000035212	1541.063	1172.392	0.39447	7.00E-05	0.00068	Leprot
ENSMUSG00000035235	62.52127	36.55103	0.77443	0.00377	0.020372	Trim13
ENSMUSG00000035246	25.4272	9.224784	1.4628	0.001867	0.011382	Pcyt1b
ENSMUSG00000035274	94.55782	50.56879	0.90295	0.000111	0.001027	Tpbg
ENSMUSG00000035299	683.8684	492.3331	0.47408	2.80E-05	0.000305	Mid1
ENSMUSG00000035349	207.8289	90.92987	1.1926	5.95E-09	1.55E-07	Mia2
ENSMUSG00000035356	1329.615	940.6444	0.49929	1.50E-06	2.32E-05	Nfkbiz
ENSMUSG00000035373	49.49724	24.92319	0.98986	0.002854	0.016135	Ccl7
ENSMUSG00000035376	412.2723	322.4163	0.35467	0.004703	0.024376	Hacd2
ENSMUSG00000035385	35.97172	16.64924	1.1114	0.002434	0.014143	Ccl2
ENSMUSG00000035437	543.7297	362.4164	0.58524	6.29E-07	1.06E-05	Rabgap1
ENSMUSG00000035443	90.30373	57.0043	0.66371	0.004617	0.024004	Thyn1
ENSMUSG00000035530	2549.259	2073.58	0.29795	0.00129	0.00829	Eif1
ENSMUSG00000035551	9.115175	1.153855	2.9818	0.006929	0.03377	Igfbpl1
ENSMUSG00000035557	135.3514	52.23036	1.3737	0.00304	0.017013	Krt17
ENSMUSG00000035637	241.1382	178.7498	0.43192	0.00816	0.03854	Grhpr
ENSMUSG00000035640	68.94808	41.87682	0.71936	0.00467	0.024227	Cbarp
ENSMUSG00000035673	1145.437	927.3088	0.30478	0.003003	0.016853	Sbno2
ENSMUSG00000035678	39.28599	12.98468	1.5972	5.07E-05	0.000517	Tnfsf9
ENSMUSG00000035818	57.61458	23.12888	1.3167	1.29E-05	0.000154	Plekhs1
ENSMUSG00000035828	766.7682	593.4932	0.36956	0.001759	0.010817	Pim3
ENSMUSG00000035896	82.41561	11.31077	2.8652	2.55E-07	4.74E-06	Rnase1
ENSMUSG00000035914	192.3	102.3814	0.9094	8.27E-06	0.000105	Cd276
ENSMUSG00000035960	162.8731	100.8085	0.69213	0.000129	0.001171	Apex1
ENSMUSG00000036006	101.709	56.05347	0.85957	0.000173	0.001514	Fam65b
ENSMUSG00000036046	552.6479	452.0545	0.28986	0.010842	0.048482	5031439G07Rik
ENSMUSG00000036053	201.1542	117.1771	0.77961	3.91E-06	5.39E-05	Fmnl2
ENSMUSG00000036078	1002.385	710.8079	0.4959	1.99E-06	2.98E-05	Sigmar1
ENSMUSG00000036099	326.3859	250.6831	0.38071	0.003924	0.021038	Vezt
ENSMUSG00000036112	2599.203	1884.804	0.46365	2.87E-07	5.23E-06	Metap2
ENSMUSG00000036160	335.4156	259.8514	0.36826	0.007878	0.03747	Surf6
ENSMUSG00000036206	401.3822	253.5739	0.66257	2.04E-07	3.84E-06	Sh3bp4
ENSMUSG00000036256	1619.839	1106.064	0.55042	9.75E-09	2.41E-07	Igfbp7
ENSMUSG00000036264	52.32925	28.02991	0.90065	0.003081	0.017166	Fstl4
ENSMUSG00000036309	2828.7	2011.543	0.49184	9.14E-08	1.85E-06	Skp1a
ENSMUSG00000036323	1479.232	1228.59	0.26784	0.004177	0.022096	Srp72

ENSMUSG00000036330	111.9989	50.37954	1.1526	0.001521	0.009551	Slc18a1
ENSMUSG00000036356	32.25238	11.53885	1.4829	0.000272	0.002213	Csgalnact1
ENSMUSG00000036371	5016.748	3834.067	0.38788	8.28E-06	0.000105	Serbp1
ENSMUSG00000036381	92.4765	60.30803	0.61674	0.009733	0.044491	P2ry14
ENSMUSG00000036438	7307.171	5847.538	0.32148	0.00171	0.01058	Calm2
ENSMUSG00000036459	308.6574	234.7454	0.39491	0.006467	0.031788	Wtip
ENSMUSG00000036480	16.87319	3.068569	2.4591	0.000287	0.002323	Prss56
ENSMUSG00000036503	636.0661	486.5094	0.38671	0.000784	0.005468	Rnf13
ENSMUSG00000036513	255.9824	172.332	0.57085	0.00017	0.001487	Commd2
ENSMUSG00000036545	187.1521	135.8449	0.46225	0.007908	0.037556	Adamts2
ENSMUSG00000036565	856.4401	594.2121	0.52737	0.00545	0.027533	Ttyh3
ENSMUSG00000036632	572.7296	388.8229	0.55874	6.40E-06	8.32E-05	Alg5
ENSMUSG00000036748	380.0527	259.5996	0.54991	0.009462	0.043488	Cuedc2
ENSMUSG00000036764	85.0708	44.74172	0.92704	6.25E-05	0.000619	Dnajc12
ENSMUSG00000036781	1453.952	1193.268	0.28506	0.003824	0.020636	Rps27l
ENSMUSG00000036834	466.0434	366.8718	0.34519	0.003077	0.017155	Plch1
ENSMUSG00000036885	405.3628	294.8356	0.4593	0.000168	0.001478	Arhgef26
ENSMUSG00000036894	99.79765	58.07621	0.78106	0.000558	0.004122	Rap2b
ENSMUSG00000036902	156.0215	47.46211	1.7169	1.98E-15	1.29E-13	Neto2
ENSMUSG00000036916	157.9208	98.50648	0.68091	0.000133	0.001202	Zfp280c
ENSMUSG00000036934	123.9852	88.20377	0.49126	0.009479	0.043543	4921524J17Rik
ENSMUSG00000036938	22.88278	0	Inf	5.35E-12	2.32E-10	Try5
ENSMUSG00000036992	160.309	116.0447	0.46617	0.008538	0.039952	Nxt1
ENSMUSG00000037017	277.4623	199.6387	0.4749	0.001088	0.007193	Zscan21
ENSMUSG00000037029	405.5967	274.407	0.56373	1.29E-05	0.000154	Zfp146
ENSMUSG00000037033	11532.52	4835.899	1.2539	5.16E-16	3.61E-14	Clca3b
ENSMUSG00000037035	108.9847	33.95801	1.6823	5.42E-12	2.35E-10	Inhbb
ENSMUSG00000037070	600.2728	418.8363	0.51923	4.17E-06	5.71E-05	Rbmxl1
ENSMUSG00000037072	2931.505	2165.402	0.43701	0.000323	0.002567	15-Sep
ENSMUSG00000037095	4064.995	282.0492	3.8492	########	########	Lrg1
ENSMUSG00000037129	144.4668	32.03061	2.1732	3.24E-10	1.06E-08	Tmprss13
ENSMUSG00000037157	315.4898	236.9962	0.41273	0.005379	0.027217	Il22ra1
ENSMUSG00000037169	46.48978	25.93121	0.84222	0.006339	0.031302	Mycn
ENSMUSG00000037188	83.99415	11.77999	2.8339	1.33E-18	1.26E-16	Grhl3
ENSMUSG00000037204	488.0526	367.6808	0.40858	0.000829	0.005732	Atg101
ENSMUSG00000037236	1324.559	1105.529	0.26078	0.008561	0.040014	Matr3
ENSMUSG00000037253	645.3656	473.108	0.44795	7.98E-05	0.000766	Mex3c
ENSMUSG00000037260	388.8865	306.4195	0.34384	0.009228	0.042609	Hgsnat
ENSMUSG00000037287	407.8163	322.0491	0.34064	0.005634	0.028298	Tbcel
ENSMUSG00000037295	317.2391	213.8686	0.56885	0.003466	0.018942	Ldlrap1
ENSMUSG00000037315	168.7914	95.14661	0.82702	3.44E-06	4.82E-05	Jade3
ENSMUSG00000037361	637.5465	479.3403	0.41148	0.006347	0.031328	Sf3b6
ENSMUSG00000037369	641.1882	408.921	0.64893	5.31E-08	1.14E-06	Kdm6a
ENSMUSG00000037376	409.8452	266.736	0.61967	1.09E-06	1.73E-05	Trmt6
ENSMUSG00000037386	42.56164	16.72516	1.3475	0.000102	0.000953	Rims2
ENSMUSG00000037405	276.7377	142.2221	0.96037	5.16E-08	1.11E-06	Icam1
ENSMUSG00000037411	148.1338	53.87854	1.4591	7.53E-13	3.60E-11	Serpine1
ENSMUSG00000037447	184.7648	92.80143	0.99347	5.79E-06	7.66E-05	Arid5a
ENSMUSG00000037465	713.0225	409.4331	0.80032	7.72E-13	3.68E-11	Klf10
ENSMUSG00000037499	251.0633	177.6961	0.49864	0.000809	0.005618	Nenf
ENSMUSG00000037536	474.5851	361.189	0.39391	0.001935	0.011727	Fbxo34
ENSMUSG00000037563	7694.369	5821.734	0.40235	3.69E-06	5.14E-05	Rps16

ENSMUSG00000037568	95.09927	15.35594	2.6306	0.000229	0.001923	Vash2
ENSMUSG00000037578	8.064795	0	Inf	0.000106	0.000988	Pkd2l1
ENSMUSG00000037580	404.0902	292.2135	0.46765	0.000466	0.003514	Gch1
ENSMUSG00000037608	1698.792	1269.072	0.42074	8.77E-06	0.00011	Bclaf1
ENSMUSG00000037613	101.8528	58.53271	0.79917	0.000461	0.003483	Tnfrsf23
ENSMUSG00000037656	442.7041	259.8969	0.7684	3.61E-09	9.84E-08	Slc20a2
ENSMUSG00000037664	222.8563	97.74024	1.1891	4.49E-06	6.09E-05	Cdkn1c
ENSMUSG00000037681	46.84209	10.6325	2.1393	0.000272	0.002212	Esyt3
ENSMUSG00000037706	1993.064	1448.13	0.4608	0.000136	0.001223	Cd81
ENSMUSG00000037736	139.0376	45.01563	1.627	1.47E-05	0.000172	Limch1
ENSMUSG00000037788	447.9304	227.2128	0.97923	1.88E-13	9.68E-12	Vopp1
ENSMUSG00000037805	7423.309	5294.367	0.4876	1.68E-08	3.98E-07	Rpl10a
ENSMUSG00000037813	54.99121	28.88574	0.92884	0.002527	0.014608	D630003M21Rik
ENSMUSG00000037820	1680.568	1323.308	0.3448	0.000256	0.0021	Tgm2
ENSMUSG00000037824	658.6837	475.8866	0.46897	2.94E-05	0.000318	Tspan14
ENSMUSG00000037852	995.0757	800.6023	0.31372	0.009512	0.043635	Cpe
ENSMUSG00000037860	685.4002	526.9931	0.37916	0.001139	0.007479	Aim2
ENSMUSG00000037902	326.4968	237.6286	0.45836	0.000631	0.004575	Sirpa
ENSMUSG00000037935	1184.376	964.8948	0.29568	0.002686	0.01536	Smarce1
ENSMUSG00000037940	107.7119	72.05521	0.58	0.00579	0.028943	Inpp4b
ENSMUSG00000037972	203.3793	71.8906	1.5003	1.79E-16	1.30E-14	Snn
ENSMUSG00000038007	224.6368	153.6466	0.54798	0.000455	0.00345	Acer2
ENSMUSG00000038059	146.9203	63.33882	1.2139	2.66E-08	6.04E-07	Smim3
ENSMUSG00000038067	8.391011	0.608702	3.785	0.00165	0.010263	Csf3
ENSMUSG00000038132	133.9487	43.63332	1.6182	3.48E-05	0.000372	Rbm24
ENSMUSG00000038147	133.2179	85.50276	0.63975	0.000612	0.004456	Cd84
ENSMUSG00000038156	310.1323	203.8555	0.60534	2.65E-05	0.000291	Spon1
ENSMUSG00000038217	142.2086	94.95138	0.58275	0.004	0.021366	Tlcd2
ENSMUSG00000038242	34.96686	4.412734	2.9862	1.14E-09	3.38E-08	Aox4
ENSMUSG00000038264	443.2194	156.5456	1.5014	9.25E-14	4.91E-12	Sema7a
ENSMUSG00000038296	67.14025	29.28543	1.197	6.44E-05	0.000635	Galnt18
ENSMUSG00000038301	479.9279	256.9999	0.90105	5.09E-12	2.24E-10	Snx10
ENSMUSG00000038357	12.52771	0.608702	4.3632	2.82E-05	0.000306	Camp
ENSMUSG00000038368	260.0402	192.7274	0.43217	0.003503	0.019113	Focad
ENSMUSG00000038400	1150.377	561.898	1.0337	2.55E-08	5.81E-07	Pmepa1
ENSMUSG00000038415	222.8685	58.50126	1.9297	3.42E-07	6.10E-06	Foxq1
ENSMUSG00000038416	484.1754	386.5066	0.32504	0.005078	0.025974	Cdc16
ENSMUSG00000038446	485.8784	368.6423	0.39837	0.001273	0.008199	Cdc40
ENSMUSG00000038467	3269.711	2589.577	0.33645	0.000267	0.002178	Chmp4b
ENSMUSG00000038495	617.9756	476.7484	0.37432	0.000746	0.005251	Otud7b
ENSMUSG00000038496	20.65746	5.788653	1.8354	0.002009	0.012117	Slc19a3
ENSMUSG00000038510	207.7899	127.6465	0.70297	2.28E-05	0.000254	Rpf2
ENSMUSG00000038525	631.3499	390.2361	0.69409	1.76E-09	5.10E-08	Armc10
ENSMUSG00000038527	50.03218	27.79513	0.84802	0.009082	0.042078	C1rl
ENSMUSG00000038545	197.3961	132.3452	0.57679	0.000529	0.003928	Cul7
ENSMUSG00000038560	30.94746	8.629139	1.8425	0.005221	0.026577	Sp6
ENSMUSG00000038572	7.75791	0	Inf	0.002086	0.01251	Bpifb5
ENSMUSG00000038578	91.09971	39.02328	1.2231	5.31E-07	9.06E-06	Susd1
ENSMUSG00000038605	233.6483	173.1373	0.43242	0.003946	0.021132	Samd10
ENSMUSG00000038622	289.3183	183.9655	0.65322	3.68E-06	5.13E-05	Med30
ENSMUSG00000038650	1817.848	1272.042	0.51508	1.04E-07	2.08E-06	Rnh1
ENSMUSG00000038775	352.9226	92.24292	1.9358	4.62E-10	1.47E-08	Vill

ENSMUSG00000038780	515.0267	371.3069	0.47204	7.93E-05	0.000762	Smurf1
ENSMUSG00000038816	377.318	273.8944	0.46216	0.000473	0.003558	Ctnnal1
ENSMUSG00000038871	824.9819	621.8562	0.40778	0.000166	0.00146	Bpgm
ENSMUSG00000038879	572.7698	452.916	0.33871	0.004403	0.023072	Nipal2
ENSMUSG00000038900	204.4368	148.0438	0.46563	0.009238	0.042644	Rpl12
ENSMUSG00000038936	222.2431	148.5195	0.58149	0.000293	0.002365	Sccpdh
ENSMUSG00000039001	3697.428	2979.267	0.31156	0.000964	0.006504	Rps21
ENSMUSG00000039005	394.9017	267.9522	0.55952	0.000729	0.005152	Tlr4
ENSMUSG00000039007	742.7347	573.5116	0.37302	0.000982	0.006614	Cpq
ENSMUSG00000039016	454.685	359.2744	0.33978	0.010655	0.047886	Timm8b
ENSMUSG00000039046	614.535	482.9666	0.34757	0.002189	0.012987	Usp6nl
ENSMUSG00000039081	224.8525	146.4398	0.61867	0.000198	0.001696	Zfp503
ENSMUSG00000039109	250.2467	129.0023	0.95595	2.25E-09	6.43E-08	F13a1
ENSMUSG00000039182	179.9424	122.6752	0.55269	0.000577	0.004244	AW209491
ENSMUSG00000039217	769.7601	477.3296	0.68942	5.42E-05	0.000548	Il18
ENSMUSG00000039230	710.2028	556.9818	0.3506	0.001095	0.007231	Tbcd
ENSMUSG00000039232	70.78779	18.9951	1.8979	7.11E-10	2.18E-08	Stx11
ENSMUSG00000039286	766.6881	524.6845	0.54719	4.82E-07	8.32E-06	Fndc3b
ENSMUSG00000039323	493.2197	286.856	0.7819	3.10E-06	4.39E-05	Igfbp2
ENSMUSG00000039361	2908.658	2463.965	0.23937	0.009787	0.044649	Picalm
ENSMUSG00000039457	185.6452	106.6353	0.79986	3.28E-06	4.64E-05	Ppl
ENSMUSG00000039483	224.8981	166.8621	0.43062	0.01027	0.046484	Asb6
ENSMUSG00000039530	467.0344	331.7478	0.49344	8.08E-05	0.000774	Tusc3
ENSMUSG00000039607	190.6567	100.4924	0.92389	0.002216	0.013098	Rbms3
ENSMUSG00000039693	98.3752	38.78247	1.3429	1.72E-05	0.000199	Msantd3
ENSMUSG00000039715	79.93612	50.75278	0.65536	0.008011	0.037975	Wdr34
ENSMUSG00000039735	1421.606	1051.912	0.43451	5.93E-06	7.83E-05	Fnbp1l
ENSMUSG00000039756	532.8962	388.2631	0.45682	0.000108	0.001001	Dnttip2
ENSMUSG00000039770	620.7802	424.5	0.54832	3.70E-06	5.15E-05	Ypel5
ENSMUSG00000039911	204.0845	115.9183	0.81606	1.82E-06	2.76E-05	Spsb1
ENSMUSG00000039934	151.05	60.34576	1.3237	6.91E-10	2.13E-08	Gsap
ENSMUSG00000039976	242.2355	134.5063	0.84874	7.50E-05	0.000723	Tbc1d16
ENSMUSG00000039982	382.5586	254.2778	0.58928	0.001079	0.00715	Dtx4
ENSMUSG00000039985	575.673	318.4276	0.85428	2.14E-12	9.83E-11	Fam60a
ENSMUSG00000040003	30.05333	10.93049	1.4592	0.00121	0.007859	Magi2
ENSMUSG00000040010	301.2322	204.084	0.56171	9.38E-05	0.000884	Slc7a5
ENSMUSG00000040026	670.025	111.7918	2.5834	7.32E-21	8.07E-19	Saa3
ENSMUSG00000040093	392.4563	194.9471	1.0094	1.10E-13	5.82E-12	Bmf
ENSMUSG00000040151	364.7393	270.4954	0.43126	0.000486	0.003646	Hs2st1
ENSMUSG00000040152	1206.725	579.4311	1.0584	7.94E-10	2.41E-08	Thbs1
ENSMUSG00000040280	23.77012	8.62278	1.4629	0.003472	0.018969	Ndufa4l2
ENSMUSG00000040359	572.0717	414.6884	0.46417	4.58E-05	0.000472	Ufl1
ENSMUSG00000040380	56.04175	33.4694	0.74366	0.009635	0.044092	Cbln3
ENSMUSG00000040410	181.0316	129.8963	0.47888	0.003567	0.019416	Fbxl4
ENSMUSG00000040430	194.2903	103.7824	0.90465	1.97E-07	3.71E-06	Pitpnc1
ENSMUSG00000040435	146.3787	97.99952	0.57886	0.001048	0.006988	Ppp1r15a
ENSMUSG00000040447	297.0956	118.2073	1.3296	7.36E-17	5.63E-15	Spns2
ENSMUSG00000040451	376.1448	293.4481	0.35818	0.003618	0.019664	Sgms1
ENSMUSG00000040464	304.8397	222.659	0.45321	0.001237	0.008012	Gtpbp10
ENSMUSG00000040466	289.104	190.9665	0.59827	0.00406	0.02156	Blvrb
ENSMUSG00000040473	55.97679	22.44429	1.3185	2.06E-05	0.000232	Cfap69
ENSMUSG00000040502	80.72528	53.07317	0.60504	0.00907	0.042045	9-Mar

ENSMUSG00000040511	237.5692	151.0105	0.6537	5.80E-05	0.000581	Pvr
ENSMUSG00000040586	141.6017	93.11688	0.60472	0.001959	0.011859	Ofd1
ENSMUSG00000040592	145.4125	93.30851	0.64007	0.00096	0.006485	Cd79b
ENSMUSG00000040613	882.6607	694.7722	0.34532	0.003894	0.020917	Apobec1
ENSMUSG00000040659	2900.549	2335.964	0.31231	0.01111	0.049427	Efhd2
ENSMUSG00000040666	188.1955	122.9806	0.6138	0.003229	0.017862	Sh3bgr
ENSMUSG00000040675	327.9379	170.7724	0.94135	1.45E-10	5.01E-09	Mthfd1l
ENSMUSG00000040681	1276.995	821.8024	0.63589	1.12E-06	1.76E-05	Hmgn1
ENSMUSG00000040694	106.4855	25.11316	2.0841	5.90E-05	0.000589	Apobec2
ENSMUSG00000040699	301.7617	169.7207	0.83025	1.16E-06	1.83E-05	Limd2
ENSMUSG00000040711	446.1815	211.5957	1.0763	2.75E-16	1.96E-14	Sh3pxd2b
ENSMUSG00000040714	32.0435	6.986982	2.1973	2.33E-05	0.000259	Klc3
ENSMUSG00000040717	177.4172	93.45363	0.92482	1.38E-07	2.68E-06	Il17rd
ENSMUSG00000040734	120.0113	80.53291	0.57552	0.003893	0.020917	Ppp1r13l
ENSMUSG00000040747	284.2612	181.7976	0.64488	1.05E-05	0.000129	Cd53
ENSMUSG00000040760	1016.751	793.7283	0.35725	0.000649	0.004678	Appl1
ENSMUSG00000040785	719.0374	588.5638	0.28887	0.008276	0.038982	Ttc3
ENSMUSG00000040809	65.22872	10.1442	2.6849	3.07E-14	1.76E-12	Chil3
ENSMUSG00000040824	927.9786	731.046	0.34413	0.002439	0.014167	Snrpd2
ENSMUSG00000040857	566.142	432.4258	0.38871	0.000739	0.005207	Erf
ENSMUSG00000040891	507.5291	312.272	0.70069	7.91E-05	0.000761	Foxa3
ENSMUSG00000040945	1592.097	1055.371	0.59318	7.85E-06	0.000101	Rcc2
ENSMUSG00000040952	4648.661	3608.739	0.36532	0.001888	0.011475	Rps19
ENSMUSG00000040964	674.3942	486.6078	0.47083	3.55E-05	0.000378	Arhgef10l
ENSMUSG00000040990	432.7129	302.2279	0.51777	0.000323	0.002567	Sh3kbp1
ENSMUSG00000040998	106.1593	44.01999	1.27	0.002303	0.013559	Npnt
ENSMUSG00000041025	343.0505	223.9794	0.61506	6.75E-06	8.73E-05	Iffo2
ENSMUSG00000041046	72.83053	12.68033	2.522	3.94E-11	1.47E-09	Ramp3
ENSMUSG00000041057	636.9092	428.479	0.57186	5.00E-07	8.58E-06	Wdr43
ENSMUSG00000041119	5861.906	2307.477	1.3451	2.45E-32	5.55E-30	Pde9a
ENSMUSG00000041134	98.48597	51.80527	0.92682	5.14E-05	0.000523	Cyyr1
ENSMUSG00000041189	115.3464	56.74412	1.0234	0.005295	0.026859	Chrnb1
ENSMUSG00000041202	56.06789	16.85192	1.7343	1.17E-07	2.32E-06	Pla2g2d
ENSMUSG00000041293	445.7896	131.1649	1.765	1.22E-34	2.98E-32	Adgrf1
ENSMUSG00000041324	95.38711	14.93119	2.6755	9.93E-12	4.14E-10	Inhba
ENSMUSG00000041347	27.97877	5.446188	2.361	2.05E-06	3.06E-05	Bdkrb1
ENSMUSG00000041360	560.5486	403.1378	0.47557	4.74E-05	0.000487	D19Bwg1357e
ENSMUSG00000041438	272.0209	173.2951	0.65049	9.03E-06	0.000113	Cirh1a
ENSMUSG00000041453	6285.133	5189.861	0.27625	0.001947	0.011794	Rpl21
ENSMUSG00000041476	100.4042	36.56973	1.4571	1.74E-09	5.06E-08	Smpx
ENSMUSG00000041483	176.0146	129.6184	0.44143	0.007624	0.03646	Zfp281
ENSMUSG00000041491	126.5557	76.59508	0.72445	0.000185	0.001605	Cep78
ENSMUSG00000041548	257.9919	158.9289	0.69894	4.99E-06	6.70E-05	Hspb8
ENSMUSG00000041570	349.7441	175.7421	0.99284	1.62E-12	7.60E-11	Camsap2
ENSMUSG00000041594	306.4912	234.7897	0.38448	0.007225	0.034867	Tmtc4
ENSMUSG00000041608	70.91891	20.8971	1.7629	0.005164	0.026315	Entpd3
ENSMUSG00000041609	169.7823	116.7039	0.54083	0.004491	0.023472	Ccdc64
ENSMUSG00000041695	121.1602	64.40903	0.91158	5.15E-06	6.88E-05	Kcnj2
ENSMUSG00000041736	1684.308	1102.516	0.61136	9.60E-06	0.000119	Tspo
ENSMUSG00000041801	145.8699	94.247	0.63016	0.000498	0.003725	Phlda3
ENSMUSG00000041836	238.7441	167.8998	0.50786	0.000579	0.004258	Ptpre
ENSMUSG00000041841	4006.218	3163.25	0.34083	0.000207	0.00176	Rpl37

ENSMUSG00000041842	221.91	153.4395	0.5323	0.001411	0.00895	Fhdc1
ENSMUSG00000041886	147.2327	68.44145	1.1052	2.91E-08	6.52E-07	Macc1
ENSMUSG00000041895	366.5978	257.7461	0.50825	0.000119	0.001092	Wipi1
ENSMUSG00000041920	168.6478	112.2015	0.58792	0.000739	0.005208	Slc16a6
ENSMUSG00000041930	94.49279	43.62662	1.115	3.02E-06	4.30E-05	Fam222a
ENSMUSG00000041957	725.5351	567.7804	0.35371	0.002037	0.012264	Pkp2
ENSMUSG00000041959	4379.737	3467.71	0.33686	0.007654	0.036583	S100a10
ENSMUSG00000041961	277.1235	139.1222	0.99418	5.34E-09	1.42E-07	Znrf3
ENSMUSG00000042029	459.0937	364.3987	0.33327	0.006125	0.030345	Ncapg2
ENSMUSG00000042042	200.097	130.5933	0.61562	0.000139	0.001248	Csgalnact2
ENSMUSG00000042043	894.1314	720.5334	0.31142	0.00284	0.01608	Tbca
ENSMUSG00000042105	233.087	168.9707	0.4641	0.002093	0.01255	Inpp5f
ENSMUSG00000042111	307.8751	194.5545	0.66217	3.06E-06	4.34E-05	Ccdc115
ENSMUSG00000042179	406.1983	39.0992	3.377	5.35E-10	1.67E-08	Pnliprp1
ENSMUSG00000042208	328.5572	199.5805	0.71917	8.46E-07	1.38E-05	0610010F05Rik
ENSMUSG00000042254	289.8536	203.7144	0.50878	0.000201	0.001717	Cilp
ENSMUSG00000042258	88.2026	36.45569	1.2747	8.57E-06	0.000108	Isl1
ENSMUSG00000042262	4.743514	0	Inf	0.008242	0.038872	Ccr8
ENSMUSG00000042265	66.57913	3.81039	4.1271	5.84E-22	7.24E-20	Trem1
ENSMUSG00000042268	30.80371	4.698008	2.713	5.44E-07	9.26E-06	Slc26a9
ENSMUSG00000042306	2554.512	995.9767	1.3589	9.01E-31	1.83E-28	S100a14
ENSMUSG00000042354	382.3103	304.1306	0.33005	0.00812	0.038407	Gnl3
ENSMUSG00000042429	80.39233	35.79013	1.1675	1.27E-05	0.000152	Adora1
ENSMUSG00000042444	505.4591	392.5624	0.36467	0.002583	0.014863	Fam63b
ENSMUSG00000042446	295.8682	222.2917	0.4125	0.004419	0.023142	Zmym4
ENSMUSG00000042447	253.5747	169.8463	0.57818	0.000146	0.001305	Mios
ENSMUSG00000042499	192.3	15.02014	3.6784	1.78E-43	7.95E-41	Hoxd11
ENSMUSG00000042506	1115.71	717.8598	0.63619	5.00E-07	8.58E-06	Usp22
ENSMUSG00000042541	1313.062	1048.909	0.32404	0.001075	0.007133	Shfm1
ENSMUSG00000042595	316.8131	229.8242	0.4631	0.000952	0.00644	Fam199x
ENSMUSG00000042622	307.6995	150.7322	1.0295	8.01E-10	2.42E-08	Maff
ENSMUSG00000042677	1116.028	666.6584	0.74335	4.79E-12	2.11E-10	Zc3h12a
ENSMUSG00000042699	2425.279	1720.172	0.4956	7.63E-07	1.26E-05	Dhx9
ENSMUSG00000042734	66.4685	14.05625	2.2415	1.87E-11	7.41E-10	Ttc9
ENSMUSG00000042742	312.2523	242.4587	0.36497	0.00723	0.034882	B630005N14Rik
ENSMUSG00000042745	1865.088	1411.126	0.4024	2.28E-05	0.000254	Id1
ENSMUSG00000042759	50.34593	28.06803	0.84295	0.005188	0.026425	Apobr
ENSMUSG00000042784	902.3394	201.6539	2.1618	1.09E-61	8.13E-59	Muc1
ENSMUSG00000042793	107.399	10.22046	3.3934	3.81E-06	5.28E-05	Lgr6
ENSMUSG00000042808	9549.452	6613.205	0.53007	7.84E-10	2.38E-08	Gpx2
ENSMUSG00000042810	271.728	158.4797	0.77787	2.51E-07	4.68E-06	Krba1
ENSMUSG00000042834	238.4053	119.6514	0.99458	0.003403	0.018641	Nrep
ENSMUSG00000042978	257.9602	188.8371	0.45001	0.009122	0.042228	Sbk1
ENSMUSG00000042988	609.7347	96.72624	2.6562	1.03E-06	1.64E-05	Notum
ENSMUSG00000043008	70.951	35.06103	1.017	0.000265	0.002164	Klhl6
ENSMUSG00000043068	93.40901	14.29705	2.7078	3.50E-07	6.23E-06	Fam89a
ENSMUSG00000043131	1726.636	1418.703	0.28339	0.002837	0.016069	Mob1a
ENSMUSG00000043252	747.3612	234.3828	1.6729	1.39E-39	5.07E-37	Tmem64
ENSMUSG00000043342	40.06278	3.25252	3.6226	7.78E-13	3.70E-11	Hoxd9
ENSMUSG00000043421	162.3908	63.36956	1.3576	1.55E-12	7.30E-11	Hilpda
ENSMUSG00000043430	32.60469	3.556871	3.1964	2.19E-09	6.26E-08	Psapl1
ENSMUSG00000043439	66.47545	28.35966	1.229	0.000815	0.005652	E130012A19Rik

ENSMUSG00000043613	158.384	16.27535	3.2827	2.43E-37	7.24E-35	Mmp3
ENSMUSG00000043621	79.25072	47.23436	0.74659	0.003074	0.017145	Ubxn10
ENSMUSG00000043629	298.6094	232.4995	0.36103	0.00938	0.043202	1700019D03Rik
ENSMUSG00000043639	86.14739	49.40293	0.80221	0.001306	0.008376	Rbm20
ENSMUSG00000043702	415.7168	321.4006	0.37123	0.003187	0.017666	Pde12
ENSMUSG00000043716	10997.63	7759.503	0.50316	5.68E-09	1.49E-07	Rpl7
ENSMUSG00000043740	16.22086	5.2435	1.6292	0.010799	0.048324	B430306N03Rik
ENSMUSG00000043760	75.67498	40.80555	0.89105	0.000819	0.005671	Pkhd1
ENSMUSG00000043924	59.16107	12.37598	2.2571	0.000624	0.00453	Ncmap
ENSMUSG00000044005	32.97643	14.19573	1.216	0.002962	0.016657	Gls2
ENSMUSG00000044037	335.0646	212.0411	0.6601	1.78E-06	2.70E-05	Als2cl
ENSMUSG00000044041	24.6311	5.021436	2.2943	3.43E-05	0.000367	Krt13
ENSMUSG00000044103	41.45862	18.37368	1.174	0.001396	0.008871	Il1f9
ENSMUSG00000044162	1025.854	497.9509	1.0428	2.09E-15	1.36E-13	Tnip3
ENSMUSG00000044244	42.60726	22.77403	0.90371	0.007672	0.03665	Il20rb
ENSMUSG00000044254	767.23	548.4936	0.48418	1.06E-05	0.00013	Pcsk9
ENSMUSG00000044294	42.39172	15.21044	1.4787	0.000214	0.001807	Krt84
ENSMUSG00000044303	16.31198	4.273256	1.9325	0.003301	0.018196	Cdkn2a
ENSMUSG00000044313	125.6627	15.10874	3.0561	0.000118	0.001079	Mab21l3
ENSMUSG00000044337	179.7007	103.1354	0.80106	4.33E-06	5.91E-05	Ackr3
ENSMUSG00000044442	180.7774	122.0977	0.56618	0.000395	0.003052	N6amt1
ENSMUSG00000044468	449.3066	339.3442	0.40495	0.001102	0.007261	Fam46c
ENSMUSG00000044502	436.16	351.4349	0.3116	0.009054	0.041989	Bod1
ENSMUSG00000044617	281.3255	210.1074	0.42111	0.003358	0.018444	Zbtb39
ENSMUSG00000044626	746.7155	481.384	0.63337	3.56E-08	7.86E-07	Liph
ENSMUSG00000044641	358.8667	258.5344	0.47309	0.000613	0.00446	Pard6b
ENSMUSG00000044674	583.5368	335.9639	0.79652	1.60E-11	6.40E-10	Fzd1
ENSMUSG00000044712	1489.761	1122.9	0.40785	0.000309	0.002465	Slc38a6
ENSMUSG00000044716	83.9816	42.14302	0.99478	0.003867	0.020833	Dok7
ENSMUSG00000044734	6867.055	4312.435	0.67119	4.81E-06	6.47E-05	Serpinb1a
ENSMUSG00000044864	790.2812	651.3554	0.27892	0.010331	0.046688	Ankrd50
ENSMUSG00000044921	43.84701	17.84756	1.2968	0.00021	0.001783	Rassf9
ENSMUSG00000044948	80.91477	52.0083	0.63766	0.005977	0.029716	Cfap43
ENSMUSG00000045027	126.5432	16.18005	2.9673	5.24E-10	1.64E-08	Prss22
ENSMUSG00000045045	510.6155	304.7982	0.74438	4.59E-05	0.000473	Lrfn4
ENSMUSG00000045062	22.29528	5.274956	2.0795	0.000135	0.001216	Pcdhb7
ENSMUSG00000045071	119.7903	82.69441	0.53465	0.011258	0.049968	E130308A19Rik
ENSMUSG00000045128	8906.825	6500.041	0.45446	1.98E-05	0.000224	Rpl18a
ENSMUSG00000045136	208.6907	63.12876	1.725	3.73E-08	8.21E-07	Tubb2b
ENSMUSG00000045165	79.67497	50.72764	0.65135	0.007207	0.03481	AI467606
ENSMUSG00000045193	739.3504	311.6385	1.2464	1.86E-15	1.22E-13	Cirbp
ENSMUSG00000045216	1497.273	1117.293	0.42233	3.95E-05	0.000415	Hs6st1
ENSMUSG00000045288	27.34556	3.448848	2.9871	0.00034	0.002684	Ush1g
ENSMUSG00000045349	17.1081	6.023096	1.5061	0.009607	0.043997	Sh2d5
ENSMUSG00000045382	145.0145	75.06125	0.95006	1.91E-06	2.87E-05	Cxcr4
ENSMUSG00000045411	601.0232	387.0935	0.63474	8.70E-08	1.78E-06	2410002F23Rik
ENSMUSG00000045502	31.18258	12.94021	1.2689	0.002539	0.014662	Hcar2
ENSMUSG00000045545	164.668	12.1479	3.7608	4.84E-07	8.34E-06	Krt14
ENSMUSG00000045551	26.75827	6.923432	1.9504	0.000129	0.001169	Fpr1
ENSMUSG00000045573	153.5423	95.02019	0.69233	0.000338	0.002677	Penk
ENSMUSG00000045624	389.2002	296.1299	0.39428	0.002207	0.01307	Esf1
ENSMUSG00000045629	62.52804	17.52418	1.8352	0.000744	0.00524	Sh3tc2

ENSMUSG00000045667	27.01945	9.351883	1.5307	0.001016	0.006804	Smtnl2
ENSMUSG00000045679	532.1513	351.2489	0.59934	0.000294	0.002365	Pqlc3
ENSMUSG00000045689	14.74603	3.949868	1.9005	0.003823	0.020636	Pcdhb4
ENSMUSG00000045761	14.68113	1.93379	2.9245	0.002337	0.013708	Fam179a
ENSMUSG00000045763	487.9479	157.0529	1.6355	8.04E-07	1.32E-05	Basp1
ENSMUSG00000045991	381.9784	47.19659	3.0167	0.000376	0.002931	Onecut2
ENSMUSG00000046008	11.00741	0	Inf	6.05E-06	7.95E-05	Pnlip
ENSMUSG00000046034	293.3045	219.376	0.41899	0.003531	0.019246	Otulin
ENSMUSG00000046191	17.18001	6.340165	1.4381	0.009749	0.044524	Pcdhb20
ENSMUSG00000046223	285.6128	53.60594	2.4136	1.88E-30	3.69E-28	Plaur
ENSMUSG00000046259	107.3664	4.951528	4.4385	1.77E-36	5.00E-34	Sprr2h
ENSMUSG00000046330	4625.448	3653.25	0.34041	0.006778	0.033148	Rpl37a
ENSMUSG00000046402	174.4678	34.96004	2.3192	3.31E-26	5.27E-24	Rbp1
ENSMUSG00000046432	342.3329	244.731	0.4842	0.002106	0.012602	Ngfrap1
ENSMUSG00000046434	3970.486	3097.179	0.35836	0.000774	0.005415	Hnrnpa1
ENSMUSG00000046441	130.242	90.4734	0.52563	0.005705	0.028614	Cmtr2
ENSMUSG00000046447	1790.893	1288.514	0.47497	3.07E-06	4.36E-05	Camk2n1
ENSMUSG00000046470	67.21221	39.86642	0.75355	0.004094	0.021707	Sox18
ENSMUSG00000046546	331.6907	181.5323	0.86961	1.45E-09	4.29E-08	Fam43a
ENSMUSG00000046623	33.094	7.1201	2.2166	2.34E-06	3.43E-05	Gjb4
ENSMUSG00000046658	101.9509	66.14078	0.62426	0.003497	0.019092	Zfp316
ENSMUSG00000046675	159.4606	109.0186	0.54863	0.001935	0.011727	Tmem251
ENSMUSG00000046688	1618.911	1036.066	0.64391	7.29E-11	2.62E-09	Tifa
ENSMUSG00000046714	23.6526	6.289673	1.9109	0.000406	0.003125	Foxc2
ENSMUSG00000046722	1276.651	1005.863	0.34393	0.000515	0.00384	Cdc42se1
ENSMUSG00000046733	2065.07	1418.709	0.54161	3.52E-07	6.26E-06	Gprc5a
ENSMUSG00000046761	1142.155	857.3889	0.41374	5.90E-05	0.00059	Fam83h
ENSMUSG00000046768	417.603	197.3499	1.0814	7.04E-16	4.87E-14	Rhoj
ENSMUSG00000046794	454.4291	355.3342	0.35488	0.003828	0.020649	Ppp1r3b
ENSMUSG00000046841	1198.085	808.0959	0.56813	1.42E-08	3.40E-07	Ckap4
ENSMUSG00000046873	326.3914	251.7719	0.37449	0.008498	0.039828	Mbtps2
ENSMUSG00000046971	304.0842	193.228	0.65417	2.87E-06	4.12E-05	Pla2g4f
ENSMUSG00000047109	116.6709	47.6213	1.2928	1.44E-08	3.44E-07	Cldn14
ENSMUSG00000047117	127.6258	36.74732	1.7962	1.16E-14	7.07E-13	Ankdd1b
ENSMUSG00000047139	5334.076	3431.122	0.63656	3.50E-11	1.32E-09	Cd24a
ENSMUSG00000047230	2136.572	1656.942	0.36678	7.20E-05	0.000697	Cldn2
ENSMUSG00000047264	169.6136	104.9871	0.69204	9.13E-05	0.000862	Zfp358
ENSMUSG00000047281	2026.703	1614.344	0.32819	0.000535	0.003969	Sfn
ENSMUSG00000047497	58.86689	27.2242	1.1126	0.000212	0.001799	Adamts12
ENSMUSG00000047501	2045.213	375.9294	2.4437	8.97E-07	1.45E-05	Cldn4
ENSMUSG00000047517	111660.9	74827.58	0.57748	5.06E-11	1.86E-09	Dmbt1
ENSMUSG00000047562	780.175	75.49774	3.3693	########	########	Mmp10
ENSMUSG00000047564	19.88771	5.00236	1.9912	0.000766	0.005371	Krtap3-1
ENSMUSG00000047586	75.9749	26.05797	1.5438	8.74E-08	1.78E-06	Nccrp1
ENSMUSG00000047714	1127.738	919.2764	0.29486	0.00436	0.022889	Ppp1r2
ENSMUSG00000047786	71.37514	21.61348	1.7235	6.20E-09	1.60E-07	Lix1
ENSMUSG00000047798	78.92439	27.00282	1.5474	2.75E-08	6.21E-07	Cd300lf
ENSMUSG00000047810	254.9648	118.429	1.1063	1.50E-05	0.000175	Ccdc88b
ENSMUSG00000047854	36.3172	13.69471	1.407	0.000453	0.003441	Stx19
ENSMUSG00000047881	622.228	475.2965	0.38862	0.000865	0.005933	Rell1
ENSMUSG00000047910	33.86387	12.95925	1.3858	0.000809	0.005618	Pcdhb16
ENSMUSG00000047945	3074.933	529.1567	2.5388	2.24E-42	9.52E-40	Marcksl1

ENSMUSG00000047989	424.2585	294.8751	0.52484	4.28E-05	0.000446	Ino80c
ENSMUSG00000048013	5.983284	0	Inf	0.001559	0.00976	Krt35
ENSMUSG00000048058	289.8462	188.4291	0.62127	1.50E-05	0.000176	Ldlrad3
ENSMUSG00000048078	575.5906	134.4683	2.0978	4.77E-06	6.42E-05	Tenm4
ENSMUSG00000048120	223.8793	158.6242	0.49711	0.002978	0.016732	Entpd1
ENSMUSG00000048232	89.1883	58.80497	0.60092	0.007049	0.03424	Fbxo10
ENSMUSG00000048234	1246.787	720.8672	0.79041	1.71E-10	5.82E-09	Rnf149
ENSMUSG00000048450	23.88755	7.982322	1.5814	0.001163	0.007608	Msx1
ENSMUSG00000048521	64.73952	23.31885	1.4732	4.98E-07	8.57E-06	Cxcr6
ENSMUSG00000048534	131.0325	56.70634	1.2083	0.000227	0.00191	Amica1
ENSMUSG00000048612	864.5994	583.9421	0.56621	2.66E-07	4.91E-06	Myof
ENSMUSG00000048644	164.7329	55.06453	1.5809	0.000951	0.006438	Ctxn1
ENSMUSG00000048696	227.3262	93.56233	1.2808	5.53E-05	0.000557	Mex3d
ENSMUSG00000048699	45.64792	11.80509	1.9511	6.62E-05	0.00065	4732456N10Rik
ENSMUSG00000048758	1693.037	1369.101	0.30638	0.001868	0.011386	Rpl29
ENSMUSG00000048911	143.671	94.05029	0.61126	0.0009	0.006134	Rnf24
ENSMUSG00000048960	249.6924	123.512	1.0155	1.83E-10	6.20E-09	Prex2
ENSMUSG00000049036	13.93062	3.556871	1.9696	0.004592	0.023912	Tmem121
ENSMUSG00000049086	122.8433	70.19833	0.80731	7.34E-05	0.00071	Bmyc
ENSMUSG00000049103	174.4998	98.20217	0.8294	0.000194	0.001673	Ccr2
ENSMUSG00000049225	248.9031	142.8308	0.80128	2.58E-07	4.78E-06	Pdp1
ENSMUSG00000049421	539.2895	407.2154	0.40527	0.000972	0.006557	Zfp260
ENSMUSG00000049470	1704.43	1298.789	0.39212	3.52E-05	0.000376	Aff4
ENSMUSG00000049517	3531.312	2695.774	0.3895	1.66E-05	0.000192	Rps23
ENSMUSG00000049690	76.17141	46.54337	0.71067	0.004057	0.021555	Nckap5
ENSMUSG00000049709	120.8007	84.45027	0.51645	0.00877	0.040868	Nlrp10
ENSMUSG00000049723	235.9384	72.73954	1.6976	2.84E-15	1.82E-13	Mmp12
ENSMUSG00000049751	2345.24	1984.91	0.24066	0.007994	0.037912	Rpl36al
ENSMUSG00000049775	7936.568	6175.141	0.36204	4.54E-05	0.000469	Tmsb4x
ENSMUSG00000049792	261.0522	188.4545	0.47012	0.00136	0.008681	Bag5
ENSMUSG00000049866	353.9272	97.84766	1.8548	3.73E-07	6.60E-06	Arl4c
ENSMUSG00000049878	415.7692	314.9844	0.4005	0.001453	0.009198	Rlf
ENSMUSG00000049939	30.28825	3.975264	2.9296	5.41E-08	1.16E-06	Lrrc4
ENSMUSG00000050071	47.14195	20.70043	1.1874	0.009219	0.042579	Bex1
ENSMUSG00000050092	70.5333	9.173953	2.9427	4.75E-10	1.50E-08	Sprr2b
ENSMUSG00000050164	14.56369	3.265237	2.1571	0.004355	0.022866	Mchr1
ENSMUSG00000050201	38.00744	1.521756	4.6425	4.36E-06	5.94E-05	Otop2
ENSMUSG00000050368	74.25962	13.52382	2.4571	1.15E-05	0.000139	Hoxd10
ENSMUSG00000050520	1444.902	686.1776	1.0743	2.10E-09	6.03E-08	Cldn8
ENSMUSG00000050530	279.7067	196.8686	0.50668	0.000486	0.003646	Fam171a1
ENSMUSG00000050578	1025.634	70.58433	3.861	########	########	Mmp13
ENSMUSG00000050635	4.978476	0	Inf	0.004751	0.024563	Sprr2f
ENSMUSG00000050640	48.075	13.58669	1.8231	2.67E-07	4.92E-06	Tmem150c
ENSMUSG00000050737	137.7718	96.49108	0.51381	0.008328	0.039171	Ptges
ENSMUSG00000050747	704.2007	295.8646	1.251	3.61E-25	5.50E-23	Trim15
ENSMUSG00000050762	44.19957	22.54595	0.97116	0.003261	0.01801	Prss27
ENSMUSG00000050912	890.1582	587.9771	0.5983	1.40E-08	3.35E-07	Tmem123
ENSMUSG00000050953	509.0559	335.4636	0.60167	4.82E-07	8.32E-06	Gja1
ENSMUSG00000050989	187.3671	115.9042	0.69293	4.69E-05	0.000482	Sepn1
ENSMUSG00000051022	198.7605	108.4483	0.87402	1.56E-07	3.01E-06	Hs3st1
ENSMUSG00000051043	52.26422	18.32951	1.5117	0.002379	0.013907	Gprc5c
ENSMUSG00000051076	103.7324	14.60772	2.8281	3.13E-07	5.66E-06	Vtcn1

ENSMUSG00000051154	481.9518	385.1448	0.32349	0.004008	0.021382	Commd3
ENSMUSG00000051159	17.50619	3.689989	2.2462	0.000389	0.003014	Cited1
ENSMUSG00000051185	234.7183	158.2814	0.56844	0.000151	0.00134	Fam174a
ENSMUSG00000051212	37.72006	20.19945	0.90102	0.008832	0.041081	Gpr183
ENSMUSG00000051223	3852.263	2845.855	0.43684	0.000561	0.004145	Bzw1
ENSMUSG00000051236	367.5957	276.3479	0.41163	0.001869	0.011386	Msrb3
ENSMUSG00000051242	35.08425	9.478643	1.8881	6.40E-05	0.000632	Pcdhb9
ENSMUSG00000051343	199.0143	101.3604	0.97338	0.000158	0.001391	Rab11fip5
ENSMUSG00000051397	280.6999	32.98776	3.089	2.02E-05	0.000228	Tacstd2
ENSMUSG00000051439	1412.243	711.9046	0.98823	3.28E-22	4.12E-20	Cd14
ENSMUSG00000051457	95.54352	64.43473	0.56832	0.008679	0.040485	Spn
ENSMUSG00000051497	45.08006	1.325088	5.0883	1.79E-16	1.30E-14	Kcnj16
ENSMUSG00000051596	38.86844	9.364601	2.0533	0.004536	0.023669	Otop1
ENSMUSG00000051748	23.48953	0.304351	6.2701	4.50E-11	1.66E-09	Wfdc21
ENSMUSG00000051790	255.533	165.845	0.62367	0.002714	0.015478	Nlgn2
ENSMUSG00000052013	79.6682	43.70891	0.86608	0.000745	0.005246	Btla
ENSMUSG00000052105	123.4764	69.96358	0.81956	3.54E-05	0.000377	Mtcl1
ENSMUSG00000052144	948.0262	723.5771	0.38978	0.000204	0.001738	Ppp4r2
ENSMUSG00000052221	9.839457	0.906695	3.4399	0.001221	0.007911	Ppp1r36
ENSMUSG00000052270	34.21634	9.960246	1.7804	2.03E-05	0.000229	Fpr2
ENSMUSG00000052302	249.9727	168.0781	0.57264	0.000244	0.002024	Tbc1d30
ENSMUSG00000052353	123.822	29.93191	2.0485	3.32E-17	2.68E-15	Cemip
ENSMUSG00000052373	112.7496	77.6285	0.53846	0.004381	0.022977	Mpp3
ENSMUSG00000052429	895.0986	538.799	0.7323	9.95E-12	4.14E-10	Prmt1
ENSMUSG00000052512	531.4688	286.1315	0.89331	0.000552	0.004089	Nav2
ENSMUSG00000052516	612.9103	369.978	0.72824	1.33E-07	2.59E-06	Robo2
ENSMUSG00000052681	1840.628	1237.301	0.573	2.77E-08	6.24E-07	Rap1b
ENSMUSG00000052684	1534.639	1148.801	0.41777	0.002886	0.016278	Jun
ENSMUSG00000052819	538.3518	410.0549	0.39273	0.000694	0.00494	Best2
ENSMUSG00000052837	1402.185	979.8204	0.51709	2.56E-06	3.73E-05	Junb
ENSMUSG00000052911	433.0802	341.5141	0.34269	0.005555	0.027964	Lamb2
ENSMUSG00000052914	804.4861	596.234	0.43219	0.001364	0.00869	Cyp2j6
ENSMUSG00000052949	81.41098	50.18885	0.69786	0.004885	0.025138	Rnf157
ENSMUSG00000052997	1023.735	781.6717	0.38921	0.000139	0.001245	Uba2
ENSMUSG00000053113	1364.367	499.9626	1.4483	3.33E-40	1.26E-37	Socs3
ENSMUSG00000053141	129.5247	77.82614	0.7349	0.000338	0.002672	Ptprt
ENSMUSG00000053158	210.8637	158.0907	0.41556	0.003898	0.02093	Fes
ENSMUSG00000053175	583.5694	452.2969	0.36763	0.001727	0.010669	Bcl3
ENSMUSG00000053289	382.1788	261.1879	0.54916	2.77E-05	0.000303	Ddx10
ENSMUSG00000053338	13.93061	2.231783	2.642	0.000578	0.004249	Tarm1
ENSMUSG00000053398	535.4995	415.1422	0.36728	0.002575	0.014824	Phgdh
ENSMUSG00000053414	266.4821	132.1231	1.0122	0.004005	0.021375	Hunk
ENSMUSG00000053470	758.173	530.2578	0.51583	2.79E-06	4.01E-05	Kdm3a
ENSMUSG00000053477	1593.473	777.2869	1.0357	0.000217	0.001829	Tcf4
ENSMUSG00000053617	525.087	358.5858	0.55024	2.65E-06	3.84E-05	Sh3pxd2a
ENSMUSG00000053646	366.2726	190.2933	0.94469	0.000652	0.004695	Plxnb1
ENSMUSG00000053675	30.00766	10.63886	1.496	0.000794	0.005527	Tgm5
ENSMUSG00000053684	88.39207	54.93133	0.68629	0.003846	0.020724	BC048403
ENSMUSG00000053687	34.11814	15.29239	1.1577	0.004179	0.022097	Dpep2
ENSMUSG00000053702	235.6907	60.56053	1.9604	4.12E-06	5.66E-05	Nebl
ENSMUSG00000053716	192.3265	135.5268	0.50498	0.001387	0.008822	Dusp7
ENSMUSG00000053746	131.1434	86.8409	0.5947	0.001211	0.00786	Ptrh1

ENSMUSG00000053797	7.829814	0.297992	4.7156	0.000963	0.0065	Krt16
ENSMUSG00000053799	392.3851	292.8138	0.42229	0.000829	0.005732	Exoc6
ENSMUSG00000053897	677.0121	394.0779	0.7807	3.98E-12	1.78E-10	Slc39a8
ENSMUSG00000054008	937.3458	628.3496	0.57701	9.09E-08	1.84E-06	Ndst1
ENSMUSG00000054079	248.1719	180.9915	0.45542	0.002145	0.012768	Utp18
ENSMUSG00000054106	4.978494	0	Inf	0.004751	0.024563	Try4
ENSMUSG00000054146	35.41039	5.319429	2.7348	7.31E-06	9.39E-05	Krt15
ENSMUSG00000054169	2120.588	1285.326	0.72233	1.12E-13	5.89E-12	Ceacam10
ENSMUSG00000054263	159.2712	111.9919	0.50809	0.003537	0.019273	Lifr
ENSMUSG00000054321	137.7264	77.77467	0.82443	2.32E-05	0.000258	Taf4b
ENSMUSG00000054364	2651.443	2097.194	0.33832	0.00019	0.001644	Rhob
ENSMUSG00000054405	860.0257	694.092	0.30925	0.002648	0.015178	Dnajc8
ENSMUSG00000054423	121.9559	37.71756	1.6931	5.56E-09	1.46E-07	Cadps
ENSMUSG00000054446	16.05775	0	Inf	1.27E-08	3.07E-07	Cpa1
ENSMUSG00000054555	121.6105	20.51682	2.5674	4.21E-16	2.97E-14	Adam12
ENSMUSG00000054693	2174.168	1642.153	0.40488	8.67E-06	0.000109	Adam10
ENSMUSG00000054793	121.8839	50.62666	1.2675	0.000244	0.002022	Cadm4
ENSMUSG00000054814	318.5881	219.6806	0.53628	0.000221	0.001859	Usp46
ENSMUSG00000054958	45.15187	18.27901	1.3046	0.000403	0.003113	Nt5c1a
ENSMUSG00000055170	28.86604	5.179951	2.4784	1.29E-06	2.02E-05	Ifng
ENSMUSG00000055193	74.40319	6.68263	3.4769	0.001125	0.007393	Klk15
ENSMUSG00000055320	666.3116	458.4582	0.53941	1.42E-06	2.20E-05	Tead1
ENSMUSG00000055333	40.38891	8.039513	2.3288	5.87E-08	1.24E-06	Fat2
ENSMUSG00000055373	274.3622	125.3246	1.1304	8.34E-11	2.97E-09	Fut9
ENSMUSG00000055447	1259.245	810.7188	0.63529	2.19E-09	6.26E-08	Cd47
ENSMUSG00000055629	197.5662	83.43691	1.2436	2.74E-12	1.23E-10	B4galnt4
ENSMUSG00000055639	141.8436	61.58792	1.2036	1.76E-09	5.10E-08	Dach1
ENSMUSG00000055723	580.0713	409.5479	0.5022	1.34E-05	0.000159	Rras2
ENSMUSG00000055835	90.8191	57.0929	0.66968	0.003683	0.019955	Zfp1
ENSMUSG00000055926	174.859	90.7145	0.94679	1.65E-07	3.16E-06	Gm14137
ENSMUSG00000055945	94.19281	19.15395	2.298	8.84E-07	1.44E-05	Prr18
ENSMUSG00000055978	3284.689	2600.279	0.33709	0.00022	0.001855	Fut2
ENSMUSG00000056054	3425.568	65.88632	5.7002	0	0	S100a8
ENSMUSG00000056071	2305.465	41.33731	5.8015	########	########	S100a9
ENSMUSG00000056124	682.343	508.3153	0.42477	0.006832	0.033382	B4galt6
ENSMUSG00000056153	572.4045	471.5846	0.27952	0.009798	0.044685	Socs6
ENSMUSG00000056201	7403.229	5984.279	0.30698	0.0003	0.002412	Cfl1
ENSMUSG00000056214	98.94876	51.66579	0.93747	8.31E-05	0.000794	Pard6g
ENSMUSG00000056487	17.78673	2.180951	3.0278	3.22E-05	0.000346	Mettl7a2
ENSMUSG00000056501	238.2883	153.3071	0.63628	0.00668	0.032745	Cebpb
ENSMUSG00000056515	415.2207	193.9762	1.098	1.11E-15	7.57E-14	Rab31
ENSMUSG00000056537	1023.084	772.2508	0.40578	4.30E-05	0.000447	Rlim
ENSMUSG00000056612	1186.559	779.2096	0.6067	0.000548	0.004062	Ppp1r14b
ENSMUSG00000056632	27.18262	1.020737	4.735	3.55E-11	1.34E-09	Dsg3
ENSMUSG00000056737	1316.738	821.9332	0.67988	3.35E-11	1.27E-09	Capg
ENSMUSG00000056832	46.88083	20.84626	1.1692	0.000454	0.003448	Ttc26
ENSMUSG00000056856	31.34555	5.984982	2.3888	0.000143	0.001281	Jakmip3
ENSMUSG00000057092	237.6674	151.8968	0.64585	0.00042	0.003224	Fxyd3
ENSMUSG00000057113	4369.834	3120.634	0.48574	3.13E-08	6.99E-07	Npm1
ENSMUSG00000057163	13.7219	0.722744	4.2469	6.10E-05	0.000606	Prss2
ENSMUSG00000057181	186.6099	131.735	0.50239	0.003333	0.018345	5730455P16Rik
ENSMUSG00000057191	49.03428	21.78471	1.1705	0.000308	0.00246	AB124611

ENSMUSG00000057193	504.9253	366.2784	0.46313	0.000207	0.001763	Slc44a2
ENSMUSG00000057236	1447.558	1190.818	0.28167	0.002977	0.01673	Rbbp4
ENSMUSG00000057363	428.2779	313.9453	0.44803	0.000367	0.002872	Uxs1
ENSMUSG00000057421	426.2802	340.5485	0.32394	0.010756	0.048159	Las1l
ENSMUSG00000057561	1072.42	818.5576	0.38971	0.001088	0.007195	Eif1a
ENSMUSG00000057604	42.84219	18.00006	1.251	0.004444	0.023258	Lmcd1
ENSMUSG00000057666	11415.45	9787.331	0.222	0.009898	0.045076	Gapdh
ENSMUSG00000057706	68.34124	30.68611	1.1552	0.001511	0.009498	Mex3b
ENSMUSG00000057719	150.5927	91.09576	0.7252	0.000304	0.002441	Sh3rf2
ENSMUSG00000057841	6558.536	4954.226	0.40471	5.04E-06	6.75E-05	Rpl32
ENSMUSG00000057858	248.5508	155.1121	0.68023	1.34E-05	0.000159	Fam204a
ENSMUSG00000057863	1899.322	1520.884	0.32058	0.000723	0.005119	Rpl36
ENSMUSG00000058057	101.4352	13.16193	2.9461	2.38E-12	1.08E-10	Mettl7a3
ENSMUSG00000058145	45.17806	16.97296	1.4124	0.000141	0.001262	Adamts17
ENSMUSG00000058239	588.5217	463.6622	0.34402	0.00194	0.011751	Usf2
ENSMUSG00000058291	372.9919	272.2141	0.4544	0.000664	0.00477	Zfp68
ENSMUSG00000058297	3620.142	526.0809	2.7827	1.73E-09	5.05E-08	Spock2
ENSMUSG00000058317	185.4296	96.81417	0.93758	1.11E-07	2.19E-06	Ube2e2
ENSMUSG00000058396	51.62427	25.13224	1.0385	0.001027	0.006861	Gpr182
ENSMUSG00000058427	290.6042	16.7506	4.1168	2.19E-07	4.10E-06	Cxcl2
ENSMUSG00000058546	1794.385	1222.793	0.55331	0.000186	0.00161	Rpl23a
ENSMUSG00000058558	11545.35	7912.391	0.54513	2.42E-10	8.05E-09	Rpl5
ENSMUSG00000058569	1934.692	1589.514	0.28352	0.00383	0.020649	Tmed9
ENSMUSG00000058579	7.712374	0	Inf	0.000238	0.001986	Cela2a
ENSMUSG00000058587	2018.893	1221.284	0.72517	9.71E-14	5.14E-12	Tmod3
ENSMUSG00000058600	5009.771	3673.575	0.44756	4.74E-07	8.21E-06	Rpl30
ENSMUSG00000058626	9.115202	0.304351	4.9045	0.000253	0.002085	Capn11
ENSMUSG00000058704	428.8841	284.0572	0.59441	2.74E-06	3.94E-05	Memo1
ENSMUSG00000058729	146.9258	102.1396	0.52455	0.006273	0.031011	Lin9
ENSMUSG00000058755	81.46886	3.620081	4.4922	9.03E-29	1.61E-26	Osm
ENSMUSG00000058799	1648.028	1265.685	0.38082	0.000795	0.005534	Nap1l1
ENSMUSG00000058818	96.05186	58.85508	0.70665	0.001905	0.011563	Pirb
ENSMUSG00000058908	1403.97	423.4783	1.7292	6.21E-36	1.61E-33	Pla2g2a
ENSMUSG00000058952	165.6272	15.81245	3.3888	9.10E-07	1.47E-05	Cfi
ENSMUSG00000059005	4845.407	4046.433	0.25997	0.002754	0.015672	Hnrnpa3
ENSMUSG00000059070	7835.124	6202.27	0.33716	8.40E-05	0.000802	Rpl18
ENSMUSG00000059108	50.01283	5.433471	3.2024	1.40E-13	7.24E-12	Ifitm6
ENSMUSG00000059119	1151.145	937.7125	0.29585	0.002389	0.013959	Nap1l4
ENSMUSG00000059149	2318.045	1333.836	0.79733	6.62E-17	5.16E-15	Mfsd4
ENSMUSG00000059182	1571.96	864.325	0.86292	3.87E-18	3.47E-16	Skap2
ENSMUSG00000059248	1054.786	861.2101	0.29251	0.004919	0.025291	9-Sep
ENSMUSG00000059288	183.6289	102.1526	0.84607	1.70E-06	2.59E-05	Cdyl
ENSMUSG00000059291	4220.245	3119.118	0.43619	4.20E-06	5.74E-05	Rpl11
ENSMUSG00000059325	557.2089	333.3205	0.74131	6.44E-08	1.36E-06	Hopx
ENSMUSG00000059401	59.68914	27.78245	1.1033	0.000142	0.001271	Mamld1
ENSMUSG00000059474	520.9694	352.6654	0.5629	2.99E-06	4.27E-05	Mbtd1
ENSMUSG00000059498	180.3793	117.324	0.62053	0.000194	0.001671	Fcgr3
ENSMUSG00000059654	28.74856	9.453586	1.6046	0.000595	0.004352	Reg1
ENSMUSG00000059657	13.36944	1.033455	3.6934	3.60E-05	0.000382	Stfa2l1
ENSMUSG00000059713	360.2029	282.7131	0.34947	0.010393	0.046944	Rcan3
ENSMUSG00000059796	7586.536	5215.597	0.54061	5.83E-10	1.82E-08	Eif4a1
ENSMUSG00000059854	18.62848	4.710726	1.9835	0.000699	0.004969	Hydin

ENSMUSG00000059898	17.59743	3.512398	2.3248	0.000305	0.002442	Dsc3
ENSMUSG00000059900	36.48021	13.33986	1.4514	0.00046	0.003482	Tmem40
ENSMUSG00000059970	127.3452	89.94763	0.50159	0.010897	0.048683	Hspa2
ENSMUSG00000060036	12524.44	9290.674	0.43089	4.55E-07	7.92E-06	Rpl3
ENSMUSG00000060063	98.45951	48.91425	1.0093	2.31E-05	0.000257	Alox5ap
ENSMUSG00000060073	1850.93	1479.701	0.32294	0.0006	0.004387	Psma3
ENSMUSG00000060090	821.7384	540.7876	0.60362	9.12E-08	1.85E-06	Rp2h
ENSMUSG00000060126	21239.38	14976.97	0.504	5.26E-09	1.40E-07	Tpt1
ENSMUSG00000060227	269.4432	196.3602	0.45648	0.001289	0.008286	Casc4
ENSMUSG00000060260	175.4339	127.7989	0.45705	0.005853	0.029177	Pwwp2b
ENSMUSG00000060402	19.7701	6.93615	1.5111	0.00788	0.03747	Chst8
ENSMUSG00000060427	180.3986	125.946	0.51838	0.001912	0.011603	Zfp868
ENSMUSG00000060487	95.36087	14.15795	2.7518	7.10E-18	6.15E-16	Samd5
ENSMUSG00000060510	563.8575	375.9213	0.5849	8.36E-07	1.37E-05	Zfp266
ENSMUSG00000060512	949.0773	756.0512	0.32804	0.001636	0.010183	0610040J01Rik
ENSMUSG00000060548	149.0402	46.81593	1.6706	5.68E-07	9.67E-06	Tnfrsf19
ENSMUSG00000060591	1662.447	919.5085	0.85437	1.34E-05	0.000159	Ifitm2
ENSMUSG00000060615	302.1001	170.5504	0.82483	1.22E-07	2.39E-06	Ang4
ENSMUSG00000060636	4439.361	3537.396	0.32766	0.000209	0.001778	Rpl35a
ENSMUSG00000060739	2049.001	1459.153	0.48979	2.82E-07	5.17E-06	Nsa2
ENSMUSG00000060743	5454.343	3950.181	0.46549	1.08E-07	2.14E-06	H3f3a
ENSMUSG00000060791	255.3233	183.4464	0.47697	0.002062	0.012392	Gmfg
ENSMUSG00000060938	5958.382	4317.1	0.46486	9.09E-08	1.84E-06	Rpl26
ENSMUSG00000061024	321.6619	246.6318	0.38319	0.003281	0.018101	Rrs1
ENSMUSG00000061136	1567.284	1208.74	0.37476	8.25E-05	0.000789	Prpf40a
ENSMUSG00000061244	1000.706	607.5433	0.71996	2.84E-08	6.37E-07	Exoc5
ENSMUSG00000061315	4211.326	3430.542	0.29584	0.000816	0.005652	Naca
ENSMUSG00000061458	132.3497	90.46704	0.54889	0.002926	0.016467	Nol10
ENSMUSG00000061477	6048.436	4174.046	0.53512	9.13E-10	2.75E-08	Rps7
ENSMUSG00000061517	273.907	92.66835	1.5635	8.84E-06	0.000111	Sox21
ENSMUSG00000061731	639.2076	415.8401	0.62026	3.21E-07	5.79E-06	Ext1
ENSMUSG00000061762	1505.158	269.4424	2.4819	7.61E-23	1.02E-20	Tac1
ENSMUSG00000061787	5275.703	3992.063	0.40223	4.75E-06	6.40E-05	Rps17
ENSMUSG00000061808	288.8678	111.1045	1.3785	3.00E-18	2.74E-16	Ttr
ENSMUSG00000061947	26.06031	11.03817	1.2394	0.005335	0.027027	Serpina10
ENSMUSG00000061981	712.3036	330.3221	1.1086	3.89E-21	4.42E-19	Flot2
ENSMUSG00000061983	4411.779	3431.431	0.36255	5.22E-05	0.00053	Rps12
ENSMUSG00000062006	4079.609	2943.937	0.47068	1.61E-07	3.09E-06	Rpl34
ENSMUSG00000062014	1105.338	893.5927	0.3068	0.003528	0.01924	Gmfb
ENSMUSG00000062070	3177.839	2075.048	0.6149	3.60E-11	1.35E-09	Pgk1
ENSMUSG00000062124	109.9108	8.578307	3.6795	1.34E-30	2.66E-28	Defb45
ENSMUSG00000062168	40.08213	6.638158	2.5941	5.10E-09	1.36E-07	Ppef1
ENSMUSG00000062175	102.4402	60.51068	0.75952	0.000492	0.003684	Tgif2
ENSMUSG00000062210	648.8286	492.6021	0.39741	0.000601	0.00439	Tnfaip8
ENSMUSG00000062327	90.64341	15.10208	2.5855	4.81E-05	0.000494	T
ENSMUSG00000062328	6847.772	5178.661	0.40306	4.05E-06	5.58E-05	Rpl17
ENSMUSG00000062373	332.636	256.7829	0.3734	0.006917	0.033721	Tmem65
ENSMUSG00000062400	9.206475	0	Inf	6.33E-05	0.000625	Krtap6-5
ENSMUSG00000062421	523.5197	233.0825	1.1674	1.23E-19	1.24E-17	Arf2
ENSMUSG00000062591	154.5412	56.11702	1.4615	4.99E-05	0.000511	Tubb4a
ENSMUSG00000062593	86.01069	39.70189	1.1153	3.90E-06	5.39E-05	Lilrb4a
ENSMUSG00000062661	88.08518	57.13102	0.62463	0.007582	0.036278	Ncs1

ENSMUSG00000062826	90.79992	36.04331	1.333	4.77E-08	1.03E-06	Ces2f
ENSMUSG00000062901	856.3838	610.2052	0.48896	5.61E-06	7.43E-05	Klhl24
ENSMUSG00000062937	217.3362	122.3769	0.8286	2.61E-06	3.79E-05	Mtap
ENSMUSG00000062963	991.0056	788.9279	0.329	0.002224	0.013143	Ufc1
ENSMUSG00000062997	1736.245	1188.905	0.54634	1.31E-08	3.17E-07	Rpl35
ENSMUSG00000063077	1245.173	1033.53	0.26877	0.009771	0.0446	Kif1b
ENSMUSG00000063316	1372.267	1113.782	0.30109	0.00262	0.015052	Rpl27
ENSMUSG00000063334	361.071	278.466	0.37478	0.00603	0.029943	Krr1
ENSMUSG00000063406	591.3192	448.7214	0.39812	0.001835	0.011233	Tmed5
ENSMUSG00000063410	1049.423	828.2618	0.34144	0.001505	0.009463	Stk24
ENSMUSG00000063522	4792.235	3581.58	0.4201	0.008814	0.041029	2010109I03Rik
ENSMUSG00000063531	121.2056	29.08843	2.0589	2.14E-17	1.78E-15	Sema3e
ENSMUSG00000063632	19.18963	3.677272	2.3836	0.0001	0.000934	Sox11
ENSMUSG00000063727	778.3615	96.26236	3.0154	3.39E-13	1.70E-11	Tnfrsf11b
ENSMUSG00000063785	361.8286	249.1433	0.53833	9.58E-05	0.000899	Utp14a
ENSMUSG00000063851	33.25711	10.74052	1.6306	0.000248	0.002048	Rnf183
ENSMUSG00000063889	108.0255	68.51241	0.65693	0.00221	0.013075	Crem
ENSMUSG00000064080	241.4575	175.9199	0.45685	0.003895	0.020917	Fbln2
ENSMUSG00000064147	24.28559	4.051531	2.5836	8.54E-06	0.000108	Rab44
ENSMUSG00000064246	31.71043	7.703746	2.0413	0.002016	0.012153	Chil1
ENSMUSG00000064264	58.41069	32.93091	0.82679	0.003069	0.017131	Zfp428
ENSMUSG00000064360	7375.843	5556.388	0.40866	5.10E-06	6.82E-05	mt-Nd3
ENSMUSG00000066148	339.4157	239.9183	0.50051	0.000292	0.002356	Prpf4
ENSMUSG00000066150	2445.105	1733.01	0.49662	8.22E-08	1.69E-06	Slc31a1
ENSMUSG00000066357	419.658	275.3289	0.60806	0.000197	0.001694	Wdr6
ENSMUSG00000066551	5282.101	4274.511	0.30535	0.000367	0.002869	Hmgb1
ENSMUSG00000066643	73.13064	46.28955	0.65979	0.007562	0.036199	Wdr35
ENSMUSG00000066705	656.2304	293.4295	1.1612	6.87E-05	0.00067	Fxyd6
ENSMUSG00000067006	1068.132	649.7276	0.71718	6.13E-05	0.000608	Serpinb5
ENSMUSG00000067071	1192.425	618.3576	0.94739	0.001253	0.008091	Hes6
ENSMUSG00000067274	15233.32	11469.77	0.40939	1.92E-06	2.88E-05	Rplp0
ENSMUSG00000067336	908.6491	657.7175	0.46626	5.75E-05	0.000576	Bmpr2
ENSMUSG00000067365	401.3423	311.8805	0.36384	0.008492	0.039828	Tmem128
ENSMUSG00000067750	38.8879	6.333806	2.6182	9.72E-09	2.40E-07	Khdc1a
ENSMUSG00000067825	369.4298	257.3614	0.5215	0.000157	0.001388	Pex26
ENSMUSG00000067889	191.7652	64.63847	1.5689	1.80E-16	1.31E-14	Sptbn2
ENSMUSG00000067995	306.714	228.5249	0.42454	0.001318	0.008439	Gtf2f2
ENSMUSG00000068011	784.0392	592.124	0.40503	0.000341	0.002689	Mkrn2os
ENSMUSG00000068101	111.5554	77.10272	0.53291	0.007742	0.036932	Cenpm
ENSMUSG00000068341	15.98591	1.331447	3.5857	1.12E-05	0.000137	Reg3d
ENSMUSG00000068349	55.55961	20.78301	1.4186	0.005683	0.028528	Gml
ENSMUSG00000068391	244.4926	176.7122	0.46839	0.001143	0.007493	Chrac1
ENSMUSG00000068739	1168.059	947.7923	0.30147	0.002642	0.015158	Sars
ENSMUSG00000068748	376.9467	113.2407	1.735	2.14E-06	3.17E-05	Ptprz1
ENSMUSG00000068758	74.32478	42.04139	0.82203	0.000645	0.004656	Il3ra
ENSMUSG00000068823	3862.127	3191.604	0.27511	0.002212	0.013087	Csde1
ENSMUSG00000068882	1520.866	1108.694	0.45603	2.47E-06	3.60E-05	Ssb
ENSMUSG00000068923	228.5327	134.8485	0.76106	0.003069	0.017131	Syt11
ENSMUSG00000069089	337.066	258.1405	0.38487	0.008193	0.038672	Cdk7
ENSMUSG00000069515	2791.482	524.5153	2.412	4.21E-08	9.22E-07	Lyz1
ENSMUSG00000069516	2642.806	1550.375	0.76945	9.66E-16	6.60E-14	Lyz2
ENSMUSG00000069744	1224.677	957.7803	0.35463	0.000309	0.002465	Psmb3

ENSMUSG00000069769	954.7307	646.0937	0.56335	4.00E-08	8.79E-07	Msi2
ENSMUSG00000069792	262.853	76.90571	1.7731	5.38E-25	8.13E-23	Wfdc17
ENSMUSG00000069833	11155.83	8617.549	0.37245	1.36E-05	0.000161	Ahnak
ENSMUSG00000070000	98.39474	62.73008	0.64942	0.004264	0.022454	Fcho1
ENSMUSG00000070304	183.7929	62.32407	1.5602	0.001047	0.00698	Scn2b
ENSMUSG00000070305	609.9034	443.5317	0.45954	4.19E-05	0.000437	Mpzl3
ENSMUSG00000070594	7.66672	0.602344	3.67	0.003124	0.017369	Gm4788
ENSMUSG00000070697	458.5396	359.9637	0.3492	0.005889	0.029343	Utp3
ENSMUSG00000070934	649.8597	489.4023	0.40911	0.000445	0.003388	Rraga
ENSMUSG00000071019	11.84912	1.020737	3.5371	0.000195	0.001678	Sdr16c6
ENSMUSG00000071356	83443.67	8982.678	3.2156	2.94E-42	1.22E-39	Reg3b
ENSMUSG00000071362	49.83032	27.7446	0.84482	0.006006	0.029851	Gm10330
ENSMUSG00000071415	8732.265	6287.263	0.47392	5.71E-08	1.22E-06	Rpl23
ENSMUSG00000071708	344.7851	231.0921	0.57723	3.08E-05	0.000333	Sms
ENSMUSG00000071713	194.7411	104.156	0.90281	8.06E-08	1.66E-06	Csf2rb
ENSMUSG00000071714	88.18342	56.22365	0.64933	0.004172	0.022083	Csf2rb2
ENSMUSG00000071847	449.5621	43.06875	3.3838	1.28E-34	3.09E-32	Apcdd1
ENSMUSG00000071855	67.70836	38.84598	0.80157	0.002291	0.013496	Ccdc112
ENSMUSG00000071866	12894.88	9650.3	0.41815	9.21E-07	1.49E-05	Ppia
ENSMUSG00000072235	521.193	330.3293	0.65791	0.002127	0.012692	Tuba1a
ENSMUSG00000072437	86.80674	21.31583	2.0259	4.93E-10	1.55E-08	Nanos1
ENSMUSG00000072571	203.0009	147.0104	0.46557	0.006449	0.031731	Tmem253
ENSMUSG00000072582	236.6433	165.6118	0.51491	0.000811	0.005627	Ptrh2
ENSMUSG00000072620	270.7354	146.8837	0.88221	1.48E-08	3.52E-07	Slfn2
ENSMUSG00000072941	310.3988	229.2096	0.43746	0.002764	0.015715	Sod3
ENSMUSG00000073000	58.24757	22.964	1.3428	0.000169	0.001479	Gm10451
ENSMUSG00000073197	87.64164	58.61436	0.58036	0.008835	0.041081	5730507C01Rik
ENSMUSG00000073399	922.1428	524.5765	0.81384	5.55E-12	2.39E-10	Trim40
ENSMUSG00000073400	52.53795	7.836486	2.7451	7.64E-12	3.24E-10	Trim10
ENSMUSG00000073489	45.08688	24.65729	0.87069	0.009483	0.043552	Ifi204
ENSMUSG00000073599	322.7519	113.8691	1.503	4.44E-07	7.74E-06	Ecscr
ENSMUSG00000073639	1490.499	1208.963	0.30203	0.006996	0.03402	Rab18
ENSMUSG00000073678	592.6312	383.1667	0.62916	1.36E-07	2.65E-06	Pgap1
ENSMUSG00000073702	7346.744	5409.245	0.44168	0.000541	0.004008	Rpl31
ENSMUSG00000074115	14432.66	11590.39	0.31641	0.000147	0.001312	Saa1
ENSMUSG00000074129	15344.91	10406.66	0.56025	3.35E-11	1.27E-09	Rpl13a
ENSMUSG00000074182	122.8365	84.95761	0.53192	0.006517	0.032003	Znhit6
ENSMUSG00000074195	34503.11	5513.922	2.6456	1.13E-35	2.81E-33	Clca4b
ENSMUSG00000074457	2985.914	1625.172	0.87758	1.42E-21	1.69E-19	S100a16
ENSMUSG00000074480	338.2217	80.69771	2.0674	1.94E-05	0.00022	Mex3a
ENSMUSG00000074656	7384.879	5671.508	0.38084	0.0002	0.001711	Eif2s2
ENSMUSG00000074766	42.41795	15.02616	1.4972	5.92E-05	0.000591	Ism1
ENSMUSG00000074796	35.0124	11.05123	1.6637	9.41E-05	0.000886	Slc4a11
ENSMUSG00000074797	550.9176	412.085	0.41889	0.000474	0.003569	Itpa
ENSMUSG00000074825	84.84282	57.14378	0.5702	0.00951	0.043635	Itpripl1
ENSMUSG00000074896	110.7912	70.43311	0.65352	0.008869	0.041219	Ifit3
ENSMUSG00000074934	1604.579	746.5693	1.1038	2.78E-10	9.20E-09	Grem1
ENSMUSG00000075033	84.5621	38.36472	1.1402	0.000302	0.002426	Nxpe3
ENSMUSG00000075232	343.917	261.5816	0.3948	0.003752	0.020284	Amd1
ENSMUSG00000075254	687.3215	394.5237	0.80087	2.60E-12	1.18E-10	Heg1
ENSMUSG00000075270	63.68232	30.24898	1.074	0.000317	0.002527	Pde11a
ENSMUSG00000075304	139.5601	20.60546	2.7598	7.26E-07	1.21E-05	Sp5

ENSMUSG00000075602	7604.197	6413.424	0.2457	0.004759	0.024591	Ly6a
ENSMUSG00000076431	2315.932	762.3537	1.6031	0.000789	0.005497	Sox4
ENSMUSG00000076432	2708.797	2122.288	0.35203	0.000109	0.00101	Ywhaq
ENSMUSG00000078161	335.6803	39.9814	3.0697	0.000426	0.00326	Erich3
ENSMUSG00000078453	757.4088	549.1407	0.4639	6.09E-05	0.000606	Abracl
ENSMUSG00000078566	876.2656	302.2681	1.5355	4.92E-39	1.63E-36	Bnip3
ENSMUSG00000078612	79.25771	45.46612	0.80176	0.00383	0.020649	1700024P16Rik
ENSMUSG00000078763	33.72707	13.17465	1.3561	0.000402	0.003107	Slfn1
ENSMUSG00000078945	1048.352	869.8799	0.26923	0.009008	0.041788	Naip2
ENSMUSG00000078949	56.40089	13.07999	2.1084	0.002565	0.014786	R3hdml
ENSMUSG00000078970	190.31	140.8967	0.43371	0.006834	0.033382	Wdr92
ENSMUSG00000078974	1132.609	904.6608	0.3242	0.001464	0.009255	Sec61g
ENSMUSG00000079012	23.11089	1.807031	3.6769	0.000258	0.002116	Serpina3m
ENSMUSG00000079018	672.2924	430.4562	0.64322	5.76E-08	1.23E-06	Ly6c1
ENSMUSG00000079180	107.83	11.07629	3.2832	0.006858	0.033477	Mptx2
ENSMUSG00000079215	1005.439	787.7317	0.35205	0.000416	0.003194	Zfp664
ENSMUSG00000079293	125.0424	58.05047	1.107	9.77E-08	1.96E-06	Clec7a
ENSMUSG00000079330	22.55649	3.740821	2.5921	0.001901	0.011546	Lemd1
ENSMUSG00000079414	15.05295	2.853203	2.3994	0.000735	0.005191	Gm11110
ENSMUSG00000079435	3847.944	2641.267	0.54286	2.90E-09	8.08E-08	Rpl36a
ENSMUSG00000079597	11.03359	0.304351	5.18	0.003963	0.021202	Gm5483
ENSMUSG00000079641	4007.907	2790.292	0.52243	6.14E-09	1.59E-07	Rpl39
ENSMUSG00000079658	603.7764	483.9964	0.31902	0.005577	0.028044	Tceb1
ENSMUSG00000081683	28.91154	5.18631	2.4789	0.000433	0.00331	Fzd10
ENSMUSG00000082361	161.6402	74.90205	1.1097	2.09E-09	5.99E-08	Btc
ENSMUSG00000083012	170.9379	126.4605	0.43479	0.009867	0.044956	Fam220a
ENSMUSG00000085111	34.45117	3.277955	3.3937	3.43E-06	4.82E-05	Ascl4
ENSMUSG00000085795	593.3701	401.8793	0.56217	3.35E-06	4.74E-05	Zfp703
ENSMUSG00000089672	143.8463	50.54369	1.5089	2.01E-12	9.25E-11	Lilr4b
ENSMUSG00000089762	108.7634	47.15843	1.2056	0.008536	0.039952	Ier5l
ENSMUSG00000089789	95.49742	34.11656	1.485	0.001341	0.008579	Rdh1
ENSMUSG00000089901	321.5052	116.0053	1.4707	6.34E-09	1.63E-07	Gm8113
ENSMUSG00000089942	21.80612	8.077288	1.4328	0.004403	0.023072	Pira2
ENSMUSG00000090176	87.9679	47.9761	0.87466	0.000455	0.00345	Cd200r2
ENSMUSG00000090394	183.0744	119.1511	0.61964	0.000235	0.001959	4930523C07Rik
ENSMUSG00000090946	980.2997	781.2505	0.32744	0.003057	0.017082	Ccdc71l
ENSMUSG00000092083	48.51856	14.53785	1.7387	0.000293	0.002363	Kcnb2
ENSMUSG00000092486	57.07282	28.50546	1.0016	0.001082	0.00717	2610524H06Rik
ENSMUSG00000093674	7672.121	5730.948	0.42085	0.000295	0.00237	Rpl41
ENSMUSG00000093904	1265.348	846.1969	0.58047	1.33E-08	3.21E-07	Tomm20
ENSMUSG00000093916	78.68948	27.09816	1.538	4.90E-08	1.06E-06	Gm379
ENSMUSG00000094818	41.13941	3.030794	3.7628	9.51E-07	1.53E-05	Gm15308
ENSMUSG00000095115	321.6095	248.9732	0.36932	0.009355	0.043128	Itpripl2
ENSMUSG00000095742	250.0183	171.0259	0.54782	0.000435	0.003319	CAAA01147332.1
ENSMUSG00000096001	3222.184	2189.943	0.55715	3.67E-05	0.000389	2610528A11Rik
ENSMUSG00000096719	6.263855	0	Inf	0.001103	0.007261	Mrgpra2b
ENSMUSG00000098078	156.1726	56.37046	1.4701	5.32E-13	2.61E-11	Gm26992
ENSMUSG00000098132	57.84946	18.31679	1.6591	0.000199	0.001705	Rassf10
ENSMUSG00000098207	180.7703	109.4006	0.72454	8.48E-05	0.000808	Arl14
ENSMUSG00000098274	4146.499	3072.216	0.43261	9.44E-07	1.52E-05	Rpl24
ENSMUSG00000101389	54.71762	22.24092	1.2988	3.56E-05	0.000379	Ms4a4a
ENSMUSG00000102037	10.4006	1.509038	2.785	0.003265	0.018018	Bcl2a1a

Supplementary Table S3: List of downregulated genes in tumors (GR_T) of *Ifngr1*$^{-/-}$ mice

Gene_id	read count_ GR_T	read count_ WT_T	log2 FoldChange	pval	padj	Gene_Name
ENSMUSG00000000088	2258.558	2949.162	−0.3849	1.33E-05	0.000159	Cox5a
ENSMUSG00000000154	433.8053	862.6006	−0.99165	7.55E-07	1.25E-05	Slc22a18
ENSMUSG00000000157	23.5351	52.61734	−1.1607	0.000254	0.002089	Itgb2l
ENSMUSG00000000266	439.8973	559.8028	−0.34775	0.002705	0.015436	Mid2
ENSMUSG00000000278	362.5457	478.96	−0.40174	0.001099	0.007246	Scpep1
ENSMUSG00000000308	11805.8	15986.87	−0.43739	1.71E-07	3.26E-06	Ckmt1
ENSMUSG00000000359	15.6597	34.3694	−1.1341	0.010326	0.046679	Rem1
ENSMUSG00000000384	606.2945	819.6762	−0.43504	5.61E-05	0.000565	Tbrg4
ENSMUSG00000000394	118.0932	354.159	−1.5845	4.58E-14	2.57E-12	Gcg
ENSMUSG00000000416	120.585	169.3136	−0.48965	0.003356	0.01844	Cttnbp2
ENSMUSG00000000532	758.7332	998.5386	−0.39623	5.94E-05	0.000593	Acvr1b
ENSMUSG00000000538	581.4099	1027.802	−0.82193	8.42E-15	5.18E-13	Tom1l2
ENSMUSG00000000579	83.73301	130.1695	−0.63652	0.000881	0.006028	Dynlt1c
ENSMUSG00000000631	1835.412	2376.487	−0.37273	3.46E-05	0.000369	Myo18a
ENSMUSG00000000738	616.3958	784.2475	−0.34745	0.001652	0.01027	Spg7
ENSMUSG00000000794	32.46796	67.22536	−1.05	0.002431	0.01413	Kcnn3
ENSMUSG00000000805	3893.824	10817.41	−1.4741	5.62E-48	3.04E-45	Car4
ENSMUSG00000000876	187.9551	247.3821	−0.39635	0.007222	0.034861	Pxmp4
ENSMUSG00000000976	178.8064	239.1066	−0.41926	0.00502	0.025721	Heatr6
ENSMUSG00000001062	115.5747	185.6223	−0.68354	6.15E-05	0.00061	Vps9d1
ENSMUSG00000001089	1074.335	1357.486	−0.33749	0.000307	0.002454	Luzp1
ENSMUSG00000001095	853.5486	2668.24	−1.6443	1.27E-26	2.12E-24	Slc13a2
ENSMUSG00000001143	114.3543	184.0624	−0.68669	4.36E-05	0.000452	Lman2l
ENSMUSG00000001151	349.6144	493.8793	−0.49839	6.66E-05	0.000653	Pcnt
ENSMUSG00000001157	276.0665	383.8081	−0.47537	0.000371	0.0029	Gmcl1
ENSMUSG00000001225	2186.563	8945.063	−2.0324	2.51E-23	3.48E-21	Slc26a3
ENSMUSG00000001229	414.81	560.0089	−0.433	0.000196	0.001682	Dpp9
ENSMUSG00000001270	621.5897	1167.018	−0.90879	0.000425	0.003257	Ckb
ENSMUSG00000001300	646.6578	903.5339	−0.48258	4.15E-06	5.69E-05	Efnb2
ENSMUSG00000001323	91.77841	165.1169	−0.84726	4.40E-06	5.99E-05	Srr
ENSMUSG00000001445	432.767	540.797	−0.3215	0.007362	0.035387	Mrpl10
ENSMUSG00000001517	237.6994	307.0152	−0.36917	0.007393	0.035507	Foxm1
ENSMUSG00000001604	157.1444	267.9545	−0.7699	3.32E-07	5.95E-06	Tcea3
ENSMUSG00000001663	103.0664	144.9753	−0.49223	0.005284	0.026838	Gstt1
ENSMUSG00000001665	204.6318	391.9762	−0.93774	1.87E-12	8.69E-11	Gstt3
ENSMUSG00000001739	502.5195	1339.204	−1.4141	3.58E-07	6.36E-06	Cldn15
ENSMUSG00000001750	466.0444	592.136	−0.34546	0.007248	0.03495	Tcirg1
ENSMUSG00000001774	364.7183	506.7991	−0.47463	9.00E-05	0.00085	Chordc1
ENSMUSG00000001855	275.5699	354.3579	−0.36279	0.00721	0.034816	Nup214
ENSMUSG00000001924	2799.403	3507.165	−0.32519	0.000268	0.002183	Uba1
ENSMUSG00000001943	37.17827	76.57724	−1.0425	0.000413	0.00317	Vsig2
ENSMUSG00000001983	129.544	183.034	−0.49867	0.003036	0.016996	Taco1
ENSMUSG00000002028	642.9713	842.1908	−0.38939	0.000325	0.002582	Kmt2a
ENSMUSG00000002032	12.24715	34.36402	−1.4885	0.00023	0.001924	Tmem25
ENSMUSG00000002108	110.472	170.7537	−0.62824	0.000273	0.002219	Nr1h3

ENSMUSG00000002222	1073.538	1479.727	−0.46296	2.66E-06	3.85E-05	Rmnd5a
ENSMUSG00000002227	387.7132	726.2972	−0.90557	2.75E-08	6.21E-07	Mov10
ENSMUSG00000002250	1245.584	2064.423	−0.72892	1.30E-14	7.83E-13	Ppard
ENSMUSG00000002280	177.2211	239.9049	−0.43691	0.004775	0.024649	Narfl
ENSMUSG00000002320	783.9338	972.8096	−0.31143	0.002783	0.015807	Tm9sf1
ENSMUSG00000002329	497.9308	750.3465	−0.59161	1.64E-07	3.15E-06	Mdp1
ENSMUSG00000002346	68.45195	120.2598	−0.81299	9.86E-05	0.000922	Slc25a42
ENSMUSG00000002384	56.90278	90.02381	−0.66181	0.002328	0.013675	Bmp8b
ENSMUSG00000002496	421.6348	551.5053	−0.38738	0.000921	0.006266	Tsc2
ENSMUSG00000002565	507.1503	923.3833	−0.86452	1.74E-15	1.15E-13	Scin
ENSMUSG00000002625	144.1077	210.196	−0.54459	0.000703	0.004993	Akap8l
ENSMUSG00000002733	337.5569	443.8349	−0.39489	0.002858	0.016145	Plekha3
ENSMUSG00000002748	1030.807	1244.203	−0.27145	0.008693	0.04054	Baz1b
ENSMUSG00000002763	318.849	546.7471	−0.778	1.64E-10	5.64E-09	Pex6
ENSMUSG00000002781	82.57203	134.7345	−0.70639	0.000532	0.003952	Tmem143
ENSMUSG00000002812	1533.022	1838.957	−0.26251	0.004614	0.024003	Flii
ENSMUSG00000002820	437.8367	616.4325	−0.49355	1.14E-05	0.000139	Atg4d
ENSMUSG00000002833	217.9623	291.6406	−0.42011	0.004853	0.024996	Hdgfrp2
ENSMUSG00000002846	181.8016	244.4391	−0.42711	0.00426	0.022448	Timmdc1
ENSMUSG00000002957	620.8919	925.9356	−0.57657	5.01E-08	1.08E-06	Ap2a2
ENSMUSG00000002981	1534.743	1897.688	−0.30625	0.001695	0.010503	Clptm1
ENSMUSG00000002984	608.2851	813.1227	−0.41873	8.55E-05	0.000814	Tomm40
ENSMUSG00000002992	56.73433	427.0275	−2.912	0.000168	0.001472	Apoc2
ENSMUSG00000003031	910.0848	1101.961	−0.276	0.008749	0.040777	Cdkn1b
ENSMUSG00000003032	4923.912	6058.845	−0.29924	0.000385	0.002991	Klf4
ENSMUSG00000003037	1027.376	1273.443	−0.30977	0.001791	0.010985	Rab8a
ENSMUSG00000003068	660.7578	836.4096	−0.34009	0.001304	0.008368	Stk11
ENSMUSG00000003099	789.468	1002.446	−0.34457	0.000905	0.006166	Ppp5c
ENSMUSG00000003299	302.0284	414.5629	−0.45691	0.000628	0.004552	Mrpl4
ENSMUSG00000003344	252.3227	365.4179	−0.53428	5.63E-05	0.000566	Btbd2
ENSMUSG00000003402	917.7318	1125.683	−0.29466	0.003658	0.019851	Prkcsh
ENSMUSG00000003546	1159.422	1509.438	−0.38061	0.0065	0.031931	Klc4
ENSMUSG00000003559	298.1781	438.3022	−0.55575	5.96E-06	7.86E-05	As3mt
ENSMUSG00000003644	1858.204	2440.521	−0.39328	1.72E-05	0.000199	Rps6ka1
ENSMUSG00000003657	43.91208	73.18499	−0.73693	0.002559	0.014759	Calb2
ENSMUSG00000003810	778.0603	1083	−0.47708	1.75E-06	2.66E-05	Mast2
ENSMUSG00000003813	262.5917	366.3282	−0.48032	0.000234	0.001958	Rad23a
ENSMUSG00000003814	7140.288	8936.446	−0.32372	0.000419	0.003218	Calr
ENSMUSG00000003847	684.1108	850.5184	−0.31411	0.006084	0.030168	Nfat5
ENSMUSG00000003849	246.2537	363.3703	−0.5613	8.68E-05	0.000823	Nqo1
ENSMUSG00000003863	59.01781	320.1833	−2.4397	2.43E-08	5.56E-07	Ppfia3
ENSMUSG00000004038	70.69675	237.6186	−1.7489	2.66E-18	2.45E-16	Gstm3
ENSMUSG00000004069	497.7469	712.4954	−0.51747	2.51E-06	3.65E-05	Dnaja3
ENSMUSG00000004110	62.44935	154.6686	−1.3084	8.03E-11	2.87E-09	Cacna1e
ENSMUSG00000004187	44.48019	92.75	−1.0602	3.79E-05	0.0004	Kifc2
ENSMUSG00000004207	5956.693	7205.961	−0.27468	0.001128	0.007409	Psap
ENSMUSG00000004264	1508.503	1784.561	−0.24245	0.010697	0.047998	Phb2

ENSMUSG00000004356	588.2855	928.4778	−0.65835	7.39E-10	2.25E-08	Utp20
ENSMUSG00000004366	59.45423	100.7975	−0.76161	0.00519	0.026429	Sst
ENSMUSG00000004460	1129.308	1556.825	−0.46317	1.80E-06	2.72E-05	Dnajb11
ENSMUSG00000004561	134.9923	182.5973	−0.43579	0.009631	0.044085	Mettl17
ENSMUSG00000004565	359.3363	516.726	−0.52407	1.31E-05	0.000156	Pnpla6
ENSMUSG00000004633	130.2297	226.7008	−0.79973	1.27E-06	1.99E-05	Chn2
ENSMUSG00000004667	423.9318	524.2823	−0.30651	0.010019	0.045603	Polr2e
ENSMUSG00000004668	3.738688	25.75366	−2.7842	6.90E-07	1.16E-05	Abca13
ENSMUSG00000004748	60.01545	136.7074	−1.1877	5.28E-09	1.40E-07	Mtfp1
ENSMUSG00000004789	1548.05	2345.84	−0.59965	9.48E-11	3.35E-09	Dlst
ENSMUSG00000004815	186.5118	347.0218	−0.89576	9.97E-11	3.51E-09	Dgkq
ENSMUSG00000004931	193.5014	292.1058	−0.59415	5.96E-05	0.000594	Apba3
ENSMUSG00000005102	138.3206	193.7113	−0.48589	0.006045	0.03	Eif2ak4
ENSMUSG00000005225	552.8683	836.8519	−0.59804	1.47E-08	3.51E-07	Plekha8
ENSMUSG00000005268	43.43009	154.0694	−1.8268	0.004687	0.024302	Prlr
ENSMUSG00000005299	978.7065	1707.814	−0.8032	7.12E-17	5.47E-15	Letm1
ENSMUSG00000005354	1162.962	1440.561	−0.30883	0.001401	0.008894	Txn2
ENSMUSG00000005360	7.431794	22.65999	−1.6084	0.001385	0.008807	Slc1a3
ENSMUSG00000005373	76.40635	208.3406	−1.4472	2.37E-06	3.47E-05	Mlxipl
ENSMUSG00000005469	456.693	596.8507	−0.38615	0.000779	0.005445	Prkaca
ENSMUSG00000005534	615.2996	799.9695	−0.37866	0.000675	0.004821	Insr
ENSMUSG00000005611	209.891	376.8389	−0.84431	7.38E-10	2.25E-08	Mrvi1
ENSMUSG00000005615	680.2542	880.2212	−0.37179	0.000675	0.004821	Pcyt1a
ENSMUSG00000005672	281.8994	550.5847	−0.96578	1.28E-14	7.73E-13	Kit
ENSMUSG00000005674	84.69213	124.6795	−0.55793	0.00321	0.017775	Tomm40l
ENSMUSG00000005683	3958.297	6637.253	−0.74571	2.66E-17	2.17E-15	Cs
ENSMUSG00000005686	407.196	512.6005	−0.33211	0.007757	0.036995	Ampd3
ENSMUSG00000005823	789.6363	973.2854	−0.30167	0.003493	0.019076	Gpr108
ENSMUSG00000005951	107.5032	150.0529	−0.48109	0.005819	0.029048	Shpk
ENSMUSG00000005973	115.2224	170.3343	−0.56395	0.002054	0.012353	Rcn1
ENSMUSG00000006050	747.627	919.3608	−0.29831	0.002685	0.015356	Sra1
ENSMUSG00000006057	1957.554	2559.84	−0.387	1.77E-05	0.000203	Atp5g1
ENSMUSG00000006276	312.2783	402.8605	−0.36745	0.003373	0.018509	Eps15l1
ENSMUSG00000006281	870.9508	1654.363	−0.92561	2.81E-20	2.96E-18	Tep1
ENSMUSG00000006310	20.37693	39.72122	−0.96297	0.003259	0.018001	Zbtb32
ENSMUSG00000006313	24.95735	53.50492	−1.1002	0.000175	0.001527	Upk1a
ENSMUSG00000006362	131.2083	211.2672	−0.68721	3.20E-05	0.000344	Cbfa2t3
ENSMUSG00000006369	523.7611	762.9725	−0.54272	4.86E-07	8.37E-06	Fbln1
ENSMUSG00000006378	181.5929	238.4106	−0.39274	0.007161	0.034644	Gcat
ENSMUSG00000006392	278.0693	354.3335	−0.34966	0.007174	0.03468	Med8
ENSMUSG00000006398	404.4161	527.8879	−0.38439	0.001577	0.009856	Cdc20
ENSMUSG00000006456	256.5574	345.9462	−0.43127	0.001753	0.010784	Rbm14
ENSMUSG00000006476	357.6789	495.3771	−0.46986	0.000146	0.001305	Nsmf
ENSMUSG00000006517	549.2535	667.5759	−0.28146	0.008118	0.038407	Mvd
ENSMUSG00000006676	821.0424	1075.768	−0.38984	0.000328	0.002602	Usp19
ENSMUSG00000006711	8.110431	21.53153	−1.4086	0.005153	0.026283	D130043K22Rik
ENSMUSG00000006731	426.4121	1030.869	−1.2735	3.14E-11	1.20E-09	B4galnt1

ENSMUSG00000006818	781.0433	958.7778	−0.29579	0.005148	0.026263	Sod2
ENSMUSG00000006850	87.87663	128.3067	−0.54604	0.004667	0.024218	Tmco6
ENSMUSG00000006920	350.5335	541.1034	−0.62635	9.39E-08	1.89E-06	Ezh1
ENSMUSG00000007029	969.7038	1168.957	−0.26961	0.01092	0.048754	Vars
ENSMUSG00000007038	777.8542	1785.216	−1.1985	1.25E-21	1.49E-19	Neu1
ENSMUSG00000007097	105.0953	170.8356	−0.70091	6.11E-05	0.000607	Atp1a2
ENSMUSG00000007817	916.2801	1376.436	−0.58708	7.04E-08	1.47E-06	Zmiz1
ENSMUSG00000007833	471.7076	723.0116	−0.61613	1.00E-07	2.00E-06	Aldh16a1
ENSMUSG00000007837	245.1786	349.3455	−0.51082	0.001561	0.009767	Prrg2
ENSMUSG00000008206	526.4178	798.8347	−0.60169	8.41E-08	1.73E-06	Cers4
ENSMUSG00000008348	751.2262	1295.756	−0.78647	2.08E-06	3.09E-05	Ubc
ENSMUSG00000008730	1565.121	1926.267	−0.29953	0.001842	0.011267	Hipk1
ENSMUSG00000008892	668.248	948.3441	−0.50503	1.45E-06	2.24E-05	Vdac3
ENSMUSG00000009073	935.3347	1185.642	−0.34211	0.000453	0.003439	Nf2
ENSMUSG00000009090	794.3409	1112.846	−0.48642	2.11E-06	3.13E-05	Ap1b1
ENSMUSG00000009092	63.04919	142.2733	−1.1741	2.90E-09	8.08E-08	Derl3
ENSMUSG00000009145	189.854	357.8472	−0.91445	9.32E-11	3.31E-09	Dqx1
ENSMUSG00000009185	66.72303	197.6732	−1.5669	7.05E-17	5.45E-15	Ccl8
ENSMUSG00000009246	21.00999	70.49068	−1.7464	2.89E-09	8.08E-08	Trpm5
ENSMUSG00000009281	182.2261	245.0276	−0.42721	0.005702	0.028607	Rarres2
ENSMUSG00000009291	1198.019	1742.877	−0.54082	8.26E-09	2.07E-07	Pttg1ip
ENSMUSG00000009356	209.8189	472.674	−1.1717	1.73E-14	1.02E-12	Lpo
ENSMUSG00000009647	983.7714	1286.119	−0.38663	8.95E-05	0.000846	Mcu
ENSMUSG00000009681	369.013	464.6983	−0.33262	0.005791	0.028943	Bcr
ENSMUSG00000009772	273.9466	468.6112	−0.7745	4.70E-06	6.35E-05	Nuak2
ENSMUSG00000009863	1655.079	2272.617	−0.45745	8.82E-07	1.43E-05	Sdhb
ENSMUSG00000009995	466.4605	588.0507	−0.33419	0.003395	0.018612	Taz
ENSMUSG00000010057	96.45699	137.195	−0.50827	0.009498	0.043606	Nprl2
ENSMUSG00000010097	1011.939	1230.751	−0.28242	0.00697	0.033941	Nxf1
ENSMUSG00000010362	77.54101	111.0544	−0.51824	0.009079	0.042076	Rdm1
ENSMUSG00000010601	98.91071	315.1335	−1.6718	1.19E-11	4.88E-10	Apol7a
ENSMUSG00000010660	182.5137	356.7562	−0.96693	1.07E-11	4.42E-10	Plcd1
ENSMUSG00000011256	238.7111	336.8207	−0.49671	0.000299	0.002408	Adam19
ENSMUSG00000011884	664.0857	875.339	−0.39847	0.000109	0.001009	Gltp
ENSMUSG00000012705	12.08413	37.60988	−1.638	6.33E-05	0.000625	Retn
ENSMUSG00000013160	625.8687	842.2189	−0.42834	8.17E-05	0.000782	Atp6v0d1
ENSMUSG00000013418	1835.053	3275.011	−0.83568	2.06E-20	2.18E-18	B4galnt2
ENSMUSG00000013523	604.9222	812.5603	−0.42573	4.03E-05	0.000422	Bcas1
ENSMUSG00000013584	27.27382	67.57355	−1.3089	4.67E-06	6.31E-05	Aldh1a2
ENSMUSG00000013593	2528.326	2982.115	−0.23815	0.007552	0.036162	Ndufs2
ENSMUSG00000013629	379.0219	522.4999	−0.46315	0.000191	0.001651	Cad
ENSMUSG00000013653	1399.747	6901.83	−2.3018	0.001583	0.009879	1810065E05Rik
ENSMUSG00000013997	249.6081	364.5857	−0.54659	4.91E-05	0.000502	Nit1
ENSMUSG00000014077	2920.909	4213.932	−0.52875	1.96E-09	5.66E-08	Chp1
ENSMUSG00000014355	874.2178	1124.947	−0.36379	0.000413	0.00317	Anapc1
ENSMUSG00000014361	166.4684	386.5704	−1.2155	5.62E-06	7.44E-05	Mertk
ENSMUSG00000014418	480.3653	902.611	−0.90997	1.07E-16	8.08E-15	Hps5

ENSMUSG00000014503	30.42513	54.95011	−0.85286	0.002505	0.014502	Pkd2l2
ENSMUSG00000014606	802.7598	1007.464	−0.32769	0.001876	0.01141	Slc25a11
ENSMUSG00000014778	240.891	321.233	−0.41524	0.001964	0.011877	Fhod1
ENSMUSG00000014786	48.02256	73.60342	−0.61606	0.007862	0.037415	Slc9a5
ENSMUSG00000014846	140.4216	453.2658	−1.6906	2.46E-32	5.55E-30	Tppp3
ENSMUSG00000015092	972.3688	1430.341	−0.55678	0.002285	0.013468	Edf1
ENSMUSG00000015095	352.5638	462.5018	−0.39157	0.002191	0.012996	Fbxw5
ENSMUSG00000015112	286.5707	425.9121	−0.57166	8.90E-06	0.000111	Slc25a13
ENSMUSG00000015202	133.5371	226.3632	−0.7614	1.00E-06	1.60E-05	Cnksr3
ENSMUSG00000015222	33.65525	67.63006	−1.0068	0.000391	0.003026	Map2
ENSMUSG00000015461	469.9849	589.6826	−0.32732	0.00501	0.025678	Atf6b
ENSMUSG00000015478	605.9636	862.5378	−0.50936	3.30E-06	4.67E-05	Rnf5
ENSMUSG00000015568	210.9607	337.3502	−0.67727	0.002539	0.014662	Lpl
ENSMUSG00000015790	396.0252	490.8459	−0.30968	0.006912	0.033711	Surf1
ENSMUSG00000015806	542.0902	690.2829	−0.34865	0.000954	0.006454	Qdpr
ENSMUSG00000015839	1117.298	1441.372	−0.36743	9.59E-05	0.0009	Nfe2l2
ENSMUSG00000015846	750.3962	968.3964	−0.36795	0.0005	0.003732	Rxra
ENSMUSG00000015869	456.1651	1041.45	−1.191	5.52E-12	2.39E-10	Prpsap1
ENSMUSG00000016128	205.663	299.2927	−0.54127	0.00017	0.001486	Stard13
ENSMUSG00000016319	9983.965	12950.85	−0.37536	1.13E-05	0.000137	Slc25a5
ENSMUSG00000016349	33.3221	59.61636	−0.83923	0.00192	0.011642	Eef1a2
ENSMUSG00000016495	504.8984	790.3842	−0.64656	3.61E-09	9.84E-08	Plgrkt
ENSMUSG00000016496	85.52145	152.0114	−0.82982	2.75E-05	0.0003	Cd274
ENSMUSG00000017119	1531.738	1984.748	−0.37379	7.06E-05	0.000685	Nbr1
ENSMUSG00000017167	47.93827	119.9246	−1.3229	5.20E-09	1.38E-07	Cntnap1
ENSMUSG00000017188	472.1055	776.5651	−0.718	1.48E-10	5.09E-09	Coa3
ENSMUSG00000017307	211.0394	312.1842	−0.56488	4.90E-05	0.000502	Acot8
ENSMUSG00000017478	646.5384	811.0205	−0.327	0.001741	0.010738	Zc3h18
ENSMUSG00000017615	1107.945	1314.836	−0.247	0.010456	0.04712	Tnfaip1
ENSMUSG00000017631	1012.441	1792.388	−0.82404	2.42E-17	1.98E-15	Abr
ENSMUSG00000017639	383.5765	502.9421	−0.39088	0.001008	0.006758	Rab11fip4
ENSMUSG00000017677	891.0579	1186.898	−0.4136	4.18E-05	0.000436	Wsb1
ENSMUSG00000017688	197.7214	312.4164	−0.66	0.00431	0.022679	Hnf4g
ENSMUSG00000017692	26.40582	50.63272	−0.93921	0.001505	0.009464	Rhbdl3
ENSMUSG00000017756	678.3029	1133.076	−0.74024	5.71E-13	2.79E-11	Slc12a7
ENSMUSG00000017817	268.9348	385.9848	−0.52129	5.25E-05	0.000532	Jph2
ENSMUSG00000017837	578.3492	724.8668	−0.32578	0.003048	0.017044	Nkiras2
ENSMUSG00000017868	464.9531	689.2348	−0.56791	1.95E-07	3.69E-06	Sgk2
ENSMUSG00000017929	455.5602	736.5703	−0.69318	0.001863	0.011367	B4galt5
ENSMUSG00000017950	5269.841	6937.994	−0.39676	4.45E-06	6.04E-05	Hnf4a
ENSMUSG00000017969	49.37976	77.9836	−0.65925	0.005803	0.028983	Ptgis
ENSMUSG00000018042	1105.985	1304.896	−0.2386	0.00943	0.043388	Cyb5r3
ENSMUSG00000018102	396.9986	603.2937	−0.60373	3.65E-07	6.47E-06	Hist1h2bc
ENSMUSG00000018166	2131.509	2721.449	−0.3525	0.000235	0.001963	Erbb3
ENSMUSG00000018372	85.11647	122.6063	−0.52652	0.007159	0.034643	Cep95
ENSMUSG00000018401	420.8201	755.4857	−0.8442	1.24E-11	5.05E-10	Mtmr4
ENSMUSG00000018427	214.8377	328.4783	−0.61255	2.15E-05	0.000241	Ypel2

ENSMUSG00000018428	631.6958	940.8247	−0.5747	5.41E-08	1.16E-06	Akap1
ENSMUSG00000018501	1664.461	2260.521	−0.4416	1.56E-06	2.40E-05	Ncor1
ENSMUSG00000018559	554.5139	702.8144	−0.34192	0.002965	0.016668	Ctdnep1
ENSMUSG00000018574	504.097	649.1714	−0.3649	0.001098	0.007245	Acadvl
ENSMUSG00000018659	326.7842	505.0596	−0.62812	3.41E-07	6.09E-06	Pnpo
ENSMUSG00000018661	408.481	529.1816	−0.37349	0.001998	0.012064	Cog1
ENSMUSG00000018677	982.2231	1331.797	−0.43925	1.35E-05	0.00016	Slc25a39
ENSMUSG00000018707	2840.47	3973.16	−0.48416	4.15E-08	9.09E-07	Dync1h1
ENSMUSG00000018740	232.4406	356.7169	−0.61792	3.63E-06	5.06E-05	Slc25a35
ENSMUSG00000018830	6806.12	10689.68	−0.65131	1.37E-14	8.25E-13	Myh11
ENSMUSG00000018846	1528.726	2305.722	−0.59289	1.04E-05	0.000128	Pank3
ENSMUSG00000018861	72.42566	104.9176	−0.53468	0.008867	0.041219	Fdxr
ENSMUSG00000018899	1060.549	2338.24	−1.1406	2.92E-24	4.27E-22	Irf1
ENSMUSG00000018900	196.3199	276.6098	−0.49465	0.000975	0.006569	Slc22a5
ENSMUSG00000018909	568.51	805.8795	−0.50338	5.10E-06	6.82E-05	Arrb1
ENSMUSG00000018999	231.404	303.3707	−0.39067	0.006729	0.032939	Slc35b4
ENSMUSG00000019039	217.5574	299.9233	−0.4632	0.000587	0.004312	Dalrd3
ENSMUSG00000019179	2574.157	3656.156	−0.50623	4.17E-06	5.71E-05	Mdh2
ENSMUSG00000019278	481.9768	2457.786	−2.3503	1.07E-16	8.06E-15	Dpep1
ENSMUSG00000019295	186.3102	245.3641	−0.39722	0.007333	0.035275	Tmem129
ENSMUSG00000019326	140.8059	186.5537	−0.40588	0.008295	0.039049	Aoc3
ENSMUSG00000019359	34.68645	116.4591	−1.7474	0.006009	0.029855	Gdpd2
ENSMUSG00000019370	4623.866	5566.764	−0.26774	0.002205	0.013061	Calm3
ENSMUSG00000019429	2.127129	12.29369	−2.5309	0.001717	0.010623	Ffar3
ENSMUSG00000019467	102.7595	148.2265	−0.52853	0.003899	0.02093	Arhgef25
ENSMUSG00000019558	4345.7	5800.637	−0.41662	0.000122	0.001109	Slc6a8
ENSMUSG00000019731	838.3322	1085.685	−0.37301	0.000253	0.002085	Slc35e1
ENSMUSG00000019734	2079.616	2539.132	−0.28802	0.001398	0.008879	Tmc4
ENSMUSG00000019763	129.5246	192.0304	−0.56811	0.001043	0.006959	Rmnd1
ENSMUSG00000019775	46.17599	98.18971	−1.0884	1.63E-06	2.50E-05	Rgs17
ENSMUSG00000019804	686.875	922.8159	−0.426	3.81E-05	0.000402	Snx3
ENSMUSG00000019864	124.6373	264.1431	−1.0836	1.34E-11	5.42E-10	Rtn4ip1
ENSMUSG00000019866	912.3167	1173.755	−0.36353	0.000307	0.002454	Aim1
ENSMUSG00000019907	776.8879	956.3012	−0.29976	0.004604	0.023968	Ppp1r12a
ENSMUSG00000019952	260.119	343.3702	−0.40059	0.003044	0.017029	Poc1b
ENSMUSG00000019969	1178.697	1689.142	−0.5191	3.15E-08	7.02E-07	Psen1
ENSMUSG00000019970	1048.279	2306.936	−1.138	3.50E-09	9.57E-08	Sgk1
ENSMUSG00000019984	289.122	366.974	−0.344	0.010221	0.046296	Med23
ENSMUSG00000019996	881.9234	1162.247	−0.39819	8.92E-05	0.000844	Map7
ENSMUSG00000020009	343.6577	2315.915	−2.7525	########	########	Ifngr1
ENSMUSG00000020038	137.4194	223.0014	−0.69847	1.73E-05	0.000199	Cry1
ENSMUSG00000020069	177.9784	275.6642	−0.63121	3.30E-05	0.000354	Hnrnph3
ENSMUSG00000020072	51.67707	121.3595	−1.2317	0.005748	0.028757	Pbld2
ENSMUSG00000020087	202.3029	274.3776	−0.43965	0.002439	0.014167	Tysnd1
ENSMUSG00000020089	1493.143	2305.06	−0.62645	3.66E-11	1.37E-09	Ppa1
ENSMUSG00000020097	828.5982	1333.788	−0.68678	0.000867	0.005941	Sgpl1
ENSMUSG00000020105	322.1057	555.4429	−0.7861	1.94E-10	6.54E-09	Lrig3

ENSMUSG00000020115	442.9523	567.3118	−0.35699	0.002208	0.013071	Tbk1
ENSMUSG00000020134	558.3096	733.4887	−0.39371	0.000129	0.001173	Peli1
ENSMUSG00000020137	75.55756	135.4128	−0.84172	3.29E-05	0.000354	Thap2
ENSMUSG00000020153	717.5243	1013.662	−0.49848	0.001213	0.007867	Ndufs7
ENSMUSG00000020173	519.9268	656.609	−0.33673	0.003027	0.016953	Cobl
ENSMUSG00000020182	309.2904	426.1424	−0.46237	0.00026	0.002127	Ddc
ENSMUSG00000020196	423.5659	583.4426	−0.462	3.63E-05	0.000385	Cabin1
ENSMUSG00000020198	960.267	1288.147	−0.42379	1.43E-05	0.000168	Ap3d1
ENSMUSG00000020220	1204.666	1608.095	−0.41672	6.65E-06	8.62E-05	Vps13d
ENSMUSG00000020229	1.683472	61.88965	−5.2002	0.010955	0.048877	Slc5a4a
ENSMUSG00000020230	105.3109	151.7643	−0.52718	0.004787	0.024706	Prmt2
ENSMUSG00000020231	495.1516	656.335	−0.40656	0.000405	0.003122	Dip2a
ENSMUSG00000020238	581.4939	793.2143	−0.44795	2.72E-05	0.000297	Ncln
ENSMUSG00000020241	952.9395	1191.79	−0.32267	0.001484	0.009362	Col6a2
ENSMUSG00000020263	1255.013	1681.101	−0.4217	1.91E-05	0.000218	Appl2
ENSMUSG00000020268	17.31687	40.04435	−1.2094	0.00084	0.005794	Lyrm7
ENSMUSG00000020272	181.9454	485.7943	−1.4168	1.68E-11	6.72E-10	Stk10
ENSMUSG00000020300	701.9358	942.0451	−0.42446	5.11E-05	0.000521	Cpeb4
ENSMUSG00000020315	5048.68	6337.551	−0.32802	0.000224	0.001883	Sptbn1
ENSMUSG00000020331	36.43467	79.84222	−1.1318	9.19E-06	0.000115	Hcn2
ENSMUSG00000020400	552.9488	813.6431	−0.55725	3.07E-07	5.56E-06	Tnip1
ENSMUSG00000020407	489.9459	2016.866	−2.0414	1.96E-12	9.07E-11	Upp1
ENSMUSG00000020412	555.526	728.8095	−0.39169	0.000682	0.004865	Ascc2
ENSMUSG00000020415	203.6006	263.6501	−0.37288	0.006408	0.031565	Pttg1
ENSMUSG00000020439	677.104	1239.695	−0.87254	5.49E-17	4.30E-15	Smtn
ENSMUSG00000020455	191.1849	265.2524	−0.4724	0.002898	0.016337	Trim11
ENSMUSG00000020456	1892.884	3798.404	−1.0048	2.58E-25	3.96E-23	Ogdh
ENSMUSG00000020467	114.1844	196.0428	−0.7798	1.60E-05	0.000186	Efemp1
ENSMUSG00000020482	241.9731	323.2722	−0.4179	0.001651	0.01027	Ccdc117
ENSMUSG00000020483	1332.327	2093.241	−0.65179	3.30E-10	1.07E-08	Dynll2
ENSMUSG00000020520	1013.744	1396.287	−0.4619	2.13E-06	3.16E-05	Galnt10
ENSMUSG00000020527	301.8657	579.5679	−0.94107	1.44E-14	8.60E-13	Myo19
ENSMUSG00000020532	744.6473	976.7219	−0.39139	0.000171	0.001498	Acaca
ENSMUSG00000020564	177.6067	241.2248	−0.44169	0.004649	0.024142	Atxn7l1
ENSMUSG00000020576	297.8393	461.1319	−0.63065	6.37E-07	1.07E-05	Nbas
ENSMUSG00000020577	1134.068	1340.288	−0.24104	0.007791	0.037146	Tspan13
ENSMUSG00000020589	93.2393	133.8856	−0.52199	0.002327	0.013672	Fam49a
ENSMUSG00000020611	1246.415	1517.126	−0.28356	0.004037	0.021479	Gna13
ENSMUSG00000020623	90.64924	125.8143	−0.47293	0.009134	0.042271	Map2k6
ENSMUSG00000020715	414.7118	511.7075	−0.30321	0.010269	0.046484	Ern1
ENSMUSG00000020718	61.09216	102.8249	−0.75113	0.000673	0.004812	Polg2
ENSMUSG00000020721	386.91	525.7126	−0.44228	0.000311	0.002481	Helz
ENSMUSG00000020733	1710.014	3735.882	−1.1274	0.002007	0.012111	Slc9a3r1
ENSMUSG00000020740	186.5062	262.9711	−0.49568	0.001021	0.00683	Gga3
ENSMUSG00000020741	905.2303	1555.412	−0.78094	2.18E-15	1.42E-13	Cluh
ENSMUSG00000020747	555.0091	1002.525	−0.85306	6.16E-15	3.84E-13	2310067B10Rik
ENSMUSG00000020752	266.9901	372.9898	−0.48235	0.011114	0.049427	Recql5

ENSMUSG00000020775	268.8502	384.213	−0.5151	0.000256	0.002102	Mrpl38
ENSMUSG00000020777	2045.938	3157.177	−0.62587	9.31E-10	2.80E-08	Acox1
ENSMUSG00000020782	1069.182	1510.075	−0.49811	3.86E-07	6.80E-06	Llgl2
ENSMUSG00000020788	7022.132	9114.755	−0.37629	1.07E-05	0.000131	Atp2a3
ENSMUSG00000020790	1096.589	1355.093	−0.30537	0.001747	0.010758	Ankfy1
ENSMUSG00000020818	185.2017	246.5314	−0.41267	0.009567	0.043846	Mfsd11
ENSMUSG00000020838	79.70154	174.4591	−1.1302	0.004671	0.024227	Slc6a4
ENSMUSG00000020850	1854.005	2334.053	−0.33219	0.000305	0.002442	Prpf8
ENSMUSG00000020864	510.8357	641.5855	−0.32878	0.003753	0.020284	Ankrd40
ENSMUSG00000020865	1381.172	2737.212	−0.98681	2.03E-13	1.04E-11	Abcc3
ENSMUSG00000020875	376.3988	470.993	−0.32344	0.008583	0.040097	Hoxb9
ENSMUSG00000020889	436.9689	564.3554	−0.36907	0.006379	0.031451	Nr1d1
ENSMUSG00000020895	37.48499	65.81701	−0.81215	0.00227	0.013386	Tmem107
ENSMUSG00000020900	126.4966	186.4895	−0.56	0.000411	0.003163	Myh10
ENSMUSG00000020902	127.3259	173.4046	−0.44562	0.007897	0.037531	Ntn1
ENSMUSG00000020917	2488.613	3037.399	−0.28749	0.001666	0.010347	Acly
ENSMUSG00000020926	28.65038	54.81737	−0.93608	0.001211	0.00786	Adam11
ENSMUSG00000020964	1094.671	1401.51	−0.35649	0.000238	0.001985	Sel1l
ENSMUSG00000020986	925.6537	1108.396	−0.25993	0.00826	0.038926	Sec23a
ENSMUSG00000020994	911.8203	1102.307	−0.2737	0.007389	0.035496	Pnn
ENSMUSG00000021037	767.2948	1026.625	−0.42006	0.000242	0.002014	Ahsa1
ENSMUSG00000021038	338.3706	462.9763	−0.45233	0.000192	0.001657	Vipas39
ENSMUSG00000021048	461.312	643.6821	−0.48061	3.67E-05	0.000389	Mthfd1
ENSMUSG00000021087	45.52357	70.88326	−0.63883	0.007641	0.036532	Rtn1
ENSMUSG00000021097	1889.474	2560.059	−0.43819	9.54E-07	1.53E-05	Clmn
ENSMUSG00000021120	64.06104	101.6698	−0.66637	0.009138	0.042272	Pigh
ENSMUSG00000021182	409.0617	534.8008	−0.38668	0.000717	0.005077	Ccdc88c
ENSMUSG00000021194	219.6725	321.4999	−0.54946	0.001238	0.008013	Chga
ENSMUSG00000021196	1816.486	2580.591	−0.50655	4.64E-08	1.01E-06	Pfkp
ENSMUSG00000021200	93.74241	160.5463	−0.77622	2.42E-05	0.000268	Asb2
ENSMUSG00000021213	779.9973	1028.971	−0.39966	5.38E-05	0.000544	Akr1c13
ENSMUSG00000021216	51.53345	170.9009	−1.7296	7.04E-08	1.47E-06	Tubal3
ENSMUSG00000021221	5.09598	30.55299	−2.5839	8.98E-07	1.45E-05	Dpf3
ENSMUSG00000021224	435.1606	580.1391	−0.41485	0.000253	0.002085	Numb
ENSMUSG00000021234	13.69566	31.79579	−1.2151	0.003366	0.018478	Fam161b
ENSMUSG00000021236	860.8539	1811.293	−1.0732	1.90E-19	1.89E-17	Entpd5
ENSMUSG00000021250	773.9195	1010.453	−0.38475	0.000897	0.006119	Fos
ENSMUSG00000021266	1222.095	1736.632	−0.50694	1.37E-07	2.67E-06	Wars
ENSMUSG00000021294	43.93838	108.1186	−1.2991	3.92E-08	8.62E-07	Kif26a
ENSMUSG00000021327	620.9177	918.0515	−0.56417	1.17E-06	1.84E-05	Zkscan3
ENSMUSG00000021339	309.0625	429.7872	−0.47572	0.000242	0.002014	Mrs2
ENSMUSG00000021361	698.9342	1000.352	−0.51728	8.57E-07	1.40E-05	Tmem14c
ENSMUSG00000021366	454.4488	621.9899	−0.45277	6.56E-05	0.000645	Hivep1
ENSMUSG00000021416	23.60707	62.29295	−1.3999	1.79E-05	0.000205	Eci3
ENSMUSG00000021420	246.2868	317.523	−0.36652	0.006993	0.034014	Fars2
ENSMUSG00000021484	2152.927	2636.711	−0.29244	0.002126	0.012692	Lman2
ENSMUSG00000021495	286.421	450.7434	−0.65417	3.42E-06	4.80E-05	Fam193b

ENSMUSG00000021496	90.47377	137.9625	−0.6087	0.001676	0.010402	Pcbd2
ENSMUSG00000021518	837.7644	1019.818	−0.2837	0.00516	0.026313	Ptdss1
ENSMUSG00000021520	1462.935	2100.636	−0.52196	2.79E-08	6.27E-07	Uqcrb
ENSMUSG00000021550	549.2035	869.4945	−0.66284	9.77E-07	1.56E-05	2210016F16Rik
ENSMUSG00000021573	184.5024	715.1307	−1.9546	5.12E-54	3.05E-51	Tppp
ENSMUSG00000021577	3450.175	4862.287	−0.49497	9.94E-09	2.44E-07	Sdha
ENSMUSG00000021591	885.2717	1431.896	−0.69373	0.000171	0.0015	Glrx
ENSMUSG00000021608	145.6212	198.3518	−0.44584	0.005509	0.027764	Lpcat1
ENSMUSG00000021622	7.549271	22.34258	−1.5654	0.003183	0.017648	Ckmt2
ENSMUSG00000021650	334.4828	490.3405	−0.55185	9.19E-06	0.000115	Ptcd2
ENSMUSG00000021666	343.8592	501.988	−0.54584	9.98E-06	0.000123	Gfm2
ENSMUSG00000021676	3879.707	6269.615	−0.69243	3.17E-15	2.02E-13	Iqgap2
ENSMUSG00000021684	113.467	159.5559	−0.49179	0.007321	0.035245	Pde8b
ENSMUSG00000021686	928.119	1116.352	−0.26641	0.005963	0.02966	Ap3b1
ENSMUSG00000021702	1733.921	2314.65	−0.41676	0.000551	0.00408	Thbs4
ENSMUSG00000021704	187.1325	270.5913	−0.53206	0.000398	0.003077	Mtx3
ENSMUSG00000021708	28.67672	76.13353	−1.4087	3.02E-07	5.47E-06	Rasgrf2
ENSMUSG00000021773	142.1699	197.6056	−0.47501	0.002883	0.016267	Comtd1
ENSMUSG00000021786	249.0787	349.2782	−0.48777	0.000241	0.002003	Oxsm
ENSMUSG00000021794	1519.732	1802.906	−0.24651	0.005127	0.026179	Glud1
ENSMUSG00000021798	165.8034	245.1187	−0.56401	0.000371	0.0029	Ldb3
ENSMUSG00000021835	166.4033	263.6227	−0.66379	1.92E-05	0.000218	Bmp4
ENSMUSG00000021866	2226.93	2710.382	−0.28344	0.001768	0.01086	Anxa11
ENSMUSG00000021906	160.6933	250.7453	−0.64191	0.000247	0.002045	Oxnad1
ENSMUSG00000021943	45.1975	79.88635	−0.82171	0.000661	0.004751	Gdf10
ENSMUSG00000021947	579.3305	1056.665	−0.86706	2.72E-07	5.00E-06	Cryl1
ENSMUSG00000021948	1386.897	2235.747	−0.6889	3.66E-13	1.82E-11	Prkcd
ENSMUSG00000021957	3169.218	3718.667	−0.23066	0.009332	0.043034	Tkt
ENSMUSG00000021993	236.3106	320.095	−0.43782	0.002809	0.01593	Mipep
ENSMUSG00000022014	408.6639	538.3361	−0.39759	0.00089	0.006077	Epsti1
ENSMUSG00000022040	122.8964	278.4952	−1.1802	0.000266	0.00217	Ephx2
ENSMUSG00000022057	1750.877	2251.16	−0.36259	0.003992	0.021333	Adamdec1
ENSMUSG00000022095	221.617	312.4818	−0.4957	0.000696	0.004953	Fam160b2
ENSMUSG00000022099	17.52553	34.77575	−0.98862	0.005663	0.028436	Dmtn
ENSMUSG00000022108	5130.046	7385.007	−0.52563	9.02E-10	2.72E-08	Itm2b
ENSMUSG00000022175	2215.106	2850.02	−0.3636	6.02E-05	0.000599	Lrp10
ENSMUSG00000022179	47.00529	80.24688	−0.77162	0.005233	0.026624	4931414P19Rik
ENSMUSG00000022181	7.992936	21.82889	−1.4494	0.006917	0.033721	C6
ENSMUSG00000022216	1046.884	1555.042	−0.57085	5.13E-09	1.37E-07	Psme1
ENSMUSG00000022228	315.4367	451.2835	−0.51668	7.71E-05	0.000742	Zscan26
ENSMUSG00000022229	3218.24	6626.301	−1.0419	1.25E-13	6.52E-12	Atp12a
ENSMUSG00000022231	365.7498	861.2442	−1.2356	3.76E-24	5.46E-22	Sema5a
ENSMUSG00000022235	226.412	327.9411	−0.53449	0.002996	0.016824	Cmbl
ENSMUSG00000022262	11.61413	29.55731	−1.3476	0.003137	0.017423	Dnah5
ENSMUSG00000022265	184.9213	337.5467	−0.86818	1.89E-05	0.000216	Ank
ENSMUSG00000022272	904.9828	1123.536	−0.31208	0.002077	0.012466	Myo10
ENSMUSG00000022332	179.0869	240.4685	−0.42519	0.006248	0.030915	Khdrbs3

ENSMUSG00000022353	366.5067	459.5284	−0.32631	0.00958	0.043896	Mtss1
ENSMUSG00000022354	1736.679	2081.51	−0.2613	0.004451	0.023283	Ndufb9
ENSMUSG00000022383	22.41289	69.90733	−1.6411	2.71E-08	6.14E-07	Ppara
ENSMUSG00000022407	249.3923	360.1536	−0.5302	7.12E-05	0.00069	Adsl
ENSMUSG00000022408	220.6773	289.9372	−0.3938	0.005229	0.026612	Fam83f
ENSMUSG00000022453	1016.499	1321.735	−0.37882	0.000127	0.001152	Naga
ENSMUSG00000022477	4314.666	5987.885	−0.4728	3.55E-08	7.86E-07	Aco2
ENSMUSG00000022479	1101.844	2849.206	−1.3706	9.75E-22	1.18E-19	Vdr
ENSMUSG00000022504	97.17438	271.5645	−1.4826	2.68E-10	8.89E-09	Ciita
ENSMUSG00000022517	623.2608	900.5032	−0.5309	1.11E-06	1.75E-05	Mgrn1
ENSMUSG00000022550	269.9465	482.0589	−0.83654	1.14E-08	2.77E-07	Adck5
ENSMUSG00000022551	2086.745	2999.896	−0.52366	1.83E-08	4.29E-07	Cyc1
ENSMUSG00000022556	171.427	237.0531	−0.46762	0.002352	0.013778	Hsf1
ENSMUSG00000022558	394.6364	495.7316	−0.32904	0.009774	0.0446	Mroh1
ENSMUSG00000022571	312.1223	492.8797	−0.65912	9.01E-08	1.84E-06	Pycrl
ENSMUSG00000022574	319.0198	602.2962	−0.91683	1.05E-05	0.000129	Naprt
ENSMUSG00000022617	288.3013	484.9547	−0.75027	7.40E-09	1.88E-07	Chkb
ENSMUSG00000022620	133.5704	240.306	−0.84727	1.57E-07	3.02E-06	Arsa
ENSMUSG00000022684	461.4822	723.7056	−0.64913	6.57E-09	1.68E-07	Bfar
ENSMUSG00000022687	143.4097	306.1035	−1.0939	7.02E-13	3.40E-11	Boc
ENSMUSG00000022761	442.1887	587.4656	−0.40984	0.000813	0.005637	Lztr1
ENSMUSG00000022769	453.7381	609.9493	−0.42683	0.000379	0.002949	Sdf2l1
ENSMUSG00000022788	864.7034	1273.396	−0.5584	2.01E-08	4.68E-07	Fgd4
ENSMUSG00000022793	292.6125	390.4468	−0.41613	0.002849	0.016119	B4galt4
ENSMUSG00000022803	51.72267	98.65905	−0.93165	0.000104	0.000969	Popdc2
ENSMUSG00000022836	2859.595	4519.202	−0.66026	4.96E-14	2.74E-12	Mylk
ENSMUSG00000022840	167.9693	283.6536	−0.75593	3.33E-07	5.97E-06	Adcy5
ENSMUSG00000022843	1408.794	2548.837	−0.85538	1.04E-19	1.06E-17	Clcn2
ENSMUSG00000022844	408.2855	575.9774	−0.49643	2.42E-05	0.000267	Pdia5
ENSMUSG00000022853	332.3491	442.2651	−0.41221	0.000609	0.004436	Ehhadh
ENSMUSG00000022861	14.53738	31.02256	−1.0936	0.007411	0.035574	Dgkg
ENSMUSG00000022878	20.44884	59.50868	−1.5411	1.05E-06	1.67E-05	Adipoq
ENSMUSG00000022884	2577.654	3550.656	−0.46203	7.07E-05	0.000686	Eif4a2
ENSMUSG00000022885	682.9861	816.8267	−0.25817	0.010577	0.047581	St6gal1
ENSMUSG00000022899	88.33941	124.5528	−0.49563	0.008896	0.041323	Slc15a2
ENSMUSG00000022900	1864.516	2249.319	−0.27069	0.001583	0.009879	Ildr1
ENSMUSG00000022938	1014.32	1259.701	−0.31257	0.001742	0.010738	Fam3b
ENSMUSG00000022946	457.3192	694.3188	−0.6024	7.67E-08	1.59E-06	Dopey2
ENSMUSG00000022956	2638.374	3172	−0.26574	0.003335	0.018346	Atp5o
ENSMUSG00000022957	423.4554	540.39	−0.35179	0.003208	0.017771	Itsn1
ENSMUSG00000022973	307.6141	403.4809	−0.39138	0.00319	0.017676	Synj1
ENSMUSG00000022974	409.2124	543.7505	−0.4101	0.000897	0.006119	Paxbp1
ENSMUSG00000022994	1798.23	2606.915	−0.53577	9.79E-09	2.41E-07	Adcy6
ENSMUSG00000023010	6788.495	9594.628	−0.49914	3.85E-07	6.80E-06	Tmbim6
ENSMUSG00000023015	452.1579	561.9155	−0.31353	0.007905	0.037556	Racgap1
ENSMUSG00000023022	3300.216	3914.14	−0.24614	0.004156	0.022005	Lima1
ENSMUSG00000023046	65.88122	108.8356	−0.72421	0.000511	0.003814	Igfbp6

ENSMUSG00000023072	163.9884	224.8483	−0.45536	0.002076	0.01246	Cep89
ENSMUSG00000023089	678.6749	833.2726	−0.29607	0.003811	0.020582	Ndufa5
ENSMUSG00000023206	53.28164	109.5772	−1.0402	1.68E-06	2.56E-05	Il15ra
ENSMUSG00000023243	404.769	700.9091	−0.79213	3.23E-12	1.45E-10	Kcnk5
ENSMUSG00000023247	1314.643	5901.77	−2.1665	1.49E-51	8.31E-49	Guca2a
ENSMUSG00000023249	325.3938	420.9952	−0.37162	0.001878	0.01142	Parp3
ENSMUSG00000023272	400.7234	712.1221	−0.82952	7.11E-13	3.43E-11	Creld2
ENSMUSG00000023328	79.66814	146.0341	−0.87423	1.98E-06	2.97E-05	Ache
ENSMUSG00000023484	51.93129	90.3784	−0.79937	0.001205	0.007829	Prph
ENSMUSG00000023800	91.77849	148.7221	−0.69639	0.000146	0.001299	Tiam2
ENSMUSG00000023827	384.3326	742.4394	−0.94992	1.22E-16	9.09E-15	Agpat4
ENSMUSG00000023829	141.1845	226.5794	−0.68244	1.48E-05	0.000173	Slc22a1
ENSMUSG00000023830	1027.05	1352.655	−0.39729	6.79E-05	0.000663	Igf2r
ENSMUSG00000023904	271.1334	393.1736	−0.53616	5.49E-05	0.000554	Hcfc1r1
ENSMUSG00000023909	1008.148	1246.19	−0.30582	0.001703	0.010549	Paqr4
ENSMUSG00000023952	1176.646	1749.71	−0.57244	3.16E-09	8.75E-08	Gtpbp2
ENSMUSG00000023959	1365.624	3062.719	−1.1653	5.14E-36	1.35E-33	Clic5
ENSMUSG00000024036	1195.488	1608.675	−0.42827	5.80E-06	7.66E-05	Slc37a1
ENSMUSG00000024044	464.7411	923.6722	−0.99095	0.000644	0.004651	Epb4.1l3
ENSMUSG00000024049	174.2524	239.5377	−0.45907	0.002583	0.014863	Myom1
ENSMUSG00000024052	387.4594	583.3019	−0.5902	0.007425	0.035614	Lpin2
ENSMUSG00000024055	167.5254	280.3262	−0.74273	3.54E-07	6.30E-06	Cyp4f13
ENSMUSG00000024066	1257.592	1688.414	−0.425	0.008381	0.039382	Xdh
ENSMUSG00000024073	1390.196	1722.35	−0.30909	0.001089	0.007199	Birc6
ENSMUSG00000024112	228.5719	351.9175	−0.62259	6.33E-06	8.24E-05	Cacna1h
ENSMUSG00000024120	514.4623	681.9346	−0.40657	0.000162	0.001431	Lrpprc
ENSMUSG00000024131	105.0067	360.9368	−1.7813	0.000433	0.00331	Slc3a1
ENSMUSG00000024132	241.7852	334.7244	−0.46925	0.000705	0.005001	Eci1
ENSMUSG00000024136	17.18002	41.12928	−1.2594	0.001857	0.011341	Dnase1l2
ENSMUSG00000024158	265.7379	466.1448	−0.81077	3.41E-09	9.35E-08	Hagh
ENSMUSG00000024180	1372.6	1732.295	−0.33577	0.000696	0.004951	Tmem8
ENSMUSG00000024181	331.4292	467.0762	−0.49496	4.87E-05	0.000499	Mrpl28
ENSMUSG00000024187	1154.623	1737.138	−0.58929	5.10E-10	1.61E-08	Itfg3
ENSMUSG00000024193	186.1346	264.7663	−0.50837	0.000818	0.005664	Phf1
ENSMUSG00000024206	23.86133	64.4602	−1.4337	2.14E-06	3.16E-05	Rfx2
ENSMUSG00000024219	307.1121	406.9503	−0.40609	0.00288	0.016255	Anks1
ENSMUSG00000024222	1020.915	2038.325	−0.99752	8.22E-25	1.23E-22	Fkbp5
ENSMUSG00000024236	386.2189	487.1917	−0.33507	0.007307	0.035187	Svil
ENSMUSG00000024247	400.8673	536.5876	−0.42069	0.000478	0.00359	Pkdcc
ENSMUSG00000024251	110.9418	165.1032	−0.57356	0.001849	0.011303	Thada
ENSMUSG00000024292	716.5898	2940.599	−2.0369	5.38E-07	9.18E-06	Cyp4f14
ENSMUSG00000024299	102.6753	151.9222	−0.56524	0.004913	0.025274	Adamts10
ENSMUSG00000024308	1349.198	2440.575	−0.85512	6.94E-08	1.45E-06	Tapbp
ENSMUSG00000024319	348.8445	474.5763	−0.44406	0.000295	0.00237	Vps52
ENSMUSG00000024338	345.3409	1280.086	−1.8901	9.40E-13	4.45E-11	Psmb8
ENSMUSG00000024339	204.0583	481.6336	−1.239	4.37E-19	4.24E-17	Tap2
ENSMUSG00000024378	1938.977	2335.693	−0.26856	0.002401	0.014005	Stard4

ENSMUSG00000024388	1157.582	1893.605	−0.71002	0.000349	0.002743	Myo7b
ENSMUSG00000024392	1266.271	1595.971	−0.33385	0.001314	0.008415	Bag6
ENSMUSG00000024393	2141.062	2837.431	−0.40626	1.34E-05	0.000159	Prrc2a
ENSMUSG00000024395	94.08812	158.8533	−0.75561	2.32E-05	0.000258	Lims2
ENSMUSG00000024411	5245.669	6655.112	−0.34334	0.009189	0.042452	Aqp4
ENSMUSG00000024422	405.1999	526.7351	−0.37844	0.002375	0.013889	Dhx16
ENSMUSG00000024426	89.70376	149.7108	−0.73894	0.000154	0.001361	Atat1
ENSMUSG00000024431	851.1311	1143.237	−0.42567	0.000101	0.000944	Nr3c1
ENSMUSG00000024456	1183.776	1488.534	−0.3305	0.001016	0.006807	Diap1
ENSMUSG00000024457	579.9874	843.6245	−0.54058	6.28E-07	1.06E-05	Trim26
ENSMUSG00000024483	896.1419	1082.056	−0.27198	0.008563	0.040014	Ankhd1
ENSMUSG00000024548	177.2667	266.9652	−0.59073	6.46E-05	0.000636	Setbp1
ENSMUSG00000024556	1248.928	2967.854	−1.2487	1.07E-39	3.99E-37	Me2
ENSMUSG00000024561	382.8577	485.9286	−0.34394	0.004479	0.023422	Mbd1
ENSMUSG00000024589	656.5623	796.0737	−0.27797	0.006991	0.034014	Nedd4l
ENSMUSG00000024610	2228.42	10505.03	−2.237	8.17E-19	7.79E-17	Cd74
ENSMUSG00000024613	429.0732	528.9604	−0.30194	0.010718	0.048047	Tcof1
ENSMUSG00000024619	2136.642	2940.171	−0.46055	5.23E-07	8.93E-06	Cdx1
ENSMUSG00000024687	946.6702	1151.665	−0.28279	0.005723	0.028684	Osbp
ENSMUSG00000024712	2582.256	4245.226	−0.71721	7.45E-12	3.17E-10	Rfk
ENSMUSG00000024713	112.815	303.9956	−1.4301	1.88E-06	2.83E-05	Pcsk5
ENSMUSG00000024727	256.3738	897.1776	−1.8071	1.72E-23	2.42E-21	Trpm6
ENSMUSG00000024747	79.85761	152.4302	−0.93265	1.01E-06	1.60E-05	Aldh1a7
ENSMUSG00000024769	866.8721	1105.668	−0.35103	0.000749	0.00526	Cdc42bpg
ENSMUSG00000024773	380.4831	552.6177	−0.53845	3.74E-06	5.19E-05	Atg2a
ENSMUSG00000024787	157.3405	224.1319	−0.51046	0.001375	0.00876	Snx15
ENSMUSG00000024807	376.6727	595.986	−0.66197	6.80E-07	1.14E-05	Syvn1
ENSMUSG00000024816	410.4259	510.8933	−0.3159	0.007005	0.034055	Frmd8
ENSMUSG00000024817	637.4704	803.7705	−0.33443	0.00325	0.01796	Uhrf2
ENSMUSG00000024866	115.2031	179.1306	−0.63683	0.004623	0.024025	Acy3
ENSMUSG00000024875	341.0936	482.4004	−0.50006	5.82E-05	0.000583	Yif1a
ENSMUSG00000024899	1455.2	2817.99	−0.95345	1.68E-25	2.65E-23	Papss2
ENSMUSG00000024922	148.669	217.184	−0.54681	0.000888	0.006068	Ovol1
ENSMUSG00000024947	255.109	360.503	−0.4989	0.000269	0.002191	Men1
ENSMUSG00000024958	186.2134	276.605	−0.57087	0.008537	0.039952	Gpr137
ENSMUSG00000024960	2422.37	3160.052	−0.38353	1.91E-05	0.000217	Plcb3
ENSMUSG00000024966	946.0627	1203.827	−0.34762	0.000958	0.006476	Stip1
ENSMUSG00000024972	174.0367	252.0648	−0.5344	0.000377	0.002936	Lgals12
ENSMUSG00000024985	569.4704	769.9043	−0.43506	0.007059	0.034273	Tcf7l2
ENSMUSG00000024990	87.90304	132.0836	−0.58747	0.007325	0.035255	Rbp4
ENSMUSG00000025002	219.4432	2058.648	−3.2298	0.004256	0.022432	Cyp2c55
ENSMUSG00000025006	201.0107	277.3613	−0.46449	0.002074	0.012454	Sorbs1
ENSMUSG00000025025	529.7394	671.2243	−0.34151	0.001765	0.010846	Mxi1
ENSMUSG00000025037	3447.171	6935.721	−1.0086	3.67E-31	7.61E-29	Maoa
ENSMUSG00000025068	2169.553	3086.819	−0.50872	9.22E-09	2.30E-07	Gsto1
ENSMUSG00000025076	1163.106	2075.918	−0.83577	3.48E-18	3.15E-16	Casp7
ENSMUSG00000025137	691.3938	1132.922	−0.71247	0.000147	0.00131	Pcyt2

ENSMUSG00000025153	2224.203	3171.755	−0.51199	1.95E-07	3.69E-06	Fasn
ENSMUSG00000025158	179.8637	266.8592	−0.56917	8.07E-05	0.000774	Rfng
ENSMUSG00000025163	9.83954	36.82427	−1.904	2.57E-05	0.000283	Cd7
ENSMUSG00000025165	50.64575	97.8416	−0.95001	4.25E-05	0.000443	Sectm1a
ENSMUSG00000025190	392.0907	801.4265	−1.0314	2.28E-18	2.12E-16	Got1
ENSMUSG00000025195	455.1661	635.4128	−0.4813	1.54E-05	0.00018	Dnmbp
ENSMUSG00000025203	10497.33	13857.88	−0.40068	5.13E-06	6.86E-05	Scd2
ENSMUSG00000025204	826.3778	1042.234	−0.33481	0.001138	0.007473	Ndufb8
ENSMUSG00000025207	2032.131	3693.106	−0.86184	4.12E-21	4.65E-19	Sema4g
ENSMUSG00000025224	651.3104	1173.213	−0.84905	2.14E-16	1.54E-14	Gbf1
ENSMUSG00000025227	175.8514	249.8226	−0.50655	0.000924	0.00628	Tmem180
ENSMUSG00000025228	1516.85	1966.307	−0.37441	6.77E-05	0.000661	Actr1a
ENSMUSG00000025232	809.7921	1027.803	−0.34394	0.000589	0.00432	Hexa
ENSMUSG00000025237	158.2277	218.2476	−0.46396	0.00445	0.023282	Parp6
ENSMUSG00000025255	15.7509	45.1036	−1.5178	2.81E-05	0.000305	Zfhx4
ENSMUSG00000025316	85.94571	133.612	−0.63655	0.004381	0.022977	Banp
ENSMUSG00000025347	261.3524	1037.688	−1.9893	3.03E-09	8.43E-08	Mettl7b
ENSMUSG00000025348	205.6629	268.2157	−0.38311	0.005456	0.027555	Itga7
ENSMUSG00000025357	1160.971	1481.489	−0.35172	0.000276	0.002237	Dgka
ENSMUSG00000025377	141.0019	244.9036	−0.7965	1.10E-05	0.000135	Enthd2
ENSMUSG00000025393	15084.63	17827.7	−0.24104	0.004087	0.021677	Atp5b
ENSMUSG00000025401	3946.909	6716.157	−0.76691	0.000129	0.001169	Myo1a
ENSMUSG00000025407	44.49956	82.1178	−0.8839	0.00046	0.003482	Gli1
ENSMUSG00000025409	481.8798	624.5734	−0.3742	0.000989	0.006656	Mbd6
ENSMUSG00000025433	5.18717	20.51648	−1.9838	0.000375	0.00293	Crisp3
ENSMUSG00000025453	143.3571	197.0147	−0.45869	0.004617	0.024004	Nnt
ENSMUSG00000025465	1005.274	1244.977	−0.30853	0.001596	0.009957	Echs1
ENSMUSG00000025466	223.3588	310.6832	−0.47608	0.002841	0.01608	Fuom
ENSMUSG00000025477	425.537	521.4163	−0.29315	0.010897	0.048683	Inpp5a
ENSMUSG00000025479	32.50662	58.45615	−0.84662	0.00273	0.015556	Cyp2e1
ENSMUSG00000025494	225.3558	311.3548	−0.46635	0.001021	0.00683	Sigirr
ENSMUSG00000025495	210.2242	364.668	−0.79465	6.58E-09	1.68E-07	Ptdss2
ENSMUSG00000025497	3281.573	6137.279	−0.90321	6.20E-06	8.11E-05	Cdhr5
ENSMUSG00000025503	1948.166	2641.197	−0.43908	5.09E-06	6.81E-05	Taldo1
ENSMUSG00000025504	1321.401	2283.431	−0.78913	9.27E-09	2.31E-07	Eps8l2
ENSMUSG00000025505	98.46667	141.0572	−0.51857	0.005047	0.02583	Tmem80
ENSMUSG00000025509	890.0801	1162.147	−0.38479	0.00046	0.003482	Pnpla2
ENSMUSG00000025512	285.2397	429.4369	−0.59027	5.53E-06	7.34E-05	Chid1
ENSMUSG00000025515	19887.92	52437.64	−1.3987	4.22E-34	9.77E-32	Muc2
ENSMUSG00000025545	181.2072	277.6991	−0.61588	1.46E-05	0.000172	Clybl
ENSMUSG00000025583	343.1226	462.6934	−0.43133	0.000569	0.00419	Rptor
ENSMUSG00000025651	1931.262	2715.05	−0.49144	8.59E-08	1.76E-06	Uqcrc1
ENSMUSG00000025733	232.6313	444.4874	−0.9341	2.48E-06	3.62E-05	Rhot2
ENSMUSG00000025736	165.1897	237.6433	−0.52467	0.000533	0.003956	Jmjd8
ENSMUSG00000025737	227.4427	296.6761	−0.38339	0.004726	0.024468	Wdr24
ENSMUSG00000025745	2461.914	2984.617	−0.27777	0.001737	0.010721	Hadha
ENSMUSG00000025780	1426.447	1804.687	−0.33932	0.001277	0.008219	Itih5

ENSMUSG00000025792	1056.015	1600.432	−0.59983	0.001743	0.010741	Slc25a10
ENSMUSG00000025793	585.1741	735.7595	−0.33037	0.00251	0.014526	Hgs
ENSMUSG00000025812	350.0055	471.4491	−0.42972	0.000411	0.00316	Pard3
ENSMUSG00000025823	1583.449	2043.4	−0.3679	0.000145	0.001297	Pdia4
ENSMUSG00000025868	742.9791	927.0511	−0.31933	0.004748	0.024561	Higd2a
ENSMUSG00000025871	987.4694	1353.207	−0.45457	4.42E-06	6.00E-05	4833439L19Rik
ENSMUSG00000025885	1599.645	2164.944	−0.43658	3.97E-06	5.47E-05	Myo5b
ENSMUSG00000025888	946.4143	1487.298	−0.65215	4.29E-11	1.59E-09	Casp1
ENSMUSG00000025964	82.66324	170.766	−1.0467	1.71E-08	4.02E-07	Adam23
ENSMUSG00000025969	540.7593	858.7661	−0.66728	1.89E-09	5.44E-08	Nrp2
ENSMUSG00000025980	2562.821	3641.119	−0.50665	1.31E-08	3.17E-07	Hspd1
ENSMUSG00000026027	118.3017	187.7895	−0.66665	6.49E-05	0.000639	Stradb
ENSMUSG00000026062	931.5795	2448.756	−1.3943	4.62E-14	2.57E-12	Slc9a2
ENSMUSG00000026077	181.9909	239.5059	−0.39619	0.010438	0.047062	Npas2
ENSMUSG00000026090	384.7237	534.3294	−0.47391	5.84E-05	0.000585	2010300C02Rik
ENSMUSG00000026104	547.9685	1510.477	−1.4628	1.96E-45	9.47E-43	Stat1
ENSMUSG00000026112	873.539	1095.497	−0.32664	0.000911	0.006199	Coa5
ENSMUSG00000026123	2841.012	3994.281	−0.49153	2.38E-08	5.44E-07	Plekhb2
ENSMUSG00000026172	67.91716	102.6854	−0.59638	0.006036	0.029964	Bcs1l
ENSMUSG00000026175	5333.789	8269.187	−0.63258	0.000276	0.002237	Vil1
ENSMUSG00000026198	204.5474	359.3753	−0.81306	2.77E-09	7.80E-08	Abcb6
ENSMUSG00000026199	202.4467	272.476	−0.42859	0.00379	0.020471	Ankzf1
ENSMUSG00000026201	351.4416	580.4748	−0.72395	2.68E-09	7.56E-08	Stk16
ENSMUSG00000026207	82.23895	140.9167	−0.77695	3.70E-05	0.000391	Speg
ENSMUSG00000026208	4225.957	6064.353	−0.52108	7.65E-08	1.59E-06	Des
ENSMUSG00000026260	1069.199	1588.211	−0.57087	5.36E-09	1.42E-07	Ndufa10
ENSMUSG00000026269	772.7824	1026.104	−0.40904	4.01E-05	0.000421	Rnpepl1
ENSMUSG00000026271	214.0802	318.498	−0.57313	6.24E-05	0.000618	Gpr35
ENSMUSG00000026307	124.8723	191.9493	−0.62027	0.00018	0.001564	Scly
ENSMUSG00000026321	707.7971	990.7707	−0.48522	8.81E-06	0.00011	Tnfrsf11a
ENSMUSG00000026322	87.12624	154.9657	−0.83077	1.33E-05	0.000158	Htr4
ENSMUSG00000026349	592.2271	733.3716	−0.30839	0.007033	0.034172	Ccnt2
ENSMUSG00000026380	3407.327	5494.196	−0.68927	6.71E-15	4.14E-13	Tfcp2l1
ENSMUSG00000026389	153.4055	354.7265	−1.2094	5.19E-17	4.08E-15	Steap3
ENSMUSG00000026398	196.8424	493.0264	−1.3246	1.25E-16	9.29E-15	Nr5a2
ENSMUSG00000026447	562.0511	900.9861	−0.6808	6.79E-08	1.43E-06	Pik3c2b
ENSMUSG00000026463	550.4485	807.7388	−0.55328	2.24E-07	4.20E-06	Atp2b4
ENSMUSG00000026469	602.5954	1002.897	−0.73491	8.88E-12	3.72E-10	Xpr1
ENSMUSG00000026483	1269.02	1580.485	−0.31665	0.000646	0.004663	Fam129a
ENSMUSG00000026490	371.3418	474.9992	−0.35518	0.003019	0.016922	Cdc42bpa
ENSMUSG00000026519	945.7544	1347.404	−0.51065	2.07E-07	3.89E-06	Tmem63a
ENSMUSG00000026531	288.9786	1411.084	−2.2878	2.05E-41	7.95E-39	Mptx1
ENSMUSG00000026546	19.0722	37.38812	−0.97111	0.00827	0.038963	Cfap45
ENSMUSG00000026547	2325.575	2743.685	−0.23853	0.009826	0.044791	Tagln2
ENSMUSG00000026553	3179.959	3738.152	−0.23332	0.010693	0.047995	Copa
ENSMUSG00000026573	2.009652	10.7783	−2.4231	0.003341	0.018374	Xcl1
ENSMUSG00000026604	124.3832	176.5671	−0.50543	0.004154	0.022001	Ptpn14

ENSMUSG00000026610	36.76085	85.81979	−1.2231	1.74E-06	2.65E-05	Esrrg
ENSMUSG00000026617	676.38	1078.423	−0.67302	2.74E-08	6.19E-07	Bpnt1
ENSMUSG00000026664	844.0845	1094.373	−0.37464	9.39E-05	0.000884	Phyh
ENSMUSG00000026672	367.5575	516.4548	−0.49067	0.000276	0.002238	Optn
ENSMUSG00000026674	277.1876	412.2488	−0.57265	5.56E-06	7.37E-05	Ddr2
ENSMUSG00000026687	602.6623	825.5086	−0.45393	0.007144	0.034578	Aldh9a1
ENSMUSG00000026688	2508.291	3344.878	−0.41525	1.62E-06	2.48E-05	Mgst3
ENSMUSG00000026698	192.281	255.7375	−0.41145	0.007522	0.036039	Pigc
ENSMUSG00000026701	8248.242	11676.02	−0.50139	3.93E-09	1.07E-07	Prdx6
ENSMUSG00000026768	41.9937	79.68905	−0.92421	0.000212	0.001793	Itga8
ENSMUSG00000026784	292.5086	393.5387	−0.42803	0.001172	0.007658	Pdss1
ENSMUSG00000026798	57.70582	95.37542	−0.7249	0.001862	0.011362	Coq4
ENSMUSG00000026834	53.35352	87.32251	−0.71077	0.002724	0.015529	Acvr1c
ENSMUSG00000026853	345.6532	474.6081	−0.45741	0.000115	0.001055	Crat
ENSMUSG00000026858	200.0915	357.129	−0.83579	7.71E-09	1.95E-07	Fam73b
ENSMUSG00000026864	7091.943	8260.514	−0.22005	0.010131	0.045981	Hspa5
ENSMUSG00000026870	517.263	651.9374	−0.33384	0.00308	0.017166	Cutal
ENSMUSG00000026893	116.8146	176.1173	−0.59232	0.000999	0.00672	Gca
ENSMUSG00000026895	699.522	1010.985	−0.53132	3.88E-07	6.83E-06	Ndufa8
ENSMUSG00000026896	626.5077	862.5515	−0.46128	1.42E-05	0.000167	Ifih1
ENSMUSG00000026915	894.4897	1096.669	−0.29399	0.003741	0.020234	Strbp
ENSMUSG00000026924	945.528	1227.846	−0.37694	0.000252	0.002075	Sec16a
ENSMUSG00000026932	359.9834	461.5535	−0.35857	0.005366	0.027166	Nacc2
ENSMUSG00000026979	546.2285	845.9849	−0.63113	5.84E-08	1.24E-06	Psd4
ENSMUSG00000026980	225.1069	510.9441	−1.1826	1.60E-14	9.54E-13	Ly75
ENSMUSG00000027015	17.10818	38.96646	−1.1875	0.002131	0.012698	Cybrd1
ENSMUSG00000027078	182.6171	317.1074	−0.79615	0.001439	0.009115	Ube2l6
ENSMUSG00000027086	153.3459	265.0608	−0.78953	0.000232	0.001946	Fastkd1
ENSMUSG00000027130	337.6464	439.7851	−0.38129	0.00127	0.008185	Slc12a6
ENSMUSG00000027185	232.2124	313.9444	−0.43506	0.002355	0.013789	Nat10
ENSMUSG00000027187	411.8222	594.7645	−0.5303	6.77E-06	8.75E-05	Cat
ENSMUSG00000027217	71.79957	116.7728	−0.70166	0.001214	0.007872	Tspan18
ENSMUSG00000027227	802.9663	1228.501	−0.61349	4.23E-09	1.15E-07	Sord
ENSMUSG00000027230	1351.988	1854.414	−0.45588	6.51E-07	1.09E-05	Creb3l1
ENSMUSG00000027254	101.0042	139.3057	−0.46384	0.010296	0.046588	Map1a
ENSMUSG00000027263	195.6865	291.038	−0.57266	0.000122	0.001116	Tubgcp4
ENSMUSG00000027274	322.027	442.9363	−0.45992	0.000235	0.00196	Mkks
ENSMUSG00000027282	1463.791	1828.596	−0.32103	0.00071	0.005037	Mtch2
ENSMUSG00000027296	25.17301	84.02119	−1.7389	1.04E-05	0.000128	Itpka
ENSMUSG00000027309	192.5354	268.2977	−0.47871	0.001255	0.008098	4930402H24Rik
ENSMUSG00000027315	1678.427	2079.744	−0.3093	0.003217	0.017803	Spint1
ENSMUSG00000027317	680.4103	1046.546	−0.62116	1.72E-09	5.02E-08	Ppp1r14d
ENSMUSG00000027318	45.85683	114.2304	−1.3167	2.25E-08	5.17E-07	Adam33
ENSMUSG00000027329	76.36057	134.7037	−0.81889	8.13E-05	0.000779	Spef1
ENSMUSG00000027332	424.4988	582.659	−0.45689	8.74E-05	0.000828	Ivd
ENSMUSG00000027367	1113.012	1360.364	−0.28952	0.002332	0.013684	Stard7
ENSMUSG00000027377	1186.798	1763.057	−0.571	4.28E-05	0.000446	Mall

ENSMUSG00000027397	850.0747	2080.226	−1.2911	3.87E-39	1.34E-36	Slc20a1
ENSMUSG00000027401	673.5828	2401.056	−1.8337	6.84E-29	1.23E-26	Tgm3
ENSMUSG00000027406	1409.768	1950.49	−0.46838	7.06E-07	1.18E-05	Idh3b
ENSMUSG00000027422	6183.257	7669.707	−0.3108	0.000228	0.001911	Rrbp1
ENSMUSG00000027457	24.51369	53.29553	−1.1204	0.000164	0.00144	Snph
ENSMUSG00000027466	691.9283	890.7389	−0.36438	0.000815	0.005649	Rbck1
ENSMUSG00000027496	252.5505	331.3157	−0.39163	0.007692	0.036724	Aurka
ENSMUSG00000027513	310.753	816.78	−1.3942	2.77E-32	6.19E-30	Pck1
ENSMUSG00000027514	356.6414	675.1937	−0.92083	5.36E-14	2.95E-12	Zbp1
ENSMUSG00000027524	76.12584	194.7645	−1.3553	3.67E-13	1.83E-11	Edn3
ENSMUSG00000027555	226.0788	341.6013	−0.59549	8.55E-06	0.000108	Car13
ENSMUSG00000027556	2228.82	16787.79	−2.9131	0.000652	0.004695	Car1
ENSMUSG00000027559	125.7723	372.242	−1.5654	2.77E-26	4.49E-24	Car3
ENSMUSG00000027562	2687.828	4332.152	−0.68864	1.08E-07	2.14E-06	Car2
ENSMUSG00000027580	727.9898	1167.719	−0.6817	1.28E-10	4.47E-09	Helz2
ENSMUSG00000027582	170.8012	238.6119	−0.48235	0.002531	0.014621	Zgpat
ENSMUSG00000027605	775.1116	970.126	−0.32377	0.001872	0.011398	Acss2
ENSMUSG00000027624	327.4948	461.8938	−0.49609	5.22E-05	0.00053	Epb4.1l1
ENSMUSG00000027634	407.6975	642.5296	−0.65626	1.45E-08	3.47E-07	Ndrg3
ENSMUSG00000027639	600.7802	1224.605	−1.0274	2.68E-23	3.65E-21	Samhd1
ENSMUSG00000027641	316.2576	421.2445	−0.41356	0.00387	0.020844	Rbl1
ENSMUSG00000027652	947.2888	1187.493	−0.32604	0.001063	0.007067	Ralgapb
ENSMUSG00000027678	393.6053	592.1776	−0.58928	1.34E-06	2.09E-05	Ncoa3
ENSMUSG00000027695	717.6473	1138.923	−0.66632	7.07E-11	2.55E-09	Pld1
ENSMUSG00000027761	59.31059	275.8759	−2.2177	6.69E-10	2.07E-08	Aadac
ENSMUSG00000027765	212.5792	303.8031	−0.51514	0.000139	0.001251	P2ry1
ENSMUSG00000027774	525.3996	682.4793	−0.37737	0.000843	0.005806	Gfm1
ENSMUSG00000027797	111.1768	168.0155	−0.59574	0.000523	0.003894	Dclk1
ENSMUSG00000027840	42.92656	118.8705	−1.4694	1.21E-07	2.39E-06	Wnt2b
ENSMUSG00000027870	384.9904	1612.515	−2.0664	3.41E-29	6.21E-27	Hao2
ENSMUSG00000027875	465.913	1562.684	−1.7459	0.004069	0.021601	Hmgcs2
ENSMUSG00000027881	905.61	1096.366	−0.27577	0.011088	0.049358	Prpf38b
ENSMUSG00000027889	260.7064	335.6675	−0.36461	0.007095	0.03437	Ampd2
ENSMUSG00000027933	469.7764	713.0409	−0.60201	1.97E-07	3.72E-06	Ints3
ENSMUSG00000027984	1652.451	2392.162	−0.53371	7.91E-08	1.64E-06	Hadh
ENSMUSG00000027993	1213.379	1936.356	−0.67431	2.49E-12	1.13E-10	Trim2
ENSMUSG00000027995	293.8269	372.8108	−0.34348	0.010985	0.048986	Tlr2
ENSMUSG00000028003	11.47738	71.96156	−2.6484	6.28E-15	3.91E-13	Lrat
ENSMUSG00000028013	408.539	570.1904	−0.48097	2.72E-05	0.000297	Ppa2
ENSMUSG00000028028	305.748	424.1181	−0.47212	0.000357	0.002798	Alpk1
ENSMUSG00000028034	851.1553	1074.541	−0.33623	0.003121	0.017355	Fubp1
ENSMUSG00000028039	18.41981	46.138	−1.3247	0.000869	0.005952	Efna3
ENSMUSG00000028042	1590.226	2048.227	−0.36514	0.000836	0.005774	Zbtb7b
ENSMUSG00000028063	2503.135	2990.959	−0.25687	0.007356	0.035368	Lmna
ENSMUSG00000028064	1320.644	1852.955	−0.48859	1.04E-05	0.000128	Sema4a
ENSMUSG00000028082	917.3198	1169.234	−0.35007	0.000403	0.003113	Sh3d19
ENSMUSG00000028096	303.509	405.9222	−0.41946	0.000886	0.006054	Gpr89

ENSMUSG00000028099	422.503	545.0462	−0.36742	0.001574	0.009842	Polr3c
ENSMUSG00000028107	242.7179	338.0508	−0.47796	0.000797	0.005543	Tars2
ENSMUSG00000028124	684.1496	867.1293	−0.34194	0.00205	0.012335	Gclm
ENSMUSG00000028132	280.5545	365.238	−0.38056	0.003105	0.017282	Tmem56
ENSMUSG00000028136	364.0995	475.5157	−0.38516	0.003104	0.017282	Snx27
ENSMUSG00000028137	24.61186	48.68019	−0.98398	0.002628	0.015086	Celf3
ENSMUSG00000028150	263.225	432.1527	−0.71524	2.45E-08	5.60E-07	Rorc
ENSMUSG00000028179	296.9384	664.6639	−1.1625	1.87E-22	2.45E-20	Cth
ENSMUSG00000028248	916.4202	1344.667	−0.55317	5.65E-08	1.21E-06	Pnisr
ENSMUSG00000028255	7478.466	16975.58	−1.1826	3.70E-34	8.69E-32	Clca1
ENSMUSG00000028268	140.5709	207.4177	−0.56124	0.000456	0.003455	Gbp3
ENSMUSG00000028270	211.1694	681.2626	−1.6898	5.33E-42	2.16E-39	Gbp2
ENSMUSG00000028289	50.17598	100.6567	−1.0044	9.40E-06	0.000117	Epha7
ENSMUSG00000028293	1115.25	1351.502	−0.2772	0.008541	0.039952	Slc35a1
ENSMUSG00000028367	8675.454	11988.54	−0.46665	5.67E-08	1.21E-06	Txn1
ENSMUSG00000028392	1290.678	1545.117	−0.25959	0.006387	0.031469	Bspry
ENSMUSG00000028399	603.0982	782.7195	−0.3761	0.000593	0.00434	Ptprd
ENSMUSG00000028402	71.70147	117.7547	−0.71571	0.000837	0.005779	Mpdz
ENSMUSG00000028438	56.13292	90.06197	−0.68207	0.002099	0.012569	Kif24
ENSMUSG00000028453	61.3726	130.2903	−1.0861	2.63E-07	4.86E-06	Fancg
ENSMUSG00000028454	250.2672	391.3106	−0.64484	1.21E-06	1.91E-05	Pigo
ENSMUSG00000028457	9.630818	29.95768	−1.6372	0.000355	0.002789	Atp8b5
ENSMUSG00000028463	596.2857	745.4574	−0.32212	0.002906	0.016379	Car9
ENSMUSG00000028464	2026.526	2896.991	−0.51555	5.68E-09	1.49E-07	Tpm2
ENSMUSG00000028465	1681.255	2163.004	−0.3635	9.60E-05	0.0009	Tln1
ENSMUSG00000028476	46.6196	71.71974	−0.62143	0.009065	0.04203	Reck
ENSMUSG00000028479	3138.063	4115.094	−0.39105	8.61E-06	0.000108	Gne
ENSMUSG00000028488	128.3377	236.642	−0.88276	9.04E-08	1.84E-06	Sh3gl2
ENSMUSG00000028521	301.2466	535.3193	−0.82945	0.000779	0.005445	Slc35d1
ENSMUSG00000028522	889.4049	1270.613	−0.51461	8.11E-08	1.67E-06	Mier1
ENSMUSG00000028532	282.2317	380.068	−0.42938	0.001001	0.006729	Cachd1
ENSMUSG00000028538	357.0707	483.3807	−0.43695	0.000207	0.001763	St3gal3
ENSMUSG00000028607	571.9152	787.7801	−0.46199	1.89E-05	0.000216	Cpt2
ENSMUSG00000028614	263.7139	358.5917	−0.44337	0.001026	0.006855	Ndc1
ENSMUSG00000028617	829.7558	1071.242	−0.36853	0.000923	0.006276	Lrrc42
ENSMUSG00000028635	21.80612	52.01559	−1.2542	0.005394	0.027287	Edn2
ENSMUSG00000028648	473.1698	637.418	−0.42988	0.000625	0.004537	Ndufs5
ENSMUSG00000028672	458.1613	623.4717	−0.44447	0.000123	0.001121	Hmgcl
ENSMUSG00000028690	71.11389	104.4921	−0.55519	0.006887	0.033601	Mmachc
ENSMUSG00000028792	2092.636	2548.515	−0.28434	0.001455	0.009208	Ak2
ENSMUSG00000028803	584.3136	817.6077	−0.48467	1.55E-05	0.000181	Nipal3
ENSMUSG00000028811	498.0995	715.6823	−0.52289	3.00E-06	4.27E-05	Yars
ENSMUSG00000028830	720.9569	1025.593	−0.50847	0.000681	0.004862	AU040320
ENSMUSG00000028842	175.8447	231.8402	−0.39883	0.010133	0.045981	Ago3
ENSMUSG00000028854	473.4487	708.0168	−0.58058	1.36E-07	2.65E-06	Slc9a1
ENSMUSG00000028890	278.7478	393.903	−0.49888	0.000117	0.001072	Mtf1
ENSMUSG00000028899	182.4996	237.3761	−0.37928	0.0094	0.04327	Taf12

ENSMUSG00000028911	533.3744	679.4245	−0.34916	0.003636	0.01975	Srsf4
ENSMUSG00000028919	161.2034	273.766	−0.76406	0.009389	0.043232	Arhgef19
ENSMUSG00000028927	32.10848	64.79665	−1.013	0.000172	0.001503	Padi2
ENSMUSG00000028937	702.1574	899.3348	−0.35706	0.000576	0.004243	Acot7
ENSMUSG00000028943	317.847	804.2192	−1.3393	0.000728	0.005149	Espn
ENSMUSG00000028944	251.1339	340.0171	−0.43715	0.000702	0.004989	Prkag2
ENSMUSG00000028959	573.6445	803.8918	−0.48684	6.04E-06	7.94E-05	Fastk
ENSMUSG00000028960	581.09	739.6906	−0.34816	0.001568	0.00981	Ube4b
ENSMUSG00000028962	772.8625	1665.428	−1.1076	2.33E-11	9.09E-10	Slc4a2
ENSMUSG00000028977	806.4001	1020.398	−0.33956	0.001477	0.009324	Casz1
ENSMUSG00000028991	478.0168	594.5695	−0.31478	0.006443	0.03173	Mtor
ENSMUSG00000028992	56.72029	101.855	−0.84458	0.000131	0.001187	Nmnat1
ENSMUSG00000028995	187.4449	281.395	−0.58613	2.83E-05	0.000307	Fam126a
ENSMUSG00000029020	960.3465	1487.86	−0.63161	2.52E-10	8.40E-09	Mfn2
ENSMUSG00000029032	1030.091	1228.944	−0.25465	0.009605	0.043997	Arhgef16
ENSMUSG00000029053	329.6291	665.0105	−1.0125	2.17E-12	9.91E-11	Prkcz
ENSMUSG00000029055	313.0167	490.6153	−0.64835	2.23E-07	4.18E-06	Plch2
ENSMUSG00000029060	143.9515	290.4128	−1.0125	4.34E-11	1.61E-09	Mib2
ENSMUSG00000029062	635.0246	825.2087	−0.37795	0.000809	0.005618	Cdk11b
ENSMUSG00000029063	1514.932	2067.238	−0.44845	1.68E-06	2.56E-05	Nadk
ENSMUSG00000029068	1147.681	1613.94	−0.49187	7.69E-07	1.27E-05	Ccnl2
ENSMUSG00000029074	38.07252	72.96356	−0.93843	0.000249	0.002062	Ttll10
ENSMUSG00000029095	37.67451	74.04111	−0.97474	0.000385	0.002988	Ablim2
ENSMUSG00000029098	415.7046	527.6315	−0.34397	0.002854	0.016135	Acox3
ENSMUSG00000029104	596.124	743.7793	−0.31926	0.00471	0.024406	Htt
ENSMUSG00000029106	1946.784	2323.268	−0.25506	0.005024	0.025735	Add1
ENSMUSG00000029153	653.5076	1578.089	−1.2719	9.62E-36	2.45E-33	Ociad2
ENSMUSG00000029163	431.9447	545.3552	−0.33635	0.00369	0.019979	Emilin1
ENSMUSG00000029166	57.89526	198.2778	−1.776	6.71E-12	2.88E-10	Mapre3
ENSMUSG00000029167	250.0184	401.6569	−0.68393	1.23E-07	2.42E-06	Ppargc1a
ENSMUSG00000029173	316.5855	468.4173	−0.5652	7.05E-06	9.09E-05	Sepsecs
ENSMUSG00000029185	527.5654	714.7005	−0.43799	6.84E-05	0.000668	Fam114a1
ENSMUSG00000029189	998.5944	1360.701	−0.44638	6.12E-06	8.02E-05	Sel1l3
ENSMUSG00000029190	640.0682	807.6909	−0.33558	0.00237	0.013869	D5Ertd579e
ENSMUSG00000029201	806.0193	2258.151	−1.4863	1.49E-22	1.98E-20	Ugdh
ENSMUSG00000029228	115.6662	158.074	−0.45063	0.009544	0.043754	Lnx1
ENSMUSG00000029260	2142.762	3420.831	−0.67488	4.62E-14	2.57E-12	Ugt2b34
ENSMUSG00000029263	193.54	260.6694	−0.42959	0.003953	0.021158	Pigg
ENSMUSG00000029269	1358.052	1830.54	−0.43073	0.001726	0.010664	Sult1b1
ENSMUSG00000029273	963.5531	1863.665	−0.95171	2.62E-23	3.60E-21	Sult1d1
ENSMUSG00000029311	1194.329	1463.019	−0.29275	0.004108	0.02177	Hsd17b11
ENSMUSG00000029313	655.2187	831.1589	−0.34315	0.001467	0.009267	Aff1
ENSMUSG00000029326	139.1818	209.9475	−0.59306	0.000585	0.004297	Enoph1
ENSMUSG00000029330	1737.703	2350.331	−0.43568	1.80E-06	2.73E-05	Cds1
ENSMUSG00000029333	231.1043	365.8088	−0.66255	1.96E-06	2.95E-05	Rasgef1b
ENSMUSG00000029361	77.84789	123.2792	−0.6632	0.000553	0.004089	Nos1
ENSMUSG00000029381	1359.523	2049.941	−0.59248	2.87E-10	9.42E-09	Shroom3

ENSMUSG00000029385	1045.607	1251.637	−0.25948	0.010309	0.046624	Ccng2
ENSMUSG00000029413	140.1079	259.5489	−0.88947	6.48E-09	1.66E-07	Naaa
ENSMUSG00000029417	4.01925	126.5932	−4.9771	6.18E-46	3.06E-43	Cxcl9
ENSMUSG00000029427	331.0577	465.5397	−0.49182	9.42E-05	0.000886	Zcchc8
ENSMUSG00000029436	57.699	85.88409	−0.57384	0.010024	0.045613	Mmp17
ENSMUSG00000029445	287.588	382.6106	−0.41187	0.000706	0.00501	Hpd
ENSMUSG00000029455	3312.129	4353.267	−0.39434	7.97E-06	0.000102	Aldh2
ENSMUSG00000029482	974.224	1266.742	−0.3788	0.00012	0.001098	Aacs
ENSMUSG00000029490	56.74676	125.8401	−1.149	7.58E-08	1.58E-06	Mfsd7a
ENSMUSG00000029502	662.2704	806.8399	−0.28486	0.00707	0.034296	Golga3
ENSMUSG00000029505	793.6431	988.9327	−0.31738	0.002648	0.015178	Ep400
ENSMUSG00000029516	176.2951	259.9287	−0.56012	0.00022	0.001856	Cit
ENSMUSG00000029521	113.7477	163.0496	−0.51947	0.004245	0.022395	Chek2
ENSMUSG00000029528	1251.732	1490.839	−0.2522	0.009683	0.044299	Pxn
ENSMUSG00000029535	244.1334	322.7016	−0.40253	0.003502	0.019112	Triap1
ENSMUSG00000029545	741.3067	1277.698	−0.7854	1.11E-14	6.76E-13	Acads
ENSMUSG00000029547	477.0064	704.1971	−0.56197	1.58E-06	2.44E-05	Ints1
ENSMUSG00000029556	165.6789	288.5411	−0.80039	6.86E-08	1.44E-06	Hnf1a
ENSMUSG00000029561	454.2862	1047.173	−1.2048	1.56E-17	1.31E-15	Oasl2
ENSMUSG00000029563	80.53617	136.099	−0.75695	6.56E-05	0.000645	Foxp2
ENSMUSG00000029577	856.5085	1096.253	−0.35604	0.000309	0.002465	Ube3b
ENSMUSG00000029640	1037.215	1229.702	−0.24559	0.011163	0.049593	Usp12
ENSMUSG00000029646	610.6479	1644.296	−1.4291	1.20E-16	8.99E-15	Cdx2
ENSMUSG00000029647	473.6397	614.0635	−0.3746	0.002305	0.013569	Pan3
ENSMUSG00000029657	250.3709	682.8546	−1.4475	4.33E-32	9.43E-30	Hsph1
ENSMUSG00000029673	227.4231	305.159	−0.42418	0.002082	0.012493	Auts2
ENSMUSG00000029718	199.3991	291.3866	−0.54728	0.000149	0.001322	Pcolce
ENSMUSG00000029723	500.1108	768.6278	−0.62004	2.73E-08	6.18E-07	Tsc22d4
ENSMUSG00000029727	742.6371	1653.921	−1.1552	5.89E-05	0.000589	Cyp3a13
ENSMUSG00000029729	451.5917	578.2306	−0.35663	0.002854	0.016135	Zkscan1
ENSMUSG00000029772	1392.375	4564.827	−1.713	1.66E-75	1.75E-72	Ahcyl2
ENSMUSG00000029776	490.0085	671.2059	−0.45395	5.18E-05	0.000527	Hibadh
ENSMUSG00000029797	8.554088	28.24528	−1.7233	0.000281	0.002276	Sspo
ENSMUSG00000029798	132.0169	204.3375	−0.63023	7.80E-05	0.000751	Herc6
ENSMUSG00000029817	682.9039	903.1588	−0.4033	0.000183	0.001586	Tra2a
ENSMUSG00000029922	1466.05	2398.366	−0.71012	3.95E-05	0.000415	Mkrn1
ENSMUSG00000030036	462.7816	655.0274	−0.50122	2.08E-05	0.000234	Mogs
ENSMUSG00000030058	1547.275	1926.527	−0.31627	0.000886	0.006054	Copg1
ENSMUSG00000030104	1373.324	1867.554	−0.44348	3.85E-06	5.33E-05	Edem1
ENSMUSG00000030120	1446.207	1954.763	−0.43472	3.85E-06	5.33E-05	Mlf2
ENSMUSG00000030157	364.8347	660.3249	−0.85593	1.49E-07	2.88E-06	Clec2d
ENSMUSG00000030168	1370.811	1820.015	−0.40892	1.17E-05	0.000141	Adipor2
ENSMUSG00000030189	1835.495	2429.883	−0.40472	1.34E-05	0.000159	Ybx3
ENSMUSG00000030201	1112.205	1487.754	−0.41971	2.57E-05	0.000283	Lrp6
ENSMUSG00000030213	542.9192	687.4947	−0.34061	0.00338	0.018538	Atf7ip
ENSMUSG00000030231	586.0828	738.0875	−0.33269	0.004353	0.022864	Plekha5
ENSMUSG00000030257	50.20225	89.21916	−0.8296	0.000468	0.003527	Srgap3

ENSMUSG00000030276	20.87316	51.97082	−1.3161	7.60E-05	0.000733	Ttll3
ENSMUSG00000030281	571.858	1122.225	−0.97263	7.92E-09	1.99E-07	Il17rc
ENSMUSG00000030287	201.272	298.7693	−0.56989	3.94E-05	0.000414	Itpr2
ENSMUSG00000030303	42.37244	84.059	−0.98828	0.00124	0.008022	Far2
ENSMUSG00000030306	83.10692	143.0147	−0.78312	5.62E-05	0.000565	Tmtc1
ENSMUSG00000030313	160.113	210.5004	−0.39473	0.010744	0.048128	Dennd5b
ENSMUSG00000030335	603.2558	880.0726	−0.54485	9.81E-07	1.57E-05	Mrpl51
ENSMUSG00000030340	702.9456	1248.088	−0.82823	5.47E-14	3.00E-12	Scnn1a
ENSMUSG00000030357	2763.264	3441.472	−0.31665	0.000598	0.004369	Fkbp4
ENSMUSG00000030451	884.1937	1106.986	−0.3242	0.001345	0.008595	Herc2
ENSMUSG00000030533	388.711	540.5123	−0.47563	6.94E-05	0.000676	Unc45a
ENSMUSG00000030539	435.9846	683.1045	−0.64783	3.08E-08	6.89E-07	Sema4b
ENSMUSG00000030545	142.5226	239.0437	−0.74608	3.72E-06	5.18E-05	Pex11a
ENSMUSG00000030546	3.693097	22.50746	−2.6075	1.64E-05	0.000191	Plin1
ENSMUSG00000030554	1193.144	1788.171	−0.58372	7.36E-10	2.25E-08	Synm
ENSMUSG00000030583	822.7121	1056.311	−0.36057	0.004791	0.02472	Sipa1l3
ENSMUSG00000030587	1610.229	2457.816	−0.61011	0.009144	0.042289	2200002D01Rik
ENSMUSG00000030590	288.882	447.5816	−0.63167	0.001262	0.00814	Fam98c
ENSMUSG00000030650	1358.908	1647.675	−0.27798	0.001954	0.011832	Tmc5
ENSMUSG00000030654	1958.645	2457.691	−0.32745	0.000261	0.002137	Arl6ip1
ENSMUSG00000030655	1695.845	2205.257	−0.37894	4.46E-05	0.000461	Smg1
ENSMUSG00000030657	27.39133	54.01136	−0.97954	0.004378	0.022976	Xylt1
ENSMUSG00000030681	3044.756	3632.562	−0.25466	0.003379	0.018538	Mvp
ENSMUSG00000030688	3634.933	4469.188	−0.29808	0.00059	0.004327	Stard10
ENSMUSG00000030691	368.0522	596.1438	−0.69575	5.78E-09	1.51E-07	Fchsd2
ENSMUSG00000030711	259.2637	1067.444	−2.0417	1.44E-70	1.23E-67	Sult1a1
ENSMUSG00000030714	136.2971	227.0717	−0.73639	6.10E-06	8.00E-05	Ccdc101
ENSMUSG00000030737	101.6373	194.9106	−0.93938	2.04E-08	4.73E-07	Slco2b1
ENSMUSG00000030739	3828.773	7809.927	−1.0284	1.59E-31	3.42E-29	Myh14
ENSMUSG00000030741	287.3683	393.0163	−0.45169	0.001753	0.010784	Spns1
ENSMUSG00000030762	851.0565	4333.237	−2.3481	########	########	Aqp8
ENSMUSG00000030766	367.0682	563.2288	−0.61767	2.61E-07	4.83E-06	Arhgap17
ENSMUSG00000030771	75.09459	127.8433	−0.7676	0.000151	0.001341	Micalcl
ENSMUSG00000030780	168.8564	235.8874	−0.4823	0.001073	0.007128	BC017158
ENSMUSG00000030782	201.4998	283.4944	−0.49254	0.000469	0.003533	Tgfb1i1
ENSMUSG00000030822	381.0196	486.2221	−0.35175	0.006341	0.031307	Prr14
ENSMUSG00000030824	2491.58	2904.221	−0.22109	0.010901	0.048683	Nucb1
ENSMUSG00000030826	360.1916	475.5571	−0.40085	0.002703	0.01543	Bcat2
ENSMUSG00000030838	1056.864	1616.158	−0.61278	3.81E-05	0.000402	Ush1c
ENSMUSG00000030865	432.78	1349.033	−1.6402	2.60E-52	1.50E-49	Chp2
ENSMUSG00000030866	592.1046	740.494	−0.32264	0.004929	0.025323	Ern2
ENSMUSG00000030868	445.6984	547.6316	−0.29714	0.009509	0.043635	Dctn5
ENSMUSG00000030869	734.6447	1042.103	−0.50438	1.00E-06	1.60E-05	Ndufab1
ENSMUSG00000030882	14.98107	34.70622	−1.2121	0.002135	0.012718	Dnhd1
ENSMUSG00000030909	804.7884	1048.202	−0.38124	0.000211	0.001791	Anks4b
ENSMUSG00000030956	163.4026	278.4807	−0.76915	2.78E-05	0.000303	Fam53b
ENSMUSG00000030979	106.0545	153.3042	−0.53159	0.004611	0.023999	Uros

ENSMUSG00000030987	878.6601	1080.39	−0.29817	0.002573	0.01482	Stim1
ENSMUSG00000031004	3173.408	3721.023	−0.22967	0.007728	0.036876	Mki67
ENSMUSG00000031015	320.468	429.767	−0.42337	0.001173	0.007663	Swap70
ENSMUSG00000031024	330.2229	439.2058	−0.41146	0.002872	0.016218	St5
ENSMUSG00000031060	429.2436	619.9739	−0.53041	6.20E-06	8.11E-05	Rbm10
ENSMUSG00000031065	916.153	1219.572	−0.41272	3.91E-05	0.000411	Cdk16
ENSMUSG00000031090	233.6803	357.3957	−0.61299	3.38E-06	4.76E-05	Nadsyn1
ENSMUSG00000031103	592.4172	848.3474	−0.51804	1.78E-06	2.70E-05	Elf4
ENSMUSG00000031145	74.76149	117.1033	−0.64741	0.001181	0.007704	Prickle3
ENSMUSG00000031153	309.166	410.6148	−0.4094	0.000902	0.00615	Gripap1
ENSMUSG00000031163	147.2593	288.0053	−0.96774	0.000207	0.001764	Glod5
ENSMUSG00000031168	532.4857	718.6735	−0.43259	0.000181	0.001571	Ebp
ENSMUSG00000031173	18.42001	173.3039	−3.234	1.49E-16	1.10E-14	Otc
ENSMUSG00000031209	1615.185	1933.905	−0.25982	0.00973	0.044489	Heph
ENSMUSG00000031214	117.5968	160.5446	−0.44913	0.011112	0.049427	Ophn1
ENSMUSG00000031216	59.16673	96.50952	−0.70589	0.000873	0.005974	Stard8
ENSMUSG00000031217	790.1664	978.6345	−0.30861	0.005139	0.026229	Efnb1
ENSMUSG00000031255	103.9802	162.0861	−0.64045	0.000544	0.004031	Sytl4
ENSMUSG00000031273	19.84207	69.26627	−1.8036	4.65E-09	1.25E-07	Col4a6
ENSMUSG00000031274	80.22929	140.2495	−0.80579	4.59E-05	0.000473	Col4a5
ENSMUSG00000031327	105.6302	171.0647	−0.69552	3.32E-05	0.000356	Chic1
ENSMUSG00000031328	6310.068	8179.283	−0.37432	1.31E-05	0.000156	Flna
ENSMUSG00000031337	576.0058	706.9272	−0.29548	0.005512	0.027767	Mtm1
ENSMUSG00000031340	3.131937	19.52148	−2.6399	3.32E-05	0.000356	Gabre
ENSMUSG00000031352	239.4618	313.0123	−0.38642	0.008543	0.039952	Hccs
ENSMUSG00000031378	259.702	453.947	−0.80567	2.99E-05	0.000323	Abcd1
ENSMUSG00000031386	1072.417	1286.439	−0.26252	0.009303	0.042923	Hcfc1
ENSMUSG00000031391	525.7134	1003.32	−0.93243	7.14E-18	6.15E-16	L1cam
ENSMUSG00000031431	509.33	666.6939	−0.38842	0.000628	0.004552	Tsc22d3
ENSMUSG00000031467	428.4146	556.988	−0.37864	0.001743	0.010739	Agpat5
ENSMUSG00000031478	39.07733	75.68263	−0.95363	0.00027	0.002202	Nek3
ENSMUSG00000031489	10.21126	24.56801	−1.2666	0.004729	0.024478	Adrb3
ENSMUSG00000031497	31.2212	54.68955	−0.80874	0.006813	0.033309	Tnfsf13b
ENSMUSG00000031551	13.18015	168.3848	−3.6753	3.38E-13	1.70E-11	Ido1
ENSMUSG00000031556	552.8642	692.8357	−0.32559	0.004297	0.022614	Tm2d2
ENSMUSG00000031613	516.9567	1467.581	−1.5053	1.05E-46	5.36E-44	Hpgd
ENSMUSG00000031618	585.3174	865.8584	−0.56491	9.04E-08	1.84E-06	Nr3c2
ENSMUSG00000031621	303.523	448.4563	−0.56316	0.000524	0.003895	Isx
ENSMUSG00000031639	299.3844	417.3231	−0.47917	4.92E-05	0.000503	Tlr3
ENSMUSG00000031666	360.5363	450.0748	−0.32002	0.008679	0.040485	Rbl2
ENSMUSG00000031706	204.991	315.4186	−0.62171	8.27E-06	0.000105	Rfx1
ENSMUSG00000031725	86.25822	295.6833	−1.7773	1.08E-08	2.65E-07	Ces1f
ENSMUSG00000031732	369.2605	818.2054	−1.1478	9.23E-24	1.32E-21	Phlpp2
ENSMUSG00000031748	61.27447	122.5364	−0.99985	1.28E-06	2.00E-05	Gnao1
ENSMUSG00000031765	1334.332	2383.986	−0.83726	6.87E-18	5.98E-16	Mt1
ENSMUSG00000031782	650.9438	783.5707	−0.26753	0.00811	0.03839	Coq9
ENSMUSG00000031818	3613.911	4854.937	−0.42589	0.000132	0.001197	Cox4i1

ENSMUSG00000031823	585.7677	801.8559	−0.45301	2.08E-05	0.000234	Zdhhc7
ENSMUSG00000031838	703.1819	954.5831	−0.44097	2.09E-05	0.000235	Ifi30
ENSMUSG00000031853	271.786	433.8139	−0.67461	2.00E-07	3.77E-06	BC021891
ENSMUSG00000031862	526.1372	776.5464	−0.56163	3.37E-07	6.03E-06	Atp13a1
ENSMUSG00000031864	348.9218	445.453	−0.35237	0.003927	0.021048	Ints10
ENSMUSG00000031865	940.8949	1239.919	−0.39814	4.57E-05	0.000472	Dctn1
ENSMUSG00000031879	263.7213	338.502	−0.36015	0.007398	0.035519	Fam96b
ENSMUSG00000031891	1888.456	3314.91	−0.81176	4.86E-19	4.69E-17	Hsd11b2
ENSMUSG00000031897	913.1847	1738.093	−0.92853	2.67E-11	1.03E-09	Psmb10
ENSMUSG00000031913	436.2043	568.8773	−0.38311	0.000639	0.004629	Vps4a
ENSMUSG00000031924	2816.999	3944.368	−0.48564	2.29E-08	5.26E-07	Cyb5b
ENSMUSG00000031930	732.0162	1069.918	−0.54755	1.35E-07	2.64E-06	Wwp2
ENSMUSG00000031938	819.1139	1192.317	−0.54163	6.72E-05	0.000658	4931406C07Rik
ENSMUSG00000031948	1169.114	1578.496	−0.43314	1.06E-05	0.00013	Kars
ENSMUSG00000031981	168.001	250.0179	−0.57356	6.19E-05	0.000614	Capn9
ENSMUSG00000032010	163.9238	321.4636	−0.97163	0.001108	0.007291	Usp2
ENSMUSG00000032012	72.04165	125.6755	−0.8028	0.004725	0.024468	Pvrl1
ENSMUSG00000032015	5.304647	17.08035	−1.687	0.004253	0.022424	Pou2f3
ENSMUSG00000032038	1534.578	3991.512	−1.3791	4.41E-06	6.00E-05	St3gal4
ENSMUSG00000032060	78.38274	132.7501	−0.7601	8.65E-05	0.000821	Cryab
ENSMUSG00000032115	1133.013	1764.094	−0.63876	5.78E-11	2.11E-09	Hyou1
ENSMUSG00000032122	87.33483	365.9023	−2.0668	3.94E-36	1.06E-33	Slc37a2
ENSMUSG00000032193	1497.655	2082.885	−0.47588	3.77E-07	6.67E-06	Ldlr
ENSMUSG00000032194	323.0772	490.3997	−0.60208	7.53E-07	1.25E-05	Kank2
ENSMUSG00000032198	339.2926	432.1782	−0.3491	0.008303	0.039075	Dock6
ENSMUSG00000032235	107.7381	150.2164	−0.47951	0.010324	0.046679	Ice2
ENSMUSG00000032246	778.1666	1122.722	−0.52885	4.99E-07	8.58E-06	Calml4
ENSMUSG00000032264	216.97	289.6121	−0.41663	0.002556	0.014748	Zw10
ENSMUSG00000032265	1286.372	1688.331	−0.39229	4.30E-05	0.000447	Fam46a
ENSMUSG00000032279	1418.885	1889.345	−0.41313	2.33E-05	0.000259	Idh3a
ENSMUSG00000032306	911.6527	1109.362	−0.28317	0.004814	0.0248	Mpi
ENSMUSG00000032312	735.2581	898.9863	−0.29005	0.005364	0.027166	Csk
ENSMUSG00000032314	1322.194	1695.821	−0.35905	0.000138	0.001245	Etfa
ENSMUSG00000032340	576.059	726.55	−0.33485	0.003245	0.017947	Neo1
ENSMUSG00000032342	82.14763	158.1423	−0.94493	7.98E-07	1.31E-05	Mto1
ENSMUSG00000032352	503.6796	637.0443	−0.33889	0.002267	0.013377	Lrrc1
ENSMUSG00000032373	293.4101	394.2591	−0.42623	0.003963	0.021202	Car12
ENSMUSG00000032377	43.86642	69.38664	−0.66154	0.010996	0.049022	Plscr4
ENSMUSG00000032387	63.82591	117.3442	−0.87853	3.77E-05	0.000399	Rbpms2
ENSMUSG00000032479	1218.265	1434.191	−0.23541	0.011039	0.049177	Map4
ENSMUSG00000032504	2201.337	2628.497	−0.25586	0.004793	0.024721	Pdcd6ip
ENSMUSG00000032525	964.8686	1390.472	−0.52717	2.61E-07	4.83E-06	Nktr
ENSMUSG00000032528	1520.482	2080.825	−0.45263	8.41E-07	1.38E-05	Vipr1
ENSMUSG00000032536	1453.514	2221.089	−0.61172	5.99E-11	2.18E-09	Trak1
ENSMUSG00000032548	655.8315	952.3807	−0.53821	5.84E-06	7.72E-05	Slco2a1
ENSMUSG00000032560	806.333	1094.216	−0.44045	1.53E-05	0.000179	Dnajc13
ENSMUSG00000032571	238.6586	304.2132	−0.35013	0.010667	0.047902	Pik3r4

ENSMUSG00000032579	58.03201	87.82454	−0.59778	0.009888	0.045042	Hemk1
ENSMUSG00000032580	926.1559	1128.579	−0.28518	0.006472	0.031802	Rbm5
ENSMUSG00000032583	319.4894	404.4348	−0.34014	0.006739	0.032979	Mon1a
ENSMUSG00000032589	191.2957	249.5309	−0.38341	0.008141	0.038466	Bsn
ENSMUSG00000032590	711.097	931.5133	−0.38953	0.000181	0.001573	Apeh
ENSMUSG00000032594	616.6113	843.1876	−0.45149	3.21E-05	0.000346	Ip6k1
ENSMUSG00000032596	404.7637	753.937	−0.89736	9.75E-15	5.98E-13	Uba7
ENSMUSG00000032601	1359.325	1830.71	−0.42951	0.002669	0.015279	Prkar2a
ENSMUSG00000032602	1528.21	1861.46	−0.28459	0.00204	0.012277	Slc25a20
ENSMUSG00000032606	64.87632	102.7484	−0.66335	0.002833	0.016051	Nicn1
ENSMUSG00000032615	73.22167	105.6657	−0.52917	0.010716	0.048047	Nt5m
ENSMUSG00000032621	382.6821	477.7375	−0.32007	0.010246	0.046399	Srek1
ENSMUSG00000032637	1076.842	1419.205	−0.39828	8.58E-05	0.000815	Atxn2l
ENSMUSG00000032648	34.53557	67.15485	−0.95941	0.000177	0.001544	Pygm
ENSMUSG00000032661	152.0097	290.8409	−0.93607	0.004533	0.023665	Oas3
ENSMUSG00000032690	74.90529	112.653	−0.58875	0.003175	0.017609	Oas2
ENSMUSG00000032727	337.1186	546.9679	−0.6982	3.01E-09	8.38E-08	Mier3
ENSMUSG00000032737	637.3285	970.4526	−0.60662	2.09E-08	4.83E-07	Inppl1
ENSMUSG00000032741	900.8397	1393.277	−0.62914	4.44E-10	1.41E-08	Tpcn1
ENSMUSG00000032743	226.4186	308.6145	−0.44681	0.00116	0.007592	D430042O09Rik
ENSMUSG00000032754	309.1675	472.6344	−0.61234	2.09E-06	3.10E-05	Slc8b1
ENSMUSG00000032777	711.9706	885.4491	−0.31459	0.002402	0.014006	Gtf3c1
ENSMUSG00000032802	1078.696	1492.193	−0.46815	0.001066	0.007083	Srxn1
ENSMUSG00000032816	84.25548	262.3568	−1.6387	4.77E-22	5.96E-20	Igdcc4
ENSMUSG00000032841	25.98839	80.52015	−1.6315	4.62E-09	1.25E-07	Prr5l
ENSMUSG00000032842	155.0241	254.5965	−0.71572	2.73E-06	3.93E-05	Abcc10
ENSMUSG00000032855	505.2575	615.7577	−0.28534	0.009532	0.043706	Pkd1
ENSMUSG00000032899	785.8652	1314.783	−0.74247	3.58E-13	1.79E-11	Styk1
ENSMUSG00000032902	1856.393	2337.416	−0.33241	0.00062	0.004505	Slc16a1
ENSMUSG00000032908	1233.3	2040.244	−0.72622	5.95E-14	3.22E-12	Sgpp2
ENSMUSG00000033009	175.9427	238.5489	−0.43918	0.004505	0.023529	Ogfod1
ENSMUSG00000033021	460.5952	664.2483	−0.52822	2.13E-06	3.16E-05	Gmppa
ENSMUSG00000033068	369.032	523.6557	−0.50487	1.80E-05	0.000207	Entpd6
ENSMUSG00000033083	120.1027	186.9461	−0.63835	0.000188	0.001623	Tbc1d4
ENSMUSG00000033088	453.2035	600.2568	−0.40542	0.000566	0.004178	Triobp
ENSMUSG00000033105	589.173	742.8129	−0.33431	0.001816	0.011124	Lss
ENSMUSG00000033124	571.7721	707.136	−0.30655	0.005002	0.025659	Atg9a
ENSMUSG00000033128	657.8293	835.2655	−0.34452	0.009514	0.043635	Gga1
ENSMUSG00000033161	21016.04	38328.97	−0.86694	8.88E-25	1.32E-22	Atp1a1
ENSMUSG00000033253	327.1822	503.3605	−0.6215	3.99E-07	7.00E-06	Szt2
ENSMUSG00000033287	193.9049	300.5297	−0.63216	1.21E-05	0.000146	Kctd17
ENSMUSG00000033295	2496.715	2946.795	−0.23912	0.007339	0.035295	Ptprf
ENSMUSG00000033327	331.5332	524.2133	−0.661	2.34E-08	5.37E-07	Tnxb
ENSMUSG00000033335	1626.558	1937.028	−0.25202	0.005289	0.026844	Dnm2
ENSMUSG00000033365	218.2817	280.7429	−0.36306	0.008808	0.04102	Ipo13
ENSMUSG00000033416	626.8686	1020.606	−0.70319	2.04E-11	7.98E-10	Gucd1
ENSMUSG00000033444	983.6924	1404.948	−0.51424	1.20E-07	2.37E-06	Specc1l

ENSMUSG00000033502	201.7031	289.262	−0.52014	0.000948	0.006421	Cdc14a
ENSMUSG00000033526	557.3382	926.838	−0.73376	4.25E-12	1.89E-10	Ppip5k1
ENSMUSG00000033542	1239.394	1474.621	−0.25071	0.009829	0.044792	Arhgef5
ENSMUSG00000033576	46.86166	138.4574	−1.563	7.65E-09	1.94E-07	Apol6
ENSMUSG00000033577	2468.671	2986.927	−0.27493	0.002268	0.013379	Myo6
ENSMUSG00000033579	593.9484	1488.778	−1.3257	1.02E-35	2.56E-33	Fa2h
ENSMUSG00000033581	124.2658	381.1594	−1.617	7.66E-28	1.31E-25	Igf2bp2
ENSMUSG00000033590	1576.374	2338.372	−0.56889	1.49E-09	4.40E-08	Myo5c
ENSMUSG00000033594	36.31722	63.59832	−0.80833	0.002828	0.01603	Spata2l
ENSMUSG00000033610	163.3168	303.3635	−0.89337	2.60E-09	7.34E-08	Pank1
ENSMUSG00000033618	140.6952	204.2682	−0.53789	0.000858	0.005896	Map3k13
ENSMUSG00000033671	733.5615	913.5152	−0.31651	0.002515	0.014548	Cep350
ENSMUSG00000033685	3797.403	4719.667	−0.31367	0.000342	0.002694	Ucp2
ENSMUSG00000033697	175.9039	282.6512	−0.68423	8.93E-06	0.000112	Arhgap39
ENSMUSG00000033715	1373.331	2044.923	−0.57437	0.000563	0.004157	Akr1c14
ENSMUSG00000033717	78.12845	296.9619	−1.9264	2.25E-30	4.35E-28	Adra2a
ENSMUSG00000033763	115.203	179.1354	−0.63687	0.000448	0.003409	Mtss1l
ENSMUSG00000033781	137.9808	187.7771	−0.44455	0.00508	0.025975	Asb13
ENSMUSG00000033809	87.31531	126.5297	−0.53517	0.010747	0.048128	Alg3
ENSMUSG00000033819	290.1723	483.9602	−0.73798	1.65E-09	4.84E-08	Ppp1r16a
ENSMUSG00000033826	168.9213	306.7745	−0.86083	4.78E-09	1.28E-07	Dnah8
ENSMUSG00000033845	355.4925	461.6792	−0.37707	0.001531	0.009604	Mrpl15
ENSMUSG00000033862	118.4716	164.426	−0.4729	0.010208	0.046257	Cdk10
ENSMUSG00000033871	171.2518	337.5737	−0.97908	0.000214	0.001811	Ppargc1b
ENSMUSG00000033910	97.50756	173.7704	−0.8336	8.14E-06	0.000104	Gucy1a3
ENSMUSG00000033938	663.7218	885.0812	−0.41523	0.004538	0.023675	Ndufb7
ENSMUSG00000033985	218.6411	380.322	−0.79866	5.53E-09	1.46E-07	Tesk2
ENSMUSG00000034022	502.7915	655.1497	−0.38186	0.000806	0.005604	Cpsf1
ENSMUSG00000034107	182.8784	378.2424	−1.0484	5.81E-14	3.16E-12	Ano7
ENSMUSG00000034108	210.6676	313.96	−0.57561	2.75E-05	0.0003	Ccs
ENSMUSG00000034109	717.7513	883.1492	−0.29917	0.004954	0.025434	Golim4
ENSMUSG00000034152	475.4129	599.0815	−0.33357	0.004014	0.021406	Exoc3
ENSMUSG00000034157	500.5262	887.9279	−0.827	1.73E-14	1.02E-12	Cipc
ENSMUSG00000034160	1488.108	2098.462	−0.49585	4.21E-07	7.36E-06	Ogt
ENSMUSG00000034163	606.3214	812.2676	−0.42187	0.000185	0.001604	Zfc3h1
ENSMUSG00000034165	402.4	522.9745	−0.37811	0.001529	0.009593	Ccnd3
ENSMUSG00000034206	80.46415	118.7263	−0.56122	0.003873	0.020851	Polq
ENSMUSG00000034243	2250.853	3027.286	−0.42755	2.04E-06	3.05E-05	Golgb1
ENSMUSG00000034247	532.5188	779.439	−0.5496	4.21E-07	7.36E-06	Plekhm1
ENSMUSG00000034248	299.067	411.3138	−0.45977	0.000389	0.003015	Slc25a37
ENSMUSG00000034254	784.1564	1162.863	−0.56847	8.03E-08	1.66E-06	Agpat1
ENSMUSG00000034255	770.2327	938.6407	−0.28528	0.005081	0.025975	Arhgap27
ENSMUSG00000034269	948.9525	1286.005	−0.43849	1.19E-05	0.000144	Setd5
ENSMUSG00000034282	383.0541	572.4286	−0.57955	1.11E-06	1.76E-05	Evpl
ENSMUSG00000034308	166.1488	272.2916	−0.71268	2.16E-06	3.18E-05	Sdr42e1
ENSMUSG00000034320	3298.47	5971.987	−0.85641	2.20E-22	2.87E-20	Slc26a2
ENSMUSG00000034342	249.7451	349.4697	−0.48471	0.000414	0.003183	Cbl

ENSMUSG00000034403	496.3245	640.9299	−0.36888	0.000655	0.004712	Pja1
ENSMUSG00000034413	212.2143	443.1044	−1.0621	1.47E-11	5.88E-10	Neurl1b
ENSMUSG00000034422	781.6541	1258.761	−0.6874	1.94E-12	8.97E-11	Parp14
ENSMUSG00000034427	4515.102	7914.389	−0.80972	4.26E-11	1.59E-09	Myo15b
ENSMUSG00000034435	1619.808	1927.996	−0.25128	0.005495	0.027713	Tmem30b
ENSMUSG00000034438	20.35762	75.13176	−1.8839	1.32E-07	2.59E-06	Gbp8
ENSMUSG00000034449	325.7415	788.7302	−1.2758	9.45E-08	1.91E-06	Dhrs11
ENSMUSG00000034471	454.3716	783.8244	−0.78666	2.73E-12	1.23E-10	Caskin2
ENSMUSG00000034472	69.62001	134.9374	−0.95472	2.00E-06	2.99E-05	Rasd2
ENSMUSG00000034485	670.1538	863.4103	−0.36555	0.000798	0.005548	Uaca
ENSMUSG00000034570	262.0905	870.2376	−1.7313	3.03E-26	4.87E-24	Inpp5j
ENSMUSG00000034579	79.62246	168.8723	−1.0847	0.000113	0.001041	Pla2g3
ENSMUSG00000034602	868.8464	1130.19	−0.37939	0.000175	0.001529	Mon2
ENSMUSG00000034613	350.5282	496.6387	−0.50267	4.10E-05	0.000429	Ppm1h
ENSMUSG00000034621	413.5912	547.8158	−0.40549	0.001052	0.007006	Gpatch8
ENSMUSG00000034731	66.6774	121.0966	−0.86089	5.51E-05	0.000556	Dgkh
ENSMUSG00000034771	23.53512	48.22296	−1.0349	0.001982	0.011983	Tle2
ENSMUSG00000034781	1723.726	2948.237	−0.77432	0.000159	0.001401	Gna11
ENSMUSG00000034785	172.5757	343.6847	−0.99386	5.96E-07	1.01E-05	Dio1
ENSMUSG00000034818	24.00509	64.56819	−1.4275	4.11E-06	5.65E-05	Celf5
ENSMUSG00000034853	414.3471	615.0112	−0.56977	5.01E-07	8.59E-06	Acot11
ENSMUSG00000034875	666.9495	848.2457	−0.3469	0.000765	0.005362	Nudt19
ENSMUSG00000034908	816.0837	997.1216	−0.28905	0.006448	0.031731	Sidt2
ENSMUSG00000034918	2473.105	5489.975	−1.1505	3.39E-09	9.32E-08	Cdhr2
ENSMUSG00000034919	657.7184	804.8207	−0.2912	0.008121	0.038407	Ttc22
ENSMUSG00000034926	1942.901	2447.21	−0.33293	0.000378	0.002946	Dhcr24
ENSMUSG00000034936	44.85195	70.54787	−0.65343	0.011036	0.049175	Arl4d
ENSMUSG00000034947	191.55	291.4084	−0.60532	2.63E-05	0.000289	Tmem106a
ENSMUSG00000034949	18.67413	36.07579	−0.94999	0.010772	0.048219	Zfr2
ENSMUSG00000035042	24.56627	67.34652	−1.4549	0.001677	0.010402	Ccl5
ENSMUSG00000035067	18.46542	40.94465	−1.1488	0.002155	0.012814	Xkr6
ENSMUSG00000035069	157.1247	351.5489	−1.1618	1.93E-15	1.27E-13	Oma1
ENSMUSG00000035112	165.3071	234.3386	−0.50345	0.001531	0.009604	Wnk4
ENSMUSG00000035186	43.49461	270.5722	−2.6371	6.66E-06	8.62E-05	Ubd
ENSMUSG00000035200	34.67934	64.88593	−0.90383	0.000428	0.003277	Chrnb4
ENSMUSG00000035202	160.2831	233.262	−0.54133	0.000478	0.003591	Lars2
ENSMUSG00000035203	1676.081	2185.013	−0.38255	0.00065	0.004687	Epn1
ENSMUSG00000035206	228.4228	319.7983	−0.48546	0.001142	0.007493	Sppl2b
ENSMUSG00000035226	8.83464	31.2757	−1.8238	9.48E-05	0.000891	Rims4
ENSMUSG00000035232	243.3898	316.1273	−0.37724	0.006718	0.032892	Pdk3
ENSMUSG00000035239	22.83717	47.77282	−1.0648	0.003074	0.017145	Neu3
ENSMUSG00000035242	2683.77	3183.767	−0.24647	0.00833	0.039171	Oaz1
ENSMUSG00000035258	103.3208	157.3613	−0.60695	0.004666	0.024218	Abi3bp
ENSMUSG00000035284	521.9279	808.1283	−0.63073	6.14E-09	1.59E-07	Vps13c
ENSMUSG00000035325	1423.896	1923.955	−0.43423	2.59E-06	3.75E-05	Sec31a
ENSMUSG00000035382	905.5265	1103.883	−0.28576	0.006265	0.030983	Pcsk7
ENSMUSG00000035413	588.5678	867.3582	−0.55942	1.88E-07	3.56E-06	Tmem98

ENSMUSG00000035429	1132.388	1779.688	−0.65226	0.000169	0.001483	Ptprh
ENSMUSG00000035431	15.30723	78.85986	−2.3651	6.02E-09	1.56E-07	Sstr1
ENSMUSG00000035441	2289.485	2928.197	−0.35499	4.64E-05	0.000477	Myo1d
ENSMUSG00000035473	247.7163	489.482	−0.98257	1.76E-14	1.03E-12	Galm
ENSMUSG00000035506	532.6807	721.3386	−0.43741	5.68E-05	0.00057	Slc12a8
ENSMUSG00000035517	466.9839	631.622	−0.43569	0.000142	0.001271	Tdrd7
ENSMUSG00000035545	1132.597	1580.64	−0.48087	1.00E-06	1.60E-05	Leng8
ENSMUSG00000035561	1069.633	2289.493	−1.0979	2.75E-13	1.39E-11	Aldh1b1
ENSMUSG00000035596	356.2431	504.2774	−0.50136	4.04E-05	0.000422	Mboat7
ENSMUSG00000035686	13.57818	45.40791	−1.7417	3.11E-06	4.41E-05	Thrsp
ENSMUSG00000035722	381.5684	641.9167	−0.75044	0.001043	0.006961	Abca7
ENSMUSG00000035735	116.2009	195.2836	−0.74895	1.07E-05	0.000131	Dagla
ENSMUSG00000035765	243.8196	351.7014	−0.52854	5.69E-05	0.000571	Dym
ENSMUSG00000035783	5559.15	7301.783	−0.39338	2.22E-06	3.26E-05	Acta2
ENSMUSG00000035790	174.4733	224.7392	−0.36525	0.009688	0.044312	Cep19
ENSMUSG00000035845	115.1959	168.4206	−0.54798	0.001763	0.010834	Alg12
ENSMUSG00000035852	3649.034	4480.429	−0.29612	0.000618	0.004494	Misp
ENSMUSG00000035929	381.4617	908.737	−1.2523	1.15E-28	2.04E-26	H2-Q4
ENSMUSG00000035944	372.3536	623.5485	−0.74383	4.68E-10	1.48E-08	Ttc38
ENSMUSG00000035958	193.1614	259.8006	−0.4276	0.004106	0.021766	Tdp2
ENSMUSG00000035967	374.4268	465.2079	−0.31319	0.011223	0.049838	Ddx26b
ENSMUSG00000035969	135.3713	185.1771	−0.45198	0.00963	0.044085	Rusc2
ENSMUSG00000036054	160.3286	229.564	−0.51786	0.001308	0.008384	Sugp2
ENSMUSG00000036120	101.3443	172.7313	−0.76926	4.35E-05	0.000452	Rfxank
ENSMUSG00000036123	765.3326	4120.851	−2.4288	########	########	Slc9a3
ENSMUSG00000036136	238.4696	337.4291	−0.50078	0.000202	0.001726	Fam110c
ENSMUSG00000036138	400.0589	673.9789	−0.75249	0.000143	0.001276	Acaa1a
ENSMUSG00000036181	1115.791	1407.953	−0.33553	0.002403	0.014006	Hist1h1c
ENSMUSG00000036298	205.4018	330.7642	−0.68735	3.70E-06	5.15E-05	Slc2a13
ENSMUSG00000036333	1050.193	1253.013	−0.25475	0.009363	0.043153	Kidins220
ENSMUSG00000036334	71.70148	196.2899	−1.4529	4.06E-14	2.30E-12	Igsf10
ENSMUSG00000036339	207.2482	294.8165	−0.50846	0.000364	0.00285	Tmem260
ENSMUSG00000036352	317.1008	406.8611	−0.35959	0.004929	0.025323	Ubac1
ENSMUSG00000036377	859.337	1180.711	−0.45836	1.25E-05	0.000151	C530008M17Rik
ENSMUSG00000036390	295.6282	380.7646	−0.36512	0.008253	0.038902	Gadd45a
ENSMUSG00000036452	181.9455	300.449	−0.72361	4.76E-07	8.24E-06	Arhgap26
ENSMUSG00000036473	63.36306	140.3582	−1.1474	5.64E-08	1.21E-06	Tbc1d24
ENSMUSG00000036501	238.2732	469.1563	−0.97745	1.91E-08	4.46E-07	Fam13b
ENSMUSG00000036550	2243.54	2901.713	−0.37113	5.12E-05	0.000521	Cnot1
ENSMUSG00000036553	221.4789	288.5215	−0.38151	0.005572	0.028035	Sh3tc1
ENSMUSG00000036594	1455.874	4581.576	−1.654	2.87E-10	9.42E-09	H2-Aa
ENSMUSG00000036606	3603.547	4501.789	−0.32108	0.00018	0.001566	Plxnb2
ENSMUSG00000036646	891.5924	1138.75	−0.353	0.000378	0.002943	Man1b1
ENSMUSG00000036698	955.5254	1150.609	−0.26803	0.009136	0.042271	Ago2
ENSMUSG00000036775	266.7356	401.9608	−0.59164	5.43E-06	7.22E-05	Decr2
ENSMUSG00000036813	594.0622	1127.319	−0.92421	6.11E-18	5.37E-16	Entpd8
ENSMUSG00000036815	160.916	217.8429	−0.43698	0.005745	0.028752	Dpp10

ENSMUSG00000036825	697.1128	866.0328	−0.31303	0.00276	0.015699	Ssx2ip
ENSMUSG00000036833	108.4622	185.762	−0.77626	0.000185	0.001603	Pnpla7
ENSMUSG00000036854	102.3809	137.9371	−0.43006	0.01115	0.049561	Hspb6
ENSMUSG00000036863	86.28433	163.0374	−0.91803	1.04E-06	1.65E-05	Syde2
ENSMUSG00000036904	84.06604	123.3679	−0.55337	0.004181	0.0221	Fzd8
ENSMUSG00000036908	561.594	702.0995	−0.32215	0.003691	0.019979	Unc93b1
ENSMUSG00000036941	154.2016	231.1925	−0.58428	0.000347	0.002726	Elac1
ENSMUSG00000036957	34.84933	71.15585	−1.0299	0.000215	0.001812	Lrfn3
ENSMUSG00000036986	255.4215	406.1006	−0.66896	2.86E-07	5.22E-06	Pml
ENSMUSG00000037012	1643.346	2090.333	−0.3471	0.000197	0.001691	Hk1
ENSMUSG00000037022	99.52941	180.1631	−0.85611	3.28E-07	5.89E-06	Mmaa
ENSMUSG00000037071	861.1492	1322.548	−0.61899	2.79E-09	7.82E-08	Scd1
ENSMUSG00000037098	194.0029	280.4329	−0.53158	0.000231	0.001932	Rab11fip3
ENSMUSG00000037106	313.2767	528.2113	−0.75368	4.57E-10	1.45E-08	Fer1l6
ENSMUSG00000037126	137.2372	242.7792	−0.82297	1.53E-07	2.95E-06	Psd
ENSMUSG00000037139	7.549289	21.82953	−1.5319	0.00243	0.01413	Myom3
ENSMUSG00000037152	555.0424	711.0804	−0.35741	0.004056	0.021555	Ndufc1
ENSMUSG00000037234	346.6192	449.737	−0.37573	0.00435	0.022861	Hook3
ENSMUSG00000037243	140.6233	217.0175	−0.62598	0.000107	0.000995	Zfp692
ENSMUSG00000037266	2106.956	3363.708	−0.67489	2.47E-13	1.26E-11	Rsrp1
ENSMUSG00000037270	864.4893	1091.026	−0.33576	0.001284	0.008257	4932438A13Rik
ENSMUSG00000037321	289.24	1091.47	−1.9159	5.71E-14	3.11E-12	Tap1
ENSMUSG00000037335	12.03852	28.58176	−1.2474	0.006106	0.03027	Hand1
ENSMUSG00000037364	896.6053	1154.907	−0.36523	0.000364	0.002853	Srrt
ENSMUSG00000037366	344.8379	629.392	−0.86804	5.12E-13	2.52E-11	Pafah2
ENSMUSG00000037370	229.4715	302.8865	−0.40046	0.010165	0.046092	Enpp1
ENSMUSG00000037390	10537.86	22602.3	−1.1009	6.16E-20	6.43E-18	Muc3
ENSMUSG00000037408	2151.072	3791.785	−0.81782	1.08E-19	1.11E-17	Cnnm4
ENSMUSG00000037419	2312.35	2907.765	−0.33055	0.000212	0.001797	Endod1
ENSMUSG00000037434	365.8027	643.0054	−0.81377	6.37E-12	2.74E-10	Slc30a1
ENSMUSG00000037455	194.2715	357.1279	−0.87837	1.64E-09	4.81E-08	Slc18b1
ENSMUSG00000037470	1206.874	1637.223	−0.43997	1.26E-05	0.000151	Uggt1
ENSMUSG00000037523	323.6456	516.1167	−0.67328	3.61E-08	7.96E-07	Mavs
ENSMUSG00000037541	141.9158	226.1984	−0.67255	3.81E-05	0.000402	Shank2
ENSMUSG00000037573	938.7105	1158.373	−0.30335	0.00342	0.018723	Tob1
ENSMUSG00000037579	63.38232	110.535	−0.80235	0.000166	0.00146	Kcnh3
ENSMUSG00000037622	468.6402	599.4532	−0.35517	0.001503	0.009459	Wdtc1
ENSMUSG00000037649	102.0422	360.6075	−1.8213	1.42E-10	4.91E-09	H2-DMa
ENSMUSG00000037652	413.8509	567.276	−0.45494	0.000364	0.002851	Phc3
ENSMUSG00000037661	211.1307	378.9337	−0.84381	3.56E-10	1.15E-08	Gpr160
ENSMUSG00000037686	148.4602	247.262	−0.73596	8.56E-06	0.000108	Aspg
ENSMUSG00000037703	711.2939	987.5646	−0.47343	8.14E-06	0.000104	Lzts3
ENSMUSG00000037709	305.629	392.638	−0.36142	0.003933	0.021074	Fam13a
ENSMUSG00000037710	519.1759	734.5131	−0.50056	0.000345	0.002716	Cisd1
ENSMUSG00000037818	81.96529	136.5301	−0.73613	0.000156	0.001377	3110057O12Rik
ENSMUSG00000037826	76.02748	121.1487	−0.67219	0.000713	0.005056	Ppm1k
ENSMUSG00000037887	77.61967	140.0869	−0.85183	2.01E-05	0.000227	Dusp8

ENSMUSG00000037904	144.2253	208.8776	−0.53433	0.000798	0.005548	Ankrd9
ENSMUSG00000037916	1355.955	1833.801	−0.43553	5.29E-05	0.000536	Ndufv1
ENSMUSG00000037921	335.4548	425.6224	−0.34346	0.007906	0.037556	Ddx60
ENSMUSG00000037949	360.6535	527.426	−0.54836	3.39E-06	4.77E-05	Ano10
ENSMUSG00000037965	1069.179	1385.831	−0.37425	0.001377	0.008771	Zc3h7a
ENSMUSG00000037991	20.24008	45.84568	−1.1796	0.000601	0.00439	Rmi2
ENSMUSG00000038002	282.7878	392.1647	−0.47174	0.000671	0.004798	Cramp1l
ENSMUSG00000038014	3068.371	4206.285	−0.45507	3.73E-07	6.60E-06	Fam120a
ENSMUSG00000038020	364.0609	624.5576	−0.77865	8.00E-11	2.86E-09	Rapgefl1
ENSMUSG00000038023	898.57	1730.642	−0.9456	0.005007	0.025677	Atp6v0a2
ENSMUSG00000038056	811.1508	1037.69	−0.35533	0.000852	0.005855	Kmt2c
ENSMUSG00000038058	138.3262	204.9327	−0.56708	0.000343	0.002701	Nod1
ENSMUSG00000038084	898.7896	1169.894	−0.38032	0.000242	0.002015	Opa1
ENSMUSG00000038128	41.90939	81.88908	−0.9664	0.000441	0.003361	Camk4
ENSMUSG00000038167	131.9135	259.1877	−0.97441	1.29E-05	0.000154	Plekhg6
ENSMUSG00000038168	42.86163	84.18437	−0.97387	0.000105	0.000976	P3h2
ENSMUSG00000038172	603.9771	786.7189	−0.38135	0.000257	0.002104	Ttc39b
ENSMUSG00000038179	39.85416	76.25386	−0.93608	0.001755	0.010795	Slamf7
ENSMUSG00000038188	40.34344	69.41873	−0.78299	0.005028	0.025747	Scarf1
ENSMUSG00000038195	54.76331	102.5714	−0.90535	0.000112	0.001031	Rilp
ENSMUSG00000038213	357.8298	562.0532	−0.65143	1.35E-05	0.00016	Tapbpl
ENSMUSG00000038225	77.60588	121.1354	−0.64238	0.00064	0.004631	Primpol
ENSMUSG00000038239	5.98333	18.86864	−1.657	0.002718	0.015499	Hrc
ENSMUSG00000038240	126.2035	262.9328	−1.0589	2.76E-11	1.06E-09	Pdss2
ENSMUSG00000038260	889.5606	1523.954	−0.77665	1.28E-05	0.000153	Trpm4
ENSMUSG00000038267	817.6737	1108.259	−0.4387	1.11E-05	0.000135	Slc22a23
ENSMUSG00000038286	94.48597	131.413	−0.47594	0.00916	0.042349	Bphl
ENSMUSG00000038291	377.2852	486.3098	−0.36622	0.001502	0.009458	Snx25
ENSMUSG00000038302	107.1051	221.6198	−1.0491	3.58E-10	1.16E-08	Lace1
ENSMUSG00000038312	436.4339	549.5783	−0.33256	0.005089	0.026008	Edem2
ENSMUSG00000038331	778.3361	1251.551	−0.68525	1.30E-11	5.28E-10	Satb2
ENSMUSG00000038342	697.5385	1089.727	−0.64362	1.03E-09	3.07E-08	Mlxip
ENSMUSG00000038365	431.6312	757.498	−0.81144	3.86E-13	1.91E-11	Fbxo25
ENSMUSG00000038366	3269.34	4147.024	−0.34308	8.29E-05	0.000793	Lasp1
ENSMUSG00000038375	1775.403	2155.656	−0.27998	0.0027	0.015421	Trp53inp2
ENSMUSG00000038412	1436.272	1768.992	−0.3006	0.000698	0.004964	Higd1a
ENSMUSG00000038422	147.1487	249.6253	−0.76249	1.38E-06	2.14E-05	Hdhd3
ENSMUSG00000038462	2164.94	2749.607	−0.3449	0.000134	0.001205	Uqcrfs1
ENSMUSG00000038497	608.5783	936.5695	−0.62194	5.05E-09	1.35E-07	Tmco3
ENSMUSG00000038507	1102.491	1593.141	−0.53111	4.33E-08	9.46E-07	Parp12
ENSMUSG00000038515	502.6869	617.6054	−0.29703	0.005421	0.027407	Grtp1
ENSMUSG00000038518	457.2806	654.2611	−0.51679	6.38E-06	8.30E-05	Jarid2
ENSMUSG00000038534	337.6093	535.5889	−0.66577	2.87E-07	5.23E-06	Osbpl7
ENSMUSG00000038583	28.46809	86.378	−1.6013	6.13E-09	1.59E-07	Pln
ENSMUSG00000038587	164.9021	226.2613	−0.45638	0.002629	0.015087	Akap12
ENSMUSG00000038668	301.7993	372.8533	−0.30502	0.00975	0.044524	Lpar1
ENSMUSG00000038671	207.9793	271.4733	−0.38437	0.008171	0.03858	Arfrp1

ENSMUSG00000038700	21.40803	56.16883	−1.3916	0.006198	0.030685	Hoxb5
ENSMUSG00000038708	1901.783	2478.478	−0.3821	2.80E-05	0.000305	Golga4
ENSMUSG00000038732	1436.893	1729.533	−0.26743	0.003465	0.018939	Mboat1
ENSMUSG00000038745	696.2204	2547.128	−1.8713	2.56E-79	3.05E-76	Nlrp6
ENSMUSG00000038756	160.3218	252.7998	−0.65702	2.16E-05	0.000242	Ttll6
ENSMUSG00000038807	713.7388	953.1681	−0.41733	4.53E-05	0.000468	Rap1gap2
ENSMUSG00000038838	182.0363	251.5274	−0.46649	0.001777	0.01091	Vars2
ENSMUSG00000038859	1224.712	1589.686	−0.3763	8.89E-05	0.000841	Baiap2l1
ENSMUSG00000038909	521.087	661.937	−0.34517	0.003139	0.017423	Kat7
ENSMUSG00000038914	657.0899	808.3073	−0.29881	0.00456	0.023769	Dido1
ENSMUSG00000038949	284.7506	454.5895	−0.67487	1.08E-07	2.14E-06	Cnst
ENSMUSG00000038967	196.6072	428.3447	−1.1235	1.11E-10	3.90E-09	Pdk2
ENSMUSG00000039110	15.63334	33.97072	−1.1197	0.002444	0.014182	Mycbpap
ENSMUSG00000039115	184.1829	262.1393	−0.50919	0.000558	0.004122	Itga9
ENSMUSG00000039117	294.3618	403.8104	−0.45609	0.000632	0.004576	Taf4a
ENSMUSG00000039157	963.7081	1326.624	−0.46109	6.25E-05	0.000619	Fam102a
ENSMUSG00000039176	387.0801	479.6668	−0.3094	0.010031	0.045631	Polg
ENSMUSG00000039197	616.9099	898.6352	−0.54268	2.71E-07	4.98E-06	Adk
ENSMUSG00000039205	288.2942	371.1353	−0.3644	0.006988	0.034008	Ciz1
ENSMUSG00000039206	175.0085	314.0082	−0.84338	2.36E-07	4.40E-06	Daglb
ENSMUSG00000039210	223.4303	374.5709	−0.74541	6.08E-08	1.29E-06	Gpatch2
ENSMUSG00000039218	3488.957	4828.738	−0.46885	1.32E-07	2.59E-06	Srrm2
ENSMUSG00000039254	155.7936	235.3088	−0.59492	0.000152	0.001348	Pomt1
ENSMUSG00000039278	40.75382	72.36724	−0.8284	0.000488	0.003659	Pcsk1n
ENSMUSG00000039285	935.1789	1144.805	−0.29179	0.003246	0.017949	Azi2
ENSMUSG00000039304	211.9335	401.7124	−0.92255	2.12E-11	8.26E-10	Tnfsf10
ENSMUSG00000039364	1019.464	3106.657	−1.6076	3.01E-38	9.26E-36	Sectm1b
ENSMUSG00000039367	1371.895	1774.692	−0.3714	9.82E-05	0.000919	Sec24c
ENSMUSG00000039414	255.4073	379.3252	−0.57064	7.86E-06	0.000101	Heatr5b
ENSMUSG00000039428	333.3987	427.9359	−0.36015	0.002744	0.015621	Tmem135
ENSMUSG00000039501	477.272	689.6583	−0.53107	1.59E-06	2.44E-05	Znfx1
ENSMUSG00000039611	260.1196	471.1907	−0.85714	1.22E-11	4.96E-10	Tmem246
ENSMUSG00000039678	849.273	1018.155	−0.26166	0.008557	0.040007	Tbc1d13
ENSMUSG00000039699	139.8729	237.3446	−0.76287	8.65E-07	1.41E-05	Batf2
ENSMUSG00000039701	558.7634	797.5133	−0.51327	0.000192	0.001655	Usp53
ENSMUSG00000039741	172.2828	259.8472	−0.59288	0.000663	0.004764	Bahcc1
ENSMUSG00000039768	636.707	786.2459	−0.30435	0.005328	0.027008	Dnajc11
ENSMUSG00000039774	910.5565	1104.581	−0.27868	0.00882	0.041043	Galnt12
ENSMUSG00000039840	229.3225	303.3198	−0.40346	0.004646	0.024132	Epg5
ENSMUSG00000039865	479.9354	604.9663	−0.33401	0.004756	0.024579	Slc44a3
ENSMUSG00000039878	187.0875	463.0789	−1.3075	2.54E-05	0.00028	Slc39a5
ENSMUSG00000039899	451.7857	621.0879	−0.45916	0.000589	0.004319	Fgl2
ENSMUSG00000039910	552.1263	671.8776	−0.2832	0.00849	0.039828	Cited2
ENSMUSG00000039953	1373.422	1886.073	−0.45761	1.34E-06	2.09E-05	Clstn1
ENSMUSG00000039958	107.0068	192.3288	−0.84587	4.38E-07	7.64E-06	Mettl20
ENSMUSG00000040033	371.8907	681.8584	−0.87459	5.76E-07	9.77E-06	Stat2
ENSMUSG00000040048	823.5583	1274.891	−0.63043	1.71E-10	5.82E-09	Ndufb10

ENSMUSG00000040054	1130.709	1368.823	−0.27571	0.004439	0.023238	Baz2a
ENSMUSG00000040061	35.38421	59.52737	−0.75045	0.010853	0.048521	Plcb2
ENSMUSG00000040147	130.9928	317.9699	−1.2794	2.77E-05	0.000303	Maob
ENSMUSG00000040189	33.46585	56.97288	−0.76759	0.006578	0.032285	Ccdc114
ENSMUSG00000040234	405.2899	519.9332	−0.35937	0.001265	0.008159	Tm7sf3
ENSMUSG00000040249	1231.091	2112.487	−0.779	8.38E-09	2.10E-07	Lrp1
ENSMUSG00000040253	220.5913	363.1449	−0.71917	9.30E-08	1.88E-06	Gbp7
ENSMUSG00000040356	662.4873	865.5421	−0.38571	0.00033	0.002617	Skiv2l
ENSMUSG00000040412	756.802	1378.953	−0.86558	7.88E-18	6.76E-16	5330417C22Rik
ENSMUSG00000040441	5.304656	18.97602	−1.8388	0.001458	0.009224	Slc26a10
ENSMUSG00000040471	280.4841	440.3376	−0.65069	5.81E-07	9.85E-06	Ggt6
ENSMUSG00000040479	994.9747	1256.492	−0.33667	0.000739	0.005207	Dgkz
ENSMUSG00000040488	808.6958	1007.571	−0.31721	0.003008	0.016876	Ltbp4
ENSMUSG00000040537	28.2787	50.37916	−0.83311	0.00573	0.028691	Adam22
ENSMUSG00000040543	121.225	170.0697	−0.48844	0.002938	0.016523	Pitpnm3
ENSMUSG00000040548	330.6405	458.1198	−0.47046	0.000292	0.002358	Tex2
ENSMUSG00000040562	414.3072	631.351	−0.60774	1.68E-07	3.21E-06	Gstm2
ENSMUSG00000040584	1058.084	1878.822	−0.82837	0.001193	0.007764	Abcb1a
ENSMUSG00000040701	309.5711	516.6671	−0.73896	1.76E-09	5.10E-08	Ap1g2
ENSMUSG00000040703	370.4022	768.6375	−1.0532	5.56E-09	1.46E-07	Cyp2s1
ENSMUSG00000040712	547.4022	979.5193	−0.83947	6.70E-15	4.14E-13	Camta2
ENSMUSG00000040721	79.60299	136.5231	−0.77825	7.48E-05	0.000722	Zfhx2
ENSMUSG00000040722	442.8675	551.8771	−0.31747	0.005402	0.027317	Scamp5
ENSMUSG00000040740	128.3501	265.0782	−1.0463	9.59E-09	2.38E-07	Slc25a34
ENSMUSG00000040746	292.2609	466.5488	−0.67477	9.96E-08	2.00E-06	Rnf167
ENSMUSG00000040761	528.2065	677.2137	−0.35851	0.001838	0.011245	Spen
ENSMUSG00000040841	77.56713	128.2863	−0.72585	0.000378	0.002943	Six5
ENSMUSG00000040859	691.196	848.164	−0.29525	0.004071	0.021605	Bsdc1
ENSMUSG00000040896	66.76858	107.3457	−0.68502	0.000959	0.006481	Kcnd3
ENSMUSG00000040950	24.53993	51.67855	−1.0744	0.00095	0.006434	Mgl2
ENSMUSG00000040957	180.8232	270.1288	−0.57907	0.000116	0.001062	Cables1
ENSMUSG00000040969	348.3994	545.5928	−0.64708	4.50E-05	0.000465	Arhgef38
ENSMUSG00000041040	417.8053	555.8112	−0.41176	0.000477	0.003587	Fam117b
ENSMUSG00000041064	77.47601	120.5641	−0.63798	0.002139	0.012739	Pif1
ENSMUSG00000041073	32.65036	62.09594	−0.9274	0.001002	0.006729	Nacad
ENSMUSG00000041115	198.3485	363.5459	−0.8741	1.82E-10	6.16E-09	Iqsec2
ENSMUSG00000041143	342.1302	568.2905	−0.73208	7.63E-10	2.32E-08	Tmco4
ENSMUSG00000041193	288.3191	803.57	−1.4788	2.84E-36	7.93E-34	Pla2g5
ENSMUSG00000041220	559.2898	860.3233	−0.62128	8.20E-09	2.06E-07	Elovl6
ENSMUSG00000041229	254.7041	348.4722	−0.45222	0.001726	0.010664	Phf8
ENSMUSG00000041237	542.1187	777.0102	−0.51932	0.000251	0.002075	Pklr
ENSMUSG00000041241	477.2015	633.9562	−0.40978	0.000339	0.002682	Mul1
ENSMUSG00000041301	757.265	1544.184	−1.028	6.56E-21	7.27E-19	Cftr
ENSMUSG00000041426	201.0299	274.0586	−0.44707	0.002155	0.012814	Hibch
ENSMUSG00000041459	1894.762	2428.16	−0.35785	0.005369	0.027177	Tardbp
ENSMUSG00000041515	730.2086	1169.717	−0.67978	3.40E-09	9.33E-08	Irf8
ENSMUSG00000041528	281.9518	369.2121	−0.389	0.004046	0.021517	Rnf123

ENSMUSG00000041577	354.0633	559.9241	−0.66122	3.21E-08	7.13E-07	Prelp
ENSMUSG00000041632	223.4428	319.9109	−0.51776	0.000173	0.001515	Mrps27
ENSMUSG00000041642	322.7126	554.5237	−0.781	1.90E-10	6.40E-09	Kif21b
ENSMUSG00000041649	5.187152	15.27967	−1.5586	0.00974	0.044512	Klf8
ENSMUSG00000041650	275.4146	503.0329	−0.86905	2.15E-10	7.17E-09	Pcca
ENSMUSG00000041697	3930.376	5430.636	−0.46645	0.000192	0.001658	Cox6a1
ENSMUSG00000041720	538.7569	725.1935	−0.42873	0.000161	0.001421	Pi4ka
ENSMUSG00000041731	534.638	657.8309	−0.29915	0.003893	0.020917	Pgm5
ENSMUSG00000041733	262.7935	429.7122	−0.70944	1.25E-08	3.03E-07	Coq5
ENSMUSG00000041740	1940.539	2405.375	−0.3098	0.000595	0.004352	Rnf10
ENSMUSG00000041757	2249.413	2991.83	−0.41148	7.48E-06	9.60E-05	Plekha6
ENSMUSG00000041779	133.0742	192.3673	−0.53163	0.00144	0.009118	Tram2
ENSMUSG00000041828	40.96942	94.98772	−1.2132	9.37E-07	1.51E-05	Abca8a
ENSMUSG00000041926	987.4646	1276.095	−0.36993	0.000335	0.002652	Rnpep
ENSMUSG00000041945	189.742	272.691	−0.52323	0.000199	0.001703	Mfsd9
ENSMUSG00000042010	107.9012	199.1531	−0.88417	0.000109	0.00101	Acacb
ENSMUSG00000042041	270.5012	411.5932	−0.60558	0.000653	0.004699	2010003K11Rik
ENSMUSG00000042055	521.7065	654.7992	−0.32781	0.003317	0.018258	Wdr11
ENSMUSG00000042073	326.1444	444.54	−0.4468	0.00046	0.003482	Abhd14b
ENSMUSG00000042082	251.474	340.0679	−0.43541	0.001249	0.008068	Arsb
ENSMUSG00000042129	221.5452	343.6809	−0.63347	9.52E-06	0.000118	Rassf4
ENSMUSG00000042148	256.8769	432.2928	−0.75093	8.95E-09	2.24E-07	Cox10
ENSMUSG00000042195	217.2386	309.0904	−0.50875	0.000271	0.002205	Slc35f2
ENSMUSG00000042203	245.843	327.2743	−0.41276	0.002566	0.014787	Tbc1d22b
ENSMUSG00000042210	58.52124	98.46268	−0.75062	0.000924	0.00628	Abhd14a
ENSMUSG00000042251	87.15266	163.8186	−0.91048	0.000557	0.00412	Pm20d1
ENSMUSG00000042333	66.65801	138.1585	−1.0515	3.98E-07	7.00E-06	Tnfrsf14
ENSMUSG00000042349	249.484	443.7491	−0.8308	1.33E-10	4.63E-09	Ikbke
ENSMUSG00000042359	81.49527	141.2469	−0.79343	3.69E-05	0.000391	Osbpl6
ENSMUSG00000042363	451.6291	593.7672	−0.39476	0.000572	0.004217	Lgalsl
ENSMUSG00000042408	96.33251	149.7101	−0.63608	0.000528	0.003924	Zmym6
ENSMUSG00000042428	966.279	1153.084	−0.25499	0.010922	0.048754	Mgat3
ENSMUSG00000042496	70.09675	106.4768	−0.60312	0.008492	0.039828	Prdm10
ENSMUSG00000042569	286.055	470.0816	−0.71662	4.66E-09	1.25E-07	Dhrs7b
ENSMUSG00000042605	298.2054	454.147	−0.60685	2.68E-06	3.87E-05	Atxn2
ENSMUSG00000042606	185.5138	258.9062	−0.4809	0.001069	0.007101	Hirip3
ENSMUSG00000042625	546.8349	789.2627	−0.5294	2.75E-06	3.96E-05	Safb2
ENSMUSG00000042638	2219.121	2726.219	−0.29691	0.000481	0.003609	Gucy2c
ENSMUSG00000042642	247.4742	342.5131	−0.46888	0.000778	0.005442	Flad1
ENSMUSG00000042659	644.3485	847.0932	−0.39468	0.000478	0.003591	Arrdc4
ENSMUSG00000042675	247.0373	317.3905	−0.36153	0.007171	0.034671	Ypel3
ENSMUSG00000042700	862.0038	1094.816	−0.34492	0.000629	0.004561	Sipa1l1
ENSMUSG00000042726	520.2261	735.9894	−0.50055	5.46E-06	7.25E-05	Trafd1
ENSMUSG00000042744	437.3348	659.0815	−0.59172	3.23E-07	5.80E-06	Gm15800
ENSMUSG00000042766	158.7363	210.392	−0.40645	0.011076	0.049316	Trim46
ENSMUSG00000042797	54.22853	134.5013	−1.3105	9.70E-08	1.95E-06	Aqp11
ENSMUSG00000042826	25.63598	43.94403	−0.7775	0.010807	0.04834	Fgf11

ENSMUSG00000042985	12.80831	49.94135	−1.9632	1.08E-07	2.14E-06	Upk3b
ENSMUSG00000043065	80.04665	137.2896	−0.77831	9.66E-05	0.000905	Spice1
ENSMUSG00000043079	467.1013	768.6029	−0.7185	6.34E-10	1.96E-08	Synpo
ENSMUSG00000043085	130.4912	279.3188	−1.098	1.13E-05	0.000137	Tmem82
ENSMUSG00000043110	5.141589	23.16699	−2.1718	6.91E-05	0.000673	Lrrn4
ENSMUSG00000043257	136.6235	186.35	−0.44781	0.005577	0.028044	Pigv
ENSMUSG00000043279	522.8954	738.8346	−0.49873	7.12E-06	9.17E-05	Trim56
ENSMUSG00000043286	17.87798	36.67779	−1.0367	0.005163	0.026315	Pnpla1
ENSMUSG00000043384	144.59	194.4782	−0.42764	0.006365	0.031396	Gprasp1
ENSMUSG00000043461	447.1136	799.7137	−0.83884	0.000211	0.001792	Sptssb
ENSMUSG00000043496	43.18781	74.94119	−0.79513	0.001221	0.007911	Tril
ENSMUSG00000043705	378.2249	532.047	−0.49231	3.49E-05	0.000373	Capn13
ENSMUSG00000043811	7.594861	23.82687	−1.6495	0.001673	0.010388	Rtn4r
ENSMUSG00000043987	224.7876	299.4472	−0.41374	0.002799	0.015891	Cep164
ENSMUSG00000043991	849.1833	1182.089	−0.47719	6.41E-06	8.32E-05	Pura
ENSMUSG00000044017	52.79936	88.20407	−0.74032	0.002915	0.016423	Adgrd1
ENSMUSG00000044134	339.1682	486.5922	−0.52071	7.37E-05	0.000712	Fam109a
ENSMUSG00000044156	810.8081	1062.306	−0.38977	9.97E-05	0.000931	Hepacam2
ENSMUSG00000044340	224.7353	312.9491	−0.4777	0.000932	0.00633	Phlpp1
ENSMUSG00000044345	302.4587	387.2895	−0.35667	0.003542	0.019291	Marveld1
ENSMUSG00000044349	54.76319	97.46061	−0.83161	0.000649	0.004678	Snhg11
ENSMUSG00000044433	398.4858	581.775	−0.54593	3.79E-06	5.26E-05	Camsap3
ENSMUSG00000044465	523.2072	668.6541	−0.35388	0.001291	0.00829	Fam160a2
ENSMUSG00000044519	243.9315	329.3107	−0.43297	0.00172	0.010635	Zfp488
ENSMUSG00000044783	1441.416	1785.268	−0.30865	0.001519	0.009543	Hjurp
ENSMUSG00000044792	311.7299	389.5377	−0.32147	0.010899	0.048683	Isca1
ENSMUSG00000044827	555.583	670.4917	−0.27122	0.009094	0.042122	Tlr1
ENSMUSG00000044860	174.7107	538.2672	−1.6234	1.90E-06	2.87E-05	Gm1123
ENSMUSG00000044881	109.4673	160.3299	−0.55054	0.002034	0.012252	Coa4
ENSMUSG00000044937	14.58301	33.8945	−1.2168	0.002101	0.012577	Ttc41
ENSMUSG00000044952	37.90949	76.4629	−1.0122	9.86E-05	0.000922	Kctd21
ENSMUSG00000044986	951.4591	1532.917	−0.68807	1.71E-12	7.99E-11	Tst
ENSMUSG00000045005	680.6995	897.0001	−0.39809	0.008421	0.03956	Fzd5
ENSMUSG00000045019	122.8561	388.3604	−1.6604	8.86E-30	1.66E-27	Acer1
ENSMUSG00000045038	164.8759	299.6356	−0.86183	3.73E-09	1.02E-07	Prkce
ENSMUSG00000045302	805.838	1074.532	−0.41515	5.07E-05	0.000517	Preb
ENSMUSG00000045316	268.6335	555.4798	−1.0481	7.02E-07	1.17E-05	Fahd1
ENSMUSG00000045348	16.85389	41.91561	−1.3144	0.005316	0.026955	Nyap1
ENSMUSG00000045409	219.7828	289.9416	−0.39968	0.006191	0.030658	Trim39
ENSMUSG00000045659	624.6897	857.4907	−0.45698	3.16E-05	0.000341	Plekha7
ENSMUSG00000045664	57.84274	127.1417	−1.1362	0.003366	0.018478	Cdc42ep2
ENSMUSG00000045672	99.32765	190.1602	−0.93695	1.78E-07	3.40E-06	Col27a1
ENSMUSG00000045680	100.3325	189.5378	−0.9177	4.64E-07	8.06E-06	Tcf21
ENSMUSG00000045775	297.7362	656.7329	−1.1413	4.40E-08	9.58E-07	Slc16a5
ENSMUSG00000045795	168.034	248.0283	−0.56175	0.000128	0.001166	Whamm
ENSMUSG00000045827	97.12867	140.0853	−0.52834	0.006276	0.031019	Serpinb9
ENSMUSG00000045868	33.13971	80.31683	−1.2771	2.16E-06	3.19E-05	Gvin1

ENSMUSG00000045871	29.12039	61.28993	−1.0736	0.000337	0.002667	Slitrk6
ENSMUSG00000045903	5.304665	16.12918	−1.6043	0.009166	0.04237	Npas4
ENSMUSG00000045930	156.5569	208.1542	−0.41097	0.007074	0.034296	Clec14a
ENSMUSG00000045973	677.4038	827.6167	−0.28895	0.007518	0.036031	Slc25a51
ENSMUSG00000045980	100.4955	139.7434	−0.47565	0.010807	0.04834	Tmem104
ENSMUSG00000046179	189.9383	269.7154	−0.50591	0.001077	0.007139	E2f8
ENSMUSG00000046295	57.12551	119.0043	−1.0588	4.90E-06	6.59E-05	Ankle1
ENSMUSG00000046324	1196.838	1501.986	−0.32764	0.000504	0.003764	Ermp1
ENSMUSG00000046329	165.5156	229.1342	−0.46923	0.002346	0.013747	Slc25a23
ENSMUSG00000046352	80.27476	126.3909	−0.65487	0.000784	0.005468	Gjb2
ENSMUSG00000046404	112.2009	154.7192	−0.46357	0.007991	0.037907	Yod1
ENSMUSG00000046417	234.641	344.2278	−0.55291	0.000115	0.001057	Lrrc75a
ENSMUSG00000046442	23.20891	47.25336	−1.0257	0.001845	0.011282	Ppm1e
ENSMUSG00000046460	7.757937	28.93025	−1.8988	3.32E-05	0.000356	Sh2d7
ENSMUSG00000046598	1153.049	1662.656	−0.52804	3.20E-08	7.12E-07	Bdh1
ENSMUSG00000046709	9.487028	21.48074	−1.179	0.011013	0.049085	Mapk10
ENSMUSG00000046743	108.7885	183.7824	−0.75647	7.92E-06	0.000101	Fat4
ENSMUSG00000046861	760.2424	1112.366	−0.5491	6.92E-08	1.45E-06	Hectd3
ENSMUSG00000046879	436.6954	881.7086	−1.0137	1.20E-19	1.22E-17	Irgm1
ENSMUSG00000047030	379.4715	555.4914	−0.54977	1.80E-05	0.000207	Spata2
ENSMUSG00000047040	1307.441	2041.101	−0.6426	8.61E-12	3.64E-10	Prr15l
ENSMUSG00000047098	214.8043	385.9341	−0.84533	3.22E-10	1.05E-08	Rnf31
ENSMUSG00000047123	292.0193	424.9758	−0.54132	2.12E-05	0.000238	Ticam1
ENSMUSG00000047153	509.8243	873.1735	−0.77627	6.92E-13	3.36E-11	Khnyn
ENSMUSG00000047180	1428.778	2018.474	−0.49848	7.88E-06	0.000101	Neurl3
ENSMUSG00000047205	93.22683	149.1455	−0.6779	0.000349	0.002742	Dusp18
ENSMUSG00000047248	256.7327	349.7121	−0.4459	0.000736	0.005195	C2cd3
ENSMUSG00000047284	328.6955	479.1522	−0.54373	1.29E-05	0.000155	Neurl4
ENSMUSG00000047371	184.1444	254.9956	−0.46963	0.00187	0.011391	Zfp768
ENSMUSG00000047409	399.4053	585.516	−0.55186	1.51E-06	2.33E-05	Ctdspl
ENSMUSG00000047417	344.4272	457.0009	−0.408	0.000943	0.006392	Rexo1
ENSMUSG00000047454	163.4272	222.2784	−0.44372	0.005442	0.027507	Gphn
ENSMUSG00000047604	138.6857	270.416	−0.96336	2.80E-10	9.25E-09	Frat2
ENSMUSG00000047656	35.82791	66.62276	−0.89493	0.001001	0.006729	Trpt1
ENSMUSG00000047730	15834.74	20479.71	−0.3711	6.65E-06	8.62E-05	Fcgbp
ENSMUSG00000047793	61.69888	150.0142	−1.2818	3.92E-10	1.26E-08	Sned1
ENSMUSG00000047804	193.5402	264.4612	−0.45042	0.002819	0.015982	Akap10
ENSMUSG00000047821	706.2229	887.2307	−0.32919	0.001502	0.009458	Trim16
ENSMUSG00000047842	23.30014	71.69469	−1.6215	2.49E-08	5.67E-07	Diras2
ENSMUSG00000047866	630.8794	786.1163	−0.31738	0.002332	0.013684	Lonp2
ENSMUSG00000047909	292.0066	381.836	−0.38695	0.004544	0.0237	Ankrd16
ENSMUSG00000048027	265.1168	380.1814	−0.52006	6.74E-05	0.00066	Rgmb
ENSMUSG00000048096	290.3483	481.7612	−0.73053	4.56E-08	9.91E-07	Lmod1
ENSMUSG00000048154	1094.769	1570.676	−0.52076	0.010519	0.047348	Kmt2d
ENSMUSG00000048217	12.67162	65.93312	−2.3794	0.003052	0.017058	Nags
ENSMUSG00000048271	590.9751	746.7713	−0.33757	0.002586	0.014873	Rbm33
ENSMUSG00000048307	232.6949	296.0162	−0.34724	0.008915	0.041389	Ankrd46

ENSMUSG00000048337	38.9598	84.4947	−1.1169	1.68E-05	0.000195	Npy4r
ENSMUSG00000048371	162.9973	250.7146	−0.6212	0.000121	0.001107	Pdp2
ENSMUSG00000048440	267.0759	555.8802	−1.0575	1.11E-05	0.000135	Cyp4f16
ENSMUSG00000048537	543.3617	757.8129	−0.47993	9.64E-06	0.00012	Phldb1
ENSMUSG00000048572	248.3353	351.708	−0.50209	0.000136	0.001223	Tmem252
ENSMUSG00000048578	3961.756	5458.625	−0.4624	1.70E-07	3.25E-06	Mlec
ENSMUSG00000048701	619.8993	899.9607	−0.53783	7.40E-07	1.23E-05	Ccdc6
ENSMUSG00000048756	819.6635	1144.635	−0.48178	1.55E-06	2.39E-05	Foxo3
ENSMUSG00000048832	398.961	508.7101	−0.3506	0.00247	0.014315	Vps37c
ENSMUSG00000048833	976.1487	1464.283	−0.58502	1.67E-09	4.90E-08	Slc39a9
ENSMUSG00000048852	3.412508	34.92227	−3.3552	6.38E-08	1.35E-06	Gm12185
ENSMUSG00000048905	555.5008	960.4348	−0.7899	4.28E-13	2.11E-11	4930539E08Rik
ENSMUSG00000048930	182.0173	242.9611	−0.41665	0.007685	0.0367	Tada3
ENSMUSG00000049091	495.2026	640.7818	−0.37181	0.000866	0.005939	Sephs2
ENSMUSG00000049241	10.23758	29.87472	−1.5451	0.000738	0.005206	Hcar1
ENSMUSG00000049265	27.2545	69.85014	−1.3578	3.09E-06	4.38E-05	Kcnk3
ENSMUSG00000049327	1211.937	1615.259	−0.41445	4.02E-05	0.000421	Setd8
ENSMUSG00000049350	13115.08	18684.95	−0.51065	1.87E-09	5.39E-08	Zg16
ENSMUSG00000049422	806.3323	1254.706	−0.6379	7.17E-11	2.58E-09	Chchd10
ENSMUSG00000049493	2706.374	4180.372	−0.62727	0.002258	0.01333	Pls1
ENSMUSG00000049502	678.7263	997.4796	−0.55546	7.65E-08	1.59E-06	Dtx3l
ENSMUSG00000049580	120.227	188.8374	−0.65138	0.001288	0.008284	Tsku
ENSMUSG00000049680	328.9379	436.3955	−0.40782	0.001348	0.008612	Urgcp
ENSMUSG00000049721	200.2862	313.1749	−0.64491	3.49E-06	4.88E-05	Gal3st1
ENSMUSG00000049764	210.8956	291.0503	−0.46474	0.001362	0.008685	Zfp280b
ENSMUSG00000049811	22.57591	65.2398	−1.531	6.07E-07	1.03E-05	Fam161a
ENSMUSG00000049892	105.6827	161.1723	−0.60886	0.000939	0.006371	Rasd1
ENSMUSG00000049922	1783.73	2374.637	−0.41281	1.02E-05	0.000126	Slc35c1
ENSMUSG00000050002	169.8946	262.2495	−0.6263	5.13E-05	0.000522	Idnk
ENSMUSG00000050014	12.01219	43.76677	−1.8653	5.03E-07	8.60E-06	Apol10b
ENSMUSG00000050029	505.2565	651.5067	−0.36676	0.006214	0.030754	Rap2c
ENSMUSG00000050069	119.5872	188.5816	−0.65713	0.000127	0.001153	Grem2
ENSMUSG00000050144	276.7067	359.8818	−0.37917	0.00757	0.036231	Slc25a44
ENSMUSG00000050270	53.59544	95.55233	−0.83418	0.00079	0.005503	Tmem220
ENSMUSG00000050288	96.40447	142.3742	−0.56252	0.002925	0.016466	Fzd2
ENSMUSG00000050315	957.3145	1272.834	−0.41098	0.001537	0.009638	Synpo2
ENSMUSG00000050373	90.04944	127.7668	−0.50472	0.010466	0.047156	Snx21
ENSMUSG00000050390	2251.133	2782.665	−0.30582	0.004716	0.024429	C77080
ENSMUSG00000050503	77.73043	130.9557	−0.75253	0.000133	0.001202	Fbxl22
ENSMUSG00000050705	277.8674	492.6062	−0.82604	1.25E-10	4.36E-09	2310061I04Rik
ENSMUSG00000050777	282.8724	425.889	−0.59033	6.30E-06	8.23E-05	Tmem37
ENSMUSG00000050866	1389.563	1812.783	−0.38358	0.002413	0.014053	Clrn3
ENSMUSG00000050965	589.0611	997.7197	−0.76022	2.68E-13	1.36E-11	Prkca
ENSMUSG00000050982	825.3339	4143.337	−2.3277	1.64E-15	1.09E-13	Apol10a
ENSMUSG00000051314	142.7699	190.833	−0.41862	0.007938	0.037678	Ffar2
ENSMUSG00000051339	780.8888	1149.41	−0.5577	7.39E-05	0.000714	2900026A02Rik
ENSMUSG00000051403	703.588	967.6072	−0.45969	1.78E-05	0.000205	Ppp1r37

ENSMUSG00000051452	12.88026	35.70818	−1.4711	0.000361	0.002829	Gm11437
ENSMUSG00000051483	1142.905	2170.037	−0.92501	2.92E-22	3.73E-20	Cbr1
ENSMUSG00000051502	51.93122	84.08402	−0.69523	0.002803	0.015904	Ufsp1
ENSMUSG00000051517	131.1294	218.4964	−0.73662	1.98E-06	2.97E-05	Arhgef39
ENSMUSG00000051652	36.36286	60.96052	−0.74541	0.00664	0.032574	Lrrc3
ENSMUSG00000051726	74.34416	189.9635	−1.3534	5.93E-13	2.89E-11	Kcnf1
ENSMUSG00000051811	19.42465	41.05907	−1.0798	0.004001	0.021369	Cox6b2
ENSMUSG00000051977	42.58105	74.55989	−0.80819	0.001893	0.011501	Prdm9
ENSMUSG00000052026	34.12507	64.34612	−0.91502	0.001984	0.011992	Slc6a7
ENSMUSG00000052085	189.6965	312.6718	−0.72096	2.92E-07	5.31E-06	Dock8
ENSMUSG00000052102	218.9289	286.1332	−0.38622	0.008089	0.038313	Gnpda1
ENSMUSG00000052214	506.0026	649.1562	−0.35942	0.002396	0.013984	Opa3
ENSMUSG00000052296	1325.15	1664.566	−0.32899	0.000489	0.003663	Ppp6r1
ENSMUSG00000052336	101.5917	156.9811	−0.62781	0.002021	0.012184	Cx3cr1
ENSMUSG00000052397	5813.66	7325.255	−0.33343	0.001341	0.008581	Ezr
ENSMUSG00000052485	460.6341	843.1629	−0.87219	8.69E-16	5.96E-14	Tmem171
ENSMUSG00000052488	347.2134	438.5567	−0.33694	0.00853	0.039952	Cherp
ENSMUSG00000052566	633.5563	774.4738	−0.28974	0.007055	0.034263	Hook2
ENSMUSG00000052595	138.0073	314.345	−1.1876	4.97E-15	3.12E-13	A1cf
ENSMUSG00000052738	2130.639	2528.569	−0.24704	0.005462	0.027569	Suclg1
ENSMUSG00000052776	283.3478	385.52	−0.44423	0.000611	0.004448	Oas1a
ENSMUSG00000053182	53.07292	81.66761	−0.62179	0.007419	0.035602	Gm609
ENSMUSG00000053199	12.50145	105.3235	−3.0747	7.13E-16	4.91E-14	Arhgap20
ENSMUSG00000053253	1184.641	1409.783	−0.25102	0.008806	0.04102	Ndfip2
ENSMUSG00000053279	329.3031	1102.7	−1.7436	1.67E-10	5.71E-09	Aldh1a1
ENSMUSG00000053286	208.6387	352.2228	−0.75548	4.85E-08	1.05E-06	Trmt11
ENSMUSG00000053329	331.6188	422.0706	−0.34796	0.007813	0.037231	D10Jhu81e
ENSMUSG00000053436	652.5164	832.9039	−0.35214	0.00161	0.010035	Mapk14
ENSMUSG00000053460	108.7953	173.9611	−0.67715	0.000199	0.001705	Ggcx
ENSMUSG00000053604	296.801	385.6851	−0.37793	0.002097	0.012566	Rpia
ENSMUSG00000053768	1129.917	1459.324	−0.36908	9.73E-05	0.000911	Chchd3
ENSMUSG00000053846	216.4617	460.6818	−1.0897	2.14E-16	1.54E-14	Lipg
ENSMUSG00000053963	165.914	242.0932	−0.54513	0.00044	0.003353	6330403A02Rik
ENSMUSG00000053965	876.5804	1212.487	−0.46801	3.42E-06	4.80E-05	Pde5a
ENSMUSG00000053977	21.96918	42.35906	−0.94719	0.004414	0.023122	Cd8a
ENSMUSG00000054072	44.35576	545.5667	−3.6206	5.91E-79	6.59E-76	Iigp1
ENSMUSG00000054099	116.743	201.2361	−0.78555	7.93E-06	0.000101	Slc25a40
ENSMUSG00000054128	39.43706	331.3398	−3.0707	0.000246	0.002037	H2-T3
ENSMUSG00000054161	2525.891	2985.935	−0.24139	0.005332	0.027019	Fam83e
ENSMUSG00000054199	638.0245	772.7026	−0.2763	0.009103	0.042152	Gon4l
ENSMUSG00000054385	18.39354	36.58948	−0.99223	0.006336	0.031295	Ceacam2
ENSMUSG00000054387	783.8682	1121.452	−0.51669	7.55E-07	1.25E-05	Mdm4
ENSMUSG00000054452	3144.27	4280.707	−0.44512	2.96E-07	5.37E-06	Aes
ENSMUSG00000054469	561.696	847.2984	−0.59308	2.04E-08	4.73E-07	Lclat1
ENSMUSG00000054509	575.5755	749.5909	−0.3811	0.000433	0.00331	Parp4
ENSMUSG00000054545	6.870615	37.67339	−2.455	2.13E-08	4.91E-07	Ugt1a6a
ENSMUSG00000054619	385.7161	629.8561	−0.70748	1.98E-09	5.70E-08	Mettl7a1

ENSMUSG00000054630	3.131927	43.7045	−3.8027	0.01017	0.046102	Ugt2b5
ENSMUSG00000054640	71.95577	127.7234	−0.82784	2.16E-05	0.000242	Slc8a1
ENSMUSG00000054662	415.3976	741.3332	−0.83563	8.79E-14	4.70E-12	Ano9
ENSMUSG00000054733	252.9945	369.0831	−0.54484	0.000214	0.001811	Msra
ENSMUSG00000054874	702.3023	865.0861	−0.30075	0.006714	0.032883	Pcnxl3
ENSMUSG00000055065	2003.762	2827.253	−0.49669	1.94E-07	3.68E-06	Ddx17
ENSMUSG00000055116	147.3834	317.6404	−1.1078	6.75E-13	3.28E-11	Arntl
ENSMUSG00000055210	259.9039	438.1513	−0.75345	2.41E-09	6.84E-08	Foxd2
ENSMUSG00000055322	1221.183	1901.685	−0.639	2.11E-11	8.26E-10	Tns1
ENSMUSG00000055553	320.9764	411.4783	−0.35835	0.003456	0.018896	Kxd1
ENSMUSG00000055632	224.9965	503.4506	−1.1619	4.16E-14	2.35E-12	Hmcn2
ENSMUSG00000055653	30.75822	74.42719	−1.2749	7.16E-06	9.21E-05	Gpc3
ENSMUSG00000055670	1136.349	1731.914	−0.60796	2.41E-09	6.84E-08	Zzef1
ENSMUSG00000055675	268.7001	818.6132	−1.6072	1.56E-38	4.87E-36	Kbtbd11
ENSMUSG00000055681	1211.011	1432.053	−0.24187	0.010142	0.045999	Cope
ENSMUSG00000055730	515.7259	878.5708	−0.76855	0.000857	0.005889	Ces2a
ENSMUSG00000055827	150.7946	307.8631	−1.0297	1.25E-05	0.000151	Gsdmc3
ENSMUSG00000055850	616.3364	858.8774	−0.47873	8.34E-06	0.000106	Rnf181
ENSMUSG00000056116	280.3982	466.4605	−0.73428	2.54E-09	7.19E-08	H2-T22
ENSMUSG00000056228	93.04434	143.8198	−0.62827	0.001737	0.010722	Cars2
ENSMUSG00000056268	434.1627	556.5593	−0.3583	0.002122	0.012674	Dennd1b
ENSMUSG00000056293	545.2075	790.0436	−0.53513	0.001085	0.007185	Gsdmc2
ENSMUSG00000056313	632.3223	868.6254	−0.45807	2.57E-05	0.000283	1810011O10Rik
ENSMUSG00000056553	167.108	238.6316	−0.514	0.001008	0.006758	Ptprn2
ENSMUSG00000056666	344.5695	579.4682	−0.74993	1.08E-10	3.78E-09	Retsat
ENSMUSG00000056673	93.47583	439.6861	−2.2338	1.20E-44	5.66E-42	Kdm5d
ENSMUSG00000056698	86.33001	132.4906	−0.61796	0.001099	0.007248	Elmod3
ENSMUSG00000056763	159.2061	269.2719	−0.75817	1.28E-06	2.00E-05	Cspp1
ENSMUSG00000056973	92.35899	160.8817	−0.80068	9.53E-06	0.000118	Ces1d
ENSMUSG00000057069	99.69954	150.4964	−0.59407	0.001277	0.008219	Ero1lb
ENSMUSG00000057110	482.4273	666.0577	−0.46534	5.94E-05	0.000593	Cntrl
ENSMUSG00000057137	21.00999	42.33362	−1.0107	0.002177	0.012927	Tmem140
ENSMUSG00000057229	253.0205	367.7751	−0.53957	6.03E-05	0.000601	Atp5sl
ENSMUSG00000057286	316.3501	491.6546	−0.63612	2.84E-07	5.19E-06	St6galnac2
ENSMUSG00000057315	105.676	147.1739	−0.47787	0.005808	0.029004	Arhgap24
ENSMUSG00000057342	525.7397	765.2941	−0.54167	6.25E-07	1.06E-05	Sphk2
ENSMUSG00000057596	188.4111	306.403	−0.70155	3.81E-05	0.000402	Trim30d
ENSMUSG00000057789	1066.715	1467.207	−0.4599	2.89E-06	4.13E-05	Bak1
ENSMUSG00000057880	903.9442	1955.748	−1.1134	5.14E-30	9.75E-28	Abat
ENSMUSG00000057897	68.66767	108.2584	−0.65678	0.002933	0.016504	Camk2b
ENSMUSG00000057963	1268.432	1846.881	−0.54204	0.000239	0.00199	Itpk1
ENSMUSG00000058022	180.2815	357.6997	−0.9885	1.67E-12	7.84E-11	Adtrp
ENSMUSG00000058056	361.9859	481.4109	−0.41134	0.001005	0.006743	Palld
ENSMUSG00000058063	718.3127	950.5565	−0.40416	6.28E-05	0.000621	Trim31
ENSMUSG00000058076	1551.685	2067.099	−0.41377	8.23E-06	0.000105	Sdhc
ENSMUSG00000058135	609.125	1109.731	−0.8654	8.71E-17	6.61E-15	Gstm1
ENSMUSG00000058163	47.06336	164.5152	−1.8055	3.06E-19	3.00E-17	Gm5431

ENSMUSG00000058254	540.5432	684.1345	−0.33987	0.002398	0.013989	Tspan7
ENSMUSG00000058290	157.6929	229.4249	−0.5409	0.001042	0.006959	Espl1
ENSMUSG00000058301	709.0625	884.3322	−0.31868	0.005242	0.026659	Upf1
ENSMUSG00000058435	450.6389	756.2583	−0.74691	2.64E-07	4.87E-06	Btnl4
ENSMUSG00000058454	626.8362	1101.769	−0.81366	4.35E-14	2.45E-12	Dhcr7
ENSMUSG00000058594	703.3242	930.387	−0.40364	6.18E-05	0.000613	Fbxo18
ENSMUSG00000058706	263.3425	359.4687	−0.44893	0.000632	0.004576	0610030E20Rik
ENSMUSG00000058793	777.4943	986.3844	−0.34332	0.001049	0.006988	Cds2
ENSMUSG00000058921	187.1588	339.6022	−0.85958	3.52E-08	7.80E-07	Slc10a5
ENSMUSG00000058997	774.1455	1180.54	−0.60877	1.08E-09	3.22E-08	Vwa8
ENSMUSG00000059273	518.3462	682.9463	−0.39786	0.000276	0.002238	Zc3h4
ENSMUSG00000059409	462.2588	626.7516	−0.43919	0.000128	0.001164	Ppp2r5d
ENSMUSG00000059430	4357.861	5214.317	−0.25886	0.002313	0.013605	Actg2
ENSMUSG00000059436	1143.053	1632.191	−0.51392	9.79E-08	1.97E-06	Max
ENSMUSG00000059824	122.2106	192.4013	−0.65475	0.010131	0.045981	Dbp
ENSMUSG00000059851	386.9292	605.8195	−0.64682	1.76E-07	3.37E-06	Suv420h2
ENSMUSG00000059883	293.624	401.9728	−0.45313	0.00025	0.002067	Irak4
ENSMUSG00000059890	737.0596	995.0817	−0.43303	4.24E-05	0.000442	Ube4a
ENSMUSG00000060012	784.2075	1064.503	−0.44087	2.34E-05	0.000259	Kif13b
ENSMUSG00000060176	2.851384	19.51516	−2.7749	2.10E-05	0.000235	Kif27
ENSMUSG00000060206	67.47346	133.0929	−0.98004	1.67E-06	2.56E-05	Zfp462
ENSMUSG00000060224	64.66776	97.81587	−0.59702	0.007805	0.037205	Pyroxd2
ENSMUSG00000060279	438.6063	560.3426	−0.35338	0.003293	0.018158	Ap2a1
ENSMUSG00000060477	289.2273	391.7164	−0.43761	0.000822	0.005681	Irak2
ENSMUSG00000060508	113.702	317.4273	−1.4812	1.70E-21	2.01E-19	Nlrp9b
ENSMUSG00000060550	156.0675	826.6094	−2.405	3.27E-73	3.07E-70	H2-Q7
ENSMUSG00000060586	1021.171	2894.469	−1.5031	2.14E-14	1.25E-12	H2-Eb1
ENSMUSG00000060600	224.9576	529.6686	−1.2354	1.20E-20	1.29E-18	Eno3
ENSMUSG00000060657	674.9765	957.5419	−0.5045	3.94E-06	5.43E-05	Marf1
ENSMUSG00000060675	478.003	675.5776	−0.4991	7.21E-06	9.28E-05	Pla2g16
ENSMUSG00000060716	612.1995	824.8159	−0.43007	6.51E-05	0.000641	Plekhh1
ENSMUSG00000060733	1725.059	2319.502	−0.42717	5.46E-06	7.25E-05	Ipmk
ENSMUSG00000060802	2777.519	9436.56	−1.7645	3.93E-87	5.01E-84	B2m
ENSMUSG00000060862	89.46892	160.8122	−0.84592	9.53E-06	0.000118	Zbtb40
ENSMUSG00000060961	347.6317	703.451	−1.0169	8.97E-14	4.78E-12	Slc4a4
ENSMUSG00000061028	202.8253	278.5319	−0.4576	0.00325	0.01796	Clasrp
ENSMUSG00000061100	8.319116	21.63256	−1.3787	0.005889	0.029343	Retnla
ENSMUSG00000061130	1016.034	1199.473	−0.23945	0.01125	0.049945	Ppm1b
ENSMUSG00000061232	4484.095	12059.49	−1.4273	7.29E-12	3.12E-10	H2-K1
ENSMUSG00000061353	366.69	655.9681	−0.83906	5.11E-07	8.74E-06	Cxcl12
ENSMUSG00000061411	356.303	621.585	−0.80285	1.88E-11	7.41E-10	Nol4l
ENSMUSG00000061451	9.278324	24.34026	−1.3914	0.002858	0.016145	Tmem151a
ENSMUSG00000061455	352.9606	526.548	−0.57706	1.95E-06	2.94E-05	Stx17
ENSMUSG00000061474	258.3252	385.0354	−0.5758	0.000243	0.002017	Mrps36
ENSMUSG00000061479	278.9964	364.3116	−0.38493	0.00829	0.039035	Snrpa
ENSMUSG00000061531	889.9684	1590.71	−0.83784	1.37E-17	1.16E-15	Tmem236
ENSMUSG00000061740	66.54076	161.0599	−1.2753	6.53E-09	1.67E-07	Cyp2d22

ENSMUSG00000061780	145.2758	316.4185	−1.123	5.58E-14	3.05E-12	Cfd
ENSMUSG00000061825	490.8119	935.4997	−0.93057	6.67E-18	5.83E-16	Ces2c
ENSMUSG00000061838	1666.445	2271.719	−0.44701	1.55E-06	2.39E-05	Suclg2
ENSMUSG00000061859	642.1893	814.5482	−0.343	0.001994	0.012043	Inadl
ENSMUSG00000061904	5536.506	7414.545	−0.42138	1.36E-06	2.12E-05	Slc25a3
ENSMUSG00000061959	27.11076	81.91477	−1.5953	7.77E-09	1.96E-07	Ces1e
ENSMUSG00000062075	463.1285	590.2079	−0.34981	0.004177	0.022096	Lmnb2
ENSMUSG00000062181	0.443666	9.009418	−4.3439	0.000252	0.002076	Ces3b
ENSMUSG00000062312	1063.838	1582.594	−0.57301	0.000151	0.001337	Erbb2
ENSMUSG00000062410	115.7048	639.6318	−2.4668	1.31E-36	3.76E-34	Hsd3b3
ENSMUSG00000062526	98.38784	149.9888	−0.6083	0.001054	0.007019	Mppe1
ENSMUSG00000062545	23.09147	50.89861	−1.1403	0.000668	0.004786	Tlr12
ENSMUSG00000062638	53.29599	403.4688	−2.9204	3.32E-09	9.13E-08	Btnl1
ENSMUSG00000062646	122.1317	175.6353	−0.52414	0.0015	0.009454	Ganc
ENSMUSG00000062760	55.66994	92.38909	−0.73082	0.001495	0.009426	1810041L15Rik
ENSMUSG00000062866	230.2291	341.4488	−0.5686	4.59E-05	0.000473	Phactr2
ENSMUSG00000062908	942.2004	1203.807	−0.3535	0.000382	0.002966	Acadm
ENSMUSG00000062980	150.7558	223.9653	−0.57106	0.000206	0.001756	Cped1
ENSMUSG00000062981	552.4646	738.3932	−0.41851	0.000168	0.001473	Mrpl42
ENSMUSG00000063146	461.1103	618.3561	−0.42333	0.000214	0.001811	Clip2
ENSMUSG00000063268	233.9746	337.5088	−0.52857	0.000689	0.004905	Parp10
ENSMUSG00000063430	55.80671	116.7032	−1.0643	6.97E-07	1.17E-05	Wscd2
ENSMUSG00000063450	688.4621	1158.109	−0.75032	3.23E-13	1.63E-11	Syne2
ENSMUSG00000063694	1168.891	1478.611	−0.3391	0.000469	0.003534	Cycs
ENSMUSG00000063730	54.33899	185.4675	−1.7711	2.21E-17	1.82E-15	Hsd3b2
ENSMUSG00000063873	130.5752	247.8369	−0.92451	5.98E-09	1.55E-07	Slc24a3
ENSMUSG00000063952	819.4886	1206.179	−0.55765	4.81E-08	1.04E-06	Brpf3
ENSMUSG00000064140	1.285397	12.7315	−3.3081	0.003991	0.02133	Trim38
ENSMUSG00000064254	1384.486	3085.055	−1.1559	2.54E-25	3.94E-23	Ethe1
ENSMUSG00000065979	655.3084	849.4727	−0.37439	0.000381	0.002962	Cpped1
ENSMUSG00000066026	279.4137	405.368	−0.53683	0.003673	0.019919	Dhrs3
ENSMUSG00000066036	1665.284	2160.906	−0.37587	5.47E-05	0.000553	Ubr4
ENSMUSG00000066090	241.23	337.9548	−0.48642	0.001825	0.011177	Insl5
ENSMUSG00000066151	405.4987	516.4768	−0.34901	0.002117	0.012654	Fkbp15
ENSMUSG00000066258	216.8334	313.9854	−0.53411	7.28E-05	0.000704	Trim12a
ENSMUSG00000066306	1257.422	1656.254	−0.39745	4.45E-05	0.00046	Numa1
ENSMUSG00000066440	200.4563	266.1796	−0.40911	0.005301	0.026886	Zfyve26
ENSMUSG00000066861	148.9427	297.3613	−0.99746	1.73E-11	6.90E-10	Oas1g
ENSMUSG00000066894	652.1975	1039.756	−0.67286	1.68E-10	5.73E-09	Vsig10
ENSMUSG00000066900	827.1031	1100.782	−0.41239	7.18E-05	0.000695	Suds3
ENSMUSG00000067206	331.9386	424.4369	−0.35463	0.004051	0.021537	Lrrc66
ENSMUSG00000067212	943.4489	1812.519	−0.94198	1.67E-10	5.72E-09	H2-T23
ENSMUSG00000067235	67.42789	175.1547	−1.3772	5.04E-13	2.49E-11	H2-Q10
ENSMUSG00000067629	29.3816	51.42567	−0.80758	0.01043	0.047053	Syngap1
ENSMUSG00000068040	1607.092	2077.752	−0.37057	5.62E-05	0.000565	Tm9sf4
ENSMUSG00000068086	194.0418	456.9263	−1.2356	5.25E-16	3.66E-14	Cyp2d9
ENSMUSG00000068245	78.45461	144.1442	−0.87758	4.18E-06	5.72E-05	Phf11d

ENSMUSG00000068284	850.5463	1455.327	−0.77488	1.26E-14	7.64E-13	Gm608
ENSMUSG00000068299	210.1138	326.6903	−0.63675	8.55E-06	0.000108	1700019G17Rik
ENSMUSG00000068328	593.6302	764.3785	−0.36472	0.000676	0.004827	Aup1
ENSMUSG00000068329	130.5559	196.6994	−0.59132	0.003047	0.01704	Htra2
ENSMUSG00000068457	11.1512	230.5022	−4.3695	1.48E-66	1.20E-63	Uty
ENSMUSG00000068551	219.2674	324.8701	−0.56717	4.63E-05	0.000477	Zfp467
ENSMUSG00000068606	2.244624	27.94127	−3.6379	5.91E-09	1.54E-07	Gm4841
ENSMUSG00000068747	3128.405	3987.55	−0.35008	8.54E-05	0.000813	Sort1
ENSMUSG00000068854	65.34635	111.96	−0.7768	0.000435	0.003319	Hist2h2be
ENSMUSG00000068874	529.5822	3406.362	−2.6853	2.55E-55	1.62E-52	Selenbp1
ENSMUSG00000068876	1333.608	1882.037	−0.49696	2.80E-07	5.14E-06	Cgn
ENSMUSG00000069045	5.331025	437.8383	−6.3598	########	########	Ddx3y
ENSMUSG00000069049	14.59015	461.2287	−4.9824	########	########	Eif2s3y
ENSMUSG00000069053	3.647507	35.91121	−3.2995	4.06E-10	1.30E-08	Uba1y
ENSMUSG00000069114	165.1107	244.9263	−0.56891	0.000188	0.001628	Zbtb10
ENSMUSG00000069844	262.3974	367.9336	−0.48769	0.000303	0.00243	Sco1
ENSMUSG00000069874	205.7733	871.5144	−2.0825	4.80E-66	3.73E-63	Irgm2
ENSMUSG00000069917	55.2262	89.3516	−0.69414	0.003683	0.019955	Hba-a2
ENSMUSG00000069919	50.78261	101.0621	−0.99284	1.33E-05	0.000158	Hba-a1
ENSMUSG00000070031	79.29627	117.7929	−0.57093	0.006714	0.032883	Sp140
ENSMUSG00000070327	1645.292	2074.377	−0.33433	0.000253	0.002085	Rnf213
ENSMUSG00000070390	89.48128	179.0082	−1.0004	1.25E-08	3.04E-07	Nlrp1b
ENSMUSG00000070427	57.56215	144.8555	−1.3314	2.71E-06	3.91E-05	Il18bp
ENSMUSG00000070563	68.94125	99.95964	−0.53598	0.007375	0.035439	Spaca4
ENSMUSG00000070661	699.6478	1081.277	−0.62804	2.32E-06	3.40E-05	Rnf186
ENSMUSG00000070704	2.407709	11.12716	−2.2084	0.007671	0.03665	Ugt2b36
ENSMUSG00000070730	374.5652	683.885	−0.86854	3.47E-14	1.98E-12	Rmdn3
ENSMUSG00000070733	1025.158	1481.389	−0.5311	1.03E-07	2.06E-06	Fryl
ENSMUSG00000070777	1003.679	1599.604	−0.67242	4.61E-12	2.04E-10	Ceacam20
ENSMUSG00000070780	1178.204	1808.332	−0.61807	0.000343	0.002702	Rbm47
ENSMUSG00000070985	60.36783	130.4809	−1.112	9.47E-08	1.91E-06	Acnat1
ENSMUSG00000071014	550.469	741.6082	−0.43	0.000966	0.006517	Ndufb6
ENSMUSG00000071054	734.6458	915.0629	−0.31682	0.004038	0.021479	Safb
ENSMUSG00000071176	55.87865	84.29871	−0.59321	0.010402	0.046975	Arhgef10
ENSMUSG00000071203	493.083	697.4263	−0.50021	1.39E-05	0.000164	Naip5
ENSMUSG00000071335	8.227908	26.69881	−1.6982	0.000461	0.003483	G630090E17Rik
ENSMUSG00000071379	446.4819	584.6908	−0.38907	0.000751	0.005275	Hpcal1
ENSMUSG00000071551	487.8483	801.6911	−0.71661	6.23E-11	2.25E-09	Akr1c19
ENSMUSG00000071604	49.80407	208.4084	−2.0651	2.58E-26	4.23E-24	Fam189a2
ENSMUSG00000071650	1936.868	2416.08	−0.31894	0.00075	0.005268	Ganab
ENSMUSG00000071657	360.7972	473.6826	−0.39273	0.000734	0.005185	Bscl2
ENSMUSG00000071711	356.8692	836.7103	−1.2293	4.22E-18	3.77E-16	Mpst
ENSMUSG00000072612	6.498891	18.62114	−1.5187	0.009819	0.04477	Gm10382
ENSMUSG00000072946	542.8338	697.6833	−0.36206	0.001141	0.007488	Ptgr2
ENSMUSG00000073043	350.0967	543.3536	−0.63414	1.12E-07	2.21E-06	Atoh1
ENSMUSG00000073409	95.09973	498.8395	−2.3911	6.18E-59	4.24E-56	H2-Q6
ENSMUSG00000073411	5938.84	9469.625	−0.67313	9.66E-09	2.39E-07	H2-D1

ENSMUSG00000073421	1055.995	3485.154	−1.7226	9.14E-08	1.85E-06	H2-Ab1
ENSMUSG00000073555	5.983275	47.76048	−2.9968	4.53E-12	2.01E-10	Gm4951
ENSMUSG00000073557	585.3432	928.3311	−0.66536	7.69E-10	2.34E-08	Ppp1r12b
ENSMUSG00000073608	94.94904	143.7817	−0.59865	0.001025	0.006852	Gm6086
ENSMUSG00000073609	157.686	216.4278	−0.45683	0.004228	0.022313	D2hgdh
ENSMUSG00000073643	109.8459	153.641	−0.48408	0.010307	0.046624	Wdfy1
ENSMUSG00000073664	692.6642	905.6072	−0.38673	0.000328	0.002604	Nbeal1
ENSMUSG00000073676	1051.652	1285.326	−0.28948	0.003678	0.019941	Hspe1
ENSMUSG00000073802	225.826	350.1713	−0.63285	0.007831	0.037296	Cdkn2b
ENSMUSG00000073838	236.1527	461.2251	−0.96575	2.72E-09	7.64E-08	Tufm
ENSMUSG00000073910	212.6193	318.5511	−0.58325	0.00014	0.001256	Mob3b
ENSMUSG00000074004	57.02744	140.98	−1.3058	5.33E-10	1.67E-08	B3gnt6
ENSMUSG00000074151	66.37049	274.2589	−2.0469	3.43E-21	3.95E-19	Nlrc5
ENSMUSG00000074218	131.7957	237.7393	−0.85108	2.47E-06	3.60E-05	Cox7a1
ENSMUSG00000074227	6021.675	8296.489	−0.46234	5.78E-08	1.23E-06	Spint2
ENSMUSG00000074272	11548.8	22331.86	−0.95136	2.49E-29	4.58E-27	Ceacam1
ENSMUSG00000074305	248.956	349.1667	−0.48802	0.000363	0.002846	Peak1
ENSMUSG00000074345	7.431785	20.35864	−1.4539	0.004349	0.022861	Tnfaip8l3
ENSMUSG00000074582	868.8484	1123.39	−0.37068	0.000306	0.00245	Arfgef2
ENSMUSG00000074604	914.1901	1172.841	−0.35944	0.000239	0.00199	Mgst2
ENSMUSG00000074622	261.3923	406.1576	−0.63582	2.55E-06	3.70E-05	Mafb
ENSMUSG00000074647	105.8983	152.2776	−0.52402	0.005709	0.028627	Fam83c
ENSMUSG00000074743	232.153	307.3885	−0.40499	0.005281	0.026831	Thbd
ENSMUSG00000074818	17.18007	74.13582	−2.1094	1.78E-11	7.06E-10	Pdzd7
ENSMUSG00000074862	13.29755	28.8667	−1.1182	0.008106	0.038385	BC025920
ENSMUSG00000074882	54.10408	88.64865	−0.71236	0.002234	0.0132	Cyp2c68
ENSMUSG00000074971	29.81839	67.8966	−1.1871	1.33E-05	0.000158	Fibin
ENSMUSG00000075415	516.2526	661.7886	−0.35829	0.001411	0.008951	Fnbp1
ENSMUSG00000075702	366.6193	490.5735	−0.42019	0.011102	0.04941	Selm
ENSMUSG00000076435	451.8904	955.6033	−1.0804	6.33E-08	1.34E-06	Acsf2
ENSMUSG00000078485	58.50194	97.03661	−0.73004	0.001739	0.010727	Plekhn1
ENSMUSG00000078490	9.604514	33.99582	−1.8236	1.88E-05	0.000215	Cfap74
ENSMUSG00000078670	379.0858	485.7254	−0.35762	0.002128	0.012692	Fam174b
ENSMUSG00000078695	601.2252	1175.003	−0.96669	1.13E-06	1.78E-05	Cisd3
ENSMUSG00000078716	45.90238	74.80171	−0.7045	0.005269	0.026783	Tmem8b
ENSMUSG00000078765	157.3209	235.4875	−0.58194	0.000318	0.002534	U2af1l4
ENSMUSG00000078813	68.52389	102.5336	−0.58142	0.005727	0.028691	Leng1
ENSMUSG00000078853	108.2537	509.9658	−2.236	7.31E-10	2.24E-08	Igtp
ENSMUSG00000078920	100.0714	602.6096	−2.5902	1.85E-75	1.84E-72	Ifi47
ENSMUSG00000078921	3.693097	37.24198	−3.334	8.98E-11	3.19E-09	Tgtp2
ENSMUSG00000078922	2.851366	30.20515	−3.4051	2.23E-06	3.26E-05	Tgtp1
ENSMUSG00000078942	819.8726	1031.436	−0.33118	0.001326	0.008491	Naip6
ENSMUSG00000079037	727.089	977.0924	−0.42636	2.43E-05	0.000268	Prnp
ENSMUSG00000079111	1163.597	1434.967	−0.30243	0.001961	0.011866	Kdelr2
ENSMUSG00000079173	185.358	563.5491	−1.6042	2.62E-18	2.42E-16	Zan
ENSMUSG00000079197	1105.478	1606.047	−0.53884	1.59E-08	3.76E-07	Psme2
ENSMUSG00000079355	45.50434	80.00709	−0.81412	0.000943	0.006392	Ackr4

ENSMUSG00000079363	41.81129	151.2824	−1.8553	2.06E-17	1.72E-15	Gbp4
ENSMUSG00000079429	76.00127	115.823	−0.60783	0.002061	0.012387	Mroh2a
ENSMUSG00000079442	232.6296	327.3049	−0.4926	0.000229	0.001919	St6galnac4
ENSMUSG00000079487	458.2272	592.6587	−0.37114	0.002192	0.012996	Med12
ENSMUSG00000079491	7.992955	23.09106	−1.5305	0.001835	0.011233	H2-T10
ENSMUSG00000079507	48.64347	227.7002	−2.2268	0.001708	0.010572	H2-Q1
ENSMUSG00000079547	68.48524	474.1103	−2.7914	6.81E-16	4.73E-14	H2-DMb1
ENSMUSG00000079620	1069.036	2053.129	−0.94151	7.33E-12	3.13E-10	Muc4
ENSMUSG00000081769	11.89474	35.58778	−1.5811	4.36E-05	0.000452	Gm12216
ENSMUSG00000084174	509.4792	819.4455	−0.68562	4.75E-10	1.50E-08	Sycn
ENSMUSG00000087153	315.7242	423.3453	−0.42317	0.001155	0.007567	Gm6483
ENSMUSG00000089960	27.13706	118.5313	−2.1269	3.47E-06	4.86E-05	Ugt1a1
ENSMUSG00000090035	1556.008	1848.762	−0.24871	0.006382	0.031454	Galnt4
ENSMUSG00000090124	495.1695	848.2502	−0.77657	7.29E-13	3.51E-11	Ugt1a7c
ENSMUSG00000090150	794.9396	1097.812	−0.46571	2.96E-06	4.23E-05	Acad11
ENSMUSG00000090210	7.829814	20.76398	−1.407	0.008716	0.040635	Itga10
ENSMUSG00000090213	394.635	537.2278	−0.44501	0.000142	0.001269	Tmem189
ENSMUSG00000090264	65.50256	172.8446	−1.3999	1.96E-13	1.01E-11	Eif4ebp3
ENSMUSG00000090700	348.5961	512.0009	−0.55459	3.38E-06	4.76E-05	Cyp4f40
ENSMUSG00000090935	610.5354	769.354	−0.33357	0.001734	0.010712	Synj2bp
ENSMUSG00000090942	6.661967	31.41585	−2.2375	2.90E-06	4.15E-05	F830016B08Rik
ENSMUSG00000091649	19.44394	52.77615	−1.4406	5.42E-06	7.21E-05	Phf11b
ENSMUSG00000091705	279.3309	1082.687	−1.9546	0.002656	0.015217	H2-Q2
ENSMUSG00000091811	288.4505	364.9784	−0.33949	0.010688	0.047986	Inafm1
ENSMUSG00000092008	0	5.135478	#NAME?	0.004382	0.022977	Cyp2c69
ENSMUSG00000092618	589.2144	1460.348	−1.3094	1.70E-23	2.41E-21	Btnl6
ENSMUSG00000093954	19.86833	54.55715	−1.4573	1.92E-05	0.000218	Gm16867
ENSMUSG00000094030	27.11072	50.46141	−0.89632	0.003696	0.019995	Gm21833
ENSMUSG00000094091	4.299857	63.95982	−3.8948	5.77E-21	6.48E-19	Gm21885
ENSMUSG00000094114	17.66931	38.25004	−1.1142	0.00369	0.019979	Gm21967
ENSMUSG00000094559	917.8432	1777.085	−0.95319	2.35E-22	3.03E-20	Cyp2d34
ENSMUSG00000094806	268.6664	352.9238	−0.39354	0.0034	0.018633	Cyp2d10
ENSMUSG00000094840	380.8815	1048.696	−1.4612	1.97E-17	1.65E-15	Muc3a
ENSMUSG00000095026	297.9045	377.6972	−0.34238	0.005733	0.028703	Gm3336
ENSMUSG00000096188	1915.052	2365.702	−0.30488	0.000904	0.006158	Cmtm4
ENSMUSG00000096463	43.02478	71.39067	−0.73057	0.003538	0.019276	Gm21750
ENSMUSG00000096727	164.7654	710.0749	−2.1076	1.23E-13	6.47E-12	Psmb9
ENSMUSG00000096768	274.1419	447.522	−0.70704	0.00096	0.006484	Erdr1
ENSMUSG00000096852	42.78988	225.0931	−2.3952	9.93E-21	1.07E-18	Cyp2d12

Supplementary Table S4: List of upregulated genes in tumors of ApcMin mice (compared to normal tissues)

Gene_id	read count_ WT_T	read count_ WT_N	log2. Fold_ change.	*p* value	*q* value	Gene Name
ENSMUSG00000000085	31.72023	12.31548	1.3649	9.68E-05	0.000578	Scmh1
ENSMUSG00000000204	69.88174	22.50236	1.6348	2.02E-10	2.55E-09	Slfn4
ENSMUSG00000000385	482.4878	144.2888	1.7415	9.42E-67	7.10E-65	Tmprss2
ENSMUSG00000000394	39.61991	5.625589	2.8161	7.71E-10	9.13E-09	Gcg
ENSMUSG00000000416	17.50082	3.344945	2.3874	0.000134	0.000782	Cttnbp2
ENSMUSG00000000440	43.87358	10.94709	2.0028	2.46E-08	2.49E-07	Pparg
ENSMUSG00000000555	48.85645	23.56666	1.0518	2.56E-05	0.000171	Itga5
ENSMUSG00000000567	43.99511	19.00537	1.2109	1.65E-05	0.000114	Sox9
ENSMUSG00000000628	200.2871	84.38383	1.247	9.94E-21	2.46E-19	Hk2
ENSMUSG00000000805	1112.882	228.5205	2.2839	########	########	Car4
ENSMUSG00000000938	49.82872	0	6.6389	8.22E-15	1.49E-13	Hoxa10
ENSMUSG00000000957	85.31648	24.02278	1.8284	1.03E-13	1.73E-12	Mmp14
ENSMUSG00000000983	21.75449	0	6.1607	2.04E-07	1.86E-06	Wfdc18
ENSMUSG00000001025	541.6746	64.77029	3.064	########	########	S100a6
ENSMUSG00000001225	990.8622	206.3223	2.2638	########	########	Slc26a3
ENSMUSG00000001349	410.6615	150.8266	1.4451	2.22E-47	1.22E-45	Cnn1
ENSMUSG00000001506	210.739	60.81718	1.7929	5.56E-31	1.96E-29	Col1a1
ENSMUSG00000001630	87.62562	23.11053	1.9228	1.11E-14	1.99E-13	Stk38l
ENSMUSG00000001642	80.09055	32.2331	1.3131	1.29E-09	1.50E-08	Akr1b3
ENSMUSG00000001670	31.72023	4.865374	2.7048	6.05E-08	5.91E-07	Tat
ENSMUSG00000001763	11.54568	1.368386	3.0768	0.000604	0.003006	Tspan33
ENSMUSG00000002058	21.14682	6.537846	1.6936	0.000368	0.001933	Unc119
ENSMUSG00000002308	18.35155	5.321503	1.786	0.000653	0.003224	Cd320
ENSMUSG00000003379	19.80995	3.496988	2.502	3.38E-05	0.00022	Cd79a
ENSMUSG00000003518	52.50245	23.87074	1.1371	5.37E-06	4.00E-05	Dusp3
ENSMUSG00000003617	23.45596	8.666448	1.4364	0.000573	0.00287	Cp
ENSMUSG00000003955	101.9666	31.77697	1.682	6.45E-15	1.18E-13	Fam162a
ENSMUSG00000004356	91.02856	23.26257	1.9683	1.65E-15	3.11E-14	Utp20
ENSMUSG00000004462	28.92496	12.92365	1.1623	0.000633	0.003135	Tbccd1
ENSMUSG00000004552	57.72839	1.216344	5.5687	1.42E-17	2.99E-16	Ctse
ENSMUSG00000004558	97.95596	25.23913	1.9565	1.74E-16	3.51E-15	Ndrg2
ENSMUSG00000004730	18.71615	4.257202	2.1363	0.000173	0.000977	Emr1
ENSMUSG00000004929	49.58565	18.3972	1.4304	5.83E-07	4.93E-06	Thop1
ENSMUSG00000005034	181.3279	40.13934	2.1755	4.47E-32	1.66E-30	Prkacb
ENSMUSG00000005087	57.60686	28.73612	1.0034	8.30E-06	6.00E-05	Cd44
ENSMUSG00000005320	15.43475	3.040859	2.3436	0.000375	0.001966	Fgfr4
ENSMUSG00000005355	18.95922	0	5.3773	9.90E-07	8.12E-06	Casp14
ENSMUSG00000005672	56.02692	19.61354	1.5143	4.44E-08	4.40E-07	Kit
ENSMUSG00000005686	52.50245	15.66042	1.7453	1.20E-08	1.27E-07	Ampd3
ENSMUSG00000005803	422.8149	105.5178	2.0025	3.85E-67	2.94E-65	Sqrdl
ENSMUSG00000005823	92.36542	42.72407	1.1123	2.46E-09	2.80E-08	Gpr108
ENSMUSG00000005873	61.25286	26.45547	1.2112	3.71E-07	3.23E-06	Reep5
ENSMUSG00000005958	27.34503	12.16344	1.1687	0.000862	0.004125	Ephb3
ENSMUSG00000006205	37.4323	13.53182	1.4679	1.08E-05	7.62E-05	Htra1
ENSMUSG00000006517	62.10359	28.88816	1.1042	1.11E-06	9.07E-06	Mvd

ENSMUSG00000008035	88.35482	41.05159	1.1059	6.07E-09	6.60E-08	Mid1ip1
ENSMUSG00000008206	77.78141	27.36773	1.5069	1.26E-10	1.62E-09	Cers4
ENSMUSG00000009013	148.2708	59.75288	1.3112	1.57E-16	3.18E-15	Dynll1
ENSMUSG00000009092	13.73328	2.736773	2.3271	0.000823	0.003967	Derl3
ENSMUSG00000009185	19.93149	1.368386	3.8645	1.45E-06	1.17E-05	Ccl8
ENSMUSG00000009356	39.49837	0	8.0212	7.71E-11	1.01E-09	Lpo
ENSMUSG00000010080	33.90783	3.801074	3.1571	3.04E-09	3.42E-08	Epn3
ENSMUSG00000010175	18.71615	4.105159	2.1888	0.000146	0.000842	Prox1
ENSMUSG00000012350	365.0865	103.3892	1.8202	3.53E-53	2.12E-51	Ehf
ENSMUSG00000012428	23.21289	6.993975	1.7307	0.000161	0.000917	Steap4
ENSMUSG00000013089	29.28956	6.385803	2.1974	1.94E-06	1.54E-05	Etv5
ENSMUSG00000013275	31.47716	15.50838	1.0213	0.000881	0.004203	Slc41a1
ENSMUSG00000013338	24.54976	1.064301	4.5277	3.68E-08	3.68E-07	Fer1l4
ENSMUSG00000013629	49.09951	20.37375	1.269	3.07E-06	2.37E-05	Cad
ENSMUSG00000013643	7654.42	2532.883	1.5955	0	0	Lypd8
ENSMUSG00000013653	274.7871	11.09913	4.6298	3.58E-76	3.07E-74	1810065E05Rik
ENSMUSG00000014444	29.7757	12.31548	1.2737	0.000272	0.001467	Piezo1
ENSMUSG00000015189	124.6933	22.04623	2.4998	2.53E-25	7.48E-24	Casd1
ENSMUSG00000016028	86.41028	11.70731	2.8838	4.92E-20	1.18E-18	Celsr1
ENSMUSG00000017002	59.79446	10.64301	2.4901	6.54E-13	1.04E-11	Slpi
ENSMUSG00000017311	49.70718	6.841932	2.861	4.18E-12	6.20E-11	Pyy
ENSMUSG00000017723	50.92252	0	7.3877	2.43E-14	4.26E-13	Wfdc2
ENSMUSG00000017868	61.86053	29.64837	1.0611	1.95E-06	1.54E-05	Sgk2
ENSMUSG00000017978	74.98614	22.95848	1.7076	1.63E-11	2.28E-10	Cadps2
ENSMUSG00000018417	63.31893	19.15741	1.7247	4.93E-10	5.94E-09	Myo1b
ENSMUSG00000018427	30.62643	11.85935	1.3688	0.000125	0.000732	Ypel2
ENSMUSG00000018740	34.39397	12.77161	1.4292	3.19E-05	0.000209	Slc25a35
ENSMUSG00000018819	45.93965	16.72472	1.4578	1.19E-06	9.66E-06	Lsp1
ENSMUSG00000019066	143.0449	43.78837	1.7078	1.35E-20	3.31E-19	Rab3d
ENSMUSG00000019832	28.68189	13.22774	1.1166	0.000868	0.004148	Rab32
ENSMUSG00000019852	26.61583	8.666448	1.6188	9.43E-05	0.000564	D10Bwg1379e
ENSMUSG00000019916	29.7757	7.298061	2.0286	3.80E-06	2.90E-05	P4ha1
ENSMUSG00000019997	34.27243	6.233761	2.4589	6.20E-08	6.04E-07	Ctgf
ENSMUSG00000020009	237.3548	96.54727	1.2977	2.98E-25	8.74E-24	Ifngr1
ENSMUSG00000020108	86.77489	35.73009	1.2801	4.61E-10	5.58E-09	Ddit4
ENSMUSG00000020121	27.22349	4.257202	2.6769	5.80E-07	4.90E-06	Srgap1
ENSMUSG00000020186	148.5139	59.90492	1.3099	1.54E-16	3.12E-15	Csrp2
ENSMUSG00000020218	16.77162	0	4.2004	6.89E-06	5.05E-05	Wif1
ENSMUSG00000020241	120.8043	56.71202	1.0909	1.48E-11	2.08E-10	Col6a2
ENSMUSG00000020263	173.7928	72.52448	1.2608	2.16E-18	4.75E-17	Appl2
ENSMUSG00000020277	145.1109	35.12192	2.0467	1.21E-24	3.46E-23	Pfkl
ENSMUSG00000020303	26.37276	2.888816	3.1905	1.54E-07	1.43E-06	Stc2
ENSMUSG00000020330	45.81811	20.67784	1.1478	1.94E-05	0.000132	Hmmr
ENSMUSG00000020376	61.25286	23.56666	1.378	5.18E-08	5.08E-07	Rnf130
ENSMUSG00000020527	52.74552	22.80644	1.2096	2.43E-06	1.90E-05	Myo19
ENSMUSG00000020573	18.59462	3.801074	2.2904	0.000111	0.000657	Pik3cg
ENSMUSG00000020581	845.1436	281.8876	1.5841	########	########	Agr2

ENSMUSG00000020788	867.5058	140.9438	2.6218	########	########	Atp2a3
ENSMUSG00000020841	389.8793	193.2466	1.0126	2.19E-31	7.92E-30	Cpd
ENSMUSG00000020865	226.4168	81.49502	1.4742	2.03E-27	6.51E-26	Abcc3
ENSMUSG00000020875	42.90131	1.216344	5.1404	1.89E-13	3.10E-12	Hoxb9
ENSMUSG00000020917	296.4201	117.3771	1.3365	4.61E-32	1.71E-30	Acly
ENSMUSG00000021062	123.8426	27.36773	2.178	1.90E-22	5.04E-21	Rab15
ENSMUSG00000021091	48.12725	3.496988	3.7827	9.43E-14	1.59E-12	Serpina3n
ENSMUSG00000021097	240.3932	51.08643	2.2344	5.76E-43	2.83E-41	Clmn
ENSMUSG00000021213	99.04976	31.47289	1.654	2.60E-14	4.52E-13	Akr1c13
ENSMUSG00000021306	42.77978	4.865374	3.1363	3.02E-11	4.11E-10	Gpr137b
ENSMUSG00000021339	41.92904	19.15741	1.13	5.07E-05	0.00032	Mrs2
ENSMUSG00000021356	16.52855	4.257202	1.957	0.000713	0.003497	Irf4
ENSMUSG00000021388	40.47064	11.70731	1.7895	4.04E-07	3.50E-06	Aspn
ENSMUSG00000021390	72.55547	30.25655	1.2618	1.56E-08	1.61E-07	Ogn
ENSMUSG00000021457	114.2414	23.11053	2.3055	7.43E-22	1.91E-20	Syk
ENSMUSG00000021556	936.5368	277.9345	1.7526	########	########	Golm1
ENSMUSG00000021559	17.25775	5.017417	1.7822	0.00096	0.004526	Dapk1
ENSMUSG00000021573	72.677	31.77697	1.1935	3.94E-08	3.93E-07	Tppp
ENSMUSG00000021608	19.44535	6.081718	1.6769	0.000677	0.003336	Lpcat1
ENSMUSG00000021614	18.35155	2.736773	2.7454	3.40E-05	0.000221	Vcan
ENSMUSG00000021662	45.57504	12.31548	1.8878	3.35E-08	3.36E-07	Arhgef28
ENSMUSG00000021702	245.7406	2.280644	6.7516	3.82E-66	2.86E-64	Thbs4
ENSMUSG00000021732	8.993475	0	4.3014	0.000944	0.004462	Fgf10
ENSMUSG00000021749	851.4634	157.0604	2.4386	########	########	Oit1
ENSMUSG00000021830	43.63051	17.02881	1.3574	5.15E-06	3.85E-05	Txndc16
ENSMUSG00000021868	105.0049	44.85267	1.2272	2.05E-11	2.83E-10	Ppif
ENSMUSG00000021950	61.61746	2.58473	4.5753	2.46E-18	5.40E-17	Anxa8
ENSMUSG00000022037	64.6558	20.5258	1.6553	7.44E-10	8.84E-09	Clu
ENSMUSG00000022043	31.9633	13.83591	1.208	0.000246	0.001335	Trim35
ENSMUSG00000022229	710.6061	0	11.19	########	########	Atp12a
ENSMUSG00000022261	52.62398	17.02881	1.6277	3.66E-08	3.67E-07	Sdc2
ENSMUSG00000022270	99.1713	44.85267	1.1447	3.48E-10	4.25E-09	Fam134b
ENSMUSG00000022272	103.9111	39.83525	1.3832	1.19E-12	1.85E-11	Myo10
ENSMUSG00000022286	35.36623	16.72472	1.0804	0.00028	0.001507	Grhl2
ENSMUSG00000022332	26.12969	8.666448	1.5922	0.000125	0.000733	Khdrbs3
ENSMUSG00000022358	75.35074	27.82386	1.4373	6.61E-10	7.89E-09	Fbxo32
ENSMUSG00000022419	182.5432	71.30814	1.3561	1.16E-20	2.85E-19	Deptor
ENSMUSG00000022440	50.67945	2.280644	4.4739	2.85E-15	5.33E-14	C1qtnf6
ENSMUSG00000022453	125.0579	58.99266	1.084	7.68E-12	1.12E-10	Naga
ENSMUSG00000022505	49.22105	10.49096	2.2301	5.23E-10	6.29E-09	Emp2
ENSMUSG00000022582	130.4054	0	9.7443	4.49E-26	1.39E-24	Ly6g
ENSMUSG00000022587	452.3475	147.7857	1.6139	3.83E-58	2.53E-56	Ly6e
ENSMUSG00000022629	61.13133	22.50236	1.4418	2.53E-08	2.57E-07	Kif21a
ENSMUSG00000022650	115.8214	1.216344	6.5732	1.88E-32	7.08E-31	Retnlb
ENSMUSG00000022665	34.39397	12.77161	1.4292	3.19E-05	0.000209	Ccdc80
ENSMUSG00000022680	473.4943	203.7375	1.2166	1.43E-45	7.42E-44	Pdxdc1
ENSMUSG00000022683	70.1248	5.473546	3.6794	4.32E-19	9.92E-18	Pla2g10

ENSMUSG00000022686	89.08402	4.257202	4.3872	1.62E-25	4.91E-24	B3gnt5
ENSMUSG00000022687	28.56036	6.233761	2.1958	2.62E-06	2.04E-05	Boc
ENSMUSG00000022696	39.74144	12.61956	1.655	1.39E-06	1.12E-05	Sidt1
ENSMUSG00000022747	130.77	26.45547	2.3054	8.77E-25	2.52E-23	St3gal6
ENSMUSG00000022844	57.36379	15.81247	1.8591	7.82E-10	9.25E-09	Pdia5
ENSMUSG00000022885	81.42741	8.058276	3.337	8.32E-21	2.07E-19	St6gal1
ENSMUSG00000022900	200.6517	5.625589	5.1565	5.36E-57	3.48E-55	Ildr1
ENSMUSG00000022947	38.64764	1.368386	4.8198	3.53E-12	5.27E-11	Cbr3
ENSMUSG00000023031	71.09707	6.689889	3.4097	1.37E-18	3.06E-17	Cela1
ENSMUSG00000023036	75.35074	30.56063	1.3019	4.64E-09	5.12E-08	Pcdhga12
ENSMUSG00000023078	21.38989	1.520429	3.8144	6.50E-07	5.46E-06	Cxcl13
ENSMUSG00000023088	47.03345	14.13999	1.7339	7.60E-08	7.35E-07	Abcc1
ENSMUSG00000023092	317.5669	46.82923	2.7616	6.31E-67	4.79E-65	Fhl1
ENSMUSG00000023122	18.35155	1.368386	3.7454	4.47E-06	3.38E-05	Sult1c2
ENSMUSG00000023247	504.85	233.3859	1.1131	3.12E-44	1.58E-42	Guca2a
ENSMUSG00000023393	46.30425	6.081718	2.9286	1.50E-11	2.11E-10	Slc17a9
ENSMUSG00000023805	36.46004	11.55526	1.6578	3.72E-06	2.85E-05	Synj2
ENSMUSG00000023827	70.97554	14.29204	2.3121	3.39E-14	5.86E-13	Agpat4
ENSMUSG00000023915	52.38092	20.22171	1.3731	5.01E-07	4.30E-06	Tnfrsf21
ENSMUSG00000023963	27.22349	8.818491	1.6262	7.54E-05	0.00046	Cyp39a1
ENSMUSG00000024039	40.95677	1.368386	4.9036	7.54E-13	1.19E-11	Cbs
ENSMUSG00000024215	44.35971	20.67784	1.1012	3.94E-05	0.000254	Spdef
ENSMUSG00000024222	205.27	31.1688	2.7193	2.19E-43	1.09E-41	Fkbp5
ENSMUSG00000024349	23.21289	8.362362	1.4729	0.000517	0.002621	Tmem173
ENSMUSG00000024353	32.20636	7.146018	2.1721	6.89E-07	5.76E-06	Mzb1
ENSMUSG00000024357	43.87358	16.42064	1.4178	2.89E-06	2.25E-05	Sil1
ENSMUSG00000024411	709.3908	23.7187	4.9025	########	########	Aqp4
ENSMUSG00000024501	30.26183	12.92365	1.2275	0.00032	0.001706	Dpysl3
ENSMUSG00000024548	25.64356	5.017417	2.3536	4.35E-06	3.29E-05	Setbp1
ENSMUSG00000024619	253.7618	121.4823	1.0627	5.04E-22	1.30E-20	Cdx1
ENSMUSG00000024659	232.372	16.87677	3.7833	3.22E-60	2.19E-58	Anxa1
ENSMUSG00000024665	165.5286	45.46084	1.8644	1.33E-25	4.05E-24	Fads2
ENSMUSG00000024697	109.7447	1.368386	6.3255	3.08E-31	1.10E-29	Gna14
ENSMUSG00000024727	97.22676	7.298061	3.7358	4.85E-26	1.50E-24	Trpm6
ENSMUSG00000024743	51.89478	21.5901	1.2652	1.67E-06	1.34E-05	Syt7
ENSMUSG00000024795	47.64111	23.7187	1.0062	4.92E-05	0.000311	Kif20b
ENSMUSG00000024817	84.22268	37.25052	1.1769	4.36E-09	4.82E-08	Uhrf2
ENSMUSG00000024972	26.37276	8.970533	1.5558	0.00014	0.000813	Lgals12
ENSMUSG00000025002	42.05057	8.666448	2.2786	6.71E-09	7.27E-08	Cyp2c55
ENSMUSG00000025150	27.70963	1.064301	4.7024	4.22E-09	4.68E-08	Cbr2
ENSMUSG00000025151	84.58728	37.40256	1.1773	4.02E-09	4.46E-08	Maged1
ENSMUSG00000025203	1412.826	135.4703	3.3825	0	0	Scd2
ENSMUSG00000025212	24.30669	8.058276	1.5928	0.000215	0.00118	Sfxn3
ENSMUSG00000025347	71.5832	29.19224	1.294	1.25E-08	1.31E-07	Mettl7b
ENSMUSG00000025348	22.96982	8.970533	1.3565	0.000944	0.004464	Itga7
ENSMUSG00000025366	57.97146	27.9759	1.0512	4.56E-06	3.44E-05	Esyt1
ENSMUSG00000025372	21.02529	7.146018	1.5569	0.000671	0.003306	Baiap2

ENSMUSG00000025515	4468.299	1182.286	1.9181	0	0	Muc2
ENSMUSG00000025647	196.3981	95.02684	1.0474	3.72E-17	7.71E-16	Shisa5
ENSMUSG00000025757	68.6664	33.75353	1.0246	8.61E-07	7.10E-06	Hspa4l
ENSMUSG00000025780	190.3214	37.70665	2.3355	1.05E-35	4.25E-34	Itih5
ENSMUSG00000025934	25.64356	5.473546	2.228	7.40E-06	5.39E-05	Gsta3
ENSMUSG00000025964	16.04242	4.105159	1.9664	0.000831	0.004	Adam23
ENSMUSG00000025969	87.62562	28.73612	1.6085	1.64E-12	2.52E-11	Nrp2
ENSMUSG00000025986	25.40049	7.602147	1.7404	7.55E-05	0.00046	Slc39a10
ENSMUSG00000025993	152.4029	22.80644	2.7404	7.64E-33	2.92E-31	Slc40a1
ENSMUSG00000026042	73.89234	24.02278	1.621	7.47E-11	9.79E-10	Col5a2
ENSMUSG00000026043	487.2276	158.8849	1.6166	1.37E-62	9.65E-61	Col3a1
ENSMUSG00000026072	26.25123	10.33892	1.3443	0.000435	0.002244	Il1r1
ENSMUSG00000026193	305.5351	83.47157	1.872	5.21E-46	2.74E-44	Fn1
ENSMUSG00000026303	97.46983	42.41998	1.2002	1.76E-10	2.22E-09	Mlph
ENSMUSG00000026322	16.16395	1.520429	3.4102	2.72E-05	0.00018	Htr4
ENSMUSG00000026335	202.9609	60.36105	1.7495	3.27E-29	1.10E-27	Pam
ENSMUSG00000026339	110.717	53.67116	1.0447	2.72E-10	3.38E-09	Ccdc93
ENSMUSG00000026365	43.99511	11.85935	1.8913	5.62E-08	5.50E-07	Cfh
ENSMUSG00000026380	522.5938	56.71202	3.204	########	########	Tfcp2l1
ENSMUSG00000026389	33.0571	10.94709	1.5944	1.58E-05	0.000109	Steap3
ENSMUSG00000026393	69.031	31.01676	1.1542	1.45E-07	1.35E-06	Nek7
ENSMUSG00000026418	20.05302	0	5.4582	4.85E-07	4.17E-06	Tnni1
ENSMUSG00000026435	35.12317	1.824515	4.2668	6.75E-11	8.89E-10	Slc45a3
ENSMUSG00000026473	321.5775	58.53653	2.4578	9.91E-62	6.83E-60	Glul
ENSMUSG00000026479	65.50653	27.51977	1.2512	8.85E-08	8.47E-07	Lamc2
ENSMUSG00000026480	32.3279	14.44408	1.1623	0.000304	0.001624	Ncf2
ENSMUSG00000026483	155.9274	49.41396	1.6579	1.09E-21	2.77E-20	Fam129a
ENSMUSG00000026509	99.1713	32.53719	1.6078	5.86E-14	9.96E-13	Capn2
ENSMUSG00000026519	131.2561	31.1688	2.0742	1.04E-22	2.80E-21	Tmem63a
ENSMUSG00000026520	27.58809	11.40322	1.2746	0.000455	0.002339	Pycr2
ENSMUSG00000026531	151.9168	1.672472	6.5052	3.42E-42	1.64E-40	Mptx1
ENSMUSG00000026574	60.88826	23.7187	1.3601	7.00E-08	6.78E-07	Dpt
ENSMUSG00000026600	60.03753	22.80644	1.3964	5.68E-08	5.55E-07	Soat1
ENSMUSG00000026605	73.64927	35.57805	1.0497	2.43E-07	2.19E-06	Cenpf
ENSMUSG00000026639	109.3801	30.1045	1.8613	1.97E-17	4.13E-16	Lamb3
ENSMUSG00000026656	19.56689	2.432687	3.0078	9.46E-06	6.76E-05	Fcgr2b
ENSMUSG00000026701	1170.489	193.7027	2.5952	########	########	Prdx6
ENSMUSG00000026712	28.07423	9.122576	1.6217	5.97E-05	0.000371	Mrc1
ENSMUSG00000026796	97.95596	39.37912	1.3147	1.87E-11	2.61E-10	Fam129b
ENSMUSG00000026797	32.81403	15.50838	1.0813	0.000463	0.002371	Stxbp1
ENSMUSG00000026811	778.0572	8.058276	6.5933	########	########	St6galnac6
ENSMUSG00000026822	13.24715	2.432687	2.4451	0.000787	0.003815	Lcn2
ENSMUSG00000026828	34.1509	7.75419	2.1389	3.86E-07	3.35E-06	Galnt5
ENSMUSG00000026837	67.08647	20.67784	1.6979	2.11E-10	2.65E-09	Col5a1
ENSMUSG00000026870	65.50653	3.496988	4.2275	5.69E-19	1.29E-17	Cutal
ENSMUSG00000026981	43.75204	2.58473	4.0813	5.06E-13	8.06E-12	Il1rn
ENSMUSG00000027006	219.2463	83.01545	1.4011	2.67E-25	7.86E-24	Dnajc10

ENSMUSG00000027068	46.06118	1.368386	5.073	2.57E-14	4.49E-13	Dhrs9
ENSMUSG00000027199	24.54976	2.888816	3.0872	5.53E-07	4.70E-06	Gatm
ENSMUSG00000027230	181.9356	21.74214	3.0649	4.20E-42	1.99E-40	Creb3l1
ENSMUSG00000027375	33.30017	3.192902	3.3826	1.88E-09	2.16E-08	Mal
ENSMUSG00000027397	213.8989	38.01074	2.4925	3.89E-42	1.85E-40	Slc20a1
ENSMUSG00000027401	259.1093	0	9.7349	3.39E-50	1.92E-48	Tgm3
ENSMUSG00000027556	613.2578	0	10.978	1.46E-95	1.63E-93	Car1
ENSMUSG00000027562	450.2814	32.2331	3.8042	########	########	Car2
ENSMUSG00000027605	96.86216	44.09245	1.1354	6.62E-10	7.89E-09	Acss2
ENSMUSG00000027684	85.43802	19.61354	2.123	1.24E-15	2.37E-14	Mecom
ENSMUSG00000027712	74.01387	29.0402	1.3497	3.22E-09	3.62E-08	Anxa5
ENSMUSG00000027750	176.8312	49.566	1.8349	7.71E-27	2.45E-25	Postn
ENSMUSG00000027870	175.8589	0	8.5908	3.10E-40	1.39E-38	Hao2
ENSMUSG00000027875	69.63867	7.75419	3.1668	1.79E-17	3.76E-16	Hmgcs2
ENSMUSG00000027876	116.9152	40.13934	1.5424	1.44E-15	2.73E-14	Reg4
ENSMUSG00000027938	24.79282	8.058276	1.6214	0.000162	0.000922	Creb3l4
ENSMUSG00000027953	49.22105	16.42064	1.5838	1.52E-07	1.41E-06	Slc50a1
ENSMUSG00000027995	29.65416	5.16946	2.5201	3.62E-07	3.15E-06	Tlr2
ENSMUSG00000028128	41.07831	18.54924	1.147	5.27E-05	0.000332	F3
ENSMUSG00000028173	103.1819	35.27396	1.5485	5.74E-14	9.80E-13	Wls
ENSMUSG00000028194	86.16722	20.98193	2.038	3.26E-15	6.07E-14	Ddah1
ENSMUSG00000028195	24.06362	8.818491	1.4483	0.000459	0.002353	Cyr61
ENSMUSG00000028199	14.46248	1.520429	3.2498	9.24E-05	0.000554	Cryz
ENSMUSG00000028211	153.1322	46.3731	1.7234	3.99E-22	1.04E-20	Trp53inp1
ENSMUSG00000028270	66.72187	32.68923	1.0293	1.16E-06	9.42E-06	Gbp2
ENSMUSG00000028293	140.2496	21.28601	2.72	3.44E-30	1.19E-28	Slc35a1
ENSMUSG00000028334	415.2798	83.9277	2.3069	5.34E-75	4.52E-73	Nans
ENSMUSG00000028358	17.13622	1.672472	3.357	1.71E-05	0.000117	Zfp618
ENSMUSG00000028369	42.77978	9.730748	2.1363	1.37E-08	1.43E-07	Svep1
ENSMUSG00000028392	147.4201	67.05094	1.1366	2.49E-14	4.36E-13	Bspry
ENSMUSG00000028479	415.2798	45.91697	3.177	4.66E-96	5.25E-94	Gne
ENSMUSG00000028518	97.95596	48.5017	1.0141	5.05E-09	5.54E-08	Prkaa2
ENSMUSG00000028527	83.12888	3.192902	4.7024	2.53E-24	7.14E-23	Ak4
ENSMUSG00000028538	46.42578	6.689889	2.7949	3.16E-11	4.29E-10	St3gal3
ENSMUSG00000028555	102.8173	17.94107	2.5187	2.74E-21	6.86E-20	Ttc39a
ENSMUSG00000028599	54.08239	10.49096	2.366	2.28E-11	3.13E-10	Tnfrsf1b
ENSMUSG00000028602	11.18108	0	3.8785	0.000304	0.001627	Tnfrsf8
ENSMUSG00000028617	111.6892	35.73009	1.6443	7.40E-16	1.43E-14	Lrrc42
ENSMUSG00000028645	98.4421	19.4615	2.3387	2.82E-19	6.58E-18	Slc2a1
ENSMUSG00000028698	50.43638	16.57268	1.6057	8.66E-08	8.30E-07	Pik3r3
ENSMUSG00000028699	1245.11	80.43072	3.9524	0	0	Tspan1
ENSMUSG00000028716	65.385	3.649031	4.1634	7.65E-19	1.72E-17	Pdzk1ip1
ENSMUSG00000028749	49.09951	12.31548	1.9952	3.90E-09	4.34E-08	Pla2g2f
ENSMUSG00000028780	115.0922	16.87677	2.7697	2.09E-25	6.28E-24	Sema3c
ENSMUSG00000028803	79.96901	38.92299	1.0388	8.80E-08	8.42E-07	Nipal3
ENSMUSG00000028860	36.21697	8.970533	2.0134	3.79E-07	3.29E-06	Sytl1
ENSMUSG00000028910	48.12725	23.87074	1.0116	4.28E-05	0.000273	Mecr

ENSMUSG00000028989	11.42414	0	4.6465	0.000165	0.000935	Angptl7
ENSMUSG00000029084	365.3296	121.6344	1.5866	1.66E-46	8.88E-45	Cd38
ENSMUSG00000029086	273.9364	132.2774	1.0503	2.26E-23	6.18E-22	Prom1
ENSMUSG00000029093	23.94209	6.233761	1.9414	4.96E-05	0.000313	Sorcs2
ENSMUSG00000029102	106.0987	22.80644	2.2179	9.22E-20	2.18E-18	Hgfac
ENSMUSG00000029154	24.06362	0	5.7213	3.74E-08	3.74E-07	Cwh43
ENSMUSG00000029246	30.26183	12.61956	1.2618	0.00026	0.001405	Ppat
ENSMUSG00000029247	88.71942	41.81181	1.0853	7.99E-09	8.61E-08	Paics
ENSMUSG00000029370	45.45351	20.37375	1.1577	1.92E-05	0.000131	Rassf6
ENSMUSG00000029371	10.45188	1.064301	3.2958	0.000842	0.004041	Cxcl5
ENSMUSG00000029388	65.26346	31.77697	1.0383	1.35E-06	1.10E-05	Eif2b1
ENSMUSG00000029426	65.26346	30.40859	1.1018	6.11E-07	5.15E-06	Scarb2
ENSMUSG00000029445	35.36623	10.18688	1.7957	2.09E-06	1.65E-05	Hpd
ENSMUSG00000029455	396.9283	191.8782	1.0487	4.48E-33	1.72E-31	Aldh2
ENSMUSG00000029482	115.5783	41.35568	1.4827	7.60E-15	1.38E-13	Aacs
ENSMUSG00000029484	153.1322	12.01139	3.6723	1.04E-39	4.56E-38	Anxa3
ENSMUSG00000029545	116.6721	49.71804	1.2306	1.49E-12	2.29E-11	Acads
ENSMUSG00000029569	67.9372	22.19827	1.6138	4.71E-10	5.69E-09	Tmem168
ENSMUSG00000029661	253.0326	93.65845	1.4338	1.28E-29	4.36E-28	Col1a2
ENSMUSG00000029675	20.78222	6.537846	1.6685	0.000458	0.002347	Eln
ENSMUSG00000029718	29.0465	9.426662	1.6235	4.40E-05	0.00028	Pcolce
ENSMUSG00000029762	55.90539	7.75419	2.8499	2.14E-13	3.48E-12	Akr1b8
ENSMUSG00000029781	61.00979	19.91763	1.615	3.53E-09	3.95E-08	Fkbp9
ENSMUSG00000029851	25.52203	8.362362	1.6098	0.000137	0.0008	Fam115c
ENSMUSG00000029865	228.4829	0	8.2315	6.02E-54	3.69E-52	Sval1
ENSMUSG00000029868	18.35155	0	6.9153	3.05E-06	2.36E-05	Trpv6
ENSMUSG00000029869	11.54568	0	3.6618	0.000297	0.001592	Ephb6
ENSMUSG00000030064	51.77325	18.24515	1.5047	1.58E-07	1.46E-06	Frmd4b
ENSMUSG00000030067	116.0644	45.3088	1.3571	1.05E-13	1.75E-12	Foxp1
ENSMUSG00000030200	61.73899	29.95246	1.0435	2.45E-06	1.91E-05	Bcl2l14
ENSMUSG00000030340	129.7977	8.818491	3.8796	8.34E-35	3.30E-33	Scnn1a
ENSMUSG00000030342	285.4821	68.87545	2.0513	6.04E-47	3.25E-45	Cd9
ENSMUSG00000030413	279.4054	121.1782	1.2052	2.52E-27	8.05E-26	Pglyrp1
ENSMUSG00000030471	115.2137	41.35568	1.4782	9.25E-15	1.67E-13	Zdhhc13
ENSMUSG00000030621	27.70963	10.49096	1.4012	0.00022	0.001205	Me3
ENSMUSG00000030653	77.41681	19.61354	1.9808	1.73E-13	2.85E-12	Pde2a
ENSMUSG00000030688	390.487	177.13	1.1405	1.82E-35	7.27E-34	Stard10
ENSMUSG00000030711	99.04976	3.192902	4.9552	6.61E-29	2.21E-27	Sult1a1
ENSMUSG00000030714	24.06362	4.865374	2.3062	1.03E-05	7.33E-05	Ccdc101
ENSMUSG00000030717	104.6403	41.20364	1.3446	2.14E-12	3.25E-11	Nupr1
ENSMUSG00000030759	244.6468	17.18085	3.8318	1.10E-63	8.05E-62	Far1
ENSMUSG00000030762	423.0579	0	9.7247	6.44E-81	5.99E-79	Aqp8
ENSMUSG00000030770	144.2602	68.41932	1.0762	2.43E-13	3.94E-12	Parva
ENSMUSG00000030800	40.59217	19.61354	1.0494	0.000127	0.000744	Prss8
ENSMUSG00000030866	69.51713	18.70128	1.8942	8.41E-12	1.22E-10	Ern2
ENSMUSG00000030867	79.96901	37.55461	1.0905	4.01E-08	3.99E-07	Plk1
ENSMUSG00000030935	88.84095	4.409245	4.3326	2.33E-25	6.97E-24	Acsm3

ENSMUSG00000030986	116.5506	53.06299	1.1352	1.26E-11	1.80E-10	Dhx32
ENSMUSG00000030987	103.9111	29.64837	1.8093	3.23E-16	6.43E-15	Stim1
ENSMUSG00000030990	58.57912	26.15139	1.1635	1.14E-06	9.33E-06	Pgap2
ENSMUSG00000031075	120.3181	15.05225	2.9988	5.22E-28	1.69E-26	Ano1
ENSMUSG00000031089	577.8916	8.362362	6.1107	########	########	Slc6a14
ENSMUSG00000031146	72.43394	26.30343	1.4614	1.01E-09	1.19E-08	Plp2
ENSMUSG00000031156	105.1264	47.89353	1.1342	1.28E-10	1.64E-09	Slc35a2
ENSMUSG00000031257	91.75776	1.368386	6.0673	1.13E-26	3.58E-25	Nox1
ENSMUSG00000031278	34.8801	9.578705	1.8645	1.58E-06	1.27E-05	Acsl4
ENSMUSG00000031375	133.2007	48.95783	1.444	1.87E-16	3.75E-15	Bgn
ENSMUSG00000031430	17.50082	0	5.8468	2.79E-06	2.17E-05	Vsig1
ENSMUSG00000031441	48.00571	15.20429	1.6587	1.09E-07	1.04E-06	Atp11a
ENSMUSG00000031442	67.08647	29.80042	1.1707	1.75E-07	1.61E-06	Mcf2l
ENSMUSG00000031451	227.7537	74.19695	1.618	3.30E-30	1.15E-28	Gas6
ENSMUSG00000031467	56.75612	27.36773	1.0523	5.67E-06	4.21E-05	Agpat5
ENSMUSG00000031502	206.3638	95.63501	1.1096	5.43E-19	1.24E-17	Col4a1
ENSMUSG00000031548	46.30425	18.54924	1.3198	3.71E-06	2.84E-05	Sfrp1
ENSMUSG00000031555	98.68516	41.81181	1.2389	6.54E-11	8.65E-10	Adam9
ENSMUSG00000031563	24.42822	10.18688	1.2618	0.001034	0.004825	Wwc2
ENSMUSG00000031608	209.4021	95.02684	1.1399	9.15E-20	2.17E-18	Galnt7
ENSMUSG00000031626	56.99919	16.87677	1.7559	2.58E-09	2.93E-08	Sorbs2
ENSMUSG00000031684	31.47716	15.35634	1.0355	0.000806	0.003891	Slc10a7
ENSMUSG00000031740	40.10604	15.20429	1.3993	8.92E-06	6.40E-05	Mmp2
ENSMUSG00000031762	179.2618	33.60149	2.4155	1.09E-34	4.27E-33	Mt2
ENSMUSG00000031765	228.726	97.30748	1.233	3.50E-23	9.51E-22	Mt1
ENSMUSG00000031775	68.42333	16.87677	2.0194	2.72E-12	4.09E-11	Pllp
ENSMUSG00000031785	37.91844	18.24515	1.0554	0.000203	0.001126	Gpr56
ENSMUSG00000031790	85.19495	26.30343	1.6955	8.43E-13	1.33E-11	Mmp15
ENSMUSG00000031824	66.8434	22.50236	1.5707	1.11E-09	1.30E-08	6430548M08Rik
ENSMUSG00000031891	319.1468	6.233761	5.678	2.10E-89	2.21E-87	Hsd11b2
ENSMUSG00000032026	48.97798	23.7187	1.0461	2.64E-05	0.000176	Rexo2
ENSMUSG00000032028	60.03753	0	6.3033	7.77E-18	1.66E-16	Nxpe2
ENSMUSG00000032053	18.83769	5.777632	1.7051	0.000742	0.00362	Pou2af1
ENSMUSG00000032068	331.0571	4.409245	6.2304	4.63E-91	4.92E-89	Plet1
ENSMUSG00000032085	818.5278	399.5688	1.0346	1.98E-65	1.46E-63	Tagln
ENSMUSG00000032109	35.85237	11.40322	1.6526	4.64E-06	3.49E-05	Nlrx1
ENSMUSG00000032122	38.64764	1.368386	4.8198	3.53E-12	5.27E-11	Slc37a2
ENSMUSG00000032232	51.16558	11.09913	2.2047	2.97E-10	3.66E-09	Cgnl1
ENSMUSG00000032261	203.3255	74.349	1.4514	2.06E-24	5.81E-23	Sh3bgrl2
ENSMUSG00000032278	25.40049	3.040859	3.0623	3.80E-07	3.30E-06	Paqr5
ENSMUSG00000032289	25.40049	8.362362	1.6029	0.000148	0.00085	Thsd4
ENSMUSG00000032290	49.34258	19.91763	1.3088	1.98E-06	1.57E-05	Ptpn9
ENSMUSG00000032332	77.41681	19.61354	1.9808	1.73E-13	2.85E-12	Col12a1
ENSMUSG00000032334	17.98695	4.409245	2.0283	0.000328	0.001744	Loxl1
ENSMUSG00000032369	138.4266	55.19159	1.3266	1.04E-15	1.99E-14	Plscr1
ENSMUSG00000032373	33.17863	15.20429	1.1258	0.000321	0.001711	Car12
ENSMUSG00000032440	105.0049	36.94643	1.5069	7.84E-14	1.33E-12	Tgfbr2

ENSMUSG00000032512	63.68353	31.47289	1.0168	2.36E-06	1.85E-05	Wdr48
ENSMUSG00000032602	162.2472	52.60686	1.6249	4.46E-22	1.16E-20	Slc25a20
ENSMUSG00000032718	64.2912	16.72472	1.9426	2.90E-11	3.96E-10	Mansc1
ENSMUSG00000032754	43.99511	7.146018	2.6221	2.97E-10	3.66E-09	Slc8b1
ENSMUSG00000032776	34.8801	7.146018	2.2872	1.23E-07	1.15E-06	Mctp2
ENSMUSG00000032816	23.21289	2.58473	3.1668	9.05E-07	7.45E-06	Igdcc4
ENSMUSG00000032849	25.88663	4.561288	2.5047	2.12E-06	1.67E-05	Abcc4
ENSMUSG00000032883	124.5718	25.08709	2.312	9.65E-24	2.65E-22	Acsl3
ENSMUSG00000033059	222.2847	71.61222	1.6341	8.30E-30	2.85E-28	Pygb
ENSMUSG00000033066	24.30669	8.666448	1.4878	0.000355	0.001872	Gas7
ENSMUSG00000033105	71.46167	33.2974	1.1018	1.80E-07	1.65E-06	Lss
ENSMUSG00000033192	24.91436	5.017417	2.312	7.05E-06	5.15E-05	Lpcat2
ENSMUSG00000033213	305.049	92.89824	1.7153	3.12E-42	1.50E-40	AA467197
ENSMUSG00000033287	30.62643	6.841932	2.1623	1.36E-06	1.10E-05	Kctd17
ENSMUSG00000033579	129.7977	0	8.1526	1.15E-31	4.22E-30	Fa2h
ENSMUSG00000033590	236.3826	42.72407	2.468	5.23E-46	2.74E-44	Myo5c
ENSMUSG00000033715	222.8924	42.57202	2.3884	2.59E-42	1.26E-40	Akr1c14
ENSMUSG00000033952	38.40457	16.72472	1.1993	6.23E-05	0.000387	Aspm
ENSMUSG00000033998	90.78549	38.16278	1.2503	3.08E-10	3.79E-09	Kcnk1
ENSMUSG00000034006	33.17863	9.730748	1.7696	5.06E-06	3.80E-05	Pqlc1
ENSMUSG00000034112	71.9478	10.64301	2.757	2.02E-16	4.05E-15	Atp2c2
ENSMUSG00000034205	50.19332	18.3972	1.448	4.20E-07	3.63E-06	Loxl2
ENSMUSG00000034353	37.31077	10.33892	1.8515	7.47E-07	6.22E-06	Ramp1
ENSMUSG00000034570	71.5832	30.86472	1.2137	3.77E-08	3.77E-07	Inpp5j
ENSMUSG00000034573	18.35155	4.561288	2.0084	0.000305	0.001629	Ptpn13
ENSMUSG00000034586	69.031	26.75956	1.3672	8.60E-09	9.25E-08	Hid1
ENSMUSG00000034659	45.21044	17.63698	1.3581	3.45E-06	2.65E-05	Tmem109
ENSMUSG00000034981	235.5318	27.51977	3.0974	2.27E-54	1.42E-52	Parm1
ENSMUSG00000035069	34.75857	17.3329	1.0039	0.000534	0.002693	Oma1
ENSMUSG00000035112	22.84829	6.385803	1.8391	0.000114	0.000669	Wnk4
ENSMUSG00000035184	21.02529	5.017417	2.0671	8.89E-05	0.000535	Fam124a
ENSMUSG00000035284	79.72594	16.42064	2.2795	1.40E-15	2.66E-14	Vps13c
ENSMUSG00000035451	78.14601	20.82988	1.9075	3.65E-13	5.85E-12	Foxa1
ENSMUSG00000035547	141.4649	34.05762	2.0544	3.82E-24	1.07E-22	Capn5
ENSMUSG00000035781	105.3695	32.38515	1.7021	1.56E-15	2.96E-14	R3hdm4
ENSMUSG00000035847	48.61338	21.74214	1.1609	9.57E-06	6.82E-05	Ids
ENSMUSG00000035930	44.48124	19.91763	1.1592	2.33E-05	0.000156	Chst4
ENSMUSG00000036078	69.88174	27.06364	1.3686	6.84E-09	7.42E-08	Sigmar1
ENSMUSG00000036123	398.1436	62.64169	2.6681	3.09E-81	2.90E-79	Slc9a3
ENSMUSG00000036273	22.48369	4.257202	2.4009	1.43E-05	9.92E-05	Lrrk2
ENSMUSG00000036298	35.73084	5.017417	2.8322	4.81E-09	5.29E-08	Slc2a13
ENSMUSG00000036334	21.63295	7.602147	1.5088	0.000689	0.003386	Igsf10
ENSMUSG00000036553	28.43883	10.94709	1.3773	0.000208	0.001146	Sh3tc1
ENSMUSG00000036585	28.92496	12.77161	1.1794	0.000574	0.002874	Fgf1
ENSMUSG00000036620	183.5155	83.9277	1.1287	2.40E-17	5.01E-16	Mgat4b
ENSMUSG00000036777	46.54731	22.04623	1.0782	3.13E-05	0.000206	Anln
ENSMUSG00000036805	62.10359	20.67784	1.5866	3.57E-09	3.98E-08	Noxa1

ENSMUSG00000036896	50.07178	22.19827	1.1736	6.22E-06	4.59E-05	C1qc
ENSMUSG00000036905	50.07178	20.67784	1.2759	2.30E-06	1.80E-05	C1qb
ENSMUSG00000037012	200.7733	25.08709	3.0006	1.28E-45	6.70E-44	Hk1
ENSMUSG00000037025	21.26835	2.888816	2.8802	5.49E-06	4.09E-05	Foxa2
ENSMUSG00000037033	524.1738	243.5728	1.1057	1.53E-45	7.85E-44	Clca4
ENSMUSG00000037049	25.64356	10.79505	1.2482	0.000831	0.003999	Smpd1
ENSMUSG00000037095	25.64356	8.970533	1.5153	0.000213	0.001169	Lrg1
ENSMUSG00000037362	140.0065	0	7.2618	4.91E-37	2.02E-35	Nov
ENSMUSG00000037370	33.66477	16.57268	1.0224	0.000578	0.002891	Enpp1
ENSMUSG00000037379	35.12317	7.298061	2.2668	1.25E-07	1.17E-06	Spon2
ENSMUSG00000037419	275.2733	40.44342	2.7669	2.43E-58	1.61E-56	Endod1
ENSMUSG00000037709	41.19984	16.87677	1.2876	1.65E-05	0.000114	Fam13a
ENSMUSG00000037860	52.25938	9.426662	2.4709	2.11E-11	2.90E-10	Aim2
ENSMUSG00000037953	13.24715	1.368386	3.2751	0.000176	0.000991	A4gnt
ENSMUSG00000038203	47.64111	0	6.5741	2.81E-14	4.87E-13	Hoxa13
ENSMUSG00000038210	36.7031	0	6.1978	1.55E-11	2.17E-10	Hoxa11
ENSMUSG00000038227	52.01632	0	7.4183	1.40E-14	2.50E-13	Hoxa9
ENSMUSG00000038236	38.03997	3.801074	3.323	1.70E-10	2.16E-09	Hoxa7
ENSMUSG00000038331	110.96	17.02881	2.704	4.02E-24	1.12E-22	Satb2
ENSMUSG00000038351	27.58809	11.40322	1.2746	0.000455	0.002339	Sgsm2
ENSMUSG00000038412	159.3304	55.34363	1.5255	1.96E-20	4.78E-19	Higd1a
ENSMUSG00000038463	17.37928	4.561288	1.9299	0.000567	0.002844	Olfml2b
ENSMUSG00000038521	63.9266	28.43203	1.1689	3.49E-07	3.05E-06	C1s1
ENSMUSG00000038732	163.4625	20.82988	2.9722	3.21E-37	1.33E-35	Mboat1
ENSMUSG00000038736	37.55384	17.3329	1.1154	0.00014	0.000811	Nudcd1
ENSMUSG00000038756	26.61583	0	6.4517	1.16E-08	1.22E-07	Ttll6
ENSMUSG00000038843	54.56852	10.94709	2.3175	2.82E-11	3.86E-10	Gcnt1
ENSMUSG00000039005	30.1403	1.520429	4.3091	1.40E-09	1.62E-08	Tlr4
ENSMUSG00000039109	14.09788	1.672472	3.0754	0.000151	0.000867	F13a1
ENSMUSG00000039236	17.50082	4.561288	1.9399	0.000525	0.002654	Isg20
ENSMUSG00000039238	11.42414	0	4.514	0.000174	0.000981	Zfp750
ENSMUSG00000039286	54.32545	24.93504	1.1235	4.26E-06	3.23E-05	Fndc3b
ENSMUSG00000039323	25.15742	0	5.0484	1.81E-08	1.86E-07	Igfbp2
ENSMUSG00000039450	43.02284	20.67784	1.057	7.47E-05	0.000457	Dcxr
ENSMUSG00000039680	42.05057	12.16344	1.7896	2.40E-07	2.16E-06	Mrps6
ENSMUSG00000039701	67.9372	19.91763	1.7702	6.60E-11	8.71E-10	Usp53
ENSMUSG00000039774	107.9217	28.12794	1.9399	7.19E-18	1.55E-16	Galnt12
ENSMUSG00000039813	37.67537	9.426662	1.9988	2.44E-07	2.19E-06	Tbc1d2
ENSMUSG00000039865	57.60686	26.75956	1.1062	2.65E-06	2.07E-05	Slc44a3
ENSMUSG00000039943	33.54323	12.92365	1.376	5.67E-05	0.000354	Plcb4
ENSMUSG00000040121	63.07586	13.07569	2.2702	1.35E-12	2.08E-11	Rep15
ENSMUSG00000040363	41.32137	19.76558	1.0639	9.82E-05	0.000585	Bcor
ENSMUSG00000040412	134.2945	29.19224	2.2017	1.96E-24	5.54E-23	5330417C22Rik
ENSMUSG00000040488	98.92823	42.41998	1.2216	8.59E-11	1.12E-09	Ltbp4
ENSMUSG00000040549	98.32056	48.04557	1.0331	3.33E-09	3.73E-08	Ckap5
ENSMUSG00000040562	65.87113	13.83591	2.2512	5.30E-13	8.42E-12	Gstm2
ENSMUSG00000040605	35.12317	10.18688	1.7857	2.41E-06	1.89E-05	Bace2

ENSMUSG00000040690	32.81403	10.64301	1.6244	1.40E-05	9.75E-05	Col16a1
ENSMUSG00000040711	22.11909	7.298061	1.5997	0.000403	0.002099	Sh3pxd2b
ENSMUSG00000040728	232.8581	104.4535	1.1566	4.05E-22	1.06E-20	Esrp1
ENSMUSG00000040964	46.66885	16.11655	1.5339	4.95E-07	4.25E-06	Arhgef10l
ENSMUSG00000040969	60.28059	26.30343	1.1964	5.45E-07	4.64E-06	Arhgef38
ENSMUSG00000040990	31.35563	8.058276	1.9602	3.08E-06	2.38E-05	Sh3kbp1
ENSMUSG00000041058	74.13541	36.64235	1.0167	3.54E-07	3.09E-06	Wwp1
ENSMUSG00000041193	78.51061	18.70128	2.0698	3.52E-14	6.07E-13	Pla2g5
ENSMUSG00000041293	13.73328	0	4.9121	3.30E-05	0.000215	Gpr110
ENSMUSG00000041351	71.09707	20.67784	1.7817	2.06E-11	2.85E-10	Rap1gap
ENSMUSG00000041439	245.4976	79.82254	1.6208	1.74E-32	6.57E-31	Mfsd6
ENSMUSG00000041577	55.29772	23.11053	1.2587	8.22E-07	6.80E-06	Prelp
ENSMUSG00000041650	52.01632	23.7187	1.1329	6.19E-06	4.58E-05	Pcca
ENSMUSG00000041654	112.0538	55.19159	1.0217	3.45E-10	4.23E-09	Slc39a11
ENSMUSG00000041736	92.60849	40.74751	1.1844	6.57E-10	7.85E-09	Tspo
ENSMUSG00000042082	31.47716	10.18688	1.6276	2.06E-05	0.000139	Arsb
ENSMUSG00000042254	22.11909	3.192902	2.7924	4.63E-06	3.49E-05	Cilp
ENSMUSG00000042303	78.51061	17.94107	2.1296	1.59E-14	2.81E-13	Sgsm3
ENSMUSG00000042306	94.06689	13.53182	2.7973	3.29E-21	8.23E-20	S100a14
ENSMUSG00000042312	28.43883	10.94709	1.3773	0.000208	0.001146	S100a13
ENSMUSG00000042436	18.35155	5.777632	1.6674	0.000995	0.004664	Mfap4
ENSMUSG00000042460	122.1411	43.94041	1.4749	1.59E-15	3.00E-14	C1galt1
ENSMUSG00000042659	87.99022	36.94643	1.2519	5.61E-10	6.73E-09	Arrdc4
ENSMUSG00000042766	20.29609	1.520429	3.7387	1.41E-06	1.14E-05	Trim46
ENSMUSG00000042784	22.36216	0	5.6155	1.10E-07	1.04E-06	Muc1
ENSMUSG00000043003	105.1264	25.54321	2.0411	3.02E-18	6.59E-17	Rasef
ENSMUSG00000043029	19.32382	5.625589	1.7803	0.000479	0.002444	Trpv3
ENSMUSG00000043391	28.31729	10.03483	1.4967	0.00011	0.000652	2510009E07Rik
ENSMUSG00000043461	58.45759	2.128601	4.7794	1.26E-17	2.68E-16	Sptssb
ENSMUSG00000043705	53.59625	21.28601	1.3322	5.65E-07	4.80E-06	Capn13
ENSMUSG00000044080	82.64275	19.15741	2.109	4.37E-15	8.07E-14	S100a1
ENSMUSG00000044162	58.09299	0	6.2558	2.41E-17	5.03E-16	Tnip3
ENSMUSG00000044229	72.06934	2.736773	4.7188	2.58E-21	6.48E-20	Nxpe4
ENSMUSG00000044340	32.93557	14.59612	1.1741	0.000247	0.00134	Phlpp1
ENSMUSG00000044350	23.09136	8.818491	1.3887	0.000788	0.003816	Lacc1
ENSMUSG00000044461	34.27243	10.49096	1.7079	5.26E-06	3.93E-05	Shisa2
ENSMUSG00000044646	14.82708	1.824515	3.0226	0.000112	0.000662	Zbtb7c
ENSMUSG00000044674	34.8801	6.993975	2.3182	1.03E-07	9.78E-07	Fzd1
ENSMUSG00000044827	61.98206	9.426662	2.717	3.29E-14	5.69E-13	Tlr1
ENSMUSG00000044986	135.6313	10.03483	3.7566	9.22E-36	3.74E-34	Tst
ENSMUSG00000045019	36.94617	11.09913	1.735	1.87E-06	1.50E-05	Acer1
ENSMUSG00000045679	38.40457	19.00537	1.0149	0.000251	0.001359	Pqlc3
ENSMUSG00000045680	21.14682	6.841932	1.628	0.000482	0.002456	Tcf21
ENSMUSG00000045930	19.56689	5.473546	1.8379	0.000356	0.001877	Clec14a
ENSMUSG00000045932	36.46004	12.16344	1.5838	6.22E-06	4.59E-05	Ifit2
ENSMUSG00000046027	34.63703	9.578705	1.8544	1.83E-06	1.46E-05	Stard5
ENSMUSG00000046314	28.56036	10.03483	1.509	9.62E-05	0.000575	Stxbp6

ENSMUSG00000046410	53.23165	21.28601	1.3224	6.83E-07	5.72E-06	Kcnk6
ENSMUSG00000046519	320.7268	103.2372	1.6354	2.75E-42	1.33E-40	Golph3l
ENSMUSG00000046589	10.93801	0	5.1687	0.000203	0.001127	Lrrc8e
ENSMUSG00000046733	143.1664	52.15073	1.4569	1.01E-17	2.16E-16	Gprc5a
ENSMUSG00000046794	34.63703	5.16946	2.7442	1.24E-08	1.31E-07	Ppp1r3b
ENSMUSG00000046841	81.42741	26.30343	1.6303	7.14E-12	1.04E-10	Ckap4
ENSMUSG00000046971	20.66069	0	5.3688	3.25E-07	2.86E-06	Pla2g4f
ENSMUSG00000047139	360.7113	145.5051	1.3098	7.28E-38	3.08E-36	Cd24a
ENSMUSG00000047407	39.37684	19.4615	1.0167	0.000206	0.001141	Tgif1
ENSMUSG00000047517	6985.014	2690.4	1.3764	0	0	Dmbt1
ENSMUSG00000047694	137.9405	64.31416	1.1008	4.23E-13	6.74E-12	Yipf6
ENSMUSG00000047821	82.52121	37.55461	1.1358	1.19E-08	1.26E-07	Trim16
ENSMUSG00000048058	19.68842	2.736773	2.8468	1.34E-05	9.32E-05	Ldlrad3
ENSMUSG00000048327	34.63703	15.81247	1.1313	0.000228	0.001248	Ckap2l
ENSMUSG00000048337	9.115009	0	4.3207	0.000864	0.004129	Npy4r
ENSMUSG00000048373	66.4788	32.53719	1.0308	1.19E-06	9.66E-06	Fgfbp1
ENSMUSG00000048537	74.13541	36.64235	1.0167	3.54E-07	3.09E-06	Phldb1
ENSMUSG00000048612	55.90539	17.94107	1.6397	1.22E-08	1.28E-07	Myof
ENSMUSG00000048779	18.95922	3.344945	2.5028	4.98E-05	0.000315	P2ry6
ENSMUSG00000049047	39.01224	16.57268	1.2351	4.14E-05	0.000265	Armcx3
ENSMUSG00000049307	108.4078	40.74751	1.4117	2.19E-13	3.55E-12	Fut4
ENSMUSG00000049521	28.56036	11.09913	1.3636	0.000218	0.001195	Cdc42ep1
ENSMUSG00000049604	159.4519	0	9.0344	1.10E-34	4.29E-33	Hoxb13
ENSMUSG00000049709	9.601143	0	5.9807	0.000533	0.002693	Nlrp10
ENSMUSG00000049892	17.01468	3.953116	2.1057	0.000376	0.001971	Rasd1
ENSMUSG00000049922	219.4894	88.03286	1.318	7.73E-24	2.14E-22	Slc35c1
ENSMUSG00000050359	13.36868	2.128601	2.6509	0.000486	0.002474	Sprr1a
ENSMUSG00000050520	76.20147	0	7.9692	1.27E-19	3.00E-18	Cldn8
ENSMUSG00000051314	17.37928	5.017417	1.7924	0.00089	0.004245	Ffar2
ENSMUSG00000051439	70.1248	11.40322	2.6205	1.83E-15	3.43E-14	Cd14
ENSMUSG00000052212	563.0645	13.07569	5.4283	########	########	Cd177
ENSMUSG00000052485	73.16314	18.09311	2.0157	5.08E-13	8.07E-12	Tmem171
ENSMUSG00000052516	39.37684	4.409245	3.1587	1.65E-10	2.10E-09	Robo2
ENSMUSG00000052560	25.76509	5.929675	2.1194	1.13E-05	7.99E-05	Cpne8
ENSMUSG00000052698	43.02284	17.02881	1.3371	7.09E-06	5.18E-05	Tln2
ENSMUSG00000052819	42.53671	0	8.1281	1.92E-11	2.67E-10	Best2
ENSMUSG00000053398	38.03997	9.426662	2.0127	1.94E-07	1.77E-06	Phgdh
ENSMUSG00000053846	45.33198	9.274619	2.2892	1.61E-09	1.86E-08	Lipg
ENSMUSG00000054161	267.4951	119.3537	1.1643	2.45E-25	7.27E-24	Fam83e
ENSMUSG00000054169	127.6101	28.58407	2.1585	6.64E-23	1.80E-21	Ceacam10
ENSMUSG00000054619	61.37439	20.5258	1.5802	4.74E-09	5.22E-08	Mettl7a1
ENSMUSG00000054675	33.0571	6.689889	2.3049	2.37E-07	2.14E-06	Tmem119
ENSMUSG00000055116	34.02937	7.450104	2.1914	2.99E-07	2.65E-06	Arntl
ENSMUSG00000055210	38.76917	0	7.9943	1.08E-10	1.39E-09	Foxd2
ENSMUSG00000055373	14.09788	0	4.8174	2.67E-05	0.000177	Fut9
ENSMUSG00000055415	316.1085	139.7275	1.1778	5.43E-30	1.87E-28	Atp10b
ENSMUSG00000055748	214.385	47.58944	2.1715	1.49E-37	6.26E-36	Gsdmc4

ENSMUSG00000055799	21.26835	3.192902	2.7358	8.35E-06	6.02E-05	Tcf7l1
ENSMUSG00000055827	34.75857	12.46752	1.4792	2.06E-05	0.00014	Gsdmc3
ENSMUSG00000055978	257.7725	13.68386	4.2356	8.93E-70	6.97E-68	Fut2
ENSMUSG00000056220	57.60686	19.30945	1.5769	1.45E-08	1.51E-07	Pla2g4a
ENSMUSG00000056293	83.00735	33.60149	1.3047	7.40E-10	8.80E-09	Gsdmc2
ENSMUSG00000056429	1084.321	321.1147	1.7556	########	########	Tgoln1
ENSMUSG00000056553	21.99755	5.625589	1.9673	9.06E-05	0.000544	Ptprn2
ENSMUSG00000056673	38.1615	7.450104	2.3568	2.05E-08	2.10E-07	Kdm5d
ENSMUSG00000056724	58.09299	26.60751	1.1265	1.92E-06	1.53E-05	Nbeal2
ENSMUSG00000056737	77.65988	21.89418	1.8266	1.32E-12	2.05E-11	Capg
ENSMUSG00000057193	33.7863	16.26859	1.0543	0.000456	0.002342	Slc44a2
ENSMUSG00000057315	14.46248	3.192902	2.1794	0.000863	0.004126	Arhgap24
ENSMUSG00000057465	31.9633	3.953116	3.0154	1.46E-08	1.52E-07	Saa2
ENSMUSG00000058135	105.8556	25.99934	2.0256	3.07E-18	6.69E-17	Gstm1
ENSMUSG00000058230	140.3711	36.03418	1.9618	5.32E-23	1.45E-21	Arhgap35
ENSMUSG00000058297	52.01632	11.55526	2.1704	2.85E-10	3.52E-09	Spock2
ENSMUSG00000058454	105.7341	49.41396	1.0975	2.37E-10	2.96E-09	Dhcr7
ENSMUSG00000058799	137.8189	68.87545	1.0007	5.79E-12	8.50E-11	Nap1l1
ENSMUSG00000059743	169.4176	81.7991	1.0504	4.74E-15	8.73E-14	Fdps
ENSMUSG00000060012	107.5571	34.96988	1.6209	3.99E-15	7.41E-14	Kif13b
ENSMUSG00000060519	40.34911	17.63698	1.1939	4.24E-05	0.000271	Tor3a
ENSMUSG00000060600	54.32545	15.66042	1.7945	4.13E-09	4.58E-08	Eno3
ENSMUSG00000061119	43.99511	20.06967	1.1323	3.25E-05	0.000212	Prcp
ENSMUSG00000061132	92.24389	24.02278	1.941	1.67E-15	3.13E-14	Blnk
ENSMUSG00000061353	70.73247	19.61354	1.8505	9.63E-12	1.39E-10	Cxcl12
ENSMUSG00000061762	28.80343	13.37978	1.1062	0.000899	0.004276	Tac1
ENSMUSG00000062098	36.58157	16.87677	1.1161	0.00017	0.000962	Btbd3
ENSMUSG00000062127	79.84748	31.77697	1.3293	1.07E-09	1.25E-08	Cttnbp2nl
ENSMUSG00000062210	53.59625	15.66042	1.775	6.34E-09	6.89E-08	Tnfaip8
ENSMUSG00000062410	52.13785	3.953116	3.7213	1.15E-14	2.07E-13	Hsd3b3
ENSMUSG00000062995	28.43883	10.94709	1.3773	0.000208	0.001146	Ica1
ENSMUSG00000063730	20.66069	6.993975	1.5627	0.00073	0.003569	Hsd3b2
ENSMUSG00000063903	267.9813	31.01676	3.111	8.61E-62	5.97E-60	Klk1
ENSMUSG00000064254	263.8491	20.37375	3.6949	2.42E-67	1.88E-65	Ethe1
ENSMUSG00000066090	34.27243	0	6.099	6.60E-11	8.71E-10	Insl5
ENSMUSG00000067006	73.52774	8.514405	3.1103	3.70E-18	8.05E-17	Serpinb5
ENSMUSG00000067149	1598.53	493.5314	1.6955	########	########	Igj
ENSMUSG00000068040	202.4747	62.94578	1.6856	3.99E-28	1.30E-26	Tm9sf4
ENSMUSG00000068086	49.34258	0	6.6248	1.08E-14	1.95E-13	Cyp2d9
ENSMUSG00000068196	13.97635	1.368386	3.3524	0.000104	0.000616	Col8a1
ENSMUSG00000068220	74.37847	36.33826	1.0334	2.67E-07	2.39E-06	Lgals1
ENSMUSG00000068457	23.82056	0	4.9697	4.44E-08	4.40E-07	Uty
ENSMUSG00000068551	30.74796	8.818491	1.8019	9.34E-06	6.68E-05	Zfp467
ENSMUSG00000068735	156.6566	50.93438	1.6209	2.54E-21	6.40E-20	Trp53i11
ENSMUSG00000068874	284.0237	21.74214	3.7074	1.79E-72	1.45E-70	Selenbp1
ENSMUSG00000068893	13.97635	2.432687	2.5224	0.000475	0.002427	Sprr2a2
ENSMUSG00000069045	43.75204	0	6.4513	2.56E-13	4.14E-12	Ddx3y

ENSMUSG00000069049	43.02284	0	6.427	3.89E-13	6.22E-12	Eif2s3y
ENSMUSG00000069094	57.84992	19.61354	1.5605	1.62E-08	1.67E-07	Pde7a
ENSMUSG00000069114	24.91436	3.496988	2.8328	1.02E-06	8.33E-06	Zbtb10
ENSMUSG00000069456	71.09707	11.40322	2.6403	9.57E-16	1.84E-14	Rdh16
ENSMUSG00000069516	169.6607	48.65374	1.802	2.20E-25	6.58E-24	Lyz2
ENSMUSG00000069808	48.24878	8.058276	2.5819	5.51E-11	7.37E-10	Fam57a
ENSMUSG00000071551	70.36787	28.27999	1.3151	1.25E-08	1.31E-07	Akr1c19
ENSMUSG00000071604	18.59462	3.953116	2.2338	0.000133	0.000776	Fam189a2
ENSMUSG00000071711	68.90947	31.62493	1.1236	2.23E-07	2.02E-06	Mpst
ENSMUSG00000071984	19.80995	5.777632	1.7777	0.00041	0.002134	Fndc1
ENSMUSG00000072244	13.85481	2.280644	2.6029	0.000423	0.002194	Trim6
ENSMUSG00000072999	15.43475	0	4.6656	1.18E-05	8.29E-05	Gm15401
ENSMUSG00000073043	49.70718	14.44408	1.783	2.08E-08	2.13E-07	Atoh1
ENSMUSG00000073418	86.41028	36.79439	1.2317	1.11E-09	1.30E-08	C4b
ENSMUSG00000073598	18.47308	5.929675	1.6394	0.001059	0.004924	1700066B19Rik
ENSMUSG00000073608	14.94861	1.216344	3.6194	4.06E-05	0.000261	Gm6086
ENSMUSG00000073758	29.28956	12.77161	1.1974	0.000477	0.002433	Sh3d21
ENSMUSG00000073987	201.1379	9.426662	4.4153	1.43E-55	9.14E-54	Ggh
ENSMUSG00000074115	1124.549	110.0791	3.3527	########	########	Saa1
ENSMUSG00000074207	626.9911	197.6558	1.6655	1.92E-82	1.86E-80	Adh1
ENSMUSG00000074272	2086.851	855.2415	1.2869	########	########	Ceacam1
ENSMUSG00000074445	242.5808	31.32085	2.9533	3.85E-54	2.37E-52	Sprr2a3
ENSMUSG00000074457	159.938	44.09245	1.8589	9.99E-25	2.87E-23	S100a16
ENSMUSG00000074656	568.1689	259.3853	1.1312	2.16E-50	1.25E-48	Eif2s2
ENSMUSG00000074715	179.2618	53.8232	1.7358	8.45E-26	2.60E-24	Ccl28
ENSMUSG00000074743	33.0571	10.94709	1.5944	1.58E-05	0.000109	Thbd
ENSMUSG00000074794	72.79854	23.11053	1.6554	6.46E-11	8.57E-10	Arrdc3
ENSMUSG00000074892	909.7994	35.42601	4.6827	########	########	B3galt5
ENSMUSG00000075420	86.04568	31.47289	1.451	3.32E-11	4.49E-10	Smim6
ENSMUSG00000075602	607.1811	38.01074	3.9976	########	########	Ly6a
ENSMUSG00000076441	25.76509	7.146018	1.8502	3.94E-05	0.000254	Ass1
ENSMUSG00000076548	21.02529	7.602147	1.4676	0.000974	0.004579	Igkv4-69
ENSMUSG00000076612	110.5954	4.105159	4.7517	7.07E-32	2.61E-30	Ighg2c
ENSMUSG00000076937	71.46167	10.94709	2.7066	4.17E-16	8.19E-15	Iglc2
ENSMUSG00000078664	13.36868	1.976558	2.7578	0.000394	0.002059	Sprr2a1
ENSMUSG00000078670	47.51958	13.98795	1.7643	4.99E-08	4.90E-07	Fam174b
ENSMUSG00000078706	64.2912	1.216344	5.724	2.39E-19	5.59E-18	Gm53
ENSMUSG00000079018	46.18271	7.75419	2.5743	1.49E-10	1.90E-09	Ly6c1
ENSMUSG00000079037	86.89642	31.47289	1.4652	2.10E-11	2.90E-10	Prnp
ENSMUSG00000079173	45.57504	10.94709	2.0577	8.54E-09	9.19E-08	Zan
ENSMUSG00000079339	357.4299	130.6049	1.4525	1.12E-41	5.24E-40	Gm14446
ENSMUSG00000079442	30.26183	5.777632	2.3889	5.08E-07	4.35E-06	St6galnac4
ENSMUSG00000079445	297.8785	29.49633	3.3361	1.34E-71	1.07E-69	B3gnt7
ENSMUSG00000079470	23.21289	8.514405	1.4469	0.000583	0.002912	Utp14b
ENSMUSG00000084174	81.54895	0	7.067	1.34E-22	3.60E-21	Sycn
ENSMUSG00000089774	45.21044	7.450104	2.6013	1.94E-10	2.45E-09	Slc5a3
ENSMUSG00000090213	51.40865	8.514405	2.594	1.20E-11	1.71E-10	Tmem189

ENSMUSG00000090264	18.10848	3.040859	2.5741	6.03E-05	0.000375	Eif4ebp3
ENSMUSG00000094559	166.987	0	10.101	2.92E-31	1.05E-29	Cyp2d34
ENSMUSG00000094806	36.7031	0	6.1978	1.55E-11	2.17E-10	Cyp2d10
ENSMUSG00000095026	34.39397	16.42064	1.0666	0.000373	0.001958	Gm3336
ENSMUSG00000096001	183.0294	4.713331	5.2792	3.34E-52	1.96E-50	2610528A11Rik
ENSMUSG00000096466	53.23165	25.69526	1.0508	1.12E-05	7.92E-05	Ighv1-83
ENSMUSG00000096852	25.27896	0	5.6599	1.67E-08	1.72E-07	Cyp2d12
ENSMUSG00000098188	54.44699	26.60751	1.033	1.08E-05	7.61E-05	Sowahc

Supplementary Table S5: List of upregulated genes in tumors of *Ifngr1*$^{-/-}$ApcMin mice (compared to normal tissues)

Gene_id	read count_ Ifngr_T	read count_ Ifngr_N	log2. Fold_ change.	*p* value	*q* value	Gene Name
ENSMUSG00000000058	21.80827	6.041367	1.8519	5.82E-05	0.000246	Cav2
ENSMUSG00000000085	33.35383	12.54746	1.4105	1.19E-05	5.74E-05	Scmh1
ENSMUSG00000000142	80.93551	39.81106	1.0236	4.82E-09	3.77E-08	Axin2
ENSMUSG00000000204	82.21835	25.09491	1.7121	5.05E-14	6.38E-13	Slfn4
ENSMUSG00000000216	10.84582	0	4.4391	0.000168	0.000641	Scnn1g
ENSMUSG00000000253	19.59246	2.013789	3.2823	2.07E-06	1.14E-05	Gmpr
ENSMUSG00000000320	28.57233	2.47851	3.5271	5.06E-09	3.94E-08	Alox12
ENSMUSG00000000555	61.92616	19.36336	1.6772	9.57E-11	9.00E-10	Itga5
ENSMUSG00000000628	366.309	129.3472	1.5018	1.51E-50	6.59E-49	Hk2
ENSMUSG00000000782	19.8257	5.731554	1.7904	0.00016	0.000615	Tcf7
ENSMUSG00000000805	373.7728	141.2751	1.4037	1.89E-48	7.71E-47	Car4
ENSMUSG00000000938	51.4302	1.239255	5.3751	1.28E-16	1.94E-15	Hoxa10
ENSMUSG00000000983	121.9864	21.99677	2.4714	6.29E-27	1.53E-25	Wfdc18
ENSMUSG00000001025	607.5994	124.8549	2.2829	########	########	S100a6
ENSMUSG00000001119	117.5547	50.18982	1.2279	1.42E-14	1.86E-13	Col6a1
ENSMUSG00000001128	19.24259	5.266833	1.8693	0.00015	0.000577	Cfp
ENSMUSG00000001349	406.4269	53.59777	2.9228	1.55E-96	1.46E-94	Cnn1
ENSMUSG00000001473	30.5549	12.85727	1.2488	7.67E-05	0.000317	Tubb6
ENSMUSG00000001506	263.4486	77.45343	1.7661	1.44E-42	5.23E-41	Col1a1
ENSMUSG00000001630	77.43685	33.9246	1.1907	7.58E-10	6.48E-09	Stk38l
ENSMUSG00000001761	33.82031	9.139504	1.8877	4.44E-07	2.71E-06	Smo
ENSMUSG00000001763	10.7292	0	3.529	0.000341	0.001209	Tspan33
ENSMUSG00000001768	71.72239	35.00895	1.0347	3.06E-08	2.17E-07	Rin2
ENSMUSG00000001819	80.00253	0	7.322	1.28E-22	2.59E-21	Hoxd13
ENSMUSG00000001823	9.21312	0	4.2037	0.000577	0.001915	Hoxd12
ENSMUSG00000001911	49.09776	20.91243	1.2313	6.39E-07	3.82E-06	Nfix
ENSMUSG00000002058	29.50531	4.182485	2.8185	2.89E-08	2.06E-07	Unc119
ENSMUSG00000002489	22.85787	7.590436	1.5904	0.000125	0.000491	Tiam1
ENSMUSG00000002602	45.71573	18.74373	1.2863	9.30E-07	5.44E-06	Axl
ENSMUSG00000002980	25.1903	8.210063	1.6174	4.91E-05	0.000211	Bcam
ENSMUSG00000003352	29.73855	12.23764	1.281	7.84E-05	0.000324	Cacnb3
ENSMUSG00000003518	45.13262	18.27901	1.304	9.27E-07	5.43E-06	Dusp3
ENSMUSG00000003541	38.0187	15.49069	1.2953	7.16E-06	3.60E-05	Ier3
ENSMUSG00000003617	57.14467	16.11031	1.8266	1.00E-10	9.40E-10	Cp
ENSMUSG00000004044	87.11646	32.06572	1.4419	8.56E-13	9.71E-12	Ptrf

ENSMUSG00000004098	18.65948	3.407951	2.4529	2.79E-05	0.000125	Col5a3
ENSMUSG00000004105	32.53747	7.90025	2.0421	2.94E-07	1.85E-06	Angptl2
ENSMUSG00000004356	54.1125	24.16547	1.163	3.65E-07	2.27E-06	Utp20
ENSMUSG00000004558	84.08429	22.46149	1.9044	1.32E-15	1.88E-14	Ndrg2
ENSMUSG00000004562	22.04151	8.674784	1.3453	0.000501	0.001696	Arhgef40
ENSMUSG00000004665	43.61654	20.60261	1.082	1.03E-05	4.98E-05	Cnn2
ENSMUSG00000004951	24.37395	2.788323	3.1279	1.80E-07	1.16E-06	Hspb1
ENSMUSG00000004988	8.046902	0	5.699	0.001099	0.003371	Fxyd4
ENSMUSG00000005034	189.627	24.01056	2.9814	1.32E-46	5.18E-45	Prkacb
ENSMUSG00000005355	26.2399	0	5.7137	3.80E-09	3.01E-08	Casp14
ENSMUSG00000005397	60.06021	29.2774	1.0366	3.93E-07	2.42E-06	Nid1
ENSMUSG00000005503	11.07907	0	6.1603	0.000147	0.000568	Evx1
ENSMUSG00000005672	25.42355	8.829691	1.5257	7.24E-05	0.000301	Kit
ENSMUSG00000005803	372.6065	137.5573	1.4376	2.25E-49	9.44E-48	Sqrdl
ENSMUSG00000005823	70.08968	29.12249	1.2671	1.63E-09	1.35E-08	Gpr108
ENSMUSG00000005873	59.59372	17.19466	1.7932	5.80E-11	5.57E-10	Reep5
ENSMUSG00000005958	33.47045	16.11031	1.0549	0.000134	0.000527	Ephb3
ENSMUSG00000006205	60.64332	12.39255	2.2909	2.00E-13	2.40E-12	Htra1
ENSMUSG00000006411	26.35652	1.549069	4.0887	6.82E-09	5.20E-08	Pvrl4
ENSMUSG00000006777	44.54952	21.84187	1.0283	1.35E-05	6.44E-05	Krt23
ENSMUSG00000007613	64.72508	30.51665	1.0847	7.42E-08	5.06E-07	Tgfbr1
ENSMUSG00000007655	89.33227	25.40472	1.8141	7.61E-16	1.11E-14	Cav1
ENSMUSG00000008090	32.77072	10.68857	1.6163	3.68E-06	1.95E-05	Fgfrl1
ENSMUSG00000008393	144.611	70.0179	1.0464	2.65E-15	3.70E-14	Carhsp1
ENSMUSG00000008398	29.15544	10.68857	1.4477	3.40E-05	0.00015	Elk3
ENSMUSG00000009356	19.8257	0	4.4149	3.69E-07	2.29E-06	Lpo
ENSMUSG00000009418	40.58438	15.49069	1.3895	1.62E-06	9.14E-06	Nav1
ENSMUSG00000009628	13.64475	0	6.4608	2.93E-05	0.000131	Tex15
ENSMUSG00000009739	13.06164	2.94323	2.1499	0.000896	0.002825	Pou6f1
ENSMUSG00000010080	22.508	8.36497	1.428	0.000296	0.001075	Epn3
ENSMUSG00000010175	72.18887	13.16708	2.4548	1.67E-16	2.52E-15	Prox1
ENSMUSG00000011832	18.54286	2.788323	2.7334	1.36E-05	6.47E-05	Evi5l
ENSMUSG00000012350	330.3895	100.8444	1.712	1.70E-51	7.64E-50	Ehf
ENSMUSG00000012428	74.28807	3.872671	4.2617	1.19E-22	2.41E-21	Steap4
ENSMUSG00000013236	33.70369	10.99839	1.6156	2.69E-06	1.46E-05	Ptprs
ENSMUSG00000013275	35.45302	15.49069	1.1945	3.05E-05	0.000136	Slc41a1
ENSMUSG00000013338	18.193	2.168696	3.0685	7.41E-06	3.72E-05	Fer1l4
ENSMUSG00000013523	60.99318	22.30659	1.4512	1.94E-09	1.60E-08	Bcas1
ENSMUSG00000013653	131.7826	2.788323	5.5626	5.95E-40	2.03E-38	1810065E05Rik
ENSMUSG00000013698	53.17953	21.22224	1.3253	7.98E-08	5.42E-07	Pea15a
ENSMUSG00000013766	11.31231	2.013789	2.4899	0.001033	0.003203	Ly6g6e
ENSMUSG00000014444	34.52004	15.49069	1.156	5.11E-05	0.000218	Piezo1
ENSMUSG00000015189	103.4435	22.6164	2.1934	4.24E-21	8.15E-20	Casd1
ENSMUSG00000015468	19.8257	8.055156	1.2994	0.001168	0.003555	Notch4
ENSMUSG00000015568	20.64205	4.182485	2.3032	1.73E-05	8.11E-05	Lpl
ENSMUSG00000015880	27.17287	13.01218	1.0623	0.000557	0.001859	Ncapg
ENSMUSG00000016024	27.75598	5.576647	2.3153	5.93E-07	3.56E-06	Lbp

ENSMUSG00000016028	97.4958	19.67317	2.3091	8.90E-21	1.66E-19	Celsr1
ENSMUSG00000016382	68.45698	18.58882	1.8808	7.40E-13	8.45E-12	Pls3
ENSMUSG00000016494	23.32435	9.294411	1.3274	0.000375	0.001313	Cd34
ENSMUSG00000016541	29.03882	13.94162	1.0586	0.000368	0.001291	Atxn10
ENSMUSG00000016918	32.18761	8.519877	1.9176	7.03E-07	4.18E-06	Sulf1
ENSMUSG00000017002	110.3242	15.02597	2.8762	3.32E-27	8.19E-26	Slpi
ENSMUSG00000017144	61.80954	22.46149	1.4604	1.36E-09	1.14E-08	Rnd3
ENSMUSG00000017204	9.796229	1.239255	2.9828	0.00112	0.003429	Gsdma
ENSMUSG00000017311	68.22373	0	7.0922	6.83E-20	1.21E-18	Pyy
ENSMUSG00000017466	83.73443	19.05354	2.1358	5.03E-17	7.69E-16	Timp2
ENSMUSG00000017493	181.6967	78.38287	1.2129	1.91E-21	3.73E-20	Igfbp4
ENSMUSG00000017723	60.41008	1.239255	5.6072	3.47E-19	5.94E-18	Wfdc2
ENSMUSG00000017817	22.97449	9.604225	1.2583	0.000579	0.001919	Jph2
ENSMUSG00000017978	63.32562	21.68696	1.546	2.94E-10	2.63E-09	Cadps2
ENSMUSG00000018217	168.2852	73.58076	1.1935	1.07E-19	1.87E-18	Pmp22
ENSMUSG00000018398	43.03343	18.27901	1.2353	3.03E-06	1.64E-05	8-Sep
ENSMUSG00000018417	67.87387	21.22224	1.6773	1.22E-11	1.26E-10	Myo1b
ENSMUSG00000018593	297.2689	90.3107	1.7188	1.13E-46	4.45E-45	Sparc
ENSMUSG00000018740	22.15814	8.829691	1.3274	0.000526	0.001767	Slc25a35
ENSMUSG00000018819	39.6514	16.72994	1.2449	6.86E-06	3.47E-05	Lsp1
ENSMUSG00000018830	639.5538	308.1097	1.0536	1.71E-62	9.93E-61	Myh11
ENSMUSG00000019066	166.186	52.35852	1.6663	3.97E-26	9.35E-25	Rab3d
ENSMUSG00000019539	16.32705	3.872671	2.0759	0.000254	0.000936	Rcn3
ENSMUSG00000019699	18.07637	5.731554	1.6571	0.000505	0.001708	Akt3
ENSMUSG00000019726	18.7761	7.435529	1.3364	0.001364	0.004077	Lyst
ENSMUSG00000019772	32.77072	15.95541	1.0384	0.000177	0.000674	Vip
ENSMUSG00000019832	35.45302	11.15329	1.6684	1.03E-06	5.97E-06	Rab32
ENSMUSG00000019846	34.52004	9.139504	1.9172	2.80E-07	1.77E-06	Lama4
ENSMUSG00000019853	16.91016	2.94323	2.5224	5.53E-05	0.000235	Hebp2
ENSMUSG00000019888	358.0288	118.6587	1.5933	3.59E-52	1.67E-50	Mgat4c
ENSMUSG00000019920	108.3416	46.78187	1.2116	2.15E-13	2.56E-12	Lims1
ENSMUSG00000019929	228.3454	80.08684	1.5116	2.39E-32	7.09E-31	Dcn
ENSMUSG00000019942	55.86183	27.72833	1.0105	1.34E-06	7.68E-06	Cdk1
ENSMUSG00000019975	26.47314	10.84348	1.2877	0.000188	0.000709	Ikbip
ENSMUSG00000019987	38.36856	5.886461	2.7045	4.55E-10	3.98E-09	Arg1
ENSMUSG00000019997	30.90477	6.351181	2.2827	1.62E-07	1.05E-06	Ctgf
ENSMUSG00000020015	23.20773	11.15329	1.0571	0.00146	0.004325	Cdk17
ENSMUSG00000020029	351.3814	118.9685	1.5625	2.44E-50	1.06E-48	Nudt4
ENSMUSG00000020044	36.15275	10.68857	1.758	4.31E-07	2.63E-06	Timp3
ENSMUSG00000020053	31.72112	9.139504	1.7953	1.75E-06	9.80E-06	Igf1
ENSMUSG00000020120	23.20773	8.674784	1.4197	0.000248	0.000917	Plek
ENSMUSG00000020121	30.20504	6.196274	2.2853	2.20E-07	1.41E-06	Srgap1
ENSMUSG00000020142	25.89003	8.984598	1.5269	6.18E-05	0.00026	Slc1a4
ENSMUSG00000020218	82.80146	8.829691	3.2292	2.69E-22	5.39E-21	Wif1
ENSMUSG00000020241	87.3497	41.82485	1.0624	6.05E-10	5.20E-09	Col6a2
ENSMUSG00000020263	113.3564	49.88001	1.1843	1.12E-13	1.38E-12	Appl2
ENSMUSG00000020275	19.94232	3.098137	2.6864	7.35E-06	3.69E-05	Rel

ENSMUSG00000020303	54.1125	6.660995	3.0222	1.46E-14	1.89E-13	Stc2
ENSMUSG00000020376	57.61115	23.23603	1.31	2.76E-08	1.97E-07	Rnf130
ENSMUSG00000020388	15.97718	2.47851	2.6885	5.97E-05	0.000252	Pdlim4
ENSMUSG00000020462	20.52543	8.519877	1.2685	0.001093	0.003355	Ccdc104
ENSMUSG00000020473	22.62462	9.914039	1.1903	0.000882	0.002796	Aebp1
ENSMUSG00000020532	66.00792	31.91081	1.0486	8.96E-08	6.03E-07	Acaca
ENSMUSG00000020581	854.3711	345.5972	1.3058	########	2.65E-99	Agr2
ENSMUSG00000020646	15.62732	2.323603	2.7496	6.27E-05	0.000264	Mboat2
ENSMUSG00000020674	39.06829	14.40634	1.4393	1.71E-06	9.58E-06	Pxdn
ENSMUSG00000020788	631.5069	125.0098	2.3368	########	########	Atp2a3
ENSMUSG00000020844	19.94232	8.055156	1.3078	0.00109	0.003348	Nxn
ENSMUSG00000020875	31.6045	0	5.9821	1.21E-10	1.13E-09	Hoxb9
ENSMUSG00000020916	21.45841	0	7.114	3.12E-07	1.96E-06	Krt36
ENSMUSG00000020917	221.2315	81.94573	1.4328	6.37E-30	1.73E-28	Acly
ENSMUSG00000021062	119.8872	20.2928	2.5626	3.39E-27	8.32E-26	Rab15
ENSMUSG00000021091	64.60846	23.23603	1.4754	4.77E-10	4.17E-09	Serpina3n
ENSMUSG00000021097	177.615	65.21579	1.4455	1.55E-24	3.37E-23	Clmn
ENSMUSG00000021108	15.27745	4.957019	1.6239	0.001538	0.004526	Prkch
ENSMUSG00000021306	71.83901	4.182485	4.1023	1.04E-21	2.04E-20	Gpr137b
ENSMUSG00000021388	34.17018	5.42174	2.6559	5.09E-09	3.96E-08	Aspn
ENSMUSG00000021390	52.71304	5.886461	3.1627	1.36E-14	1.78E-13	Ogn
ENSMUSG00000021457	88.16606	23.23603	1.9239	2.00E-16	3.00E-15	Syk
ENSMUSG00000021493	45.83235	19.98298	1.1976	2.06E-06	1.14E-05	Pdlim7
ENSMUSG00000021508	29.27206	10.84348	1.4327	3.58E-05	0.000158	Cxcl14
ENSMUSG00000021556	982.0719	368.0587	1.4159	########	########	Golm1
ENSMUSG00000021559	18.7761	5.576647	1.7514	0.000276	0.00101	Dapk1
ENSMUSG00000021573	18.7761	7.125715	1.3978	0.00107	0.00329	Tppp
ENSMUSG00000021614	20.99192	3.717765	2.4973	7.63E-06	3.81E-05	Vcan
ENSMUSG00000021662	35.56964	11.61801	1.6143	1.44E-06	8.17E-06	Arhgef28
ENSMUSG00000021701	28.10585	8.36497	1.7484	8.76E-06	4.31E-05	Plk2
ENSMUSG00000021702	167.7021	2.788323	5.9104	6.90E-50	2.93E-48	Thbs4
ENSMUSG00000021720	10.02947	0	4.4317	0.000297	0.001077	Rnf180
ENSMUSG00000021749	762.7064	282.5501	1.4326	7.79E-99	7.62E-97	Oit1
ENSMUSG00000021830	58.07764	13.94162	2.0586	6.23E-12	6.64E-11	Txndc16
ENSMUSG00000021950	240.5907	27.1087	3.1498	1.15E-60	6.49E-59	Anxa8
ENSMUSG00000021978	47.58168	23.70075	1.0055	8.59E-06	4.24E-05	Extl3
ENSMUSG00000021998	80.23578	37.02274	1.1158	1.26E-09	1.06E-08	Lcp1
ENSMUSG00000022032	9.562985	0	3.363	0.000841	0.002678	Scara5
ENSMUSG00000022033	35.56964	16.88485	1.0749	7.14E-05	0.000298	Pbk
ENSMUSG00000022043	34.52004	12.23764	1.4961	4.62E-06	2.40E-05	Trim35
ENSMUSG00000022096	25.77341	8.674784	1.571	5.11E-05	0.000218	Hr
ENSMUSG00000022146	31.95436	10.84348	1.5592	6.97E-06	3.52E-05	Osmr
ENSMUSG00000022150	19.47584	5.266833	1.8867	0.000128	0.000503	Dab2
ENSMUSG00000022229	313.0128	0	9.9806	5.28E-60	2.93E-58	Atp12a
ENSMUSG00000022261	40.58438	14.71615	1.4635	8.83E-07	5.19E-06	Sdc2
ENSMUSG00000022263	50.73047	14.09652	1.8475	9.10E-10	7.73E-09	Trio
ENSMUSG00000022272	78.13658	39.03653	1.0012	1.26E-08	9.33E-08	Myo10

ENSMUSG00000022297	20.52543	6.041367	1.7645	0.000136	0.000532	Fzd6
ENSMUSG00000022332	16.91016	3.407951	2.3109	9.84E-05	0.000398	Khdrbs3
ENSMUSG00000022358	58.54413	15.33578	1.9326	1.90E-11	1.91E-10	Fbxo32
ENSMUSG00000022376	26.93963	7.590436	1.8275	8.95E-06	4.39E-05	Adcy8
ENSMUSG00000022404	56.91142	20.60261	1.4659	5.68E-09	4.39E-08	Slc25a17
ENSMUSG00000022425	23.20773	2.633417	3.1396	3.43E-07	2.14E-06	Enpp2
ENSMUSG00000022440	48.16479	5.42174	3.1512	1.95E-13	2.34E-12	C1qtnf6
ENSMUSG00000022505	50.14736	4.337392	3.5313	9.49E-15	1.26E-13	Emp2
ENSMUSG00000022582	203.505	3.717765	5.7745	2.02E-60	1.14E-58	Ly6g
ENSMUSG00000022587	413.6574	118.9685	1.7979	7.20E-67	4.47E-65	Ly6e
ENSMUSG00000022594	18.42624	2.94323	2.6463	1.82E-05	8.48E-05	Lynx1
ENSMUSG00000022629	57.02804	24.47528	1.2203	9.13E-08	6.14E-07	Kif21a
ENSMUSG00000022641	25.77341	10.99839	1.2286	0.000314	0.001131	Bbx
ENSMUSG00000022650	210.7355	37.02274	2.509	8.49E-46	3.27E-44	Retnlb
ENSMUSG00000022651	20.29219	1.394162	3.8635	4.89E-07	2.96E-06	Retnlg
ENSMUSG00000022665	35.56964	2.788323	3.6732	4.50E-11	4.37E-10	Ccdc80
ENSMUSG00000022683	72.53874	5.731554	3.6618	5.50E-21	1.05E-19	Pla2g10
ENSMUSG00000022686	221.3481	5.111926	5.4363	5.08E-66	3.07E-64	B3gnt5
ENSMUSG00000022696	38.83505	14.87106	1.3849	2.81E-06	1.53E-05	Sidt1
ENSMUSG00000022797	890.4072	285.8031	1.6394	########	########	Tfrc
ENSMUSG00000022816	119.0708	51.42908	1.2112	1.44E-14	1.87E-13	Fstl1
ENSMUSG00000022883	34.52004	4.802113	2.8457	1.74E-09	1.44E-08	Robo1
ENSMUSG00000022885	67.05752	21.06733	1.6704	1.77E-11	1.79E-10	St6gal1
ENSMUSG00000022900	174.816	4.492299	5.2822	1.38E-52	6.57E-51	Ildr1
ENSMUSG00000022947	32.42085	4.027578	3.0089	2.76E-09	2.22E-08	Cbr3
ENSMUSG00000022949	59.36048	23.70075	1.3246	1.43E-08	1.05E-07	Clic6
ENSMUSG00000022995	20.05894	5.886461	1.7688	0.00016	0.000613	Enah
ENSMUSG00000023021	33.35383	14.25143	1.2267	4.19E-05	0.000182	Cers5
ENSMUSG00000023031	110.5574	19.36336	2.5134	7.35E-25	1.62E-23	Cela1
ENSMUSG00000023034	13.87799	3.253044	2.0929	0.000713	0.002311	Nr4a1
ENSMUSG00000023036	71.13928	23.23603	1.6143	9.35E-12	9.79E-11	Pcdhga12
ENSMUSG00000023055	27.8726	12.54746	1.1515	0.00028	0.001023	Calcoco1
ENSMUSG00000023078	33.00396	4.647206	2.8282	4.24E-09	3.34E-08	Cxcl13
ENSMUSG00000023088	41.16748	18.27901	1.1713	8.53E-06	4.21E-05	Abcc1
ENSMUSG00000023092	290.2716	28.50286	3.3482	2.13E-75	1.49E-73	Fhl1
ENSMUSG00000023122	27.63936	3.407951	3.0197	3.90E-08	2.74E-07	Sult1c2
ENSMUSG00000023247	107.6419	45.2328	1.2508	1.10E-13	1.36E-12	Guca2a
ENSMUSG00000023277	22.62462	10.53367	1.1029	0.001344	0.004026	Twf2
ENSMUSG00000023393	41.40073	4.957019	3.0621	1.42E-11	1.45E-10	Slc17a9
ENSMUSG00000023805	42.45032	11.92783	1.8314	2.40E-08	1.73E-07	Synj2
ENSMUSG00000023827	34.86991	10.68857	1.7059	9.78E-07	5.70E-06	Agpat4
ENSMUSG00000023885	29.97179	8.055156	1.8956	1.92E-06	1.06E-05	Thbs2
ENSMUSG00000023915	54.81223	12.54746	2.1271	1.26E-11	1.29E-10	Tnfrsf21
ENSMUSG00000023963	28.9222	10.53367	1.4572	3.45E-05	0.000152	Cyp39a1
ENSMUSG00000023972	39.6514	12.70236	1.6423	2.90E-07	1.83E-06	Ptk7
ENSMUSG00000024039	39.06829	0	5.3935	5.52E-13	6.38E-12	Cbs
ENSMUSG00000024164	208.2865	74.35529	1.4861	3.32E-29	8.85E-28	C3

ENSMUSG00000024215	47.6983	19.20845	1.3122	4.21E-07	2.57E-06	Spdef
ENSMUSG00000024232	11.89542	2.013789	2.5624	0.000668	0.002183	Bambi
ENSMUSG00000024247	34.05356	15.8005	1.1078	8.07E-05	0.000333	Pkdcc
ENSMUSG00000024349	50.14736	16.26522	1.6244	9.50E-09	7.10E-08	Tmem173
ENSMUSG00000024401	10.49596	1.549069	2.7604	0.001021	0.003173	Tnf
ENSMUSG00000024411	490.1613	29.74212	4.0427	########	########	Aqp4
ENSMUSG00000024501	45.24925	10.06895	2.168	5.51E-10	4.78E-09	Dpysl3
ENSMUSG00000024511	72.42212	29.58721	1.2915	6.14E-10	5.28E-09	Rab27b
ENSMUSG00000024529	27.52274	4.182485	2.7182	1.23E-07	8.15E-07	Lox
ENSMUSG00000024538	18.7761	5.731554	1.7119	0.00032	0.00115	Ppic
ENSMUSG00000024597	565.6156	195.6474	1.5316	1.80E-78	1.34E-76	Slc12a2
ENSMUSG00000024659	458.5568	46.16224	3.3123	########	########	Anxa1
ENSMUSG00000024664	21.57503	7.90025	1.4494	0.00036	0.001268	Fads3
ENSMUSG00000024665	132.4823	58.24498	1.1856	9.57E-16	1.38E-14	Fads2
ENSMUSG00000024673	12.94502	0	4.7999	3.45E-05	0.000152	Ms4a1
ENSMUSG00000024697	119.8872	1.394162	6.4261	2.62E-35	8.26E-34	Gna14
ENSMUSG00000024727	24.02408	1.394162	4.107	3.07E-08	2.18E-07	Trpm6
ENSMUSG00000024730	516.6344	176.5938	1.5487	1.43E-72	9.69E-71	Ms4a8a
ENSMUSG00000024743	46.18222	8.055156	2.5194	2.73E-11	2.71E-10	Syt7
ENSMUSG00000024766	15.16083	4.492299	1.7548	0.001073	0.0033	Lipo1
ENSMUSG00000024774	14.4611	1.858882	2.9597	7.85E-05	0.000324	Ankrd22
ENSMUSG00000024924	35.80288	9.294411	1.9456	1.40E-07	9.20E-07	Vldlr
ENSMUSG00000024940	24.02408	8.984598	1.419	0.000194	0.00073	Ltbp3
ENSMUSG00000025002	21.69165	2.94323	2.8817	1.64E-06	9.24E-06	Cyp2c55
ENSMUSG00000025040	31.72112	14.71615	1.108	0.000142	0.000551	Fundc1
ENSMUSG00000025150	34.98653	0	6.8193	3.84E-11	3.75E-10	Cbr2
ENSMUSG00000025151	85.25051	29.89702	1.5117	4.65E-13	5.43E-12	Maged1
ENSMUSG00000025153	181.2302	84.42424	1.1021	1.17E-19	2.03E-18	Fasn
ENSMUSG00000025203	929.2423	207.5752	2.1624	########	########	Scd2
ENSMUSG00000025245	30.20504	9.449318	1.6765	6.18E-06	3.15E-05	Lztfl1
ENSMUSG00000025268	17.95975	3.872671	2.2134	8.06E-05	0.000332	Maged2
ENSMUSG00000025321	8.979876	0	4.2723	0.000658	0.002153	Itgb8
ENSMUSG00000025348	18.65948	6.196274	1.5904	0.000527	0.00177	Itga7
ENSMUSG00000025352	12.47853	2.94323	2.084	0.001358	0.004065	Gdf11
ENSMUSG00000025366	60.99318	28.81268	1.0819	1.82E-07	1.17E-06	Esyt1
ENSMUSG00000025372	24.49057	6.970809	1.8128	2.45E-05	0.000112	Baiap2
ENSMUSG00000025473	16.44367	4.492299	1.872	0.000452	0.001548	Adam8
ENSMUSG00000025491	41.98384	17.50447	1.2621	3.18E-06	1.71E-05	Ifitm1
ENSMUSG00000025492	139.5963	61.49802	1.1826	1.79E-16	2.69E-15	Ifitm3
ENSMUSG00000025515	1780.931	821.9358	1.1155	########	########	Muc2
ENSMUSG00000025555	49.44763	23.23603	1.0895	2.44E-06	1.33E-05	Farp1
ENSMUSG00000025666	13.52813	3.098137	2.1265	0.000767	0.002465	Tmem47
ENSMUSG00000025743	28.9222	8.674784	1.7373	6.91E-06	3.49E-05	Sdc3
ENSMUSG00000025757	68.10711	28.19305	1.2725	2.56E-09	2.07E-08	Hspa4l
ENSMUSG00000025780	132.0158	21.22224	2.6371	2.16E-30	5.99E-29	Itih5
ENSMUSG00000025867	18.30962	3.562858	2.3615	4.35E-05	0.000189	Cplx2
ENSMUSG00000025934	34.17018	14.09652	1.2774	2.36E-05	0.000108	Gsta3

ENSMUSG00000025969	47.81493	11.3082	2.0801	3.71E-10	3.29E-09	Nrp2
ENSMUSG00000025986	26.58976	11.3082	1.2335	0.000245	0.000907	Slc39a10
ENSMUSG00000025993	158.0225	69.55318	1.1839	1.84E-18	3.06E-17	Slc40a1
ENSMUSG00000025997	12.82839	0	4.0499	5.43E-05	0.000231	Ikzf2
ENSMUSG00000026037	23.5576	11.15329	1.0787	0.001206	0.003653	Orc2
ENSMUSG00000026042	103.91	21.06733	2.3023	5.46E-22	1.08E-20	Col5a2
ENSMUSG00000026043	583.3421	117.1096	2.3165	########	########	Col3a1
ENSMUSG00000026072	36.85248	10.22385	1.8498	1.76E-07	1.14E-06	Il1r1
ENSMUSG00000026121	22.85787	5.42174	2.0759	1.50E-05	7.11E-05	Sema4c
ENSMUSG00000026185	139.363	67.38448	1.0484	7.95E-15	1.07E-13	Igfbp5
ENSMUSG00000026187	40.701	18.43392	1.1427	1.22E-05	5.87E-05	Xrcc5
ENSMUSG00000026193	578.5606	68.62374	3.0757	########	########	Fn1
ENSMUSG00000026208	425.0863	141.5849	1.5861	2.35E-61	1.36E-59	Des
ENSMUSG00000026235	22.508	5.111926	2.1385	1.36E-05	6.48E-05	Epha4
ENSMUSG00000026278	31.48788	14.25143	1.1437	0.000119	0.00047	Bok
ENSMUSG00000026303	113.2397	43.37392	1.3845	1.27E-15	1.81E-14	Mlph
ENSMUSG00000026327	8.280146	0	5.7402	0.000936	0.002939	Serpinb11
ENSMUSG00000026335	157.556	43.99355	1.8405	4.52E-27	1.11E-25	Pam
ENSMUSG00000026365	244.3226	33.30497	2.875	3.83E-58	2.07E-56	Cfh
ENSMUSG00000026380	303.5665	70.79243	2.1003	1.32E-56	7.02E-55	Tfcp2l1
ENSMUSG00000026389	13.99461	3.098137	2.1754	0.00055	0.00184	Steap3
ENSMUSG00000026393	90.73174	21.99677	2.0443	1.07E-17	1.70E-16	Nek7
ENSMUSG00000026405	11.89542	1.703975	2.8034	0.000438	0.001509	C4bp
ENSMUSG00000026411	25.89003	6.506088	1.9925	6.04E-06	3.09E-05	Tmem9
ENSMUSG00000026418	15.62732	0	4.3346	6.70E-06	3.39E-05	Tnni1
ENSMUSG00000026421	350.2152	102.8582	1.7676	4.80E-56	2.50E-54	Csrp1
ENSMUSG00000026435	61.10981	9.604225	2.6697	4.86E-15	6.67E-14	Slc45a3
ENSMUSG00000026473	242.6899	52.35852	2.2126	1.37E-47	5.50E-46	Glul
ENSMUSG00000026475	7.813658	0	4.0716	0.001615	0.004722	Rgs16
ENSMUSG00000026478	82.56821	39.81106	1.0524	2.09E-09	1.71E-08	Lamc1
ENSMUSG00000026480	46.76533	13.01218	1.8456	4.17E-09	3.28E-08	Ncf2
ENSMUSG00000026483	122.5695	23.70075	2.3706	3.18E-26	7.57E-25	Fam129a
ENSMUSG00000026519	88.63254	34.07951	1.3789	1.64E-12	1.83E-11	Tmem63a
ENSMUSG00000026520	24.95706	11.77292	1.084	0.000838	0.002669	Pycr2
ENSMUSG00000026531	26.47314	1.084348	4.6096	3.57E-09	2.83E-08	Mptx1
ENSMUSG00000026574	41.28411	7.745343	2.4142	5.90E-10	5.10E-09	Dpt
ENSMUSG00000026581	8.630011	0	3.7999	0.001079	0.003315	Sell
ENSMUSG00000026639	139.363	50.49963	1.4645	8.03E-20	1.42E-18	Lamb3
ENSMUSG00000026656	23.67422	6.660995	1.8295	3.11E-05	0.000138	Fcgr2b
ENSMUSG00000026674	27.8726	11.61801	1.2625	0.000147	0.000568	Ddr2
ENSMUSG00000026676	21.92489	5.731554	1.9356	3.94E-05	0.000172	Ccdc3
ENSMUSG00000026712	29.85517	11.3082	1.4006	3.64E-05	0.00016	Mrc1
ENSMUSG00000026727	43.96641	13.16708	1.7395	2.90E-08	2.06E-07	Rsu1
ENSMUSG00000026728	199.3066	48.48585	2.0394	7.31E-37	2.39E-35	Vim
ENSMUSG00000026779	20.64205	6.506088	1.6657	0.000195	0.000734	Mastl
ENSMUSG00000026796	132.4823	47.09168	1.4923	3.12E-19	5.37E-18	Fam129b
ENSMUSG00000026797	62.74251	11.46311	2.4524	1.56E-14	2.02E-13	Stxbp1

ENSMUSG00000026811	670.5752	9.759132	6.1025	########	########	St6galnac6
ENSMUSG00000026828	43.73316	7.90025	2.4688	1.27E-10	1.19E-09	Galnt5
ENSMUSG00000026837	73.47171	16.26522	2.1754	2.44E-15	3.42E-14	Col5a1
ENSMUSG00000026870	47.46506	2.94323	4.0114	9.15E-15	1.23E-13	Cutal
ENSMUSG00000026879	228.462	102.7032	1.1535	2.22E-25	5.03E-24	Gsn
ENSMUSG00000026970	71.60577	27.72833	1.3687	2.53E-10	2.28E-09	Rbms1
ENSMUSG00000026981	87.93281	5.42174	4.0196	4.90E-26	1.15E-24	Il1rn
ENSMUSG00000027068	101.6942	7.590436	3.7439	4.78E-29	1.27E-27	Dhrs9
ENSMUSG00000027189	49.68087	18.74373	1.4063	9.45E-08	6.35E-07	Trim44
ENSMUSG00000027199	24.84044	3.562858	2.8016	3.78E-07	2.34E-06	Gatm
ENSMUSG00000027204	48.16479	19.05354	1.3379	2.88E-07	1.82E-06	Fbn1
ENSMUSG00000027230	134.3483	26.48907	2.3425	2.50E-28	6.42E-27	Creb3l1
ENSMUSG00000027298	17.49327	6.506088	1.4269	0.001428	0.004249	Tyro3
ENSMUSG00000027331	37.08572	18.27901	1.0207	7.60E-05	0.000315	Knstrn
ENSMUSG00000027333	62.27602	29.2774	1.0889	1.24E-07	8.24E-07	Smox
ENSMUSG00000027375	32.88734	2.94323	3.4821	4.10E-10	3.61E-09	Mal
ENSMUSG00000027398	32.30423	4.647206	2.7973	7.05E-09	5.36E-08	Il1b
ENSMUSG00000027401	67.75725	0	7.0823	8.80E-20	1.55E-18	Tgm3
ENSMUSG00000027435	25.07368	10.22385	1.2942	0.000268	0.000984	Cd93
ENSMUSG00000027469	61.69292	28.81268	1.0984	1.26E-07	8.36E-07	Tpx2
ENSMUSG00000027556	203.3884	0	8.0367	4.06E-51	1.81E-49	Car1
ENSMUSG00000027559	13.29488	1.239255	3.4233	7.62E-05	0.000315	Car3
ENSMUSG00000027562	272.1952	37.64237	2.8542	2.95E-64	1.75E-62	Car2
ENSMUSG00000027602	23.09111	10.53367	1.1323	0.001037	0.003213	Map1lc3a
ENSMUSG00000027684	80.93551	35.93839	1.1712	4.33E-10	3.80E-09	Mecom
ENSMUSG00000027712	74.52131	24.32038	1.6155	2.98E-12	3.24E-11	Anxa5
ENSMUSG00000027737	38.13532	2.788323	3.7737	6.80E-12	7.21E-11	Slc7a11
ENSMUSG00000027750	238.6081	9.449318	4.6583	2.07E-70	1.36E-68	Postn
ENSMUSG00000027803	24.95706	10.37876	1.2658	0.000322	0.001155	Wwtr1
ENSMUSG00000027848	13.52813	2.94323	2.2005	0.000641	0.002104	Olfml3
ENSMUSG00000027858	15.86056	4.647206	1.771	0.000782	0.002507	Tspan2
ENSMUSG00000027870	38.95167	0	6.2836	1.28E-12	1.43E-11	Hao2
ENSMUSG00000027875	38.83505	12.39255	1.6479	3.68E-07	2.28E-06	Hmgcs2
ENSMUSG00000027876	208.8696	72.3415	1.5297	4.76E-30	1.31E-28	Reg4
ENSMUSG00000027907	335.8707	147.6262	1.186	1.82E-37	5.97E-36	S100a11
ENSMUSG00000027938	20.29219	8.674784	1.226	0.001402	0.004176	Creb3l4
ENSMUSG00000027962	22.15814	6.506088	1.768	7.27E-05	0.000302	Vcam1
ENSMUSG00000027995	24.72381	5.886461	2.0704	6.91E-06	3.49E-05	Tlr2
ENSMUSG00000028031	22.04151	0	4.5677	7.39E-08	5.03E-07	Dkk2
ENSMUSG00000028128	76.03739	26.79889	1.5045	9.26E-12	9.70E-11	F3
ENSMUSG00000028173	104.4931	15.33578	2.7684	3.23E-25	7.26E-24	Wls
ENSMUSG00000028175	14.57772	3.407951	2.0968	0.000518	0.001745	Depdc1a
ENSMUSG00000028194	78.13658	12.23764	2.6747	7.97E-19	1.35E-17	Ddah1
ENSMUSG00000028195	16.67691	3.717765	2.1653	0.000167	0.000639	Cyr61
ENSMUSG00000028199	22.04151	0	4.5677	7.39E-08	5.03E-07	Cryz
ENSMUSG00000028211	165.6029	62.27256	1.4111	1.67E-22	3.37E-21	Trp53inp1
ENSMUSG00000028270	20.99192	6.041367	1.7969	0.0001	0.000404	Gbp2

ENSMUSG00000028293	106.7089	19.51826	2.4508	1.29E-23	2.75E-22	Slc35a1
ENSMUSG00000028334	377.9712	97.28151	1.958	5.46E-66	3.27E-64	Nans
ENSMUSG00000028347	27.40612	5.42174	2.3377	6.32E-07	3.78E-06	Tmeff1
ENSMUSG00000028357	14.57772	2.013789	2.8558	8.95E-05	0.000364	Kif12
ENSMUSG00000028358	21.45841	2.168696	3.3066	6.43E-07	3.84E-06	Zfp618
ENSMUSG00000028364	118.6043	34.07951	1.7992	2.19E-20	3.98E-19	Tnc
ENSMUSG00000028369	30.43828	6.660995	2.1921	3.19E-07	1.99E-06	Svep1
ENSMUSG00000028392	116.3885	45.0779	1.3685	7.42E-16	1.08E-14	Bspry
ENSMUSG00000028464	198.3736	81.3261	1.2864	1.60E-24	3.47E-23	Tpm2
ENSMUSG00000028479	299.0182	62.27256	2.2636	2.67E-59	1.47E-57	Gne
ENSMUSG00000028525	21.22516	7.745343	1.4544	0.000393	0.001372	Pde4b
ENSMUSG00000028527	120.1204	24.32038	2.3042	3.48E-25	7.78E-24	Ak4
ENSMUSG00000028528	13.4115	0	4.114	3.50E-05	0.000154	Dnajc6
ENSMUSG00000028532	24.25733	5.111926	2.2465	4.02E-06	2.11E-05	Cachd1
ENSMUSG00000028538	34.63667	5.111926	2.7604	2.42E-09	1.95E-08	St3gal3
ENSMUSG00000028555	112.3068	28.50286	1.9783	5.73E-21	1.09E-19	Ttc39a
ENSMUSG00000028599	91.66471	15.02597	2.6089	1.93E-21	3.76E-20	Tnfrsf1b
ENSMUSG00000028602	17.1434	1.084348	3.9828	3.29E-06	1.76E-05	Tnfrsf8
ENSMUSG00000028617	80.11915	18.1241	2.1442	2.09E-16	3.13E-15	Lrrc42
ENSMUSG00000028633	41.98384	19.51826	1.105	1.23E-05	5.91E-05	Ctps
ENSMUSG00000028645	161.4045	51.73889	1.6414	4.31E-25	9.59E-24	Slc2a1
ENSMUSG00000028654	34.63667	15.8005	1.1323	5.88E-05	0.000249	Mycl
ENSMUSG00000028678	23.90746	10.99839	1.1202	0.000898	0.00283	Kif2c
ENSMUSG00000028698	53.0629	16.57503	1.6787	2.04E-09	1.67E-08	Pik3r3
ENSMUSG00000028699	1120.035	99.76002	3.4889	########	########	Tspan1
ENSMUSG00000028716	54.57899	4.182485	3.7059	2.94E-16	4.34E-15	Pdzk1ip1
ENSMUSG00000028749	88.86579	18.1241	2.2937	5.55E-19	9.47E-18	Pla2g2f
ENSMUSG00000028763	102.8604	47.09168	1.1271	4.90E-12	5.27E-11	Hspg2
ENSMUSG00000028776	94.69688	43.99355	1.106	5.07E-11	4.90E-10	Tinagl1
ENSMUSG00000028780	187.8777	12.70236	3.8866	5.12E-53	2.46E-51	Sema3c
ENSMUSG00000028860	26.70638	4.957019	2.4296	5.92E-07	3.56E-06	Sytl1
ENSMUSG00000028989	11.19569	0	4.4849	0.000129	0.000508	Angptl7
ENSMUSG00000029007	32.6541	14.56124	1.1651	7.67E-05	0.000317	Agtrap
ENSMUSG00000029070	22.27476	9.294411	1.261	0.000694	0.002256	Mxra8
ENSMUSG00000029084	341.0021	155.9912	1.1283	2.53E-36	8.24E-35	Cd38
ENSMUSG00000029093	39.30154	6.196274	2.6651	3.48E-10	3.09E-09	Sorcs2
ENSMUSG00000029094	14.34448	4.027578	1.8325	0.001176	0.003576	Afap1
ENSMUSG00000029102	92.1312	32.53044	1.5019	6.47E-14	8.06E-13	Hgfac
ENSMUSG00000029103	47.11519	23.39094	1.0102	9.08E-06	4.45E-05	Lrpap1
ENSMUSG00000029108	17.95975	4.492299	1.9992	0.00016	0.000615	Pcdh7
ENSMUSG00000029154	26.12328	2.168696	3.5904	1.98E-08	1.43E-07	Cwh43
ENSMUSG00000029246	34.05356	16.42013	1.0523	0.00012	0.000474	Ppat
ENSMUSG00000029247	128.6338	44.76808	1.5227	4.75E-19	8.12E-18	Paics
ENSMUSG00000029304	38.13532	5.266833	2.8561	2.35E-10	2.13E-09	Spp1
ENSMUSG00000029309	193.009	89.07144	1.1156	4.63E-21	8.86E-20	Sparcl1
ENSMUSG00000029371	95.74647	4.647206	4.3648	6.63E-29	1.74E-27	Cxcl5
ENSMUSG00000029373	13.29488	1.703975	2.9639	0.000152	0.000585	Pf4

ENSMUSG00000029380	13.06164	1.239255	3.3978	9.11E-05	0.00037	Cxcl1
ENSMUSG00000029445	28.68896	12.08273	1.2475	0.000128	0.000504	Hpd
ENSMUSG00000029484	161.2879	15.49069	3.3802	6.53E-43	2.40E-41	Anxa3
ENSMUSG00000029569	77.90334	28.34795	1.4584	1.05E-11	1.09E-10	Tmem168
ENSMUSG00000029570	21.34178	6.506088	1.7138	0.000124	0.000489	Lfng
ENSMUSG00000029581	14.81096	4.647206	1.6722	0.001569	0.004611	Fscn1
ENSMUSG00000029661	311.9632	72.65132	2.1023	3.61E-58	1.97E-56	Col1a2
ENSMUSG00000029675	35.56964	6.815902	2.3837	1.07E-08	7.94E-08	Eln
ENSMUSG00000029718	18.193	6.660995	1.4496	0.001051	0.003243	Pcolce
ENSMUSG00000029761	267.647	85.8184	1.641	1.70E-40	5.90E-39	Cald1
ENSMUSG00000029763	65.19157	26.02435	1.3248	2.82E-09	2.25E-08	Exoc4
ENSMUSG00000029781	57.72778	12.08273	2.2563	1.04E-12	1.17E-11	Fkbp9
ENSMUSG00000029832	22.39138	3.872671	2.5315	3.38E-06	1.81E-05	Nfe2l3
ENSMUSG00000029838	36.73586	4.337392	3.0823	1.80E-10	1.65E-09	Ptn
ENSMUSG00000029851	27.40612	10.37876	1.4009	7.60E-05	0.000315	Fam115c
ENSMUSG00000029865	370.3907	1.858882	7.6385	1.62E-95	1.50E-93	Sval1
ENSMUSG00000029868	40.35113	0	8.0251	2.04E-11	2.04E-10	Trpv6
ENSMUSG00000029869	20.40881	0	4.7197	2.06E-07	1.32E-06	Ephb6
ENSMUSG00000029919	13.64475	3.562858	1.9372	0.001177	0.003578	Hpgds
ENSMUSG00000030064	51.78007	22.30659	1.2149	3.76E-07	2.33E-06	Frmd4b
ENSMUSG00000030067	89.91538	39.50125	1.1867	3.59E-11	3.52E-10	Foxp1
ENSMUSG00000030123	22.85787	10.37876	1.1391	0.001064	0.003277	Plxnd1
ENSMUSG00000030218	61.22643	8.210063	2.8987	7.07E-16	1.03E-14	Mgp
ENSMUSG00000030223	21.45841	3.253044	2.7217	2.97E-06	1.61E-05	Ptpro
ENSMUSG00000030247	20.17557	7.280622	1.4705	0.000513	0.001731	Kcnj8
ENSMUSG00000030263	46.64871	8.055156	2.5339	1.96E-11	1.97E-10	Lrmp
ENSMUSG00000030340	68.69022	12.85727	2.4175	1.32E-15	1.88E-14	Scnn1a
ENSMUSG00000030342	358.2621	62.11765	2.5279	5.01E-77	3.63E-75	Cd9
ENSMUSG00000030366	10.61258	0	6.0982	0.000198	0.000746	Ceacam12
ENSMUSG00000030413	401.1789	143.4437	1.4838	1.39E-54	6.88E-53	Pglyrp1
ENSMUSG00000030471	182.7463	67.22958	1.4427	3.72E-25	8.29E-24	Zdhhc13
ENSMUSG00000030554	114.0561	45.2328	1.3343	3.11E-15	4.31E-14	Synm
ENSMUSG00000030579	18.42624	7.280622	1.3396	0.001494	0.004416	Tyrobp
ENSMUSG00000030605	86.99984	41.51504	1.0674	6.00E-10	5.17E-09	Mfge8
ENSMUSG00000030616	60.06021	22.92621	1.3894	5.40E-09	4.18E-08	Sytl2
ENSMUSG00000030653	63.79211	15.33578	2.0565	5.96E-13	6.88E-12	Pde2a
ENSMUSG00000030671	25.89003	8.674784	1.5775	4.75E-05	0.000204	Pde3b
ENSMUSG00000030711	25.89003	5.266833	2.2974	1.53E-06	8.67E-06	Sult1a1
ENSMUSG00000030717	138.7799	24.7851	2.4853	1.56E-30	4.36E-29	Nupr1
ENSMUSG00000030729	34.2868	11.77292	1.5422	3.63E-06	1.93E-05	Pgm2l1
ENSMUSG00000030759	183.9125	16.72994	3.4585	2.88E-49	1.19E-47	Far1
ENSMUSG00000030762	70.67279	0	6.8336	6.02E-21	1.14E-19	Aqp8
ENSMUSG00000030770	115.3389	44.30336	1.3804	7.62E-16	1.11E-14	Parva
ENSMUSG00000030772	45.71573	19.98298	1.1939	2.20E-06	1.21E-05	Dkk3
ENSMUSG00000030796	22.85787	3.098137	2.8832	8.59E-07	5.06E-06	Tead2
ENSMUSG00000030798	13.99461	3.098137	2.1754	0.00055	0.00184	Cd37
ENSMUSG00000030866	47.81493	18.43392	1.3751	2.22E-07	1.41E-06	Ern2

ENSMUSG00000030873	13.87799	0	5.4853	1.74E-05	8.14E-05	Scnn1b
ENSMUSG00000030935	70.43955	3.562858	4.3053	1.28E-21	2.52E-20	Acsm3
ENSMUSG00000030986	132.0158	63.82162	1.0486	3.98E-14	5.06E-13	Dhx32
ENSMUSG00000030987	81.9851	30.82646	1.4112	6.54E-12	6.95E-11	Stim1
ENSMUSG00000031075	119.0708	13.01218	3.1939	3.76E-31	1.09E-29	Ano1
ENSMUSG00000031089	591.1557	26.64398	4.4717	########	########	Slc6a14
ENSMUSG00000031146	65.42481	19.67317	1.7336	1.42E-11	1.45E-10	Plp2
ENSMUSG00000031156	91.54809	44.61317	1.0371	3.76E-10	3.33E-09	Slc35a2
ENSMUSG00000031176	80.11915	39.50125	1.0202	6.08E-09	4.69E-08	Dynlt3
ENSMUSG00000031207	97.84566	34.54423	1.5021	1.09E-14	1.45E-13	Msn
ENSMUSG00000031257	102.3939	2.633417	5.2811	1.61E-31	4.71E-30	Nox1
ENSMUSG00000031278	43.84978	19.82808	1.145	5.50E-06	2.83E-05	Acsl4
ENSMUSG00000031328	562.1169	219.0383	1.3597	7.32E-70	4.75E-68	Flna
ENSMUSG00000031360	29.27206	12.85727	1.1869	0.000158	0.000609	Ctps2
ENSMUSG00000031375	139.363	48.95057	1.5095	2.37E-20	4.30E-19	Bgn
ENSMUSG00000031430	39.6514	4.027578	3.2994	1.36E-11	1.39E-10	Vsig1
ENSMUSG00000031441	64.02535	13.78671	2.2154	9.55E-14	1.18E-12	Atp11a
ENSMUSG00000031442	51.19696	24.7851	1.0466	2.54E-06	1.39E-05	Mcf2l
ENSMUSG00000031451	194.9916	67.38448	1.5329	3.25E-28	8.29E-27	Gas6
ENSMUSG00000031502	335.7541	93.71865	1.841	9.15E-56	4.65E-54	Col4a1
ENSMUSG00000031503	213.4178	67.8492	1.6533	7.46E-33	2.24E-31	Col4a2
ENSMUSG00000031530	27.40612	13.32199	1.0407	0.000597	0.001975	Dusp4
ENSMUSG00000031538	91.89795	36.86783	1.3177	1.97E-12	2.17E-11	Plat
ENSMUSG00000031548	41.40073	18.27901	1.1795	7.50E-06	3.76E-05	Sfrp1
ENSMUSG00000031563	20.05894	6.506088	1.6244	0.000284	0.001033	Wwc2
ENSMUSG00000031591	245.7221	115.2507	1.0923	7.06E-26	1.63E-24	Asah1
ENSMUSG00000031617	22.15814	4.802113	2.2061	1.22E-05	5.89E-05	Tmem184c
ENSMUSG00000031626	50.96371	7.280622	2.8073	3.31E-13	3.88E-12	Sorbs2
ENSMUSG00000031633	106.9422	52.51342	1.0261	1.63E-11	1.66E-10	Slc25a4
ENSMUSG00000031636	57.72778	20.13789	1.5194	2.40E-09	1.94E-08	Pdlim3
ENSMUSG00000031661	85.95024	18.89864	2.1852	9.58E-18	1.53E-16	Nkd1
ENSMUSG00000031700	16.91016	4.957019	1.7703	0.000524	0.001764	Gpt2
ENSMUSG00000031722	45.59911	17.50447	1.3813	3.99E-07	2.45E-06	Hp
ENSMUSG00000031740	48.86452	14.87106	1.7163	6.17E-09	4.74E-08	Mmp2
ENSMUSG00000031757	11.66218	0	4.5438	9.14E-05	0.000371	Mt4
ENSMUSG00000031760	17.95975	1.703975	3.3978	4.46E-06	2.33E-05	Mt3
ENSMUSG00000031775	72.53874	33.9246	1.0964	1.03E-08	7.68E-08	Pllp
ENSMUSG00000031785	55.86183	27.1087	1.0431	9.28E-07	5.43E-06	Gpr56
ENSMUSG00000031790	100.9945	22.46149	2.1688	1.86E-20	3.42E-19	Mmp15
ENSMUSG00000031799	263.5652	105.9563	1.3147	1.12E-32	3.34E-31	Tpm4
ENSMUSG00000031808	22.97449	11.15329	1.0426	0.001657	0.004831	Slc27a1
ENSMUSG00000031824	60.75994	23.23603	1.3868	4.55E-09	3.56E-08	6430548M08Rik
ENSMUSG00000031825	33.2372	10.84348	1.616	3.14E-06	1.69E-05	Crispld2
ENSMUSG00000031841	19.24259	2.788323	2.7868	8.10E-06	4.03E-05	Cdh13
ENSMUSG00000031844	58.54413	20.13789	1.5396	1.47E-09	1.23E-08	Hsd17b2
ENSMUSG00000031891	175.2825	7.745343	4.5002	7.34E-52	3.38E-50	Hsd11b2
ENSMUSG00000032011	22.97449	10.22385	1.1681	0.000897	0.002827	Thy1

ENSMUSG00000032024	40.93424	16.26522	1.3315	2.36E-06	1.29E-05	Clmp
ENSMUSG00000032026	40.46775	19.51826	1.0519	2.76E-05	0.000124	Rexo2
ENSMUSG00000032028	41.75059	0	6.4893	2.81E-13	3.31E-12	Nxpe2
ENSMUSG00000032050	31.83774	14.40634	1.144	0.000108	0.000434	Rdx
ENSMUSG00000032068	573.3126	55.92137	3.3579	########	########	Plet1
ENSMUSG00000032085	870.9314	155.5265	2.4854	########	########	Tagln
ENSMUSG00000032109	33.2372	10.68857	1.6367	2.74E-06	1.49E-05	Nlrx1
ENSMUSG00000032135	39.53478	19.67317	1.0069	4.93E-05	0.000211	Mcam
ENSMUSG00000032194	29.38869	14.09652	1.0599	0.000336	0.001195	Kank2
ENSMUSG00000032232	61.45967	10.22385	2.5877	8.35E-15	1.12E-13	Cgnl1
ENSMUSG00000032245	28.10585	12.70236	1.1458	0.000273	0.000999	Cln6
ENSMUSG00000032261	210.6189	94.49318	1.1564	1.41E-23	2.99E-22	Sh3bgrl2
ENSMUSG00000032278	25.1903	8.674784	1.538	7.34E-05	0.000305	Paqr5
ENSMUSG00000032289	27.17287	9.294411	1.5477	3.62E-05	0.000159	Thsd4
ENSMUSG00000032290	48.28141	17.50447	1.4637	8.22E-08	5.56E-07	Ptpn9
ENSMUSG00000032320	38.13532	15.95541	1.2571	9.34E-06	4.57E-05	Rcn2
ENSMUSG00000032327	44.54952	7.280622	2.6133	3.29E-11	3.24E-10	Stra6
ENSMUSG00000032332	85.25051	22.15168	1.9443	4.56E-16	6.67E-15	Col12a1
ENSMUSG00000032334	15.16083	3.407951	2.1534	0.000343	0.001215	Loxl1
ENSMUSG00000032349	56.67818	25.24982	1.1665	1.86E-07	1.20E-06	Elovl5
ENSMUSG00000032374	18.193	4.337392	2.0685	0.000116	0.000458	Plod2
ENSMUSG00000032420	183.2128	52.20361	1.8113	8.75E-31	2.50E-29	Nt5e
ENSMUSG00000032481	74.75455	36.40311	1.0381	1.49E-08	1.09E-07	Smarcc1
ENSMUSG00000032512	62.27602	28.65777	1.1198	8.39E-08	5.67E-07	Wdr48
ENSMUSG00000032702	68.10711	14.40634	2.2411	1.18E-14	1.56E-13	Kank1
ENSMUSG00000032718	56.67818	17.34957	1.7079	4.23E-10	3.72E-09	Mansc1
ENSMUSG00000032733	13.99461	3.717765	1.9124	0.001088	0.003341	Snx33
ENSMUSG00000032754	24.25733	9.294411	1.384	0.000215	0.000804	Slc8b1
ENSMUSG00000032776	36.9691	6.815902	2.4393	3.99E-09	3.15E-08	Mctp2
ENSMUSG00000032849	36.9691	10.06895	1.8764	1.40E-07	9.23E-07	Abcc4
ENSMUSG00000032875	22.15814	6.970809	1.6684	0.000112	0.000446	Arhgef17
ENSMUSG00000032883	152.0748	60.72349	1.3245	1.14E-19	1.99E-18	Acsl3
ENSMUSG00000032898	22.97449	9.294411	1.3056	0.00046	0.001573	Fbxo21
ENSMUSG00000033022	32.53747	14.25143	1.191	6.63E-05	0.000278	Cdo1
ENSMUSG00000033032	30.67153	11.61801	1.4005	2.85E-05	0.000128	Afap1l1
ENSMUSG00000033059	218.316	50.18982	2.121	1.54E-41	5.43E-40	Pygb
ENSMUSG00000033066	20.05894	7.435529	1.4317	0.000626	0.002057	Gas7
ENSMUSG00000033149	34.52004	13.78671	1.3242	1.54E-05	7.28E-05	Phldb2
ENSMUSG00000033192	36.61924	7.590436	2.2703	1.27E-08	9.40E-08	Lpcat2
ENSMUSG00000033200	19.70908	7.280622	1.4367	0.000684	0.002228	Tpsg1
ENSMUSG00000033213	380.8867	153.5127	1.311	2.45E-46	9.52E-45	AA467197
ENSMUSG00000033287	18.07637	4.027578	2.1661	8.87E-05	0.000361	Kctd17
ENSMUSG00000033327	31.02139	11.46311	1.4363	2.05E-05	9.43E-05	Tnxb
ENSMUSG00000033420	24.60719	8.519877	1.5302	9.25E-05	0.000375	Antxr1
ENSMUSG00000033508	10.26272	0	3.7279	0.000385	0.001346	Asprv1
ENSMUSG00000033579	57.96102	0	6.857	1.97E-17	3.09E-16	Fa2h
ENSMUSG00000033590	144.7276	37.95218	1.9311	4.99E-26	1.16E-24	Myo5c

ENSMUSG00000033715	140.1794	35.16386	1.9951	5.94E-26	1.38E-24	Akr1c14
ENSMUSG00000033721	10.7292	2.013789	2.4136	0.001595	0.004671	Vav3
ENSMUSG00000033849	11.54556	0	5.2198	8.77E-05	0.000358	B3galt2
ENSMUSG00000033952	28.45571	13.78671	1.0454	0.000456	0.001559	Aspm
ENSMUSG00000034006	28.80558	12.39255	1.2169	0.000149	0.000577	Pqlc1
ENSMUSG00000034055	16.91016	5.886461	1.5224	0.001225	0.003701	Phka1
ENSMUSG00000034112	88.04944	21.68696	2.0215	4.55E-17	7.00E-16	Atp2c2
ENSMUSG00000034205	73.47171	19.36336	1.9239	6.12E-14	7.65E-13	Loxl2
ENSMUSG00000034220	30.20504	4.957019	2.6072	4.84E-08	3.37E-07	Gpc1
ENSMUSG00000034312	24.25733	9.759132	1.3136	0.000306	0.001107	Iqsec1
ENSMUSG00000034353	41.40073	8.829691	2.2292	1.93E-09	1.58E-08	Ramp1
ENSMUSG00000034362	8.513389	0	4.0897	0.000988	0.003082	Csta1
ENSMUSG00000034401	26.2399	2.788323	3.2343	4.51E-08	3.15E-07	Spata6
ENSMUSG00000034480	21.80827	8.055156	1.4369	0.000354	0.001251	Diap2
ENSMUSG00000034573	16.91016	3.717765	2.1854	0.000142	0.000551	Ptpn13
ENSMUSG00000034607	144.4944	63.51181	1.1859	4.88E-17	7.47E-16	Pof1b
ENSMUSG00000034675	26.47314	6.196274	2.0951	2.92E-06	1.58E-05	Dbn1
ENSMUSG00000034684	26.70638	10.37876	1.3636	0.000115	0.000458	Sema3f
ENSMUSG00000034723	18.89273	7.745343	1.2864	0.001611	0.004709	Tmx4
ENSMUSG00000034981	237.4419	33.30497	2.8338	5.15E-56	2.67E-54	Parm1
ENSMUSG00000035165	116.7384	57.31554	1.0263	1.95E-12	2.15E-11	Kcne3
ENSMUSG00000035184	50.26398	3.098137	4.0201	1.48E-15	2.09E-14	Fam124a
ENSMUSG00000035284	49.7975	16.42013	1.6006	1.35E-08	9.90E-08	Vps13c
ENSMUSG00000035451	73.70496	17.19466	2.0998	5.69E-15	7.76E-14	Foxa1
ENSMUSG00000035547	139.0131	39.19143	1.8266	6.35E-24	1.36E-22	Capn5
ENSMUSG00000035557	9.91285	0	5.9998	0.000313	0.001128	Krt17
ENSMUSG00000035776	23.67422	7.280622	1.7012	5.60E-05	0.000238	Cd99l2
ENSMUSG00000035781	113.473	46.00734	1.3024	7.50E-15	1.02E-13	R3hdm4
ENSMUSG00000035783	530.7457	168.0739	1.6589	2.51E-79	1.88E-77	Acta2
ENSMUSG00000035847	37.55221	14.56124	1.3668	4.71E-06	2.45E-05	Ids
ENSMUSG00000035901	29.27206	12.85727	1.1869	0.000158	0.000609	Dennd5a
ENSMUSG00000035914	16.32705	5.42174	1.5904	0.001184	0.003594	Cd276
ENSMUSG00000035967	38.0187	14.71615	1.3693	4.03E-06	2.12E-05	Ddx26b
ENSMUSG00000036078	88.3993	29.74212	1.5715	6.22E-14	7.77E-13	Sigmar1
ENSMUSG00000036103	18.89273	3.407951	2.4709	2.36E-05	0.000107	Colec12
ENSMUSG00000036256	140.296	66.30013	1.0814	2.57E-15	3.59E-14	Igfbp7
ENSMUSG00000036273	18.89273	3.562858	2.4067	2.86E-05	0.000128	Lrrk2
ENSMUSG00000036298	19.47584	2.168696	3.1668	2.84E-06	1.54E-05	Slc2a13
ENSMUSG00000036437	9.329741	0	4.3274	0.000504	0.001705	Npy1r
ENSMUSG00000036446	28.68896	8.055156	1.8325	4.46E-06	2.33E-05	Lum
ENSMUSG00000036533	17.02678	4.802113	1.8261	0.000417	0.00144	Cdc42ep3
ENSMUSG00000036545	17.26002	4.802113	1.8457	0.000356	0.001257	Adamts2
ENSMUSG00000036585	22.39138	10.37876	1.1093	0.001381	0.004123	Fgf1
ENSMUSG00000036667	22.74124	5.886461	1.9498	2.66E-05	0.00012	Fam115a
ENSMUSG00000036745	25.77341	7.90025	1.7059	2.56E-05	0.000116	Ttll7
ENSMUSG00000036834	40.93424	17.81429	1.2003	7.09E-06	3.57E-05	Plch1
ENSMUSG00000036902	14.4611	1.858882	2.9597	7.85E-05	0.000324	Neto2

ENSMUSG00000036905	45.59911	22.30659	1.0315	1.04E-05	5.03E-05	C1qb
ENSMUSG00000037012	147.8764	28.34795	2.3831	2.02E-31	5.87E-30	Hk1
ENSMUSG00000037025	22.15814	3.098137	2.8384	1.44E-06	8.18E-06	Foxa2
ENSMUSG00000037033	1145.226	225.0797	2.3471	########	########	Clca4
ENSMUSG00000037060	23.67422	6.041367	1.9704	1.66E-05	7.81E-05	Prkcdbp
ENSMUSG00000037071	72.07225	6.815902	3.4025	3.72E-20	6.72E-19	Scd1
ENSMUSG00000037095	354.5302	146.8517	1.2715	4.69E-42	1.66E-40	Lrg1
ENSMUSG00000037104	20.17557	6.506088	1.6327	0.000263	0.000965	Socs5
ENSMUSG00000037129	10.61258	0	4.4077	0.0002	0.00075	Tmprss13
ENSMUSG00000037362	114.1727	0	7.9406	9.45E-30	2.55E-28	Nov
ENSMUSG00000037379	21.45841	8.055156	1.4136	0.000438	0.001509	Spon2
ENSMUSG00000037405	24.14071	7.435529	1.699	4.78E-05	0.000206	Icam1
ENSMUSG00000037419	210.1524	25.24982	3.0571	2.86E-52	1.35E-50	Endod1
ENSMUSG00000037499	19.8257	7.280622	1.4452	0.000637	0.002089	Nenf
ENSMUSG00000037664	16.91016	5.266833	1.6829	0.000704	0.002287	Cdkn1c
ENSMUSG00000037788	39.41816	12.70236	1.6338	3.36E-07	2.09E-06	Vopp1
ENSMUSG00000037860	65.77468	8.055156	3.0295	2.13E-17	3.32E-16	Aim2
ENSMUSG00000037902	28.22247	11.77292	1.2614	0.000134	0.000526	Sirpa
ENSMUSG00000037936	21.45841	8.984598	1.256	0.00089	0.002813	Scarb1
ENSMUSG00000037953	17.26002	2.94323	2.552	4.28E-05	0.000186	A4gnt
ENSMUSG00000037972	17.02678	5.111926	1.7359	0.000563	0.001877	Snn
ENSMUSG00000038112	8.046902	0	3.699	0.001702	0.004939	AW551984
ENSMUSG00000038119	25.42355	8.36497	1.6037	4.86E-05	0.000209	Cdon
ENSMUSG00000038175	22.85787	6.970809	1.7133	7.13E-05	0.000297	Mylip
ENSMUSG00000038203	42.91681	0	6.4235	1.18E-13	1.44E-12	Hoxa13
ENSMUSG00000038210	26.00665	0	5.7008	4.42E-09	3.47E-08	Hoxa11
ENSMUSG00000038227	49.68087	2.47851	4.3251	1.00E-15	1.44E-14	Hoxa9
ENSMUSG00000038236	31.02139	6.351181	2.2882	1.49E-07	9.77E-07	Hoxa7
ENSMUSG00000038305	51.08033	23.85566	1.0984	1.52E-06	8.62E-06	Spats2l
ENSMUSG00000038331	66.82427	2.94323	4.5049	8.24E-21	1.55E-19	Satb2
ENSMUSG00000038351	22.39138	10.53367	1.0879	0.001529	0.004503	Sgsm2
ENSMUSG00000038463	18.7761	2.323603	3.0145	5.99E-06	3.06E-05	Olfml2b
ENSMUSG00000038481	49.68087	20.2928	1.2917	2.98E-07	1.87E-06	Cdk19
ENSMUSG00000038521	62.1594	21.68696	1.5191	5.93E-10	5.12E-09	C1s1
ENSMUSG00000038732	133.0654	23.39094	2.5081	1.53E-29	4.13E-28	Mboat1
ENSMUSG00000038756	14.92759	0	4.8999	8.49E-06	4.20E-05	Ttll6
ENSMUSG00000038775	27.05625	11.3082	1.2586	0.000188	0.000709	Vill
ENSMUSG00000038807	65.77468	32.84025	1.0021	1.75E-07	1.13E-06	Rap1gap2
ENSMUSG00000038843	51.89669	2.633417	4.3006	2.46E-16	3.67E-15	Gcnt1
ENSMUSG00000038855	23.09111	9.139504	1.3371	0.000382	0.001338	Itpkb
ENSMUSG00000038872	21.57503	8.055156	1.4214	0.000408	0.001415	Zfhx3
ENSMUSG00000039005	39.41816	1.394162	4.8214	4.87E-13	5.67E-12	Tlr4
ENSMUSG00000039007	69.85644	22.6164	1.627	1.21E-11	1.24E-10	Cpq
ENSMUSG00000039081	19.94232	7.745343	1.3644	0.000859	0.002729	Zfp503
ENSMUSG00000039109	24.25733	2.633417	3.2034	1.57E-07	1.03E-06	F13a1
ENSMUSG00000039236	18.193	5.731554	1.6664	0.000468	0.001596	Isg20
ENSMUSG00000039238	13.06164	0	5.3978	3.05E-05	0.000136	Zfp750

ENSMUSG00000039270	17.02678	5.42174	1.651	0.000752	0.002427	Megf9
ENSMUSG00000039323	44.31627	1.703975	4.7009	2.00E-14	2.57E-13	Igfbp2
ENSMUSG00000039450	53.99588	22.30659	1.2754	1.10E-07	7.30E-07	Dcxr
ENSMUSG00000039530	43.84978	17.50447	1.3248	1.10E-06	6.35E-06	Tusc3
ENSMUSG00000039680	36.61924	17.34957	1.0777	5.47E-05	0.000233	Mrps6
ENSMUSG00000039697	29.03882	13.78671	1.0747	0.000333	0.001189	Ncoa7
ENSMUSG00000039701	43.03343	16.88485	1.3497	1.11E-06	6.43E-06	Usp53
ENSMUSG00000039774	76.50388	35.31876	1.1151	3.08E-09	2.46E-08	Galnt12
ENSMUSG00000039813	34.63667	11.46311	1.5953	2.25E-06	1.23E-05	Tbc1d2
ENSMUSG00000039943	33.12058	6.815902	2.2808	5.92E-08	4.09E-07	Plcb4
ENSMUSG00000039976	17.72651	4.802113	1.8842	0.00026	0.000955	Tbc1d16
ENSMUSG00000039985	51.31358	25.09491	1.0319	2.89E-06	1.57E-05	Fam60a
ENSMUSG00000040026	49.56425	16.42013	1.5938	1.55E-08	1.13E-07	Saa3
ENSMUSG00000040121	67.64063	15.02597	2.1704	3.23E-14	4.15E-13	Rep15
ENSMUSG00000040339	106.4757	44.61317	1.255	1.37E-13	1.66E-12	Fam102b
ENSMUSG00000040363	34.98653	15.8005	1.1468	4.86E-05	0.000209	Bcor
ENSMUSG00000040412	71.48914	24.16547	1.5648	1.65E-11	1.68E-10	5330417C22Rik
ENSMUSG00000040488	69.27333	28.03814	1.3049	1.20E-09	1.01E-08	Ltbp4
ENSMUSG00000040562	39.6514	17.19466	1.2054	9.46E-06	4.62E-05	Gstm2
ENSMUSG00000040605	28.33909	12.54746	1.1754	0.000216	0.000808	Bace2
ENSMUSG00000040666	19.00935	3.098137	2.6172	1.46E-05	6.91E-05	Sh3bgr
ENSMUSG00000040690	39.53478	6.041367	2.7102	2.41E-10	2.17E-09	Col16a1
ENSMUSG00000040711	38.36856	9.449318	2.0216	2.96E-08	2.10E-07	Sh3pxd2b
ENSMUSG00000040728	195.8079	96.35206	1.0231	8.86E-20	1.55E-18	Esrp1
ENSMUSG00000040747	25.30692	9.914039	1.352	0.000185	0.000703	Cd53
ENSMUSG00000040760	95.86309	33.15007	1.532	1.16E-14	1.53E-13	Appl1
ENSMUSG00000040852	15.97718	4.957019	1.6885	0.000972	0.003042	Plekhh2
ENSMUSG00000040891	39.30154	18.74373	1.0682	3.15E-05	0.00014	Foxa3
ENSMUSG00000040964	59.71035	15.49069	1.9466	1.04E-11	1.08E-10	Arhgef10l
ENSMUSG00000040990	43.3833	13.78671	1.6539	7.32E-08	5.00E-07	Sh3kbp1
ENSMUSG00000041025	28.22247	9.604225	1.5551	2.46E-05	0.000112	Iffo2
ENSMUSG00000041058	66.47441	28.96758	1.1984	1.07E-08	7.97E-08	Wwp1
ENSMUSG00000041119	546.6062	159.709	1.7751	9.03E-87	7.34E-85	Pde9a
ENSMUSG00000041293	42.10046	5.111926	3.0419	1.06E-11	1.10E-10	Gpr110
ENSMUSG00000041351	72.42212	27.41851	1.4013	1.25E-10	1.17E-09	Rap1gap
ENSMUSG00000041390	17.1434	4.802113	1.8359	0.000385	0.001346	Mdfic
ENSMUSG00000041439	197.9071	53.90759	1.8763	3.91E-34	1.21E-32	Mfsd6
ENSMUSG00000041476	8.396767	0	3.4384	0.001646	0.004801	Smpx
ENSMUSG00000041538	11.07907	1.084348	3.3529	0.00033	0.001179	H2-Ob
ENSMUSG00000041548	22.97449	7.590436	1.5978	0.000116	0.000459	Hspb8
ENSMUSG00000041570	31.6045	8.36497	1.9177	8.86E-07	5.20E-06	Camsap2
ENSMUSG00000041577	32.88734	11.77292	1.4821	8.52E-06	4.21E-05	Prelp
ENSMUSG00000041731	51.66344	25.09491	1.0417	2.41E-06	1.32E-05	Pgm5
ENSMUSG00000041734	14.69434	4.492299	1.7097	0.001465	0.004338	Kirrel
ENSMUSG00000041736	129.2169	57.78026	1.1611	4.03E-15	5.57E-14	Tspo
ENSMUSG00000041886	13.99461	2.788323	2.3274	0.000381	0.001333	Macc1
ENSMUSG00000041961	21.80827	9.139504	1.2547	0.000813	0.002596	Znrf3

ENSMUSG00000042029	41.98384	18.89864	1.1516	8.24E-06	4.09E-05	Ncapg2
ENSMUSG00000042179	29.03882	2.013789	3.85	1.82E-09	1.50E-08	Pnliprp1
ENSMUSG00000042254	24.49057	1.703975	3.8453	3.37E-08	2.38E-07	Cilp
ENSMUSG00000042286	35.21977	16.26522	1.1146	5.80E-05	0.000246	Stab1
ENSMUSG00000042303	60.99318	19.05354	1.6786	1.30E-10	1.21E-09	Sgsm3
ENSMUSG00000042306	204.5546	23.23603	3.1381	9.79E-52	4.46E-50	S100a14
ENSMUSG00000042312	33.58707	13.32199	1.3341	1.88E-05	8.71E-05	S100a13
ENSMUSG00000042367	27.98922	10.37876	1.4312	5.36E-05	0.000228	Gjb3
ENSMUSG00000042436	18.54286	4.492299	2.0453	0.000107	0.00043	Mfap4
ENSMUSG00000042446	27.98922	12.39255	1.1754	0.000236	0.000878	Zmym4
ENSMUSG00000042460	141.6954	59.79405	1.2447	1.85E-17	2.91E-16	C1galt1
ENSMUSG00000042485	20.52543	5.731554	1.8404	0.000101	0.000406	Mustn1
ENSMUSG00000042499	16.32705	0	5.0292	3.14E-06	1.69E-05	Hoxd11
ENSMUSG00000042524	37.66883	13.32199	1.4996	1.66E-06	9.33E-06	Sun2
ENSMUSG00000042712	100.9945	40.12088	1.3319	1.22E-13	1.49E-12	Wbp5
ENSMUSG00000042742	29.27206	13.4769	1.119	0.00024	0.00089	B630005N14Rik
ENSMUSG00000042766	13.99461	2.013789	2.7969	0.000139	0.000541	Trim46
ENSMUSG00000042784	78.25321	0	7.9806	8.12E-21	1.53E-19	Muc1
ENSMUSG00000042793	7.580415	0	5.6128	0.001518	0.004477	Lgr6
ENSMUSG00000042810	20.75867	8.210063	1.3382	0.000754	0.00243	Krba1
ENSMUSG00000042834	16.79353	5.266833	1.6729	0.00076	0.002445	Nrep
ENSMUSG00000042943	8.979876	0	5.8572	0.000582	0.00193	Erich3
ENSMUSG00000042988	42.56695	9.604225	2.148	2.05E-09	1.68E-08	Notum
ENSMUSG00000043003	74.63793	30.36174	1.2977	3.07E-10	2.74E-09	Rasef
ENSMUSG00000043029	14.11123	0	4.1874	2.07E-05	9.52E-05	Trpv3
ENSMUSG00000043391	30.5549	4.647206	2.717	2.51E-08	1.80E-07	2510009E07Rik
ENSMUSG00000043461	43.84978	11.46311	1.9356	6.10E-09	4.70E-08	Sptssb
ENSMUSG00000043587	33.70369	15.95541	1.0789	0.000108	0.000431	Pxylp1
ENSMUSG00000043613	14.34448	1.084348	3.7256	2.71E-05	0.000122	Mmp3
ENSMUSG00000043629	26.93963	8.984598	1.5842	3.21E-05	0.000143	1700019D03Rik
ENSMUSG00000043705	35.9195	11.61801	1.6284	1.16E-06	6.66E-06	Capn13
ENSMUSG00000044080	82.80146	36.0933	1.1979	1.75E-10	1.60E-09	S100a1
ENSMUSG00000044162	94.34701	1.084348	6.4431	4.07E-28	1.03E-26	Tnip3
ENSMUSG00000044229	63.44224	0	6.356	1.59E-19	2.75E-18	Nxpe4
ENSMUSG00000044337	15.74394	5.111926	1.6229	0.001307	0.00393	Ackr3
ENSMUSG00000044345	28.68896	12.54746	1.1931	0.000178	0.000676	Marveld1
ENSMUSG00000044461	24.95706	9.449318	1.4012	0.000159	0.000612	Shisa2
ENSMUSG00000044646	18.42624	0	4.5723	8.61E-07	5.07E-06	Zbtb7c
ENSMUSG00000044674	49.91412	5.42174	3.2026	5.41E-14	6.78E-13	Fzd1
ENSMUSG00000044827	52.59642	9.449318	2.4767	1.65E-12	1.84E-11	Tlr1
ENSMUSG00000044986	84.08429	8.829691	3.2514	1.06E-22	2.15E-21	Tst
ENSMUSG00000045045	39.30154	15.18087	1.3723	2.71E-06	1.47E-05	Lrfn4
ENSMUSG00000045545	10.61258	0	6.0982	0.000198	0.000746	Krt14
ENSMUSG00000045573	14.69434	2.323603	2.6608	0.000126	0.000495	Penk
ENSMUSG00000045763	36.73586	15.02597	1.2897	1.07E-05	5.16E-05	Basp1
ENSMUSG00000045896	29.73855	13.16708	1.1754	0.000151	0.000582	Paip2b
ENSMUSG00000045932	45.48249	7.125715	2.6742	1.38E-11	1.42E-10	Ifit2

ENSMUSG00000045954	26.12328	13.01218	1.0055	0.000977	0.003051	Sdpr
ENSMUSG00000046259	9.562985	0	4.2575	0.000442	0.00152	Sprr2h
ENSMUSG00000046314	31.37126	7.745343	2.018	5.48E-07	3.29E-06	Stxbp6
ENSMUSG00000046410	59.24386	19.51826	1.6018	5.73E-10	4.97E-09	Kcnk6
ENSMUSG00000046432	26.70638	10.06895	1.4073	9.07E-05	0.000369	Ngfrap1
ENSMUSG00000046519	236.1591	100.0698	1.2388	6.50E-28	1.63E-26	Golph3l
ENSMUSG00000046546	27.52274	5.731554	2.2636	8.37E-07	4.94E-06	Fam43a
ENSMUSG00000046589	13.52813	0	4.4484	2.63E-05	0.000119	Lrrc8e
ENSMUSG00000046768	34.98653	13.32199	1.393	8.26E-06	4.10E-05	Rhoj
ENSMUSG00000046794	39.6514	13.01218	1.6075	3.80E-07	2.34E-06	Ppp1r3b
ENSMUSG00000046841	102.7438	29.2774	1.8112	5.72E-18	9.33E-17	Ckap4
ENSMUSG00000046971	26.2399	0	7.4042	2.36E-08	1.70E-07	Pla2g4f
ENSMUSG00000047109	9.796229	0	5.9828	0.000338	0.001201	Cldn14
ENSMUSG00000047139	483.164	167.1445	1.5314	2.71E-67	1.69E-65	Cd24a
ENSMUSG00000047562	68.34036	31.44609	1.1199	1.98E-08	1.44E-07	Mmp10
ENSMUSG00000047881	55.51196	25.24982	1.1365	3.48E-07	2.17E-06	Rell1
ENSMUSG00000047907	22.97449	8.210063	1.4846	0.000196	0.000739	Tshz2
ENSMUSG00000047945	246.655	47.86622	2.3654	5.80E-51	2.58E-49	Marcksl1
ENSMUSG00000048058	25.89003	1.394162	4.2149	7.81E-09	5.91E-08	Ldlrad3
ENSMUSG00000048078	39.41816	5.576647	2.8214	1.41E-10	1.30E-09	Tenm4
ENSMUSG00000048126	68.5736	20.4477	1.7457	3.94E-12	4.28E-11	Col6a3
ENSMUSG00000048285	16.21043	5.731554	1.4999	0.001671	0.004866	Frmd6
ENSMUSG00000048373	83.0347	16.88485	2.298	7.29E-18	1.18E-16	Fgfbp1
ENSMUSG00000048612	78.95294	21.22224	1.8954	1.09E-14	1.44E-13	Myof
ENSMUSG00000048644	11.66218	2.323603	2.3274	0.001181	0.003587	Ctxn1
ENSMUSG00000048779	15.62732	4.957019	1.6565	0.001224	0.0037	P2ry6
ENSMUSG00000049047	28.80558	13.94162	1.047	0.000417	0.00144	Armcx3
ENSMUSG00000049103	17.84313	6.196274	1.5259	0.000886	0.002802	Ccr2
ENSMUSG00000049134	13.64475	3.098137	2.1389	0.000706	0.002293	Nrap
ENSMUSG00000049225	21.69165	3.872671	2.4857	5.60E-06	2.88E-05	Pdp1
ENSMUSG00000049313	182.1632	72.3415	1.3323	2.28E-23	4.81E-22	Sorl1
ENSMUSG00000049521	32.42085	9.759132	1.7321	1.99E-06	1.10E-05	Cdc42ep1
ENSMUSG00000049604	154.2906	0	8.2695	3.38E-38	1.12E-36	Hoxb13
ENSMUSG00000049709	11.54556	1.394162	3.0499	0.000366	0.001288	Nlrp10
ENSMUSG00000049723	19.24259	6.351181	1.5992	0.000416	0.00144	Mmp12
ENSMUSG00000049866	27.17287	13.16708	1.0452	0.000614	0.002027	Arl4c
ENSMUSG00000049971	16.0938	2.788323	2.529	8.23E-05	0.000338	Glt1d1
ENSMUSG00000050315	77.43685	37.33255	1.0526	6.57E-09	5.03E-08	Synpo2
ENSMUSG00000050520	144.611	0	8.176	2.73E-36	8.86E-35	Cldn8
ENSMUSG00000050530	24.25733	8.829691	1.458	0.000148	0.000574	Fam171a1
ENSMUSG00000050578	86.7666	13.16708	2.7202	5.75E-21	1.09E-19	Mmp13
ENSMUSG00000050989	16.21043	4.492299	1.8514	0.00053	0.001778	Sepn1
ENSMUSG00000051236	33.93693	6.970809	2.2835	4.02E-08	2.81E-07	Msrb3
ENSMUSG00000051343	15.5107	2.633417	2.5583	0.000103	0.000415	Rab11fip5
ENSMUSG00000051359	46.53209	21.84187	1.0911	4.76E-06	2.47E-05	Ncald
ENSMUSG00000051397	17.1434	0	4.4682	2.19E-06	1.21E-05	Tacstd2
ENSMUSG00000051439	124.6687	11.77292	3.4046	1.10E-33	3.39E-32	Cd14

ENSMUSG00000051579	19.59246	3.717765	2.3978	2.09E-05	9.62E-05	Tceal8
ENSMUSG00000052212	553.2537	18.74373	4.8835	########	########	Cd177
ENSMUSG00000052419	38.36856	19.05354	1.0099	6.22E-05	0.000262	2610001J05Rik
ENSMUSG00000052512	39.76802	12.39255	1.6821	2.05E-07	1.31E-06	Nav2
ENSMUSG00000052516	58.07764	1.239255	5.5504	1.59E-18	2.65E-17	Robo2
ENSMUSG00000052560	22.15814	5.111926	2.1159	1.73E-05	8.12E-05	Cpne8
ENSMUSG00000052698	37.31897	15.02597	1.3125	7.62E-06	3.81E-05	Tln2
ENSMUSG00000052819	47.6983	0	8.2664	6.67E-13	7.66E-12	Best2
ENSMUSG00000052911	35.21977	11.46311	1.6194	1.56E-06	8.85E-06	Lamb2
ENSMUSG00000052957	14.4611	4.337392	1.7373	0.001475	0.004366	Gas1
ENSMUSG00000053141	10.96245	1.703975	2.6856	0.000888	0.002809	Ptprt
ENSMUSG00000053398	47.46506	21.68696	1.13	2.62E-06	1.43E-05	Phgdh
ENSMUSG00000053414	17.60989	5.266833	1.7414	0.000444	0.001525	Hunk
ENSMUSG00000053477	118.8376	52.51342	1.1782	3.33E-14	4.26E-13	Tcf4
ENSMUSG00000053617	44.43289	17.81429	1.3186	9.86E-07	5.74E-06	Sh3pxd2a
ENSMUSG00000053641	20.8753	8.984598	1.2163	0.001249	0.003769	Dennd4a
ENSMUSG00000053646	27.17287	8.210063	1.7267	1.39E-05	6.59E-05	Plxnb1
ENSMUSG00000053702	17.26002	2.94323	2.552	4.28E-05	0.000186	Nebl
ENSMUSG00000054027	17.49327	4.337392	2.0119	0.000188	0.000709	Nt5dc3
ENSMUSG00000054169	203.0385	25.09491	3.0163	3.85E-50	1.66E-48	Ceacam10
ENSMUSG00000054200	13.76137	3.407951	2.0136	0.000919	0.002889	Ffar4
ENSMUSG00000054364	225.3133	95.88734	1.2325	1.38E-26	3.33E-25	Rhob
ENSMUSG00000054423	9.562985	1.394162	2.7781	0.001676	0.004879	Cadps
ENSMUSG00000054675	26.12328	6.351181	2.0402	4.38E-06	2.29E-05	Tmem119
ENSMUSG00000054814	29.38869	14.56124	1.0131	0.000448	0.001537	Usp46
ENSMUSG00000055172	19.35921	5.886461	1.7175	0.000253	0.000932	C1ra
ENSMUSG00000055210	24.14071	0	6.2839	2.31E-08	1.67E-07	Foxd2
ENSMUSG00000055322	108.225	53.59777	1.0138	1.61E-11	1.64E-10	Tns1
ENSMUSG00000055373	27.05625	0	7.4484	1.53E-08	1.12E-07	Fut9
ENSMUSG00000055748	168.8683	2.633417	6.0028	4.77E-50	2.04E-48	Gsdmc4
ENSMUSG00000055799	31.13801	1.394162	4.4812	1.74E-10	1.59E-09	Tcf7l1
ENSMUSG00000055827	17.1434	0	5.0996	1.77E-06	9.89E-06	Gsdmc3
ENSMUSG00000055926	16.0938	4.957019	1.699	0.000901	0.002835	Gm14137
ENSMUSG00000055978	299.1348	124.7	1.2623	1.62E-35	5.17E-34	Fut2
ENSMUSG00000056054	315.8117	4.647206	6.0866	1.14E-91	9.92E-90	S100a8
ENSMUSG00000056071	202.2221	7.125715	4.8268	2.91E-60	1.62E-58	S100a9
ENSMUSG00000056220	61.80954	16.72994	1.8854	8.97E-12	9.41E-11	Pla2g4a
ENSMUSG00000056293	56.21169	1.703975	5.0439	5.15E-18	8.40E-17	Gsdmc2
ENSMUSG00000056429	951.7503	416.0798	1.1937	########	########	Tgoln1
ENSMUSG00000057193	41.05086	17.03975	1.2685	3.87E-06	2.04E-05	Slc44a2
ENSMUSG00000057329	18.42624	7.125715	1.3707	0.001324	0.003974	Bcl2
ENSMUSG00000057465	40.11789	1.239255	5.0167	2.74E-13	3.24E-12	Saa2
ENSMUSG00000057614	16.32705	4.337392	1.9124	0.000418	0.001444	Gnai1
ENSMUSG00000058057	7.93028	0	5.6779	0.001191	0.003611	Mettl7a3
ENSMUSG00000058070	39.18491	17.03975	1.2014	1.10E-05	5.33E-05	Eml1
ENSMUSG00000058230	136.4475	27.41851	2.3151	1.73E-28	4.51E-27	Arhgap35
ENSMUSG00000058297	272.7783	10.99839	4.6324	3.80E-80	2.87E-78	Spock2

ENSMUSG00000058427	19.00935	1.549069	3.6172	1.61E-06	9.08E-06	Cxcl2
ENSMUSG00000058755	7.697037	0	4.6348	0.00145	0.004305	Osm
ENSMUSG00000058799	156.0399	70.32771	1.1498	8.69E-18	1.39E-16	Nap1l1
ENSMUSG00000058908	118.9542	56.23119	1.081	3.28E-13	3.85E-12	Pla2g2a
ENSMUSG00000059149	212.0184	98.67567	1.1034	9.38E-23	1.91E-21	Mfsd4
ENSMUSG00000059173	11.31231	2.013789	2.4899	0.001033	0.003203	Pde1a
ENSMUSG00000059430	423.1038	89.38126	2.243	1.57E-82	1.25E-80	Actg2
ENSMUSG00000059713	34.86991	11.92783	1.5477	2.89E-06	1.57E-05	Rcan3
ENSMUSG00000060012	71.02266	34.85404	1.027	3.99E-08	2.80E-07	Kif13b
ENSMUSG00000060227	24.14071	9.604225	1.3297	0.000292	0.001062	Casc4
ENSMUSG00000060519	37.90207	14.56124	1.3801	3.84E-06	2.03E-05	Tor3a
ENSMUSG00000060600	17.60989	5.266833	1.7414	0.000444	0.001525	Eno3
ENSMUSG00000061119	52.82966	21.99677	1.2641	1.69E-07	1.10E-06	Prcp
ENSMUSG00000061517	19.70908	1.394162	3.8214	7.56E-07	4.47E-06	Sox21
ENSMUSG00000061762	153.5909	9.449318	4.0227	3.16E-44	1.19E-42	Tac1
ENSMUSG00000061780	12.59515	2.94323	2.0974	0.00125	0.003772	Cfd
ENSMUSG00000061808	25.1903	4.337392	2.538	8.10E-07	4.78E-06	Ttr
ENSMUSG00000061887	89.91538	33.76969	1.4128	6.21E-13	7.15E-12	Ssbp3
ENSMUSG00000062078	24.95706	8.984598	1.4739	0.00011	0.000438	Qk
ENSMUSG00000062124	10.37934	0	4.3756	0.000238	0.000884	Defb45
ENSMUSG00000062127	72.07225	26.02435	1.4696	5.24E-11	5.05E-10	Cttnbp2nl
ENSMUSG00000062210	61.69292	14.56124	2.083	1.07E-12	1.20E-11	Tnfaip8
ENSMUSG00000062421	49.33101	23.54584	1.067	3.15E-06	1.69E-05	Arf2
ENSMUSG00000062591	11.19569	2.168696	2.368	0.001373	0.004101	Tubb4a
ENSMUSG00000062995	31.13801	8.984598	1.7932	2.21E-06	1.21E-05	Ica1
ENSMUSG00000063142	12.94502	1.394162	3.2149	0.000126	0.000495	Kcnma1
ENSMUSG00000063531	10.02947	1.084348	3.2093	0.000742	0.002396	Sema3e
ENSMUSG00000063727	61.22643	8.674784	2.8193	1.32E-15	1.88E-14	Tnfrsf11b
ENSMUSG00000063903	309.9807	29.12249	3.412	3.29E-81	2.53E-79	Klk1
ENSMUSG00000064080	23.79084	5.266833	2.1754	6.64E-06	3.37E-05	Fbln2
ENSMUSG00000064254	125.485	14.71615	3.092	3.98E-32	1.17E-30	Ethe1
ENSMUSG00000065954	37.90207	15.95541	1.2482	1.07E-05	5.16E-05	Tacc1
ENSMUSG00000066090	22.62462	0	5.4998	4.15E-08	2.90E-07	Insl5
ENSMUSG00000066357	35.45302	11.77292	1.5904	1.77E-06	9.88E-06	Wdr6
ENSMUSG00000066705	49.33101	12.23764	2.0112	3.57E-10	3.17E-09	Fxyd6
ENSMUSG00000067006	107.5253	20.91243	2.3622	3.83E-23	7.98E-22	Serpinb5
ENSMUSG00000067818	285.0236	120.9823	1.2363	2.77E-33	8.40E-32	Myl9
ENSMUSG00000067889	15.74394	2.47851	2.6673	7.09E-05	0.000296	Sptbn2
ENSMUSG00000068040	150.4421	66.45504	1.1788	1.39E-17	2.21E-16	Tm9sf4
ENSMUSG00000068086	18.7761	0	6.9214	1.41E-06	8.04E-06	Cyp2d9
ENSMUSG00000068196	12.94502	1.549069	3.0629	0.000158	0.000608	Col8a1
ENSMUSG00000068220	74.9878	17.65938	2.0862	3.95E-15	5.46E-14	Lgals1
ENSMUSG00000068551	19.35921	4.492299	2.1075	6.09E-05	0.000257	Zfp467
ENSMUSG00000068699	44.89938	15.8005	1.5067	1.59E-07	1.03E-06	Flnc
ENSMUSG00000068735	145.0775	54.52721	1.4118	6.41E-20	1.14E-18	Trp53i11
ENSMUSG00000068748	29.38869	2.323603	3.6608	2.20E-09	1.79E-08	Ptprz1
ENSMUSG00000068923	17.95975	6.660995	1.431	0.001215	0.003676	Syt11

ENSMUSG00000069094	52.36317	20.2928	1.3676	6.42E-08	4.41E-07	Pde7a
ENSMUSG00000069114	15.74394	1.549069	3.3453	1.89E-05	8.75E-05	Zbtb10
ENSMUSG00000069456	67.99049	20.4477	1.7334	5.69E-12	6.08E-11	Rdh16
ENSMUSG00000069516	236.3923	42.13466	2.4881	7.71E-51	3.39E-49	Lyz2
ENSMUSG00000069792	23.44098	2.013789	3.541	1.16E-07	7.73E-07	Wfdc17
ENSMUSG00000069808	42.21708	10.37876	2.0242	5.95E-09	4.59E-08	Fam57a
ENSMUSG00000070056	26.00665	12.70236	1.0338	0.000858	0.002725	Mfhas1
ENSMUSG00000070436	71.02266	29.12249	1.2861	9.67E-10	8.20E-09	Serpinh1
ENSMUSG00000071711	32.88734	13.94162	1.2381	4.39E-05	0.000191	Mpst
ENSMUSG00000071847	35.56964	1.549069	4.5212	8.55E-12	8.99E-11	Apcdd1
ENSMUSG00000071856	14.81096	4.337392	1.7718	0.001167	0.003552	Mcc
ENSMUSG00000071984	24.25733	1.394162	4.121	2.59E-08	1.85E-07	Fndc1
ENSMUSG00000072235	37.43559	13.94162	1.425	3.13E-06	1.69E-05	Tuba1a
ENSMUSG00000072812	11.19569	1.549069	2.8535	0.000599	0.001979	Ahnak2
ENSMUSG00000072941	30.5549	9.604225	1.6697	5.69E-06	2.92E-05	Sod3
ENSMUSG00000072999	12.01204	0	3.955	0.000101	0.000406	Gm15401
ENSMUSG00000073043	31.25463	11.3082	1.4667	1.57E-05	7.42E-05	Atoh1
ENSMUSG00000073598	19.59246	6.660995	1.5565	0.000437	0.001508	1700066B19Rik
ENSMUSG00000073599	24.14071	10.22385	1.2395	0.000461	0.001575	Ecscr
ENSMUSG00000073608	9.91285	1.549069	2.6779	0.001592	0.004667	Gm6086
ENSMUSG00000073758	27.75598	13.32199	1.059	0.000495	0.00168	Sh3d21
ENSMUSG00000073987	130.0333	37.48746	1.7944	3.79E-22	7.55E-21	Ggh
ENSMUSG00000074115	1222.546	31.13628	5.2951	0	0	Saa1
ENSMUSG00000074364	32.30423	10.53367	1.6167	4.30E-06	2.25E-05	Ehd2
ENSMUSG00000074445	244.9057	97.74623	1.3251	1.06E-30	3.02E-29	Sprr2a3
ENSMUSG00000074457	253.4191	60.87839	2.0575	9.59E-47	3.79E-45	S100a16
ENSMUSG00000074480	25.77341	4.802113	2.4241	9.52E-07	5.56E-06	Mex3a
ENSMUSG00000074653	23.79084	10.99839	1.1131	0.000958	0.003002	Lrrc31
ENSMUSG00000074656	709.5268	269.8477	1.3947	8.38E-90	7.08E-88	Eif2s2
ENSMUSG00000074743	23.5576	11.46311	1.0392	0.00147	0.004352	Thbd
ENSMUSG00000074749	20.29219	7.590436	1.4187	0.000615	0.002027	Kiz
ENSMUSG00000074794	74.87118	34.69914	1.1095	4.93E-09	3.84E-08	Arrdc3
ENSMUSG00000074892	875.1297	388.3515	1.1721	1.02E-93	9.02E-92	B3galt5
ENSMUSG00000074934	157.6726	40.89541	1.9469	2.11E-28	5.44E-27	Grem1
ENSMUSG00000075602	661.7119	32.99516	4.3259	########	########	Ly6a
ENSMUSG00000076431	160.3549	44.61317	1.8457	1.37E-27	3.39E-26	Sox4
ENSMUSG00000076441	27.8726	6.506088	2.099	1.57E-06	8.87E-06	Ass1
ENSMUSG00000076612	35.45302	11.92783	1.5716	2.02E-06	1.11E-05	Ighg2c
ENSMUSG00000078161	14.92759	0	5.5904	8.54E-06	4.22E-05	BC007180
ENSMUSG00000078234	13.52813	2.47851	2.4484	0.000364	0.00128	Klhdc7a
ENSMUSG00000078670	36.73586	15.02597	1.2897	1.07E-05	5.16E-05	Fam174b
ENSMUSG00000078706	34.05356	0	6.0897	2.61E-11	2.59E-10	Gm53
ENSMUSG00000079018	64.02535	8.984598	2.8331	2.68E-16	3.97E-15	Ly6c1
ENSMUSG00000079020	20.40881	8.36497	1.2868	0.001044	0.003225	Slc45a4
ENSMUSG00000079037	65.8913	13.01218	2.3402	1.10E-14	1.46E-13	Prnp
ENSMUSG00000079339	268.2301	107.1955	1.3232	2.02E-33	6.15E-32	Gm14446
ENSMUSG00000079442	21.69165	3.562858	2.606	3.77E-06	1.99E-05	St6galnac4

ENSMUSG00000079445	295.1697	68.15902	2.1146	2.12E-55	1.07E-53	B3gnt7
ENSMUSG00000084174	47.11519	0	8.2486	8.70E-13	9.86E-12	Sycn
ENSMUSG00000089774	36.85248	15.33578	1.2649	1.25E-05	5.97E-05	Slc5a3
ENSMUSG00000089901	25.65679	11.61801	1.143	0.000514	0.001734	Gm8113
ENSMUSG00000090176	7.93028	0	3.9874	0.001554	0.00457	Cd200r2
ENSMUSG00000091337	28.68896	11.61801	1.3041	9.15E-05	0.000371	Eid1
ENSMUSG00000094559	82.91808	1.239255	6.0641	2.24E-25	5.05E-24	Cyp2d34
ENSMUSG00000094806	25.1903	0	5.3453	6.95E-09	5.29E-08	Cyp2d10
ENSMUSG00000095742	23.09111	10.84348	1.0905	0.001274	0.003837	Cd99
ENSMUSG00000096001	305.1992	4.182485	6.1892	3.12E-88	2.61E-86	2610528A11Rik
ENSMUSG00000098557	69.27333	32.37553	1.0974	2.18E-08	1.57E-07	Kctd12
